# CircEYA3 aggravates intervertebral disc degeneration through the miR-196a-5p/EBF1 axis and NF-κB signaling

**DOI:** 10.1038/s42003-024-06055-2

**Published:** 2024-03-30

**Authors:** Tianfu Wang, Xiaobing Yan, Dehui Song, Yingxia Li, Zhengwei Li, Dapeng Feng

**Affiliations:** 1 Department of Spinal Surgery, The Second Hospital of Dalian Medical University, No. 467, Zhongshan Road, Shahekou District, Dalian, 116023 Liaoning China; 2 Department of Orthopaedics, Tangdu Hospital, Fourth Military Medical University, Xi’an, 710038 China; 3 Department of Orthopaedics, Dandong Central Hospital, 338 Jinshan Street, Zhenxing District, Dandong, 118000 Liaoning China

**Keywords:** miRNAs, Inflammation, Cell signalling, Apoptosis

## Abstract

Intervertebral disc degeneration (IDD) is a well-established cause of disability, and extensive evidence has identified the important role played by regulatory noncoding RNAs, specifically circular RNAs (circRNAs) and microRNAs (miRNAs), in the progression of IDD. To elucidate the molecular mechanism underlying IDD, we established a circRNA/miRNA/mRNA network in IDD through standardized analyses of all expression matrices. Our studies confirmed the differential expression of the transcription factors early B-cell factor 1 (EBF1), circEYA3, and miR-196a-5p in the nucleus pulposus (NP) tissues of controls and IDD patients. Cell proliferation, apoptosis, and extracellular mechanisms of degradation in NP cells (NPC) are mediated by circEYA3. MiR-196a-5p is a direct target of circEYA3 and EBF1. Functional analysis showed that miR-196a-5p reversed the effects of circEYA3 and EBF1 on ECM degradation, apoptosis, and proliferation in NPCs. EBF1 regulates the nuclear factor kappa beta (NF-кB) signalling pathway by activating the IKKβ promoter region. This study demonstrates that circEYA3 plays an important role in exacerbating the progression of IDD by modulating the NF-κB signalling pathway through regulation of the miR196a-5p/EBF1 axis. Consequently, a novel molecular mechanism underlying IDD development was elucidated, thereby identifying a potential therapeutic target for future exploration.

## Abstract (web summary)

An in vivo study demonstrates that circEYA3 plays an important role in exacerbating the progression of intervertebral disc degeneration by modulating the NF-κB signalling pathway through regulation of the miR196a-5p/EBF1 axis.

## Introduction

Intervertebral disc degeneration (IDD), common in the ageing population, is responsible for an increased obesity rate worldwide and is a leading cause of disability^1–3^. As IDD is aggravated over a period of time, patients suffer from mechanical pain, loss of myodynamia and reduced quality of life^4,5^. Surgical therapy places a major burden on the wider social economy. Because the molecular mechanisms of pathogenesis have not been fully elucidated, nonsurgical therapeutic options are limited and suboptimal^6–8^. The degeneration of the extracellular matrix (ECM) and reduction in nucleus pulposus cells cause IDD to worsen^9–11^. Thus, there is an urgent need to elucidate the molecular mechanisms of IDD.

Recent research has revealed that endogenous noncoding RNAs (ncRNAs), encompassing circular RNAs (circRNAs) and microRNAs (miRNAs), actively engage in a multitude of biological and pathological processes^12–16^. Notably, circRNAs have a complete circular structure, are stable, and show biological functional diversity. Several studies have indicated that circRNAs act as sponges for miRNAs and specifically bind to them^17–19^. The RNA-induced silencing complex is involved in the development of numerous diseases by regulating the expression of downstream target genes of miRNAs. However, the roles of circRNAs and the circRNA/miRNA axis in IDD progression remain unclear.

Early B-cell factor-1 (EBF1) plays a crucial role as a B-cell-specific transcription factor in the pathogenesis of several diseases. Its functional necessity in inducing various phenotypic mutations in vitro and in vivo underscores its importance in the development of immune-mediated inflammatory diseases^20–22^. The nuclear factor kappa beta (NF-κB) signalling pathway is a classic prosurvival signalling pathway in cells. The abnormal phosphorylation of IκBα disassociates NF-κB from IκB and allows NF-κB to translocate to the nucleus, leading to the activation of NF-κB-related genes and pathological changes in cells^23–25^. Unnatural modulation of the NF-κB signalling pathway can result in cell death and abnormal cell function in NPCs^26–28^. A previous study revealed that EBF1 is involved in the activation of the NF-κB signalling pathway^29^. Nevertheless, the function of EBF1 in NPCs remains relatively unknown.

Through bioinformatics analysis of NPCs in IDD, we constructed a regulatory network for circRNAs/miRNAs/mRNAs. Further experiments were carried out to elucidate the mechanism of circEYA3 and its downstream miR-196a-5p/EBF1/IKKβ axis.

## Results

### CircEYA3 modulated the progression of IDD

**Fig. 1:**
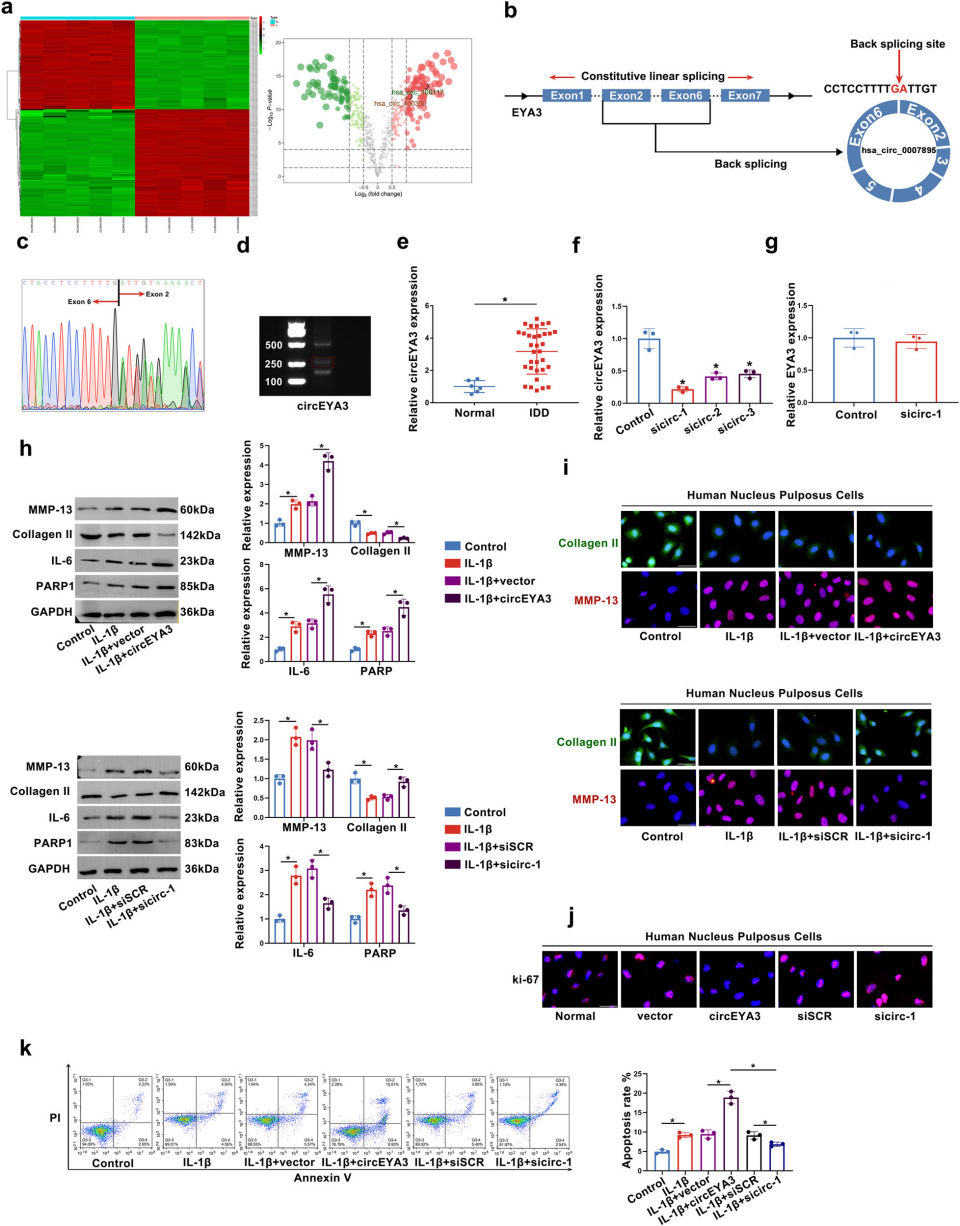
CircEYA3 regulates the progression of NP degeneration. **a** Differential expression of circRNA in IDD. **b** Diagram illustrating the formation of circEYA3 from exons 2–6 of EYA3. **c** The existence of circEYA3 was confirmed by Sanger sequencing. **d** The expression of circEYA3 was analysed by RT-PCR. **e, f** Expression levels of circEYA3 detected by q-PCR. **g** The expression of EYA3 was analysed by q-PCR. **h** The expression levels of MMP-13, collagen II, IL-6 and PARP1 were analysed in by western blot. **i** The expression levels of MMP-13 and collagen II were detected in NPCs by immunofluorescence staining. Scale bar, 50 μm. **j** Ki67 immunofluorescence staining was used to identify the proliferative capability. Scale bar, 50 μm. **k** Apoptosis rate was evaluated by flow cytometry. Data were means ± SD of three independent assays (*P < 0.05).

Initially, standardized analyses of all expression matrices were performed. The heatmap depicts the differential expression of circRNAs in IDD (Fig. 1a). CircRNAs with logFC > 1 were selected for further investigation. As shown in Fig. 1b, c, circEYA3 (hsa_circ_0007895) is formed by reverse splicing from exon 2 to exon 6 of EYA3. CircEYA3 was stably expressed in nucleus pulposus, as shown by PCR validation of reverse splicing sequences (Fig. 1d). The results of qPCR analysis showed that the expression level of circEYA3 in the IDD group was significantly higher than that in the normal group (Fig. 1e). Three interfering RNAs that were designed to downregulate circEYA3 in NPCs significantly downregulated the expression of circEYA3 (Fig. 1f). Moreover, the utilization of the initial interfering RNA, sicirc-1, in the subsequent experiment exhibited no discernible impact on the expression level of EYA3 in NPCs, as depicted in Fig. 1g. Western blotting and immunofluorescence analyses revealed that circEYA3 facilitated IL-1β-induced ECM degradation, the inflammatory response, and apoptosis in NPCs. In contrast, downregulation of circEYA3 expression reversed these regulatory effects (Fig. 1h, i and Supplementary Fig. 1a). Additionally, we determined the proliferative ability of NPC by Ki-67 immunofluorescence staining (Fig. 1j). Flow cytometry analysis showed that circEYA3 promoted IL-1β-induced NPC apoptosis (Fig. 1k). The aforementioned findings suggest that aberrant circEYA3 plays a role in the progression of IDD through its regulation of ECM degradation, proliferation, and apoptosis in NPCs.

### Construction of the circRNA/miRNA/mRNA network in IDD

**Fig. 2:**
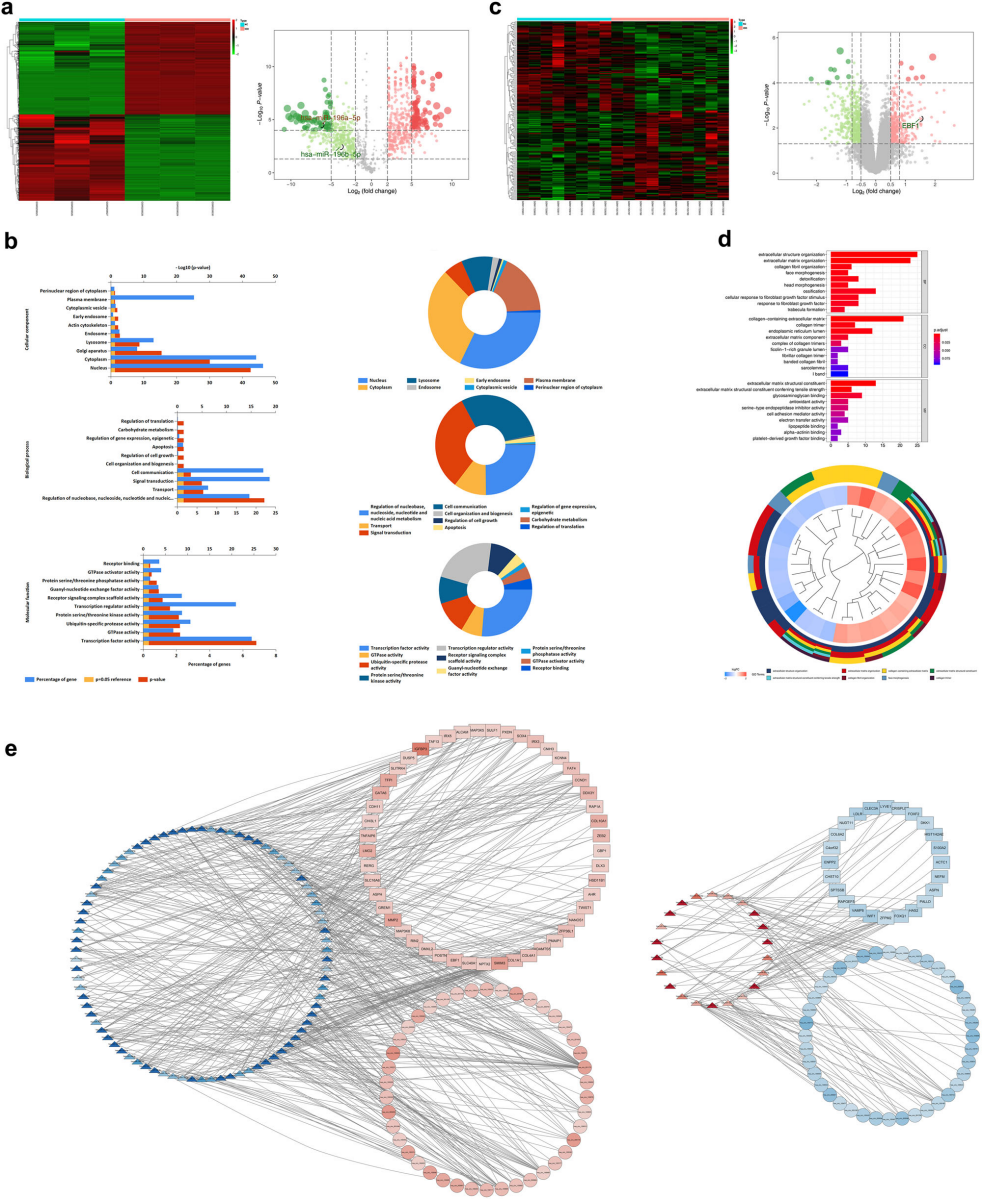
CircRNA/miRNA/mRNA regulatory network in IDD. **a** Differential expression of miRNA in IDD. **b** GO enrichment analysis of miRNA differential expression in IDD. **c** Differential expression of mRNA in IDD. **d** GO enrichment analysis of mRNA differential expression in IDD. **e** The pairing relationship between miRNAs, circRNAs and mRNAs in IDD.

MiRNAs with logFC > 2 (Fig. 2a) and mRNAs with logFC > 0.6 (Fig. 2c) were selected as genes of the network. Furthermore, Gene Ontology (GO) analysis was performed to study the differences in cellular components (CC), molecular functions (MF), and biological processes (BP) between the IDD and normal groups based on the variant expression matrices of miRNAs (Fig. 2b) and mRNAs (Fig. 2d). To enhance comprehension of the interplay among circRNAs, miRNAs, and mRNAs, we established a comprehensive circRNA/miRNA/mRNA network through the integration of circRNA/miRNA/mRNA interactions (Fig. 2e, Supplementary Data 5).

### CircEYA3 regulated the expression of EBF1 by acting as a sponge for miR-196a-5p

**Fig. 3:**
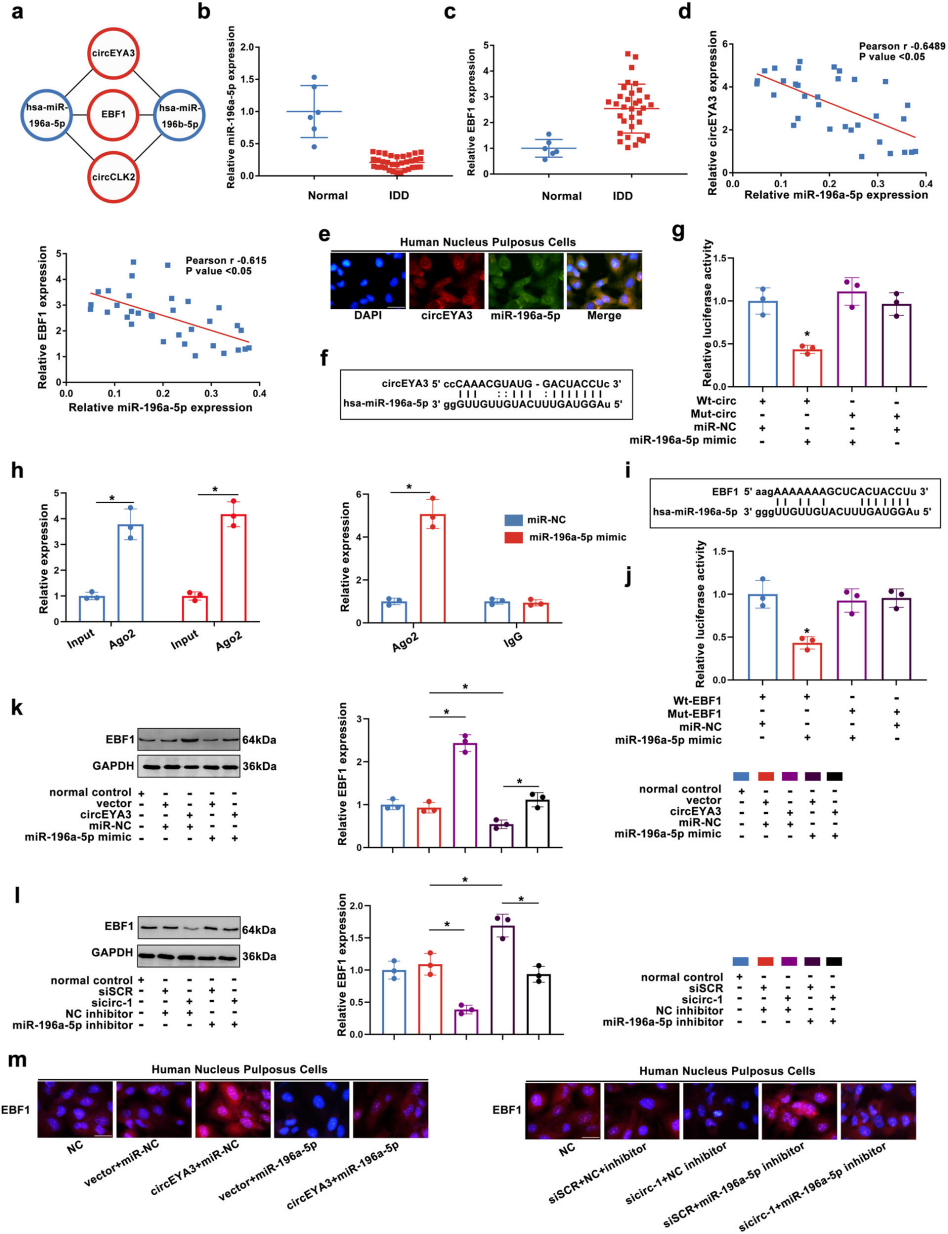
CircEYA3 acts as a sponge for mir-196a-5p to regulate EBF1 expression. **a** Regulatory network of circEYA3/ mir-196a-5p /EBF1. **b, c** The expression levels of mir-196a-5p and EBF1 were analysed by q-PCR. **d** The correlation analysis of the expressions of circEYA3 and EBF1. **e** Localization of circEYA3 and mir-196a-5p was determined by FISH. Scale bar, 50 μm. **f** Base pairing information of circEYA3 and mir-196a-5p. **g** CircEYA3 was a direct target of mir-196a-5p in NPC by dual luciferase reporter assay. **h** Anti-Argonaute 2 (Ago2) RIP analysis verified the endogenous phase interaction between circEYA3 and mir-195a-5p. **i** Detailed base pairing information of EBF1 and mir-196a-5p. **j** EBF1 was confirmed as a direct target of mir-196a-5p by dual luciferase reporter gene assay in NPC. **k**–**m** CircEYA3 and mir-196a-5p co-regulate the expression level of EBF1 in NPC. Scale bar, 50 μm. Data were means ± SD of three independent assays (*P < 0.05).

Based on the aforementioned findings, the circRNA EYA3/miR-196a-5p/EBF1 network was chosen for further investigation (Fig. 3a). The results of quantitative polymerase chain reaction (qPCR) analysis demonstrated a significant decrease in the expression of miR-196a-5p in intervertebral disc degeneration (IDD) (Fig. 3b). Conversely, the expression of EBF1 in IDD was found to be elevated (Fig. 3c). Correlation analysis revealed a negative association between the expression levels of circEYA3 and EBF1 and that of miR-196a-5p (Fig. 3d). The locations of circEYA3 and miR-196a-5p in the cytoplasm were determined by fluorescence in situ hybridization (FISH) (Fig. 3e). The binding sites between circEYA3 and miR-196a-5p were predicted using an online website (starbase.sysu.edu.cn/) (Fig. 3f ). Dual-luciferase reporter gene analysis confirmed that circEYA3 was a direct target of miR-196a-5p in NPC (Fig. 3g). Furthermore, the endogenous interaction between circEYA3 and miR-195a-5p was confirmed through anti-Argonaute 2 (Ago2) RIP analysis, as depicted in Fig. 3h. Additionally, EBF1 was deemed to be a direct target of miR-196a-5p in NPCs through bioinformatics and dual-luciferase reporter gene analyses (Fig. 3i, j). The expression of EBF1 in NPCs was jointly regulated by circEYA3 and miR-196a-5p (Fig. 3k, l). Moreover, the results of the immunofluorescence analysis were similar to those of Western blotting. (Fig. 3m). As described above, circEYA3 sponges miR-196a-5p and indirectly regulates the expression of EBF1 in NPC.

### The circEYA3/miR-196a-5p/EBF1 network mediated IDD progression via ECM degradation, proliferation, and apoptosis

**Fig. 4:**
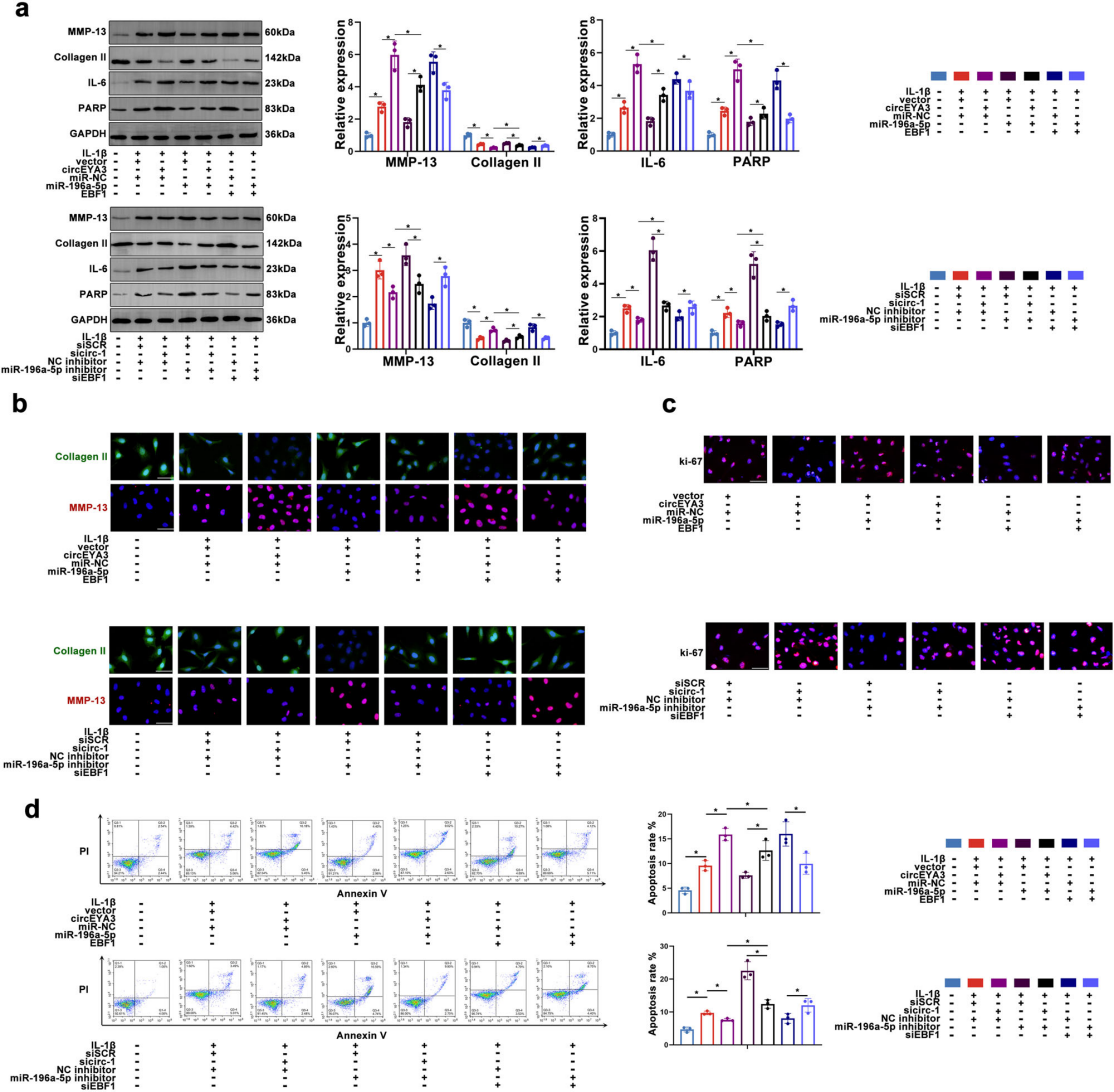
CircEYA3/mir-196a-5p/EBF1 axis mediates ECM degradation, proliferation and apoptosis of NPC. **a**, **b** The expression levels of NP degeneration related proteins analysed by Western blot and immunofluorescence staining. Scale bar, 50 μm. **c** The proliferation of NPC was identified by Ki67 immunofluorescence staining. Scale bar, 50 μm. **d** Apoptosis rate of NPCs was evaluated by flow cytometry. Data were means ± SD of three independent assays (*P < 0.05).

Combined analyses of the aforementioned data were used to perform rescue experiments to analyse the function of the circEYA3/miR-196a-5p/EBF1 axis in IDD progression. Firstly, it has been demonstrated that siEBF1 could significantly decrease the EBF1 expression level (Supplementary Fig. 1b). As shown in Fig. 4a, b and Supplementary Fig. 1c, overexpression of circEYA3 and EBF1 promoted ECM degradation, the inflammatory response, and apoptosis in NPCs, whereas overexpression of miR-196a-5p reversed these effects. However, the levels of MMP-13, IL-6, ADAMTS-5, and PARP in NPCs were decreased as a result of the depletion of circEYA3 or EBF1 levels, while the suppression of miR-196a-5p successfully halted this effect. Furthermore, the miR-196a-5p inhibitor nullified the proliferative and antiapoptotic effects caused by sicirc-1/siEBF1 while effectively reversing the antiproliferative and apoptotic effects mediated by circEYA3/EBF1 (Fig. 4c, d).

### The circEYA3/miR-196a-5p/EBF1 network mediated IDD progression in vivo

**Fig. 5:**
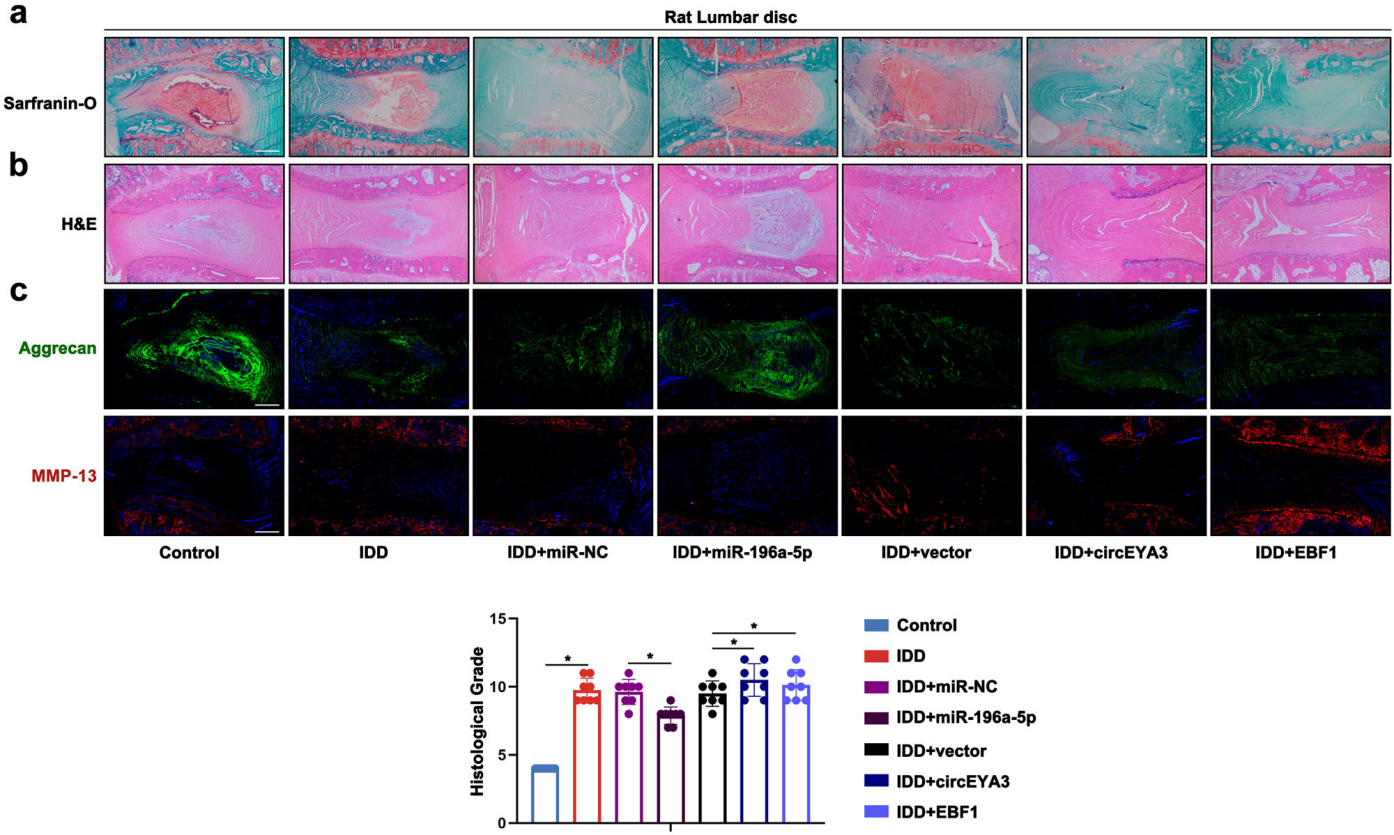
The circEYA3/miR-196a-5p/EBF1 network mediated IDD progression in vivo. **a** Safranin-O staining of Rat lumbar disc structures. Scale bar, 500 μm. **b** HE staining of Rat lumbar disc structures. Scale bar, 500 μm. **c** Immunofluorescence staining of lumbar intervertebral disc in Rats. Scale bar, 500 μm. Data were means ± SD of three independent assays (*P < 0.05).

Furthermore, the regulatory role of the circEYA3/miR-196a-5p/EBF1 axis in IDD progression was investigated in Sprague‒Dawley (SD) rats. Various reagents were injected as treatments into the lumbar discs of SD rats. The vertebral bodies of the SD rats were harvested by surgery and analysed eight weeks after treatment. As shown in Fig. 5a, b, complete intervertebral discs were determined by safranin O and fast green staining and HE staning. The group with overexpression of circEYA3/EBF1 exhibited severe degeneration of NP tissues, which promoted IDD severity compared with that of the vector group (Fig. 5c). The data presented here demonstrate that the circEYA3/miR-196a-5p/EBF1 axis plays a role in the breakdown of the ECM, the inflammatory response, cell growth, and programmed cell death during the progression of IDD.

### EBF1 promoted the activation of the NF-κB signalling pathway

**Fig. 6:**
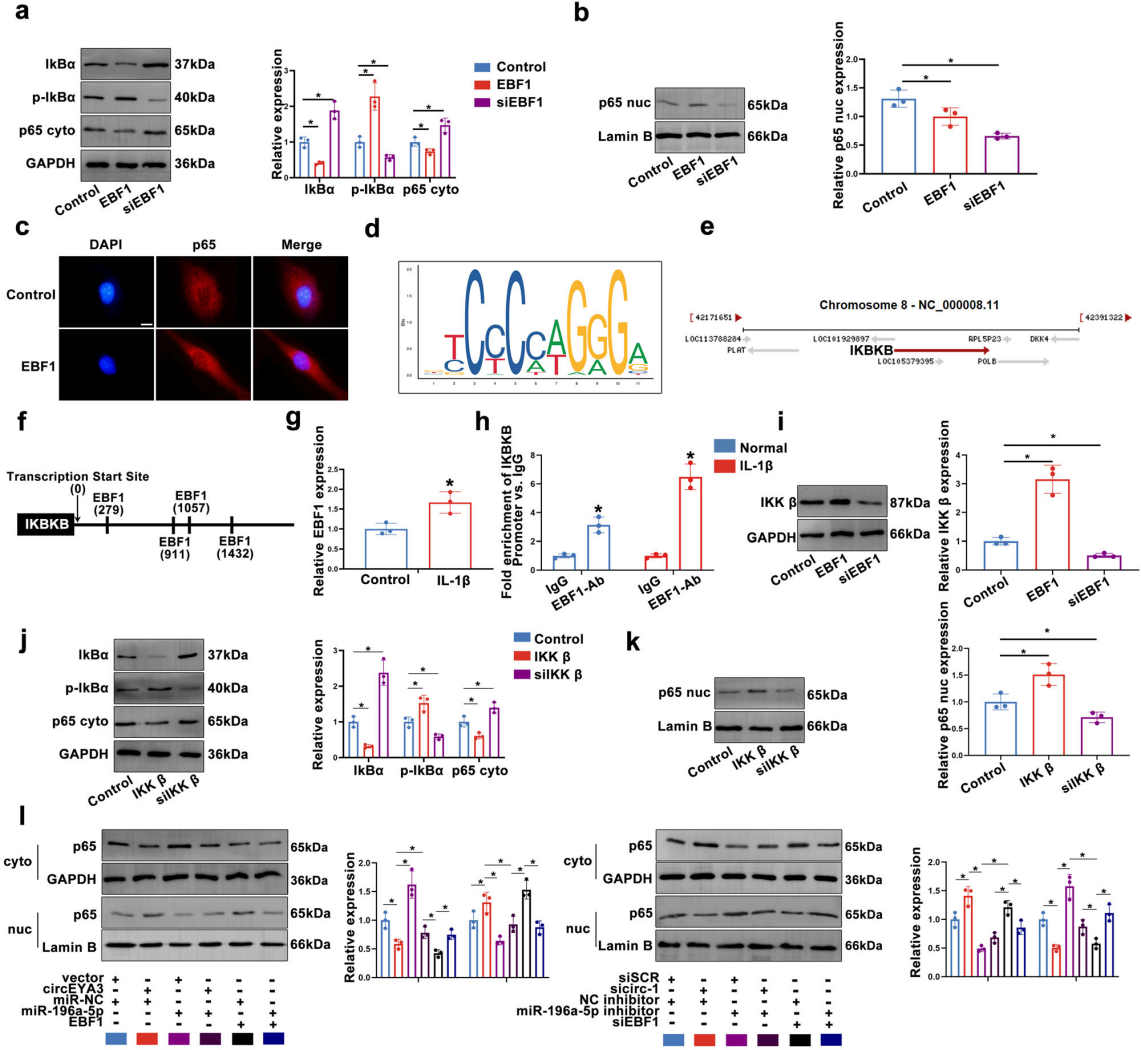
EBF1 promoted the activation of NF-κB signaling pathway by promoting IKBKB expression. **a**, **b** Western blot was used to detect the regulatory effect of EBF1 on NF-κB signaling pathway. **c** Immunofluorescence assay was used to detect the function of EBF1 on P65 enucleation. Scale bar, 10 μm. **d** Motif diagram of transcription factor EBF1. **e** Schematic diagram of IKBKB transcription direction. **f** The predicted possible binding sites of IKBKB and EBF1. **g** The binding of EBF1 to the promoter region of IKBKB was detected by PCR. **h** Q-PCR showed that EBF1 binds to the promoter region of IKBKB. **i** Modulation of iKKβ expression by EBF1 was detected by Western blot. **j**, **k** The regulatory effect of IKKβ on NF-κB signaling pathway was detected by Western blot. **l** The regulatory effect of circEYA3/ mir-196a-5p /EBF1 axis on NF-κB signaling pathway was detected by Western blot.

As shown in Fig. 6a and b, EBF1 promoted the phosphorylation of IκBα, which promoted the entry of p65 into the nucleus. Immunofluorescence analysis provided strong evidence for this phenomenon (Fig. 6c). To understand the exact mechanism of EBF1 and the NF-κB signalling pathway, we generated a motif model illustration (Fig. 6d). According to the Chip-seq database of the online website, there may be binding sites between EBF1 and the promoter region of the inhibitor of nuclear factor kappa B kinase beta (IKBKB) (Supplementary Fig. 2). As shown in Fig. 6e and f, based on the transcription direction of IKBKB, we predicted four possible binding sites. The results of the ChIP experiment verified that EBF1 could bind to the promoter region of IKBKB in IL-1β-treated NPCs (Fig. 6g, h). Western blotting indicated that EBF1 promoted the expression of IKKβ (Fig. 6i). Furthermore, upregulated IKKβ promoted the phosphorylation of IκBα and played a role in the entry of p65 into the nucleus. As shown in Fig. 6j, k, p65 was activated and entered the nucleus in transfected circEYA3 NPCs. However, these effects were effectively reversed by cotransfection of NPCs with miR-196a-5p. The miR-196a-5p inhibitor abolished the inhibitory effect mediated by sicirc-1 on NF-кB (Fig. 6l). These data show that the circEYA3/miR-196a-5p/EBF1 axis modulates the activation of the NF-кB pathway.

### Molecular mechanism of circEYA3 and the miR-196a-5p/EBF1/IKKβ axis in NPCs

**Fig. 7:**
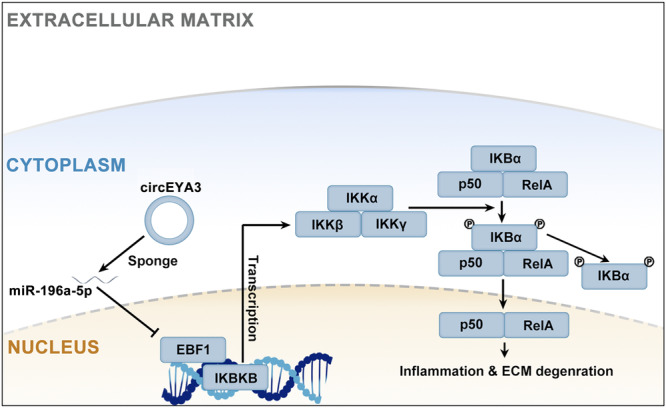
Specific molecular mechanism of circEYA3 and Mir-196a-5p/EBF1/IKKβ axis in NPC. The competitive binding of CircEYA3 on miR-196a-5p leads to the modulation of EBF1 expression, consequently disrupting the normal activity of the NF-κB signaling pathway and influencing the progression of IDD.

The exact molecular mechanism uncovered by this study is shown in Fig. 7. As a sponge of miR-196a-5p, circEYA3 inhibits its inhibitory effect on EBF1 in NPCs. CircEYA3 plays a role in the development of IDD and influences the function of the NF-κB signalling pathway through its regulation of the miR196a-5p/EBF1 axis.

## Discussion

Intervertebral disc degeneration is a degenerative disease that occurs with advancing age. The degeneration of the nucleus pulposus within the intervertebral disc is initiated earlier than the degeneration of muscular and osseous tissues^30^. Intervertebral disc degeneration (IDD) can result in a reduction in intervertebral disc height and subsequently contribute to the development of diverse spinal pathologies due to alterations in mechanical characteristics. Presently, low back pain ranks as the fifth most prevalent cause for medical intervention globally, causing considerable discomfort for numerous patients. These conditions not only substantially diminish patients’ quality of life but also impose a substantial strain on healthcare resources. Consequently, research endeavours aimed at comprehending the fundamental mechanisms underlying IDD, with the ultimate goal of offering novel therapeutic interventions, are urgently needed.

NcRNAs, including miRNAs, long ncRNAs (lncRNAs), and recently discovered circRNAs, play an important role in a variety of cellular processes as regulatory elements encoded by the genome^31–33^. Previous studies have shown that the upregulation of circ-4099 in NPCs may act as a self-protective mechanism against the development of IDD by regulating the miR-616-5p/Sox9 pathway^34^. Yu et al. aimed to establish a circRNA/miRNA regulatory network to investigate the impact of downstream mRNA on IDD^35^. However, unlike this study, the mRNA data was not integrated. Similarly, other studies constructed ceRNA research networks using GEO databases, but the circRNA, mRNA, and miRNA databases chosen in this study differed from those utilized in the aforementioned studies^13,35,36^. Future research could summarize these studies to obtain more precise potential regulatory sites. Liang et al. showed that circEYA3 promotes the progression of PDAC^37^. In current study, circEYA3 was confirmed to be present at high levels in IDD. Through functional experiments, we found that circEYA3 promoted IL-1β-induced ECM degradation and apoptosis while inhibiting NPC proliferation. This result indicates that circEYA3 plays a critical role in IDD progression.

CircRNAs are a type of single-stranded RNA molecule that adopts a covalently closed ring structure, thereby exerting regulatory control over downstream pathways by functioning as competing endogenous RNAs (ceRNAs)^36,38–40^. A previous study showed that circRNA_104670 regulates NPC survival and promotes IDD development by sponging miR-17-3p^41^. This study found that the expression levels of circEYA3 and EBF1 in NPCs are negatively correlated with miR-196a-5p. Moreover, the results of dual-luciferase analysis proved that circEYA3 could sponge miR-196a-5p, which regulates the expression of downstream genes. Similarly, the binding between miR-196a-5p and EBF1 mRNA specifically inhibited the expression level of EBF1. Furthermore, circEYA3 and miR-196a-5p coregulated the expression of EBF1 in NPC. The rescue experiments determined that circEYA3/miR-196a-5p/EBF1 mediated IDD in numerous aspects, including ECM degradation, the inflammatory response, cell proliferation, and apoptosis.

EBF1 is a transcription factor with a unique DNA-binding structure that binds to the IPT/TiG-like domain using atypical zinc fingers^42–44^. In this study, EBF1 levels were found to be abnormally high in IDD tissues. Using a ChIP assay, we found that EBF1 directly binds to the promoter region of IKBKB to promote its transcription, thus increasing its expression level. IKKβ, the translated protein of IKBKB, promotes the phosphorylation of IκBα, thereby promoting the activation of the NF-κB signalling pathway. Additionally, we verified the regulatory role of the circEYA3/miR-196a-5p/EBF1 axis in NF-κB signalling, and rescue experiments showed that this axis mediates the activity of p65.

In conclusion, our findings suggest that circEYA3 exerts a competitive binding effect on miR-196a-5p, thereby modulating the expression of EBF1. As a result, this molecular interaction disrupts the normal functioning of the NF-κB signalling pathway, ultimately impacting the advancement of IDD. The circEYA3/miR-196a-5p/EBF1 axis may provide new therapeutic targets for the nonsurgical treatment of IDD.

## Materials and Methods

### Bioinformatics Analysis

A search was performed on Gene Expression Omnibus (GEO; http://www.ncbi.nlm.nih.gov/geo/) to obtain datasets related to IDD. Microarray data from the GEO datasets (GSE70362, GSE67566, and GSE116726) were collected. The acquired microarray data were downloaded and normalized using the ‘affy’ and ‘simpleaffy’ packages of the ‘R’ software (available at www.R-project.org). The processed gene expression matrix can be found in Supplementary Data 2, Supplementary Data 3, and Supplementary Data 4. For data visualization, the R packages ‘pheatmap’ and ‘ggplot2’ were used. A dataset from GO (geneontology.org) was used to calculate the enrichment analysis. The regulatory network was mapped using Cytoscape (cytoscape.org/) based on the base pairings predicted by starBase (starbase.sysu.edu.cn/). ChIP-seq data were collected from the Cistrome Data Browser (cistrome.org/db/#/). In the current study, data were analysed after searching using the keyword ‘EBF1’).

### Clinical samples

The present study obtained approval from the Ethics Committee of the Second Hospital of Dalian Medical University (2023-309). Between October 2019 and March 2022, we procured NP tissues from a cohort of patients who had undergone transforaminal lumbar interbody fusion (n = 33, mean age 66.8 ± 2.1 years). All ethical regulations relevant to human research participants were followed. Additionally, we obtained NP tissues from a group of patients who had undergone percutaneous endoscopic lumbar discectomy without IDD (n = 6, mean age 32.7 ± 2.5 years). Comprehensive patient data, including radiographic assessments, were uploaded in the Supplementary Table 1. Following surgical procedures, tissue samples were cryopreserved in liquid nitrogen prior to experimentation. A subset of tissues from healthy patients was subjected to direct digestion for subsequent cell culture.

### Real-time PCR Analysis

An RNeasy Mini Kit (74106, Qiagen, Valencia, CA) was used to isolate total RNA from NP tissues and cultured NPCs. The QuantiTect Reverse Transcription Kit (205314) from Qiagen was used to synthesize cDNA. A QuantiFast SYBR Green PCR Kit (204056) from Qiagen, Valencia, CA, was utilized for quantitative real-time PCR (qPCR). Relative RNA expression was calculated using the 2-ΔΔCT method with normalization to U6 small nuclear RNA. Each experiment was performed in triplicate.

### NPC culture

NPCs were extracted from normal patient NP tissues. After the NP tissue was cut into small pieces, it was digested for 20 minutes with 0.1% trypsin (15400054, Gibco Company, USA). Afterwards, the sample was digested in Dulbecco’s modified Eagle’s medium (DMEM, 11965092, Gibco Company, USA) at 37 °C for 30 minutes with Type II collagenase (17101015, Gibco Company, USA). Strainers (40 nm) were used to remove undigested tissue. DMEM containing 10% foetal bovine serum (30044333, Gibco Company) was then used to culture NPCs. In the experiment, NPCs from the second and third passages were used.

### Cell transfection

The pcDNA3.1 vector (Invitrogen, USA) was used to clone the cDNA of EBF1, circEYA3 and IKKβ. The miR-196a-5p mimic, the negative control oligonucleotides (miR-NC), the inhibitor for miR-196a-5p, and the negative control oligonucleotide (NC inhibitor) were obtained from RiboBio (Guangzhou, China). RNAi of EBF1, circEYA3 and IKKβ (siEBF1, sicirc-1, sicirc-2, sicirc-3, siIKKβ) and scramble siRNA (siSCR) were purchased from RiboBio (Guangzhou, China). NPCs were transfected with Lipofectamine 3000 (Invitrogen, Carlsbad, CA, USA) in 6-well plates. An Olympus fluorescence microscope (Japan) was used to measure transfection efficiency. qPCR was used to determine the mRNA expression level. For further analysis, NPCs were exposed to IL-1β (10 ng/ml) for 24 hours and then utilized.

### Western blot analysis

A Nuclear and Cytoplasmic Extraction Reagents kit (78835, Thermo Fisher Scientific, USA) was used to extract cytoplasmic and nuclear proteins. A BCA Assay Kit (23223, Thermo Scientific, USA) was used to measure the protein concentration. In this experiment, the protein was electrophoresed on 10% SDS‒PAGE gels and then transferred to PVDF membranes (Millipore, Bedford, MA, USA). The membranes were coincubated overnight at 4 °C with specific primary antibodies (ab214429, ab32535, ab3778, ab41037, ab307674, ab32561, ab15580, ab133462, ab76429, ab124957, ab181602, ab32536, Abcam, Cambridge, UK) (PA5-61136, Invitrogen, USA) (18165-1-AP, Proteintech, China). The membranes were rewarmed for 2 hours and then incubated with rabbit IgG (ab97051, Abcam, Cambridge, UK) at 37 °C for 1 hour. The bands were detected using an ECL Western blot kit (32209, Thermo Fisher Scientific, USA) and ImageQuant™ LAS500 (Cytiva, China). In this study, GAPDH and Lamin B were used as controls. The methodology employed for the utilization of antibodies is documented in Supplementary Table 2.

### Immunofluorescence (IF) staining

After IL-1 stimulation for 24 hours, NPCs were cultured in a 24-well plate and fixed in 4% paraformaldehyde for 20 minutes. After treatment with 0.2% Triton X-100 for 3 minutes, the cells were blocked for 1 hour with 5% BSA. At 4 °C, the cells were incubated overnight with primary antibody. After PBS washes, NPCs were incubated at room temperature for 1 hour with goat anti-rabbit IgG (SA00013-4, SA00013-2, Proteintech, China). As a final step, nuclear staining was performed with DAPI (4’,6-diamidino-2-phenylindole) (c0065, Solarbio, China). Fluorescence was observed using a fluorescence microscope (Olympus, Japan).

### Flow cytometry

The apoptosis rate of treated NPCs was determined using a PE Annexin V Apoptosis Detection Kit I (559763, BD Pharmingen, USA). A fluorescence-activated cell sorting (FACS) flow cytometer (BD Biosciences, USA) was then used to analyse the results. In each experiment, three replicates were used.

### Dual luciferase reporter gene assay

The miRNA target expression vector pmirGLO Dual-Luciferase was purchased from GenePharma (China). For generation of wild type (WT) and mutant (3UT) EBF1 3’-UTR luciferase vectors, the amplified DNA sequences were cloned and inserted into the pmirGLO reporter plasmid. As described above, pmirGLO luciferase reporter vectors were constructed for circEYA3. Transfection of NPCs with plasmid and miR-196a-5p mimic or the control was performed using Lipofectamine 3000 (Invitrogen, Carlsbad, CA, USA). After 48 hours, dual-luciferase reporter gene assays (E1910, Promega, USA) were used to measure luciferase activity. A triplicate experiment was performed, and the mean and SD are presented.

### RNA immunoprecipitation (RIP) assay

The RIP assay was conducted using the Magna RIPTM RNA Binding Protein Immunoprecipitation Kit (17-700, Millipore, USA). MiR-196a-5p and circEYA3 were pulled down together. We collected and resuspended cultured NPCs in RIPA buffer (R0020, Solarbio, China). A negative control was performed by incubating the cell extracts with RIP buffer containing magnetic beads conjugated with mouse IgG (CBL610, Millipore, USA) or anti-Ago2 antibody (07-590, Millipore, USA) conjugated with human anti-Ago2. After three washes, proteinase K was applied to the magnetic beads the following morning. TRIzol reagent was used to isolate total RNA from the extracts. A RT‒qPCR analysis was conducted for circEYA3 and miR-196a-5p to determine their relative enrichment.

### Fluorescence in situ hybridization (FISH) assays

GenePharma, China, provided the FISH kit (F03402). Approximately five minutes of predenaturation at 78 °C was used to add probes specific for miR-196a-5p and circEYA3. Hybridization was then performed overnight at 42 °C. In 20 minutes of darkness, we counterstained the nuclei with DAPI (c0065, Solarbio, China). An Olympus microscope was used to scan and photograph the sample.

### Chromatin immunoprecipitation (ChIP)

An EZ-Magna ChIP kit (17-10086, EMD Millipore, Germany) was used to conduct the ChIP assay. DNA‒protein crosslinks were generated by fixing NPCs in 4% paraformaldehyde and incubating them for 10 minutes with glycine. A sonicator was used to generate chromatin fragments of 400 to 800 bp after the cells were lysed with Nuclear Lysis Buffer and Cell Lysis Buffer. An antibody conjugate for EBF1 (ab108369, Abcam, Cambridge, UK) or IgG was used to immunoprecipitate the lysates. After precipitation of DNA, PCR was conducted to analyse it.

### Rat model of IDD

In all animal experiments, the Animal Ethics Committee at Dalian Medical University ratified all procedures. The 2011 guidelines for the care and use of laboratory animals were followed during the experiment. The Experimental Animal Center at Dalian Medical University provided the rats. The IDD model was established as described previously^45^. Briefly, Following the administration of anesthesia, the rats were positioned in a supine manner, whereupon the abdominal wall was incised, thereby revealing the omentum and abdominal contents. Concurrently, the surrounding abdominal wall was provided with support. Subsequently, the posterior peritoneum was exposed and incised, while ensuring the preservation of the inferior vena cava and the detachment of the psoas major from the spine. This procedure facilitated the complete exposure and puncturing of the lumbar disc. The annulus fibrosus was then punctured with a syringe and left for 1 minute. Finally, the rat abdomen was sutured layer by layer. A total of 50 nM miR-NC, miR-196a-5p agomir and lentivirus expressing EBF1 or circEYA3 were injected into the lumbar discs of the recipients (*n* = 8 per group). After a period of eight weeks following the surgical procedure, the lumbar region of rats was subjected to scanning and subsequently utilized for further experimental investigations.

### Histology staining

The lumbar region of the rats was fixed in a 4% paraformaldehyde solution for paraffin embedding. Tissue decalcification was performed using EDTA decalcification solution for a duration of two months. The sections were subjected to pretreatment steps, including drying, deparaffinization, and rehydration. Subsequently, the tissues were sliced into 4 μm sections. Safranin O and fast green staining (G1371, Solarbio, China) and H&E staining (G1120, Solarbio, China) were employed to assess the degeneration of the nucleus pulposus and the morphology of the lumbar region.

### Statistics and reproducibility

The data were analysed using SPSS 17.0 software. The data were reported as the average plus or minus the standard deviation (SD). A significant difference between two groups was determined using Student’s t test. The variation in the data values is represented by the standard deviation (SD). For determination of the notable distinction among several groups, ANOVA (analysis of variance) was employed in a unidirectional manner. The correlation was identified using the Pearson correlation coefficient. At least three repetitions were used for each experiment. A P value less than 0.05 was used to define statistical significance.

### Reporting summary

Further information on research design is available in the Nature Portfolio Reporting Summary linked to this article.

### Supplementary information

Supplementary Information

Description of Supplementary Materials

Supplementary Data 1

Supplementary Data 2

Supplementary Data 3

Supplementary Data 4

Supplementary Data 5

Reporting Summary

## Data Availability

Datasets used and/or analyzed during the present study can be obtained from the corresponding author upon reasonable request. Images of uncropped blots are provided in Supplementary Fig. 3. Source data for graphs are available in Supplementary Data 1.
