# Supplementary material for: CircEYA3 aggravates intervertebral disc degeneration through the miR-196a-5p/EBF1 axis and NF-κB signaling
Source: Commun Biol. 2024 Mar 30;7:390. doi: 10.1038/s42003-024-06055-2 (PMC10981674; doi:10.1038/s42003-024-06055-2)

Supplementary Table 1. The gene expression matrix of circRNAs

id	logFC	AveExpr	t	P.Value	B
hsa_circ_000178	2.45118972	8.59736988	210.4181487	3.82E-19	34.66045582
hsa_circ_100646	2.04021004	12.46600778	192.5930067	9.04E-19	33.85086049
hsa_circ_000094	-1.7381442	12.953486	-183.5410947	1.44E-18	33.40203875
hsa_circ_100790	-1.6490793	13.24564825	-174.1361965	2.41E-18	32.9053814
hsa_circ_100018	2.57126848	12.80710734	163.8254308	4.37E-18	32.32154013
hsa_circ_001175	2.28857146	10.54738051	154.2115299	7.87E-18	31.7357697
hsa_circ_100319	-2.1792524	11.88554452	-151.7916319	9.18E-18	31.58146084
hsa_circ_000864	1.77543696	9.40277206	146.1514128	1.33E-17	31.21027961
hsa_circ_100227	1.87763952	8.8926687	140.3665111	1.97E-17	30.81179723
hsa_circ_100723	-1.88863978	7.43045043	-133.0191002	3.32E-17	30.27746386
hsa_circ_100604	2.67199762	11.74985539	132.2309533	3.52E-17	30.21814779
hsa_circ_100273	-2.08731664	11.5244404	-131.5268205	3.71E-17	30.16481318
hsa_circ_100236	1.52880272	13.74598334	129.0608938	4.46E-17	29.97544185
hsa_circ_100772	-2.6984196	8.59185784	-128.9869215	4.48E-17	29.9696979
hsa_circ_100684	-2.38677888	10.03441654	-127.4334222	5.04E-17	29.84820057
hsa_circ_100422	1.57102538	8.29801329	127.136405	5.16E-17	29.82478066
hsa_circ_100876	1.91686344	10.43978712	122.1288938	7.63E-17	29.42040238
hsa_circ_000750	-2.3494982	10.72759014	-120.6968944	8.56E-17	29.30133577
hsa_circ_000791	1.116836	13.7170995	117.933427	1.07E-16	29.06704143
hsa_circ_100637	-2.13558324	8.05461754	-117.6851329	1.09E-16	29.04569185
hsa_circ_100329	1.45547144	8.41234392	112.6693855	1.67E-16	28.60339903
hsa_circ_000881	2.02632116	10.88736208	108.4964546	2.41E-16	28.21855807
hsa_circ_100272	-1.54717282	12.08083349	-106.6081275	2.86E-16	28.03904529
hsa_circ_100420	-1.65018748	11.61666386	-102.2025193	4.32E-16	27.60639162
hsa_circ_100085	2.0592209	10.59952591	99.89306154	5.40E-16	27.37143043
hsa_circ_000046	1.51798832	8.17655924	92.23706746	1.17E-15	26.54846585
hsa_circ_100845	1.3355756	11.24255804	90.44652385	1.42E-15	26.34542802
hsa_circ_000911	1.55309534	11.21024543	90.23938803	1.45E-15	26.3216638
hsa_circ_100086	2.23862012	8.21801448	89.1693113	1.63E-15	26.197964
hsa_circ_001067	1.0692433	12.45337159	88.12755986	1.83E-15	26.07601248
hsa_circ_001405	1.49576896	8.3511731	87.83149766	1.89E-15	26.04107468
hsa_circ_100754	-1.17357174	9.09261267	-87.42277378	1.97E-15	25.99263589
hsa_circ_001401	-1.55259906	12.52729707	-85.70545865	2.40E-15	25.78645498
hsa_circ_100035	-1.51137716	7.79545584	-85.45428237	2.46E-15	25.75593235
hsa_circ_001108	1.31896692	11.17098522	84.76850307	2.67E-15	25.67211153
hsa_circ_100034	-1.56709236	8.60465648	-84.32397158	2.81E-15	25.61739387
hsa_circ_100040	1.71433716	6.48578432	83.75766369	3.00E-15	25.54724361
hsa_circ_100051	-1.39212314	9.14940261	-83.03265128	3.26E-15	25.45669976
hsa_circ_100525	1.41262602	8.20413407	82.74972209	3.37E-15	25.42113944
hsa_circ_001046	1.3837194	9.5915602	81.32879787	3.99E-15	25.24058936
hsa_circ_100411	1.31800384	10.47217824	80.32553088	4.50E-15	25.11109793
hsa_circ_100438	0.93936936	11.81524342	78.47537619	5.65E-15	24.86778096
hsa_circ_000963	1.31472582	7.81189843	78.33765785	5.74E-15	24.84942934
hsa_circ_100205	1.04341994	9.46955699	77.83477147	6.11E-15	24.78212899
hsa_circ_100823	-1.22774796	6.1726551	-77.58610534	6.31E-15	24.74868197
hsa_circ_000200	2.48946402	7.60727927	77.14830412	6.66E-15	24.68952151
hsa_circ_100836	-1.42659808	9.12767482	-76.51995228	7.22E-15	24.60399493
hsa_circ_000638	1.38585508	7.68966904	76.07600929	7.64E-15	24.54312477
hsa_circ_000941	-2.26248584	8.46632492	-75.39183335	8.34E-15	24.44858502
hsa_circ_001583	-1.1081408	8.59382118	-75.08566176	8.67E-15	24.40598749
hsa_circ_000662	1.4290372	8.14743368	74.4377413	9.44E-15	24.31524174

hsa_circ_100192	-1.1517077	12.22282515	-74.29585344	9.61E-15	24.29525939
hsa_circ_000671	1.26898056	8.6138709	74.24561041	9.68E-15	24.28817402
hsa_circ_100290	-2.1012706	8.23330774	-73.75221483	1.03E-14	24.21832771
hsa_circ_100508	1.59004516	13.02753552	73.67865767	1.04E-14	24.20787307
hsa_circ_000166	2.16922376	6.96415	72.17072167	1.27E-14	23.99112235
hsa_circ_100640	-1.50197352	8.64699864	-72.07016805	1.29E-14	23.97650157
hsa_circ_100571	2.00809222	8.90012861	72.02133645	1.30E-14	23.96939366
hsa_circ_100427	-2.84656613	7.073818815	-71.81402707	1.34E-14	23.93916189
hsa_circ_001153	1.2897528	9.44752942	71.63806006	1.37E-14	23.91342949
hsa_circ_100045	1.2146054	9.26524766	71.3894239	1.42E-14	23.87695816
hsa_circ_100606	1.58940508	7.2679467	71.18468306	1.46E-14	23.84682642
hsa_circ_001654	2.12861288	9.10859654	70.79894459	1.54E-14	23.78981188
hsa_circ_100476	-2.162316	8.724251	-68.69545266	2.06E-14	23.47312018
hsa_circ_100844	-2.38715834	9.97152331	-67.8825809	2.31E-14	23.34803791
hsa_circ_100593	-0.87421242	10.88284529	-67.59979312	2.41E-14	23.30415965
hsa_circ_001059	-1.46923822	11.27169815	-67.50968828	2.44E-14	23.2901388
hsa_circ_100579	-1.60890002	8.12827113	-67.02744098	2.62E-14	23.21476784
hsa_circ_100202	1.63754254	11.37879823	66.01306643	3.03E-14	23.05438444
hsa_circ_100117	1.66873586	8.17857433	65.53492658	3.26E-14	22.9779009
hsa_circ_001950	1.29487692	6.8385217	65.52567083	3.26E-14	22.97641467
hsa_circ_100412	0.80484324	9.19605542	65.4807503	3.28E-14	22.96919854
hsa_circ_002086	-2.11371662	10.77186431	-65.25282955	3.40E-14	22.93250598
hsa_circ_100213	-1.63603092	10.98319524	-65.06382836	3.49E-14	22.90197863
hsa_circ_000780	1.8686227	8.07333315	65.02219815	3.51E-14	22.89524225
hsa_circ_100257	-1.52222976	10.24247028	-64.48764704	3.81E-14	22.80834687
hsa_circ_001754	-1.5191623	8.42620851	-63.50149737	4.42E-14	22.64607571
hsa_circ_001409	1.72689272	7.54703046	63.10816477	4.70E-14	22.58062781
hsa_circ_100188	-1.29563954	7.11243081	-62.91629382	4.84E-14	22.54854933
hsa_circ_001264	1.13788016	7.33494424	62.66435835	5.03E-14	22.50627566
hsa_circ_100004	-1.17333056	8.82267152	-62.59193554	5.09E-14	22.49409109
hsa_circ_000684	1.38981146	9.18909817	62.16301828	5.44E-14	22.42163069
hsa_circ_100270	-1.4706532	7.93125118	-61.87529073	5.69E-14	22.37273407
hsa_circ_000598	1.30106672	9.91299204	61.49512994	6.04E-14	22.30776991
hsa_circ_001302	-0.75481284	11.33194536	-61.47157468	6.07E-14	22.30373108
hsa_circ_100226	1.5606671	7.48159913	61.20879142	6.32E-14	22.25856579
hsa_circ_100090	1.22659068	11.21526448	60.81160529	6.74E-14	22.18992115
hsa_circ_100882	1.4391607	7.80225149	60.01128241	7.66E-14	22.05019534
hsa_circ_100616	-1.43464798	7.48413809	-59.95327526	7.74E-14	22.03999391
hsa_circ_000711	1.51091378	7.46078127	59.69577405	8.07E-14	21.99458599
hsa_circ_001100	1.2243097	10.37800627	59.06922863	8.94E-14	21.88325703
hsa_circ_001594	-0.66963986	11.07497123	-59.03620387	8.99E-14	21.87735545
hsa_circ_100219	-1.2632643	7.72023849	-58.05215228	1.06E-13	21.69993591
hsa_circ_001216	1.00565562	9.55740661	57.67803792	1.13E-13	21.63167652
hsa_circ_100332	-1.285155	8.52189858	-56.97614971	1.27E-13	21.50238299
hsa_circ_100685	1.04794418	11.35396649	56.81673221	1.30E-13	21.47279024
hsa_circ_000956	-1.06885542	13.05191179	-56.03682615	1.49E-13	21.32678305
hsa_circ_100701	-1.98306782	5.71951711	-55.28857098	1.70E-13	21.18473897
hsa_circ_100893	-1.0679431	8.33520623	-55.27461726	1.70E-13	21.1820715
hsa_circ_100477	-1.18776898	8.95398659	-54.38097928	2.00E-13	21.00979364
hsa_circ_100283	-1.17878776	8.43381654	-54.27670228	2.03E-13	20.9895032
hsa_circ_100108	1.12849542	10.86209037	54.18493723	2.07E-13	20.97161448
hsa_circ_100851	0.85000344	9.16463826	54.10139053	2.10E-13	20.95530101
hsa_circ_100223	1.10901524	13.03183188	53.90749636	2.17E-13	20.91734185

hsa_circ_100883	1.92324206	7.38746705	53.7404466	2.24E-13	20.88452647
hsa_circ_100698	-2.41655144	6.2012452	-53.30967221	2.42E-13	20.79942349
hsa_circ_100528	-0.7105696	7.90069864	-53.03479294	2.55E-13	20.74475243
hsa_circ_000872	-0.8051739	11.26231555	-52.97584426	2.57E-13	20.73299053
hsa_circ_100033	-1.31053668	9.61702286	-52.84736307	2.63E-13	20.70730879
hsa_circ_100084	-0.98354126	9.02917599	-52.10134921	3.02E-13	20.55692454
hsa_circ_100620	-1.06034624	10.2168556	-51.98545356	3.09E-13	20.53336567
hsa_circ_100533	-1.11518278	11.11426209	-51.69488197	3.26E-13	20.47406394
hsa_circ_100735	-1.04504212	7.04980538	-51.47715445	3.40E-13	20.42940645
hsa_circ_100770	-1.7176014	10.17852398	-50.52064484	4.08E-13	20.23092136
hsa_circ_001012	-0.7409334	9.10897098	-49.92955737	4.57E-13	20.10635044
hsa_circ_100806	-0.71952262	9.60782749	-49.85531363	4.64E-13	20.0905981
hsa_circ_100395	0.86120276	9.77763214	49.42176957	5.05E-13	19.99813552
hsa_circ_100498	-0.7112877	9.45742225	-49.29229239	5.18E-13	19.97036235
hsa_circ_100374	2.11558444	10.1379365	49.24763334	5.23E-13	19.96076574
hsa_circ_001653	2.22151978	11.63910189	48.96166554	5.53E-13	19.89910558
hsa_circ_100079	-2.28266352	10.4807411	-48.62993584	5.91E-13	19.82711991
hsa_circ_100180	-1.09638402	7.08563063	-48.2786728	6.34E-13	19.75035183
hsa_circ_100464	-1.0795639	7.14144717	-48.03284585	6.66E-13	19.69628986
hsa_circ_001040	1.61164237	6.869397575	47.67944253	7.15E-13	19.61807762
hsa_circ_100802	1.62299234	7.08581567	47.17480129	7.93E-13	19.50537295
hsa_circ_100269	-0.87123226	8.89183747	-47.13427364	8.00E-13	19.49626887
hsa_circ_100478	-0.93538688	8.24800646	-46.78231314	8.60E-13	19.41687086
hsa_circ_100879	-0.8122987	8.36382225	-46.61010998	8.91E-13	19.37780388
hsa_circ_001363	1.12083162	6.37814885	46.60304922	8.93E-13	19.37619893
hsa_circ_000950	-1.50967756	7.92760874	-45.92747396	1.03E-12	19.2214905
hsa_circ_001072	-1.28896542	6.82326711	-45.79587506	1.06E-12	19.19108696
hsa_circ_100350	1.10611924	6.83032784	45.62916588	1.10E-12	19.15244506
hsa_circ_001729	-1.68015529	5.747324995	-45.58781315	1.11E-12	19.14283778
hsa_circ_000585	-0.67000574	7.65530993	-45.36962447	1.16E-12	19.09200111
hsa_circ_000481	-0.84648114	11.42880931	-44.92206734	1.27E-12	18.98694724
hsa_circ_001389	1.49677202	7.77453993	44.91403163	1.28E-12	18.98505141
hsa_circ_100433	-1.89530152	13.66628122	-44.8550516	1.29E-12	18.97112608
hsa_circ_100445	-0.79149278	10.97211551	-44.76814816	1.32E-12	18.95057432
hsa_circ_000943	-2.56591412	10.16033372	-44.64273037	1.35E-12	18.92084334
hsa_circ_001225	1.11023206	8.24813391	44.4630894	1.41E-12	18.87811159
hsa_circ_000676	-1.27696896	13.60724394	-43.7426003	1.65E-12	18.70496205
hsa_circ_100892	-1.00594502	7.96127781	-43.6781013	1.67E-12	18.68932178
hsa_circ_100453	1.09279654	8.62362099	43.35857055	1.80E-12	18.61149493
hsa_circ_100351	-0.80997772	9.54775768	-43.23202418	1.85E-12	18.58051294
hsa_circ_100688	1.02484966	6.35771087	42.7581118	2.06E-12	18.46367062
hsa_circ_100817	-1.2147678	6.97935174	-42.62096045	2.12E-12	18.42961325
hsa_circ_001296	-0.9259799	7.30513705	-42.5357296	2.16E-12	18.40839323
hsa_circ_100668	1.59573632	7.19926162	42.5300019	2.17E-12	18.40696567
hsa_circ_100175	0.97280588	7.69686286	42.074401	2.41E-12	18.29278929
hsa_circ_100230	1.46204079	6.785059205	41.97427209	2.46E-12	18.26752998
hsa_circ_100530	-0.64283744	6.71612182	-41.90561381	2.50E-12	18.25017471
hsa_circ_001396	-1.23204966	9.18653341	-41.89593369	2.51E-12	18.2477255
hsa_circ_100037	-1.43572602	6.80883501	-41.68074339	2.64E-12	18.19313201
hsa_circ_100808	-1.07095042	10.23786301	-41.40383938	2.81E-12	18.12246415
hsa_circ_000645	-0.72546028	9.81833956	-41.36853015	2.83E-12	18.11341891
hsa_circ_001724	1.05159374	6.89077349	41.08110141	3.03E-12	18.03949828
hsa_circ_100449	-0.79624654	8.07985223	-40.97256885	3.11E-12	18.01145098

hsa_circ_100759	0.91637092	9.31109046	40.68855916	3.33E-12	17.93770238
hsa_circ_100322	-0.6841298	6.45340102	-40.66742202	3.34E-12	17.93219309
hsa_circ_100629	-0.77896462	6.33530601	-40.10203453	3.83E-12	17.78375298
hsa_circ_002106	-1.03718066	7.34481851	-39.92251928	4.00E-12	17.73618318
hsa_circ_001304	-1.30897735	6.131369165	-39.47451575	4.46E-12	17.61652599
hsa_circ_100696	0.61790562	13.79506561	39.36914997	4.58E-12	17.58818643
hsa_circ_002143	0.84933926	6.32632009	39.36342915	4.59E-12	17.58664557
hsa_circ_100361	-0.96254472	7.60775968	-39.08417137	4.91E-12	17.51115566
hsa_circ_001240	1.60280134	6.82447159	38.83549904	5.23E-12	17.44347815
hsa_circ_100896	-1.27638136	9.93404124	-38.21075326	6.12E-12	17.27151983
hsa_circ_100750	0.98513102	11.39696051	37.8583011	6.69E-12	17.17326514
hsa_circ_100266	-1.0576901	6.27543595	-37.74365135	6.89E-12	17.14110667
hsa_circ_000868	-0.88781754	7.61136199	-37.69528914	6.98E-12	17.12751213
hsa_circ_100229	1.15445158	7.48051373	37.48722645	7.36E-12	17.06882678
hsa_circ_100861	0.96008134	10.80916689	37.37052938	7.59E-12	17.03576916
hsa_circ_100745	-1.27463816	5.9804166	-36.60349799	9.27E-12	16.8158891
hsa_circ_001038	-0.95127656	11.77434638	-36.17401383	1.04E-11	16.69075627
hsa_circ_100373	1.03950884	10.8357246	35.92557533	1.11E-11	16.61769457
hsa_circ_001486	0.96611814	5.84277937	35.31704274	1.31E-11	16.43658769
hsa_circ_100655	-0.96976206	9.05027879	-35.29846297	1.32E-11	16.43100938
hsa_circ_100714	0.99051948	8.53406486	35.18778023	1.36E-11	16.39771776
hsa_circ_100531	-0.9136769	6.20223051	-34.84672893	1.49E-11	16.29447594
hsa_circ_100421	1.50703614	7.74471713	34.84010077	1.49E-11	16.29245955
hsa_circ_100499	0.91380882	6.76935527	34.78997407	1.52E-11	16.27719783
hsa_circ_100832	0.91138036	7.71574328	34.34278786	1.72E-11	16.14007072
hsa_circ_100439	-0.74705484	7.10046274	-33.95379188	1.92E-11	16.01933648
hsa_circ_100072	1.10010334	7.13679171	33.73913529	2.04E-11	15.9521231
hsa_circ_100389	-0.80034552	7.35457118	-33.15007029	2.42E-11	15.76547096
hsa_circ_100850	0.60739534	9.64973967	32.65117764	2.80E-11	15.60479981
hsa_circ_100849	0.76020616	9.16600224	32.23711483	3.17E-11	15.46959004
hsa_circ_000441	-1.01660484	6.36911402	-31.7807893	3.63E-11	15.31857302
hsa_circ_000629	0.90864142	14.61068189	31.76093181	3.66E-11	15.31195272
hsa_circ_100179	0.82226674	5.67462505	31.69855071	3.73E-11	15.29112878
hsa_circ_100641	0.81211784	8.8032765	31.67538751	3.75E-11	15.28338616
hsa_circ_100751	1.4488197	12.03660011	31.48048738	3.98E-11	15.21801537
hsa_circ_100633	-1.81514382	13.62869961	-31.40694093	4.07E-11	15.19324333
hsa_circ_001769	-1.1074031	6.34114505	-31.30095661	4.21E-11	15.15744443
hsa_circ_100881	0.82006118	8.36321503	31.0627871	4.53E-11	15.07655742
hsa_circ_100147	0.78611994	6.36882247	30.86413615	4.82E-11	15.0086215
hsa_circ_100069	0.72292428	9.5912844	30.78403409	4.94E-11	14.98110538
hsa_circ_000996	0.9079796	8.9016227	30.67200872	5.12E-11	14.94250435
hsa_circ_100815	1.06539044	6.17557174	30.50006258	5.40E-11	14.88298448
hsa_circ_100365	-0.73870582	6.51041951	-30.48475467	5.43E-11	14.87766954
hsa_circ_001350	1.61125082	8.62815033	30.36537118	5.64E-11	14.83612865
hsa_circ_100017	-0.6813063	6.87419521	-29.84473949	6.66E-11	14.65306458
hsa_circ_001800	0.80421008	6.22699784	29.76117216	6.85E-11	14.62338767
hsa_circ_100407	-1.40093756	7.62629172	-29.36632332	7.79E-11	14.48204656
hsa_circ_100827	1.2179772	6.7771812	29.23298446	8.14E-11	14.43389303
hsa_circ_100647	1.10998246	5.43012173	28.82456004	9.32E-11	14.28503886
hsa_circ_100053	0.6387845	12.18317417	28.75490426	9.54E-11	14.25944512
hsa_circ_100413	0.9589644	5.86815702	28.65557902	9.86E-11	14.22284418
hsa_circ_000926	1.08593422	6.95932569	28.57769283	1.01E-10	14.19405606
hsa_circ_100891	0.84088412	7.17401674	28.54980462	1.02E-10	14.18372931

hsa_circ_100443	-1.01819448	6.5540979	-28.35673492	1.09E-10	14.1119642
hsa_circ_002172	0.9018417	5.85458213	28.08308524	1.20E-10	14.00941997
hsa_circ_100008	0.81826162	6.53118621	27.75185872	1.34E-10	13.88397891
hsa_circ_100693	0.9472488	6.61445094	27.34539106	1.55E-10	13.72801903
hsa_circ_000162	0.65161268	5.97917774	27.32836601	1.56E-10	13.72143708
hsa_circ_100870	-0.80497202	5.22673171	-27.19735163	1.63E-10	13.67065144
hsa_circ_100748	1.128857	7.14759948	27.13247864	1.67E-10	13.64541561
hsa_circ_001830	-0.70968266	7.51574129	-27.05192947	1.72E-10	13.61399926
hsa_circ_100302	0.80074496	6.42507744	26.78126215	1.89E-10	13.50775608
hsa_circ_100840	-0.60286564	8.1987215	-26.0941828	2.43E-10	13.23326733
hsa_circ_001915	-0.85255006	8.17878085	-25.97308774	2.54E-10	13.18415802
hsa_circ_100600	1.03440484	6.24393942	25.95151052	2.56E-10	13.17538404
hsa_circ_001838	0.74915307	5.481824815	25.18806056	3.41E-10	12.86026526
hsa_circ_100821	-0.87033672	12.11807786	-24.53762763	4.39E-10	12.58435737
hsa_circ_000031	1.02720825	5.913059335	24.44283212	4.55E-10	12.54355245
hsa_circ_100403	-0.6961789	8.66224683	-24.42720819	4.58E-10	12.53681232
hsa_circ_100112	0.68063574	7.95760401	24.41105903	4.61E-10	12.52984119
hsa_circ_100146	-0.67087006	7.47907429	-24.06074013	5.29E-10	12.37750428
hsa_circ_001109	1.1582763	9.40980431	23.98255835	5.46E-10	12.34321257
hsa_circ_100695	-0.8700856	5.86082794	-23.81160524	5.85E-10	12.26785001
hsa_circ_100300	0.84753485	5.854050535	23.72825807	6.05E-10	12.2309167
hsa_circ_100583	-0.81343056	6.2641466	-23.66219346	6.21E-10	12.20155215
hsa_circ_100005	-0.68682784	6.85489034	-23.29643477	7.22E-10	12.03752549
hsa_circ_002144	1.73060314	6.80733501	23.24557023	7.37E-10	12.01451744
hsa_circ_100875	-0.86865484	7.63686874	-23.19995316	7.51E-10	11.99384148
hsa_circ_001671	-0.67956872	8.0347035	-23.06751691	7.93E-10	11.93359074
hsa_circ_100075	1.16660692	7.26169276	23.05146009	7.98E-10	11.92626306
hsa_circ_100106	0.61193903	5.368265565	22.92426939	8.42E-10	11.86804314
hsa_circ_100303	-0.8887566	5.5673047	-22.72562384	9.15E-10	11.77648716
hsa_circ_100068	0.67155182	6.71185239	22.64359851	9.47E-10	11.73845551
hsa_circ_100465	-1.1002799	6.21843007	-22.31247623	1.09E-09	11.58356185
hsa_circ_001288	0.60564615	5.817666745	22.00268741	1.25E-09	11.43662402
hsa_circ_100488	-1.01855696	6.78787638	-21.98347478	1.26E-09	11.42744553
hsa_circ_100156	-0.67854831	5.198036555	-21.56184456	1.51E-09	11.22404761
hsa_circ_100331	-1.0498519	6.82781333	-21.53261428	1.53E-09	11.20980499
hsa_circ_000993	1.48485164	8.61267374	21.31842056	1.69E-09	11.10486646
hsa_circ_100409	-0.6386725	6.43627925	-21.14690108	1.82E-09	11.02010115
hsa_circ_100036	-1.16736316	6.01934508	-21.12519601	1.84E-09	11.00932738
hsa_circ_100719	0.75871234	6.90280067	21.08428467	1.87E-09	10.98899121
hsa_circ_002178	-0.91284898	8.79076161	-21.0522065	1.90E-09	10.97301928
hsa_circ_100660	0.64624225	5.841950995	20.44274811	2.52E-09	10.66504312
hsa_circ_001369	0.66946624	5.48605694	20.41641284	2.55E-09	10.65153758
hsa_circ_100298	-0.62373632	5.95373924	-20.3444523	2.64E-09	10.61454874
hsa_circ_100308	0.69777434	6.20698741	20.13907353	2.90E-09	10.50828781
hsa_circ_100470	1.22561234	6.01993435	19.95400459	3.17E-09	10.41164329
hsa_circ_000815	0.99278802	6.15204395	19.27571847	4.41E-09	10.04996631
hsa_circ_100013	-0.7416012	5.52431494	-19.14018209	4.71E-09	9.976243478
hsa_circ_100385	-0.89317763	5.981162035	-19.01666296	5.01E-09	9.908623402
hsa_circ_000987	-0.75775262	11.45966949	-18.88152426	5.36E-09	9.834162272
hsa_circ_100651	0.69972992	6.20615516	18.60574886	6.17E-09	9.68062747
hsa_circ_100868	1.40284112	6.07126454	18.08337528	8.08E-09	9.38379591
hsa_circ_100852	0.92795272	5.81689484	17.98084852	8.53E-09	9.324585674
hsa_circ_000620	-0.73378086	6.48712499	-17.59065659	1.05E-08	9.096293882

hsa_circ_100357	-0.70751866	5.75957177	-17.25647437	1.26E-08	8.896948558
hsa_circ_100627	-0.7724521	5.58501173	-17.15961623	1.33E-08	8.838492747
hsa_circ_100510	-0.60363982	7.69321571	-16.9118349	1.52E-08	8.687531188
hsa_circ_000446	-0.86544474	6.34332729	-16.72850456	1.69E-08	8.574497091
hsa_circ_100907	-0.7392281	5.49979827	-16.71016267	1.71E-08	8.563124521
hsa_circ_100659	0.7302851	6.11220341	16.55297122	1.87E-08	8.465179465
hsa_circ_000942	0.72109792	6.19230612	16.39201099	2.05E-08	8.363982051
hsa_circ_001689	-0.83435386	6.11976023	-16.32740384	2.12E-08	8.323101995
hsa_circ_100547	-2.03177404	6.53483538	-16.28354192	2.18E-08	8.295262455
hsa_circ_100252	-0.94765136	6.3965294	-16.18084303	2.31E-08	8.229804422
hsa_circ_000526	0.81141526	5.53914417	16.12371503	2.39E-08	8.19322473
hsa_circ_100895	0.60409118	5.43765079	15.43053555	3.61E-08	7.739523289
hsa_circ_100830	-0.7019625	5.54994595	-15.27385035	3.98E-08	7.634360181
hsa_circ_100833	0.63787429	5.298796105	14.95646835	4.84E-08	7.418260494
hsa_circ_100552	-0.66703968	5.77454062	-14.51191486	6.42E-08	7.108384808
hsa_circ_100177	1.39822884	10.44189104	14.41549576	6.83E-08	7.040031485
hsa_circ_100612	0.62254288	7.2996452	13.60909536	1.17E-07	6.45149992
hsa_circ_000082	0.97885796	5.87527974	13.13276112	1.63E-07	6.088888695
hsa_circ_100339	0.65794238	5.82622459	13.10120695	1.66E-07	6.064453522
hsa_circ_100115	0.60675094	6.71560895	12.98587698	1.80E-07	5.974693664
hsa_circ_100906	1.03217136	5.97236976	12.13826201	3.36E-07	5.292387251
hsa_circ_100471	0.62319041	5.489965345	11.98588979	3.78E-07	5.165299956
hsa_circ_100136	0.61107388	5.54607096	11.75838414	4.50E-07	4.972894578
hsa_circ_100296	0.68134984	5.38400472	11.4739779	5.63E-07	4.727764055
hsa_circ_100104	0.72846394	5.97685301	11.02908117	8.07E-07	4.333592046
hsa_circ_100566	-0.62153796	6.07614204	-9.658743415	2.66E-06	3.028862271
hsa_circ_100107	1.19228874	6.23301025	8.981766606	5.06E-06	2.327474387
hsa_circ_100526	-0.72797432	5.50609692	-8.950999623	5.21E-06	2.294613505
hsa_circ_100009	-0.61199438	10.08602687	-8.950954113	5.21E-06	2.294564833
hsa_circ_100029	-0.62888442	6.35047651	-7.574803108	2.20E-05	0.72797872
hsa_circ_001459	1.26707614	6.18350239	7.491329699	2.41E-05	0.626423657
hsa_circ_100129	-0.657674	5.98545808	-7.394461898	2.69E-05	0.507568648
hsa_circ_001241	1.04059416	7.64722924	6.689999128	6.16E-05	-0.390491612
hsa_circ_100657	0.61995338	6.13705287	2.632600795	0.02556943	-6.750696087
hsa_circ_000617	0.7498601	5.16097187	2.452510344	0.034703814	-7.054092426

Supplementary Table 2. The gene expression matrix of miRNAs

id	logFC	AveExpr	t	P.Value	B
hsa-miR-32-5p	-1.1363987	1.75372875	-17783.60474	6.87E-18	30.85181771
hsa-miR-3160-3p	2.959943578	3.801899889	4776.571531	1.83E-15	27.04995892
hsa-miR-3155b	2.9598338	3.801954778	4707.370925	1.95E-15	26.98663059
hsa-miR-3922-5p	2.9598338	3.801954778	4707.370925	1.95E-15	26.98663059
hsa-miR-4296	2.9598338	3.801954778	4707.370925	1.95E-15	26.98663059
hsa-miR-3183	3.640370856	4.142113528	2005.235256	7.33E-14	22.90333899
hur_2	-26.97642237	26.56937332	-599.5138063	1.24E-11	16.65301886
hsa-miR-1301-5p	4.3605878	4.502222	573.3838889	1.50E-11	16.41983489
hsa-miR-1915-5p	4.3605878	4.502222	573.3838889	1.50E-11	16.41983489
hsa-miR-1262	4.360478022	4.502276889	573.3132332	1.50E-11	16.41918997
hsa-miR-200a-5p	4.360478022	4.502276889	573.3132332	1.50E-11	16.41918997
hsa-miR-200b-5p	3.628946972	4.193321981	435.0038201	4.84E-11	14.97336256
hsa-miR-2682-3p	5.227993194	4.935924697	383.5861574	8.27E-11	14.31407534
hsa-miR-1261	3.651956789	4.148016272	383.0566215	8.32E-11	14.30683366
hsa-miR-186-3p	3.651956789	4.148016272	383.0566215	8.32E-11	14.30683366
hsa-miR-18b-5p	3.651956789	4.148016272	383.0566215	8.32E-11	14.30683366
hsa-miR-203a-3p	3.651956789	4.148016272	383.0566215	8.32E-11	14.30683366
hsa-miR-376b-3p	3.651956789	4.148016272	383.0566215	8.32E-11	14.30683366
hsa-miR-129-5p	2.142238942	3.393047571	339.3855229	1.39E-10	13.67219882
hsa-miR-3939	2.142238942	3.393047571	339.3855229	1.39E-10	13.67219882
hsa-miR-1250-5p	2.142129164	3.39310246	339.3198305	1.39E-10	13.67118375
hsa-miR-378h	2.142129164	3.39310246	339.3198305	1.39E-10	13.67118375
hsa-miR-4715-5p	5.257492417	4.950784086	338.7656547	1.40E-10	13.66261285
hsa-miR-492	5.257492417	4.950784086	338.7656547	1.40E-10	13.66261285
hsa-miR-6084	3.664963028	4.154409614	323.7403657	1.70E-10	13.42471457
hsa-miR-1204	4.382537344	4.513196772	319.9829759	1.79E-10	13.3634937
hsa-miR-4255	4.399175444	4.521515822	296.0592899	2.49E-10	12.95594698
hsa-miR-1258	2.971385733	3.807730744	280.5289182	3.13E-10	12.67332413
hsa-miR-1298-3p	2.971385733	3.807730744	280.5289182	3.13E-10	12.67332413
hsa-miR-29b-2-5p	2.971385733	3.807730744	280.5289182	3.13E-10	12.67332413
hsa-miR-30d-3p	2.971385733	3.807730744	280.5289182	3.13E-10	12.67332413
hsa-miR-3605-3p	2.971385733	3.807730744	280.5289182	3.13E-10	12.67332413
hsa-miR-369-3p	2.971385733	3.807730744	280.5289182	3.13E-10	12.67332413
hsa-miR-3912-5p	2.971385733	3.807730744	280.5289182	3.13E-10	12.67332413
hsa-miR-3927-5p	4.400500972	4.522288364	271.9681731	3.56E-10	12.51075879
hsa-miR-15b-3p	6.255974744	5.488742889	266.0153969	3.92E-10	12.39466885
hsa-miR-4482-3p	5.324833311	4.984454533	264.8550799	3.99E-10	12.3717379
hsa-miR-187-3p	8.349468633	6.496662417	235.4092218	6.58E-10	11.7534524
hsa-miR-141-3p	8.349358856	6.496717306	235.4050653	6.58E-10	11.75335977
hsa-miR-4315	4.385442289	4.5283824	219.7337957	8.82E-10	11.39192376
hsa-miR-302c-5p	5.283757194	4.963806697	214.9484389	9.69E-10	11.27639918
hsa-miR-100-3p	4.377116122	4.510595939	209.9111438	1.07E-09	11.15197825
hsa-miR-143-5p	4.377116122	4.510595939	209.9111438	1.07E-09	11.15197825
hsa-miR-181b-3p	4.377116122	4.510595939	209.9111438	1.07E-09	11.15197825
hsa-miR-20a-3p	4.377116122	4.510595939	209.9111438	1.07E-09	11.15197825
hsa-miR-25-5p	4.377116122	4.510595939	209.9111438	1.07E-09	11.15197825
hsa-miR-301a-3p	4.377116122	4.510595939	209.9111438	1.07E-09	11.15197825
hsa-miR-132-5p	4.322042439	4.521494681	208.4751556	1.10E-09	11.11596183
hsa-miR-3185	2.987969272	3.815912736	207.861666	1.12E-09	11.10049899
hsa-miR-514a-5p	2.987969272	3.815912736	207.861666	1.12E-09	11.10049899
hsa-miR-19b-1-5p	5.20579835	4.947022119	205.3600663	1.18E-09	11.03697048

hsa-miR-1244	2.153888914	3.398982335	204.2038761	1.20E-09	11.00734676
hsa-miR-3064-5p	2.153888914	3.398982335	204.2038761	1.20E-09	11.00734676
hsa-miR-3200-3p	2.153888914	3.398982335	204.2038761	1.20E-09	11.00734676
hsa-miR-382-3p	2.153888914	3.398982335	204.2038761	1.20E-09	11.00734676
hsa-miR-383-5p	2.153888914	3.398982335	204.2038761	1.20E-09	11.00734676
hsa-miR-4316	2.153888914	3.398982335	204.2038761	1.20E-09	11.00734676
hsa-miR-127-5p	7.598674919	6.135108493	198.9069072	1.35E-09	10.86944722
hsa-miR-3130-5p	3.639424383	4.155483225	192.1692618	1.56E-09	10.68863348
hsa-miR-3678-3p	3.639424383	4.155483225	192.1692618	1.56E-09	10.68863348
hsa-miR-3197	3.639534161	4.155428336	191.7990316	1.57E-09	10.67851484
hsa-miR-411-3p	4.338392956	4.513319422	191.2313987	1.59E-09	10.66296305
hsa-miR-376a-5p	-1.082985689	1.780545033	-187.148834	1.75E-09	10.5497309
hsa-miR-410-5p	-1.076912806	1.783471697	-186.1309188	1.79E-09	10.52111358
hsa-miR-6894-3p	-5.889545011	5.169650111	-183.5535933	1.90E-09	10.44794958
hsa-miR-3687	4.363247444	4.539479822	183.0366539	1.92E-09	10.43315125
hsa-miR-1273h-3p	5.250190517	4.947133136	179.8780795	2.07E-09	10.34181296
hsa-miR-130b-5p	5.250190517	4.947133136	179.8780795	2.07E-09	10.34181296
hsa-miR-15a-3p	5.250190517	4.947133136	179.8780795	2.07E-09	10.34181296
hsa-miR-1911-5p	5.250190517	4.947133136	179.8780795	2.07E-09	10.34181296
hsa-miR-27a-5p	5.250190517	4.947133136	179.8780795	2.07E-09	10.34181296
hsa-miR-339-5p	5.250190517	4.947133136	179.8780795	2.07E-09	10.34181296
hsa-miR-379-3p	5.250190517	4.947133136	179.8780795	2.07E-09	10.34181296
hsa-miR-4695-3p	4.395189528	4.519632642	179.5257722	2.08E-09	10.33152579
hsa-miR-135b-3p	4.36468275	4.540197475	176.829558	2.22E-09	10.25212269
hsa-miR-300	2.94077255	3.839511097	170.9218158	2.57E-09	10.07382089
hsa-miR-3200-5p	5.247829194	4.981770697	170.1087586	2.62E-09	10.04880061
hsa-miR-3620-3p	5.247829194	4.981770697	170.1087586	2.62E-09	10.04880061
hsa-miR-1291	4.399344889	4.557528544	169.1027846	2.68E-09	10.0176776
hsa-miR-1296-5p	2.941297383	3.839248681	169.0466463	2.69E-09	10.01593533
hsa-miR-3130-3p	2.970381039	3.820961553	168.7038266	2.71E-09	10.00528325
hsa-miR-597-3p	6.447491044	5.593610733	168.0416795	2.76E-09	9.984647605
hsa-miR-3606-3p	5.236567139	4.953944825	163.2108444	3.12E-09	9.831588141
hsa-miR-3074-3p	2.158668164	3.401262182	162.7701758	3.16E-09	9.817401233
hsa-miR-3934-3p	2.158558386	3.401317071	162.7566498	3.16E-09	9.816965167
hsa-miR-5010-3p	4.514224189	4.628172933	160.0601854	3.39E-09	9.729302027
hsa-miR-4461	2.96532245	3.827236147	159.9433398	3.40E-09	9.725470016
hsa-miR-4303	2.972949811	3.822245939	158.5988559	3.53E-09	9.681174426
hsa-miR-3978	2.973059589	3.82219105	158.2879497	3.56E-09	9.670877801
hsa-miR-7855-5p	-3.134494036	3.750708882	-158.1745102	3.57E-09	9.667115859
hsa-miR-2114-3p	3.629871722	4.159058806	155.0990828	3.88E-09	9.564085358
hsa-miR-1301-3p	3.618475539	4.167093869	152.7972684	4.13E-09	9.485625998
hsa-miR-3663-5p	3.631372	4.1735421	152.1383312	4.21E-09	9.462947837
hsa-miR-4300	3.631372	4.1735421	152.1383312	4.21E-09	9.462947837
hsa-miR-296-3p	2.974126339	3.822834203	152.0193879	4.22E-09	9.45884379
hsa-miR-3619-5p	2.961397878	3.816469972	146.9861306	4.87E-09	9.282165738
hsa-miR-1288-5p	5.254148194	4.949002197	146.9656826	4.87E-09	9.281435699
hsa-miR-1247-5p	2.974887461	3.845299831	145.9633797	5.02E-09	9.245526138
hsa-miR-223-5p	7.594413278	6.119244517	143.4497984	5.40E-09	9.154375994
hsa-miR-138-2-3p	4.336063383	4.532557614	141.8642306	5.66E-09	9.096053263
hsa-miR-3164	5.276455294	4.960155747	141.2679666	5.76E-09	9.07395178
hsa-miR-516a-3p	5.276455294	4.960155747	141.2679666	5.76E-09	9.07395178
hsa-miR-4521	3.632208694	4.160227292	140.3035735	5.94E-09	9.038006786
hsa-miR-182-5p	2.937748733	3.812997311	137.3874687	6.49E-09	8.927794966

hsa-miR-3680-3p	2.161354183	3.449433469	136.9277327	6.58E-09	8.910206436
hsa-miR-3923	2.148213497	3.406489515	128.6641548	8.57E-09	8.583571923
hsa-miR-3622b-3p	2.140265536	3.405794024	125.9477997	9.39E-09	8.471605069
hsa-miR-6731-3p	4.658037483	4.690582036	124.3565823	9.91E-09	8.404889004
hsa-miR-1273h-5p	5.227101278	4.969458361	121.3762067	1.10E-08	8.277600101
hsa-miR-1199-5p	2.19107445	3.431308258	121.3380956	1.10E-08	8.275952256
hsa-miR-377-5p	2.950477194	3.819361542	118.748552	1.21E-08	8.162756439
hsa-miR-3714	2.187940906	3.429741486	118.6565284	1.21E-08	8.158688578
hsa-miR-3177-3p	3.654371194	4.181335264	115.8352285	1.34E-08	8.032419372
hsa-miR-193b-5p	7.565595739	11.50211767	115.5190998	1.36E-08	8.0180797
hsa-miR-95-3p	-5.722889022	5.183371767	-113.9990898	1.43E-08	7.948579349
hsa-miR-939-3p	3.328729906	4.022221053	111.3404443	1.59E-08	7.824759248
hsa-miR-1295b-3p	2.987442544	3.81575915	110.238371	1.65E-08	7.772564052
hsa-miR-4434	5.509303256	5.112507728	99.16504876	2.59E-08	7.217132222
hsa-miR-4260	2.991433239	3.853572719	98.17537912	2.71E-08	7.164507039
hsa-miR-335-3p	2.103570636	3.412381724	95.98477194	2.98E-08	7.046111973
hsa-miR-4725-3p	4.4369455	4.586854072	93.10422109	3.39E-08	6.886250078
hsa-miR-136-5p	2.122354025	3.419419251	92.20847445	3.53E-08	6.835530169
hsa-miR-4746-3p	-5.243067867	5.410965456	-87.01979622	4.52E-08	6.531683997
hsa-miR-4466	4.008294983	12.90572712	85.54936191	4.85E-08	6.442279428
hsa-miR-205-5p	2.136473319	3.412359604	84.9353984	5.01E-08	6.404494353
hsa-miR-365b-5p	2.136473319	3.412359604	84.9353984	5.01E-08	6.404494353
hsa-miR-3685	2.178115011	3.410985606	83.18903648	5.47E-08	6.295507519
hsa-miR-4506	2.972415589	3.884656611	81.79333823	5.87E-08	6.206748931
hsa-miR-513b-3p	4.408545189	4.53993385	81.75542588	5.89E-08	6.204316866
hsa-miR-4642	3.780330139	4.262770464	81.67038875	5.91E-08	6.198857661
hsa-miR-7845-5p	-5.3893196	5.604780694	-80.27201254	6.36E-08	6.108261245
hsa-miR-6840-3p	-4.218612317	4.636336014	-78.84397283	6.87E-08	6.0141014
hsa-miR-5703	3.878082589	12.16873804	78.32700077	7.06E-08	5.979593598
hsa-miR-3613-3p	2.137248261	3.436088453	77.70147538	7.31E-08	5.937534459
hsa-miR-421	2.114944142	3.406694971	77.68845057	7.31E-08	5.936655106
hsa-miR-6776-3p	5.810980994	5.378776086	76.4947735	7.81E-08	5.855434048
hsa-miR-6885-3p	2.546367728	3.631039964	75.09991695	8.44E-08	5.758904973
hsa-miR-2110	2.164381856	3.417852183	73.67374192	9.16E-08	5.658339479
hsa-miR-4667-3p	4.569829828	4.642771014	73.32557098	9.34E-08	5.633493312
hsa-miR-7641	-13.53809602	10.02512963	-73.15248407	9.44E-08	5.621097615
hsa-let-7f-5p	-12.84678967	10.32601466	-71.42487682	1.04E-07	5.495744282
hsa-miR-3940-3p	2.146856483	3.431284342	68.17526334	1.27E-07	5.251530389
hsa-miR-431-5p	2.146856483	3.431284342	68.17526334	1.27E-07	5.251530389
hsa-miR-4425	2.146856483	3.431284342	68.17526334	1.27E-07	5.251530389
hsa-miR-8082	3.112232989	3.878044594	66.6817553	1.40E-07	5.135366189
hsa-miR-1285-3p	2.171598731	3.42992231	66.22270313	1.44E-07	5.099139107
hsa-miR-6785-3p	5.775158761	5.349725936	65.22619805	1.54E-07	5.019627401
hsa-miR-22-3p	-13.39614562	10.54159297	-61.22307428	2.01E-07	4.68750804
hsa-miR-6728-3p	5.673185811	5.18071585	60.45424639	2.12E-07	4.621247239
hsa-miR-509-5p	4.425380733	4.570546467	60.45151934	2.12E-07	4.621010717
hsa-miR-937-3p	-5.0882498	4.845615006	-60.44201301	2.12E-07	4.620186133
hsa-miR-154-3p	2.922310839	3.832268192	59.78484723	2.22E-07	4.562867286
hsa-miR-98-3p	-5.517885628	5.339726075	-59.61990831	2.25E-07	4.548382414
hsa-miR-6810-3p	5.439003528	5.077357864	58.13947696	2.50E-07	4.416552434
hsa-miR-520b	7.073403739	5.936640581	58.03590488	2.52E-07	4.407204553
hsa-miR-4697-3p	5.53444835	5.125080275	57.44872276	2.63E-07	4.353891881
hsa-miR-4651	5.103394883	10.30894746	57.09722976	2.70E-07	4.3217177

hsa-miR-486-5p	-11.59599494	7.653207156	-56.79744707	2.76E-07	4.294120372
hsa-miR-509-3-5p	3.711737961	4.213725081	56.41231848	2.84E-07	4.258452295
hsa-miR-7154-5p	-5.083209978	4.766305844	-55.27631789	3.10E-07	4.151811053
hsa-miR-4787-5p	4.761749761	10.00617949	54.97028484	3.18E-07	4.122708591
hsa-miR-373-3p	5.3337113	5.002516906	54.61297803	3.26E-07	4.088525063
hsa-miR-6857-5p	-3.479000933	3.549635956	-54.49497602	3.29E-07	4.077186809
hsa-miR-3187-3p	5.3740517	5.04488195	52.59080266	3.83E-07	3.890759296
hsa-miR-6850-3p	4.675961522	4.695836861	52.24627101	3.94E-07	3.856310166
hsa-miR-21-5p	21.83345567	29.14085667	51.88969739	4.06E-07	3.820417666
hsa-let-7e-5p	-8.750119272	8.177032747	-50.48745	4.56E-07	3.676842625
hsa-miR-3944-5p	2.172499833	3.477139767	50.38380463	4.60E-07	3.666073208
hsa-miR-4648	5.49261865	5.298117458	49.5608903	4.93E-07	3.579775185
hsa-miR-338-3p	8.101539289	6.433048744	49.51739839	4.95E-07	3.575174595
hsa-miR-1236-5p	2.511245344	8.8871882	48.61334045	5.35E-07	3.478620109
hsa-let-7a-5p	-13.68176841	12.01849557	-47.74708447	5.77E-07	3.384409418
hsa-miR-7157-3p	2.113182906	3.392362486	47.72525665	5.78E-07	3.38201357
hsa-miR-5585-3p	-5.107324572	5.215514575	-46.81688036	6.28E-07	3.281328704
hsa-miR-4636	-2.819733228	5.337275792	-46.14024661	6.68E-07	3.205057412
hsa-miR-4472	1.167733239	2.941722719	45.80609209	6.88E-07	3.166979013
hsa-miR-431-3p	5.407113061	5.104904536	44.5106159	7.78E-07	3.016691374
hsa-miR-942-3p	-4.704901886	4.693128215	-44.50326445	7.78E-07	3.015826164
hsa-miR-8069	-21.19788627	15.93854798	-44.03488122	8.14E-07	2.960405587
hsa-miR-4695-5p	5.479270778	9.539629794	43.62675567	8.47E-07	2.911634244
hsa-let-7i-5p	-10.44359602	9.611664944	-43.35585694	8.69E-07	2.879010154
hsa-miR-557	-1.803635917	5.803136281	-43.28324037	8.75E-07	2.870230514
hsa-miR-6791-5p	2.563423483	9.077080392	43.13743822	8.88E-07	2.852558072
hsa-miR-8063	4.120156533	8.283197072	42.89884781	9.09E-07	2.823510251
hsa-miR-4733-5p	3.731435133	4.2014886	42.84977705	9.14E-07	2.817516066
hsa-miR-3196	-2.329912994	7.154178592	-42.56138844	9.40E-07	2.782149597
hsa-miR-6875-3p	3.200409022	3.922242389	41.54418401	1.04E-06	2.655472047
hsa-miR-211-3p	5.102910644	9.368358267	41.47591309	1.05E-06	2.646859718
hsa-miR-6511b-3p	5.656653839	5.186183019	40.41978495	1.17E-06	2.511802442
hsa-miR-4793-5p	6.652628589	11.18783351	40.20428595	1.20E-06	2.48381356
hsa-miR-455-5p	17.33461372	16.88492123	39.84554108	1.24E-06	2.436887298
hsa-miR-146a-5p	15.41758108	15.90812926	39.44702577	1.30E-06	2.384263524
hsa-miR-4443	4.879239	15.45993389	39.40139069	1.30E-06	2.378203721
hsa-miR-6799-3p	5.675486772	5.195599486	39.14210939	1.34E-06	2.343641149
hsa-miR-192-5p	9.469256328	8.894915414	38.84621913	1.38E-06	2.303919281
hsa-miR-4646-5p	4.254309894	9.861698097	38.70830394	1.41E-06	2.285301904
hsa-miR-6831-3p	4.643090928	4.657206719	38.52476211	1.43E-06	2.260422757
hsa-miR-6759-3p	5.683478011	5.199595106	37.88342775	1.54E-06	2.172554727
hsa-miR-767-3p	-4.612865006	4.708893647	-37.81448876	1.55E-06	2.163021638
hsa-miR-378a-5p	-2.874885031	2.827410882	-36.91644018	1.72E-06	2.037233609
hsa-miR-1305	6.715402656	10.30753223	36.35842712	1.83E-06	1.957532533
hsa-miR-518b	5.636353833	5.15394795	36.33174734	1.84E-06	1.953691472
hsa-miR-4674	5.511575617	5.137330558	35.99295382	1.91E-06	1.904670483
hsa-miR-765	-5.744570656	5.400993033	-35.87284251	1.94E-06	1.887181096
hsa-miR-874-5p	3.191369194	3.917722475	35.55833889	2.01E-06	1.841109232
hsa-miR-151b	-5.508910372	5.404745831	-35.18345033	2.11E-06	1.785659786
hsa-miR-6855-5p	3.899556956	4.271816356	35.17874468	2.11E-06	1.784960053
hsa-miR-7846-3p	7.2511047	6.473328756	34.69788211	2.24E-06	1.712961074
hsa-miR-9500	-7.010851456	6.274582478	-34.4508	2.30E-06	1.675579464
hsa-miR-6874-5p	2.53952285	3.591799303	34.30118903	2.35E-06	1.652814872

hsa-miR-6867-5p	5.715970994	6.538666453	34.12174882	2.40E-06	1.625381127
hsa-miR-582-5p	-3.329699428	3.620742814	-33.6700189	2.54E-06	1.555679169
hsa-miR-10b-5p	-8.213593006	7.477264414	-33.50531981	2.59E-06	1.530034945
hsa-miR-222-3p	-4.5975339	7.877437578	-33.32710594	2.65E-06	1.502145112
hsa-miR-6803-5p	-11.49950901	8.935376056	-33.3167598	2.65E-06	1.500521436
hsa-miR-3195	2.821831033	12.69686382	33.26525709	2.67E-06	1.492431381
hsa-miR-1185-1-3p	3.908054639	9.745836681	33.11349247	2.72E-06	1.468519688
hsa-miR-8052	-4.813386311	4.690555511	-32.99513104	2.77E-06	1.449795326
hsa-miR-6810-5p	4.609745611	4.626910683	32.76229254	2.85E-06	1.412765788
hsa-miR-3198	-9.679587511	7.913530733	-32.70287898	2.87E-06	1.403275122
hsa-miR-6127	-11.29518238	8.812550789	-32.6978197	2.87E-06	1.402466167
hsa-miR-6861-3p	3.677205372	4.337413781	32.65765994	2.89E-06	1.396040404
hsa-miR-4533	3.060353189	3.86583785	32.50420494	2.95E-06	1.371414415
hsa-miR-7975	4.306209939	9.225330536	32.42064397	2.98E-06	1.357956261
hsa-miR-6089	-15.89154959	12.01059665	-32.31071291	3.02E-06	1.340198491
hsa-miR-6807-5p	-5.524870372	5.194777392	-31.86809387	3.20E-06	1.268088202
hsa-miR-6789-5p	-5.759959417	5.330195775	-31.86347671	3.21E-06	1.267330763
miRNABrightCorner30	-20.39726732	14.0633889	-31.8519077	3.21E-06	1.265432406
hsa-miR-3174	2.912887972	3.914259458	31.75691271	3.25E-06	1.249818784
hsa-miR-6891-5p	6.429423733	9.014119961	31.65042704	3.30E-06	1.232261373
hsa-miR-1273g-5p	4.487739417	4.580267714	31.63449504	3.31E-06	1.229629462
hsa-miR-184	5.583761322	5.414568528	31.24026044	3.49E-06	1.164081166
hsa-miR-518e-5p	5.769247772	5.242479986	31.07423713	3.57E-06	1.136231186
hsa-miR-129-1-3p	4.439147722	4.714547083	30.96494292	3.62E-06	1.117816742
hsa-miR-3156-5p	3.382335811	9.751526772	30.90959674	3.65E-06	1.108467149
hsa-miR-3911	-2.254236317	6.311346514	-30.89044786	3.66E-06	1.105228477
hsa-miR-92a-1-5p	2.4292683	3.53872475	30.80758954	3.70E-06	1.091191584
hsa-miR-1295a	3.6462381	4.259934733	30.63679088	3.79E-06	1.062138423
hsa-miR-373-5p	4.617189394	4.685657303	30.25317777	3.99E-06	0.996295699
hsa-miR-125b-5p	-14.49668563	11.12559471	-30.19405207	4.03E-06	0.986073945
hsa-miR-4634	5.360401156	12.43260029	30.11819585	4.07E-06	0.972930708
hsa-miR-4538	-3.191891711	3.827730739	-29.92929829	4.18E-06	0.940058306
hsa-miR-610	8.580824772	6.894421919	29.86081592	4.22E-06	0.928090058
hsa-miR-7-2-3p	4.626021883	4.635048819	29.61368475	4.37E-06	0.884673202
hsa-miR-718	-9.296765294	8.443457086	-28.98192482	4.79E-06	0.772031308
hsa-miR-6880-5p	-5.443667067	5.199008117	-28.91962167	4.83E-06	0.76079134
hsa-miR-1185-2-3p	3.069868739	8.409250719	28.59137458	5.07E-06	0.701174384
hsa-miR-4666b	5.44454425	5.251191164	28.53883977	5.11E-06	0.691570056
hsa-miR-4505	-10.25107851	9.438697814	-28.53342827	5.12E-06	0.69057974
hsa-miR-6846-3p	3.853027978	4.248551867	28.04360625	5.51E-06	0.600162738
hsa-miR-563	7.868320228	6.503152308	27.42201622	6.05E-06	0.483146395
hsa-miR-874-3p	-9.001030183	8.427734408	-27.38065452	6.09E-06	0.475267099
hsa-miR-6881-3p	-3.485005739	3.961840931	-27.35995321	6.11E-06	0.471319134
hsa-miR-99a-3p	2.4083897	3.548317794	27.19634044	6.27E-06	0.44001208
hsa-miR-615-3p	5.040709206	5.085330692	27.18095896	6.28E-06	0.437059287
hsa-miR-7111-3p	-5.748054761	5.171605569	-27.15094555	6.31E-06	0.431292835
hsa-miR-4532	4.041395439	10.1992596	27.04693145	6.42E-06	0.411259819
hsa-miR-150-3p	5.69367555	10.142553	26.92213755	6.54E-06	0.387123898
hsa-miR-625-3p	5.207761967	5.150862239	26.90104251	6.56E-06	0.383033063
hsa-miR-4728-5p	-5.658001844	5.432632628	-26.89515099	6.57E-06	0.381889989
hsa-miR-3682-3p	3.947404867	5.866677861	26.89489201	6.57E-06	0.381839736
hsa-miR-6775-5p	-7.768993189	7.11968525	-26.89478111	6.57E-06	0.381818215
hsa-miR-1267	4.405240878	4.738667267	26.7915768	6.68E-06	0.361753984

hsa-miR-6871-5p	-3.594573489	4.125336544	-26.74946443	6.72E-06	0.353544852
hsa-miR-5001-5p	-5.328256772	4.926947919	-26.74127209	6.73E-06	0.351946403
hsa-miR-6778-5p	-5.356787689	5.178488961	-26.63089173	6.85E-06	0.330362216
hsa-miR-8075	-4.64373455	4.770940336	-26.61356787	6.87E-06	0.32696662
hsa-miR-129-2-3p	3.748094817	4.366209214	26.51149915	6.98E-06	0.306916002
hsa-miR-6892-5p	2.9497235	3.958159317	26.48237257	7.01E-06	0.301180318
hsa-miR-1249-5p	8.074942039	11.24318485	26.48225919	7.01E-06	0.301157981
hsa-miR-24-3p	-8.852555428	13.53755906	-26.25981632	7.27E-06	0.257147105
hsa-miR-6790-5p	5.251900111	5.085623394	26.24834275	7.28E-06	0.254867052
hsa-miR-4653-3p	5.882898194	9.815743897	26.13767611	7.41E-06	0.232824482
hsa-let-7g-5p	-9.970715511	8.253857806	-25.7800535	7.86E-06	0.160957759
hsa-miR-1273e	-3.029982783	3.611009553	-25.75025668	7.90E-06	0.154925526
hsa-miR-3137	5.775062272	10.06142454	25.71769413	7.94E-06	0.148325499
hsa-miR-181a-5p	-8.086162172	12.11566614	-25.58337406	8.12E-06	0.121012989
hur_6	6.103431478	9.216849939	25.47668514	8.26E-06	0.09921791
hsa-miR-574-3p	-8.47308405	7.323574358	-25.37754823	8.40E-06	0.078884706
hsa-miR-627-5p	5.709221794	5.224163942	25.31788672	8.48E-06	0.066610178
hsa-miR-320b	-6.736763533	11.28833822	-25.21445782	8.63E-06	0.045263326
hsa-miR-99b-5p	3.634678189	4.302983422	25.10483603	8.79E-06	0.022543788
hsa-miR-6819-3p	3.1941102	6.127957972	24.98862554	8.97E-06	-0.00164841
hsa-miR-130a-3p	-7.083430956	6.090075594	-24.91651309	9.08E-06	-0.016716339
hsa-miR-4441	5.663590044	7.711872539	24.72353723	9.38E-06	-0.057251287
hsa-miR-5696	5.474550383	5.211134853	24.57194794	9.63E-06	-0.089312335
hsa-miR-6132	4.928871239	8.109420592	24.39429875	9.93E-06	-0.127134174
hsa-miR-1268a	2.267965222	11.01767912	24.39329054	9.93E-06	-0.127349598
hsa-miR-3127-5p	5.4033506	8.853673878	24.22165477	1.02E-05	-0.164151459
hsa-miR-6716-5p	4.326394039	4.536990692	24.14950242	1.04E-05	-0.17969905
hsa-miR-3163	6.677264994	8.122047142	24.04280503	1.06E-05	-0.202774609
hsa-miR-1249-3p	5.640854439	10.85045301	23.93032238	1.08E-05	-0.227210875
hsa-miR-6872-5p	-5.844866594	5.294280814	-23.88534902	1.08E-05	-0.237012788
hsa-miR-6895-5p	-4.843333378	4.830122428	-23.71636432	1.12E-05	-0.274006108
hsa-miR-664a-5p	-4.83378695	4.871420508	-23.65930622	1.13E-05	-0.286555654
hsa-miR-5581-5p	3.073898072	8.500927503	23.56658067	1.15E-05	-0.307013737
hsa-miR-8077	-4.854382369	4.646705996	-23.50002517	1.16E-05	-0.32174684
hsa-miR-6831-5p	-2.302288428	7.374562597	-23.49787137	1.16E-05	-0.322224302
hsa-miR-1268b	12.04697941	14.89588841	23.48528078	1.17E-05	-0.325016301
hsa-miR-8059	2.410413539	3.540868025	23.4743199	1.17E-05	-0.327448107
mr_1	-13.29588234	11.36929683	-23.43940019	1.17E-05	-0.335202934
hsa-miR-195-5p	-7.895415794	11.00497768	-23.41207189	1.18E-05	-0.341279831
hsa-miR-155-5p	-7.055342872	5.988672225	-22.97509403	1.28E-05	-0.439409264
hsa-miR-7155-5p	-3.859861706	4.200196753	-22.9728987	1.28E-05	-0.43990688
hsa-miR-199a-3p	-8.798220544	12.69286117	-22.87220006	1.30E-05	-0.462782655
hsa-miR-6893-5p	6.628698172	9.059638969	22.85146106	1.31E-05	-0.46750625
hsa-miR-6869-5p	-14.22105093	11.43780398	-22.80003254	1.32E-05	-0.479238034
hsa-miR-3940-5p	-1.895968878	6.629683856	-22.78682047	1.32E-05	-0.482256152
hsa-miR-199a-5p	-7.464223567	11.11799591	-22.75516709	1.33E-05	-0.489493944
hsa-miR-371a-3p	5.135575606	5.087619997	22.69746172	1.35E-05	-0.502714252
hsa-miR-4750-3p	5.395408183	5.231232631	22.68319911	1.35E-05	-0.505986913
hsa-miR-7108-5p	5.050102411	6.876918078	22.34311122	1.44E-05	-0.584627978
hsa-miR-7515	-8.5164112	6.986881194	-22.33596511	1.44E-05	-0.586293024
hsa-miR-4514	6.255378911	6.988667422	22.30268414	1.45E-05	-0.5940544
hsa-miR-4269	5.104754544	6.912560306	22.130029	1.50E-05	-0.634502524
hsa-miR-125a-3p	5.388786833	12.41208445	21.88838657	1.57E-05	-0.691636244

hsa-miR-6776-5p	5.475592411	5.112604044	21.87900174	1.57E-05	-0.693867667
hsa-miR-6126	3.3626295	8.483941728	21.86679848	1.57E-05	-0.696770633
hsa-miR-29c-3p	21.99464321	23.91166331	21.78174327	1.60E-05	-0.717048254
hsa-miR-6781-5p	-3.576088489	3.737767283	-21.72580186	1.62E-05	-0.730427433
hsa-miR-6877-5p	-7.307548456	6.778786856	-21.59135049	1.66E-05	-0.762722262
hsa-miR-133a-3p	-2.639515594	3.258485642	-21.51219707	1.69E-05	-0.781827162
hsa-miR-6801-3p	5.967272872	5.116502519	21.46617687	1.70E-05	-0.792966603
hsa-miR-6125	-9.339678456	8.4526323	-21.46486609	1.70E-05	-0.793284229
hsa-miR-4685-5p	6.790178433	7.47921735	21.42056597	1.72E-05	-0.804030126
hsa-miR-6722-3p	4.78692075	8.324587508	21.35423538	1.74E-05	-0.820160823
hsa-miR-26b-5p	-6.376560789	8.87696095	-21.01045346	1.86E-05	-0.904559819
hsa-miR-937-5p	3.46368515	9.802696736	20.88359081	1.91E-05	-0.936047447
hsa-miR-6073	-3.249752372	3.537108892	-20.87644037	1.91E-05	-0.937827785
hsa-miR-3170	2.256597811	3.464069939	20.82241537	1.94E-05	-0.951298448
hsa-miR-623	-2.461594661	3.063660919	-20.6910211	1.99E-05	-0.984204031
hsa-miR-5195-3p	6.534320478	10.26594906	20.63299015	2.01E-05	-0.99880222
hsa-miR-6873-5p	-3.401917817	3.930439986	-20.54220506	2.05E-05	-1.021720924
hsa-miR-6886-5p	-6.444763472	6.574810975	-20.52681815	2.06E-05	-1.025615188
hsa-miR-6131	-5.054806439	4.591455319	-20.51248091	2.06E-05	-1.029246365
hsa-miR-708-5p	-4.858929633	4.654342422	-20.50863355	2.06E-05	-1.030221207
hsa-miR-6723-5p	-4.478883494	4.666076619	-20.44773677	2.09E-05	-1.045675085
hsa-miR-320d	-6.863046828	11.59077273	-20.42991753	2.10E-05	-1.050205635
hsa-miR-3189-3p	2.282803883	3.499258042	20.401328	2.11E-05	-1.057482629
hsa-miR-744-3p	2.402366344	3.52322105	20.35284389	2.13E-05	-1.069846335
hsa-miR-642b-3p	3.487137428	9.701777036	20.34541063	2.13E-05	-1.071744406
hsa-miR-34a-5p	17.52203323	18.26910602	20.25191999	2.18E-05	-1.09567527
hsa-miR-6756-5p	3.871342894	9.474314625	20.23260596	2.19E-05	-1.100632582
hsa-miR-101-3p	-4.824183106	4.838413553	-20.13574218	2.23E-05	-1.125564622
hsa-miR-4707-3p	3.63321425	4.299556281	20.09916535	2.25E-05	-1.135009796
hsa-miR-7157-5p	2.4548069	3.658992111	20.09492548	2.25E-05	-1.136105739
hsa-miR-125b-2-3p	-4.860159778	4.524744706	-20.04884625	2.27E-05	-1.148031154
hsa-miR-23b-3p	-13.60579603	10.66059976	-19.88016202	2.35E-05	-1.191917139
hsa-miR-4713-3p	2.450688044	8.976376606	19.77885717	2.40E-05	-1.218448774
hsa-miR-6858-5p	-5.447667489	5.708288367	-19.68568334	2.45E-05	-1.242968559
hsa-miR-583	5.351997311	5.449252167	19.67801681	2.46E-05	-1.244991149
hsa-miR-8087	-5.494385972	5.143330103	-19.66472901	2.46E-05	-1.248498575
hsa-miR-454-3p	-1.793108858	3.298821746	-19.62484163	2.48E-05	-1.259041123
hsa-miR-1273f	5.854265011	9.287551494	19.6106653	2.49E-05	-1.262793085
hsa-miR-6829-5p	-2.131398428	6.284644997	-19.55582905	2.52E-05	-1.277331289
hsa-miR-762	3.821965522	9.380320939	19.54666076	2.53E-05	-1.279765879
hsa-miR-100-5p	-12.73368183	10.12352969	-19.53635225	2.53E-05	-1.28250458
hsa-miR-671-5p	2.747671272	9.477722714	19.53204607	2.53E-05	-1.283649041
hsa-miR-4257	-4.4113491	4.653352611	-19.43270218	2.59E-05	-1.310120519
hsa-miR-6827-3p	5.558048572	4.826661008	19.32927227	2.65E-05	-1.337821733
hsa-miR-6815-5p	-3.2494525	3.564893539	-19.3199209	2.65E-05	-1.340333413
hsa-miR-6851-5p	-3.726064939	3.737577886	-19.29088324	2.67E-05	-1.348140199
hsa-miR-518a-5p	7.931686222	6.763518017	19.24405622	2.70E-05	-1.360753892
hsa-miR-1273d	4.376267267	4.695019739	19.17400623	2.74E-05	-1.379679215
hsa-miR-4690-5p	-4.151011294	4.369761892	-19.04776203	2.82E-05	-1.413957693
hsa-miR-4667-5p	3.845583761	9.540002292	19.0315926	2.83E-05	-1.418364126
hsa-miR-6792-5p	3.903691372	5.852080875	19.02528878	2.83E-05	-1.42008301
hsa-miR-6869-3p	-4.194677239	4.324211269	-19.00638121	2.84E-05	-1.425241939
hsa-miR-371b-5p	4.3795039	9.02362785	18.86984039	2.93E-05	-1.462646579

hsa-miR-6507-3p	6.379618594	4.883993931	18.80883414	2.97E-05	-1.479444419
hsa-miR-6851-3p	4.536057611	4.895077906	18.70173513	3.04E-05	-1.509062791
hsa-miR-196a-5p	-6.248512556	8.194120361	-18.68376988	3.06E-05	-1.514047314
hsa-miR-6820-5p	3.321553678	7.76572475	18.57118283	3.13E-05	-1.5453919
hsa-miR-4656	-2.849422333	6.023894139	-18.44976957	3.22E-05	-1.579402186
hsa-miR-1915-3p	5.85795345	13.52102483	18.41801066	3.25E-05	-1.588334514
hsa-miR-139-5p	8.5453454	7.106524389	18.33664148	3.31E-05	-1.611288665
hsa-miR-1471	6.821587878	10.64598853	18.32548377	3.31E-05	-1.614443984
hsa-miR-6890-5p	-4.670922367	4.724749372	-18.3048885	3.33E-05	-1.620273096
hur_1	3.009973267	11.62794577	18.28815984	3.34E-05	-1.625012545
hsa-miR-766-5p	-4.775670617	4.617240664	-18.26403772	3.36E-05	-1.631854104
hsa-miR-208a-5p	3.916176656	7.357602806	18.2595133	3.37E-05	-1.633138308
hsa-miR-6876-5p	-5.014693117	4.742363336	-18.12939744	3.47E-05	-1.670203552
hsa-miR-892b	-4.7761889	4.748944572	-18.11114586	3.48E-05	-1.675423482
hsa-miR-3614-5p	5.171426761	6.830340764	17.8699993	3.68E-05	-1.744876436
hsa-miR-5100	-7.867248144	7.629962872	-17.83310304	3.72E-05	-1.755583418
hsa-miR-4311	2.731176644	3.87542095	17.79006153	3.75E-05	-1.768100988
hsa-miR-532-5p	-5.295597783	5.053690031	-17.70614437	3.83E-05	-1.792591248
hsa-miR-6779-5p	2.421543978	9.027278522	17.58792329	3.94E-05	-1.827285066
hsa-miR-4462	5.349908128	8.123946486	17.56497921	3.96E-05	-1.834044649
hsa-miR-6839-5p	5.048421911	4.348244933	17.50801516	4.02E-05	-1.850864101
hsa-miR-4658	3.39698155	4.249843486	17.46703363	4.06E-05	-1.86299744
hsa-miR-4516	3.339489711	16.74675276	17.40006369	4.12E-05	-1.882884838
hsa-miR-6800-5p	2.047948267	13.49026983	17.39245869	4.13E-05	-1.885147924
hsa-miR-99b-3p	-5.583314267	5.556867383	-17.28962466	4.23E-05	-1.915843951
hsa-miR-4470	3.868393456	5.970161017	17.2834482	4.24E-05	-1.917693274
hsa-miR-28-3p	4.284790761	4.760008019	17.21624815	4.31E-05	-1.937855574
hsa-miR-6763-5p	2.328271889	11.00181498	17.14657469	4.38E-05	-1.958840889
hsa-miR-6889-5p	-6.188757117	5.606768431	-17.12940502	4.40E-05	-1.964025036
hsa-miR-1908-3p	5.496666894	6.711351414	17.08874036	4.45E-05	-1.976323334
hsa-miR-4465	-5.728071867	5.281137739	-16.93620373	4.62E-05	-2.022709967
hsa-miR-6799-5p	-4.44439025	4.632719892	-16.90653661	4.65E-05	-2.031778888
hsa-miR-6793-5p	5.329046667	5.489461239	16.89029009	4.67E-05	-2.036751821
hsa-miR-4298	-2.858647433	7.632513561	-16.76208681	4.82E-05	-2.076157508
hsa-miR-5194	-4.479316344	4.613711294	-16.73179502	4.86E-05	-2.085510967
hsa-miR-652-3p	5.160146978	7.750272811	16.69130289	4.91E-05	-2.098039759
hsa-miR-1224-5p	2.3455628	9.022875883	16.65589596	4.95E-05	-2.109019311
hsa-miR-4669	-3.563877439	8.835167708	-16.57442919	5.05E-05	-2.13436804
hsa-miR-409-3p	-3.334154089	5.599872261	-16.56693897	5.06E-05	-2.136704714
hsa-miR-212-3p	4.252929717	4.735729064	16.5374721	5.10E-05	-2.145907249
hsa-miR-6887-3p	-3.337919278	3.884991206	-16.51173523	5.13E-05	-2.153957915
hsa-miR-4419a	3.555982789	9.175007911	16.44630855	5.22E-05	-2.174478713
hsa-miR-4688	-3.548992556	3.994527967	-16.44245966	5.23E-05	-2.175688364
hsa-miR-575	2.191979083	13.1779488	16.44031762	5.23E-05	-2.176361697
hsa-miR-10a-5p	-5.642922356	5.169860133	-16.40586162	5.28E-05	-2.18720433
hsa-miR-7851-3p	-8.305966428	7.337619458	-16.34737418	5.35E-05	-2.205659801
hsa-miR-6796-5p	-3.885008561	4.372512153	-16.33679901	5.37E-05	-2.209003578
hsa-miR-6841-3p	4.865746083	4.519116453	16.31581901	5.40E-05	-2.215643488
hsa-miR-518c-5p	3.287564422	4.347831489	16.3043384	5.41E-05	-2.21928046
hsa-miR-5088-5p	-4.281716783	4.621381019	-16.29788824	5.42E-05	-2.22132491
hsa-miR-7113-5p	-3.5353949	3.987212261	-16.21598114	5.54E-05	-2.247354659
hsa-miR-4776-5p	6.875297583	8.760381814	16.16476393	5.61E-05	-2.263696052
hsa-miR-4496	6.958580428	10.18690784	16.1247034	5.67E-05	-2.276512759

hsa-miR-4734	4.253188344	4.698392578	16.11049403	5.69E-05	-2.281066217
hsa-miR-887-3p	-6.364294394	6.304269997	-16.06192046	5.77E-05	-2.29666125
hsa-miR-4486	5.120692628	8.245134819	16.04837163	5.79E-05	-2.30101938
hsa-miR-875-3p	2.400557767	3.535940139	16.0152727	5.84E-05	-2.311680997
hsa-miR-25-3p	-8.261238533	7.958467683	-15.98912535	5.88E-05	-2.3201185
hsa-miR-6090	3.226735111	16.87812444	15.90946037	6.00E-05	-2.345908154
hsa-miR-6757-5p	3.413976567	8.991545794	15.87048561	6.06E-05	-2.358570842
hsa-miR-512-3p	-2.593642956	3.017245022	-15.86356443	6.07E-05	-2.360822638
hsa-miR-6081	4.858621339	5.145789631	15.84872411	6.10E-05	-2.365654119
hsa-miR-877-5p	-3.979460428	4.188279358	-15.84852084	6.10E-05	-2.365720327
hsa-miR-519e-5p	5.736725278	8.831761022	15.71501884	6.32E-05	-2.409382052
hsa-miR-7151-3p	2.802394033	4.015326478	15.65784917	6.41E-05	-2.428189052
hsa-miR-378a-3p	-4.964257378	8.1848621	-15.64475423	6.44E-05	-2.432506209
hsa-miR-6717-5p	-4.604257606	4.975796619	-15.62983324	6.46E-05	-2.437429631
hsa-miR-27b-3p	-8.0156531	11.34901386	-15.54492436	6.61E-05	-2.465533407
hsa-let-7c-5p	-13.21630578	11.44584194	-15.53092309	6.64E-05	-2.470181883
hsa-miR-4280	2.114209583	3.549469308	15.50596351	6.68E-05	-2.478478601
hsa-miR-550b-2-5p	4.274030389	5.015780039	15.3940015	6.89E-05	-2.515854769
hsa-miR-7108-3p	2.345527433	3.690961428	15.35629241	6.96E-05	-2.528502225
hsa-miR-4741	4.013833161	12.80648225	15.29856832	7.07E-05	-2.547920769
hsa-miR-6845-5p	-4.512890156	4.854927217	-15.15011635	7.36E-05	-2.598186746
hsa-miR-548ai	5.165357978	4.250703767	15.08415836	7.50E-05	-2.620672673
hsa-miR-4767	-3.310252156	3.873274244	-15.05232324	7.57E-05	-2.631559589
hsa-miR-532-3p	-3.840000894	3.767426775	-15.05158883	7.57E-05	-2.631811004
hsa-miR-185-5p	-5.277619667	5.311766517	-14.92428263	7.84E-05	-2.675571797
hsa-miR-6765-5p	-1.469800956	3.9190872	-14.84940313	8.01E-05	-2.701479349
hsa-miR-7856-5p	-3.688818439	4.069286825	-14.83693736	8.04E-05	-2.705804584
hsa-miR-6775-3p	5.878315069	5.161876576	14.81623055	8.08E-05	-2.712996933
hsa-miR-6785-5p	-10.82280041	8.431269975	-14.7885217	8.15E-05	-2.722636529
hsa-miR-6772-5p	-5.567124078	5.174962206	-14.73465966	8.27E-05	-2.741424336
hsa-miR-3147	3.478552878	5.092889839	14.69934616	8.36E-05	-2.753778043
hsa-miR-126-3p	-6.930241006	6.466485819	-14.69037361	8.38E-05	-2.756921451
hsa-miR-10b-3p	-4.764221289	4.969423906	-14.67875381	8.41E-05	-2.76099503
hsa-miR-4271	-2.3160515	7.121186956	-14.55053727	8.72E-05	-2.806151134
hsa-miR-425-5p	-3.864192222	6.16596225	-14.54511735	8.73E-05	-2.808068367
hsa-miR-4530	-5.306786922	13.40560767	-14.54308347	8.74E-05	-2.808788004
hsa-miR-6864-3p	-4.906899794	4.820922275	-14.41765055	9.06E-05	-2.8533572
hsa-miR-186-5p	-3.5183031	6.295220328	-14.38096348	9.16E-05	-2.866463332
hsa-miR-6820-3p	4.7792641	4.495968944	14.30619685	9.36E-05	-2.893272681
hsa-miR-4327	-1.70960425	6.339104825	-14.26306674	9.48E-05	-2.908799202
hsa-miR-370-3p	-2.885163261	6.524147647	-14.26222226	9.48E-05	-2.909103657
hsa-miR-214-3p	-7.264414194	10.97574959	-14.22862475	9.57E-05	-2.921230437
hsa-miR-130b-3p	-5.001102139	5.449459042	-14.15277527	9.79E-05	-2.948709032
hsa-miR-6809-5p	3.364820517	9.873659986	14.14355769	9.82E-05	-2.952057973
hsa-miR-6821-5p	1.7265935	13.48190872	14.12280479	9.88E-05	-2.9596056
hsa-miR-4647	-4.963075078	4.961370644	-14.11237581	9.91E-05	-2.963402534
hsa-miR-134-5p	4.379412022	10.75980983	14.0999367	9.94E-05	-2.967934822
hsa-miR-3917	4.27059655	8.054323147	14.08649864	9.98E-05	-2.97283539
hsa-miR-1233-5p	1.9545666	5.677409517	14.08388039	9.99E-05	-2.973790731
hsa-miR-6830-5p	5.680194378	5.018056811	14.06890715	0.000100352	-2.9792574
hsa-miR-6069	3.092414983	6.616925653	14.04463652	0.000101078	-2.988130361
hsa-miR-320e	-6.966401144	11.23008534	-14.02586825	0.000101644	-2.995001819
hsa-miR-6754-5p	5.112570453	5.291411179	13.99113212	0.000102701	-3.007742714

hsa-miR-8089	-5.847969072	5.993178281	-13.96438165	0.000103525	-3.017575199
hsa-miR-513a-5p	-5.859622622	5.538052022	-13.92762366	0.00010467	-3.031115536
hsa-miR-664a-3p	-3.980411772	3.943859781	-13.92661655	0.000104701	-3.031486999
hsa-miR-8072	5.126619567	4.348553983	13.90214614	0.000105472	-3.040520631
hsa-miR-514b-5p	-1.877431072	5.766478525	-13.84015781	0.000107457	-3.063472856
hsa-miR-6797-3p	3.008750594	6.612861092	13.75456476	0.000110274	-3.095327283
hsa-miR-5006-5p	5.482845561	12.6219798	13.75188942	0.000110364	-3.096325995
hsa-miR-33b-3p	5.872766428	7.162638025	13.73977069	0.00011077	-3.100852269
hsa-miR-107	-8.674325711	7.472460539	-13.65833269	0.000113551	-3.131368243
hsa-miR-30d-5p	-5.242261483	8.877193642	-13.56202541	0.000116951	-3.167681103
hsa-miR-4763-3p	-3.123912522	9.739559783	-13.52439036	0.000118314	-3.181938434
hsa-miR-6813-3p	4.238238461	5.988281481	13.43883706	0.000121485	-3.214490098
hsa-miR-7111-5p	-4.534796911	4.786481439	-13.40322882	0.000122835	-3.228096771
hsa-miR-4716-3p	-1.887184872	5.801638503	-13.39219794	0.000123257	-3.232318903
hsa-miR-7109-5p	5.033922883	6.313378875	13.38452551	0.000123552	-3.235257525
hsa-miR-6863	-4.472493167	4.558174683	-13.31282533	0.000126348	-3.262797366
hsa-miR-4738-3p	-2.50031875	5.378998514	-13.28453415	0.000127473	-3.273702848
hsa-miR-5195-5p	-2.786821439	3.291410914	-13.28411256	0.00012749	-3.273865528
hsa-miR-605-5p	4.335717133	5.179704733	13.25107001	0.000128819	-3.286630976
hsa-miR-6737-5p	5.060471611	4.229131428	13.18897799	0.000131364	-3.310701462
hsa-miR-1825	2.44698365	5.536680003	13.17382281	0.000131994	-3.31659288
hsa-miR-1247-3p	-3.55655225	4.030945736	-13.1269857	0.000133966	-3.334841188
hsa-miR-760	3.333884622	4.456847578	13.10733925	0.000134804	-3.342514144
hsa-miR-93-3p	-3.349425236	3.849819276	-13.05987178	0.000136855	-3.361097907
hsa-miR-6768-5p	3.447849778	7.700196339	13.04052234	0.000137703	-3.368691759
hur_4	3.442494156	9.713286206	13.03424887	0.000137979	-3.371156135
hsa-miR-6752-3p	4.362172128	6.006480486	13.02954295	0.000138186	-3.373005478
hsa-miR-3692-5p	3.477391622	7.682967506	13.01604246	0.000138784	-3.378314465
hsa-miR-1539	6.009022861	6.927491392	12.99891172	0.000139547	-3.38505857
hsa-miR-455-3p	-8.018121789	10.76620213	-12.90992412	0.000143593	-3.420228126
hsa-miR-3616-3p	2.07802945	3.550077625	12.89792078	0.00014415	-3.424989694
hsa-miR-6068	2.94864905	8.531363858	12.87644149	0.000145153	-3.433520769
hsa-miR-6855-3p	3.996399322	4.650635378	12.83487376	0.000147119	-3.450068967
hsa-miR-7150	5.975402611	6.395051089	12.77212754	0.000150148	-3.475145018
hsa-miR-3138	-1.697725367	7.325027889	-12.57464322	0.000160197	-3.554838135
hsa-miR-634	5.65978765	4.811984053	12.48540134	0.000165009	-3.591240875
hsa-miR-6780b-5p	2.270447806	10.53852685	12.44138888	0.000167448	-3.609284867
hsa-miR-221-3p	-7.122492578	6.794790539	-12.43473237	0.00016782	-3.612019125
hsa-miR-423-5p	-7.40609135	7.037062814	-12.4117925	0.000169112	-3.621452639
hsa-miR-148a-3p	-6.9178194	9.634935522	-12.3676003	0.000171636	-3.6396723
hsa-miR-6857-3p	3.526099344	3.854267056	12.3368259	0.000173421	-3.652396457
hsa-miR-26a-5p	-7.344052117	11.63921855	-12.3275602	0.000173963	-3.656233381
hsa-miR-199b-5p	-6.704986694	7.311248081	-12.31792312	0.000174529	-3.660226991
hsa-miR-1182	2.2109183	5.894434433	12.27392204	0.000177141	-3.678498673
hsa-miR-1470	4.040280017	5.184568269	12.18836798	0.00018236	-3.714203333
hsa-miR-214-5p	-3.476266878	5.599481661	-12.16484982	0.000183827	-3.724059754
hsa-miR-15a-5p	-7.474686522	6.7937208	-12.1584258	0.000184231	-3.726755179
hsa-miR-31-3p	-2.653487053	4.268254346	-12.13353628	0.000185804	-3.73721117
hsa-miR-6133	3.511352106	6.117101964	12.12513639	0.000186339	-3.740744497
hsa-miR-3663-3p	2.363254583	12.04103113	12.09365343	0.000188359	-3.754008081
hsa-miR-4294	2.819070967	4.023345794	12.02610888	0.000192787	-3.782574455
hsa-miR-8085	-6.274605233	6.284848483	-11.98468404	0.000195565	-3.800169055
hsa-miR-320c	1.871100867	16.24273179	11.85678856	0.00020446	-3.854854681

hsa-miR-93-5p	4.022878217	7.426022886	11.84211074	0.000205512	-3.861166127
hsa-miR-1281	-1.62446585	4.251420808	-11.82060508	0.000207066	-3.870426839
hsa-miR-296-5p	7.468313222	9.988892572	11.81467835	0.000207497	-3.872981781
hsa-miR-1226-5p	3.118623517	7.273930769	11.80857592	0.000207942	-3.875613728
hsa-miR-4739	4.020891183	13.1800232	11.78085614	0.000209977	-3.887585241
hsa-miR-27a-3p	-7.027874406	10.22518986	-11.78074426	0.000209985	-3.887633614
hsa-miR-564	-4.199784672	3.978881947	-11.76874606	0.000210873	-3.892823615
hsa-miR-4701-3p	6.602850683	8.817822364	11.72041936	0.000214499	-3.913778569
hsa-miR-6879-5p	4.483871328	6.007777697	11.69033143	0.000216795	-3.926866067
hsa-miR-8060	3.430216017	5.821555108	11.68961542	0.00021685	-3.927177895
hsa-miR-4665-5p	4.831235889	9.144041222	11.63715188	0.000220927	-3.950075443
hsa-miR-3141	5.484513233	12.40808994	11.62344292	0.000222008	-3.956074653
hsa-miR-320a	-5.854823167	9.741194328	-11.62285854	0.000222054	-3.956330529
hsa-miR-4468	-2.736077372	3.363908269	-11.52379213	0.000230063	-3.999883752
hsa-miR-625-5p	-4.125041689	3.950719767	-11.51173109	0.000231063	-4.005210197
hsa-miR-98-5p	-5.885407408	5.939730296	-11.45990495	0.000235417	-4.028157578
hsa-miR-6753-5p	5.554359328	5.166566614	11.44517973	0.000236673	-4.0346953
hsa-miR-6165	-3.783964311	9.759254911	-11.443307	0.000236833	-4.035527324
hsa-miR-7704	3.324316922	7.092294856	11.4240251	0.000238491	-4.044101377
hsa-miR-6819-5p	2.63096795	8.852250569	11.41692149	0.000239106	-4.047263546
hsa-miR-6862-5p	3.348693061	3.464746592	11.39744062	0.000240801	-4.055944914
hsa-miR-4507	-8.308022344	7.879164567	-11.37491349	0.000242779	-4.066001147
hsa-miR-6088	2.739043222	14.96013	11.34155432	0.000245746	-4.080927174
hsa-miR-146b-5p	-5.318359211	5.118285244	-11.33625135	0.000246222	-4.083303689
hsa-miR-20b-5p	-2.910447375	4.473077085	-11.2996272	0.000249538	-4.099745181
hsa-miR-4697-5p	-4.685659922	6.085638672	-11.23218502	0.00025579	-4.130152505
hsa-miR-4299	-6.035472589	10.15890923	-11.20037589	0.000258806	-4.14455342
hsa-miR-3656	-3.290973167	8.745631906	-11.15083864	0.00026359	-4.167056644
hsa-miR-7114-5p	3.617387972	5.697358108	11.14593846	0.000264069	-4.169287719
hsa-miR-1238-5p	4.260528356	5.288257461	11.11078578	0.000267537	-4.185319777
hsa-miR-210-3p	-3.869260494	6.571073036	-11.07939343	0.000270682	-4.199676937
hsa-miR-7114-3p	-3.575289078	4.012224783	-11.07302551	0.000271325	-4.202593912
hsa-miR-6884-3p	-4.3238237	4.663725661	-11.0646898	0.00027217	-4.206414643
hsa-miR-940	-3.010369917	6.089559008	-11.04402512	0.000274279	-4.215898032
hsa-miR-5571-5p	4.886849822	5.547419561	10.95680996	0.000283405	-4.256105766
hsa-miR-4312	4.8580208	5.494862411	10.94623434	0.000284537	-4.261001577
hsa-miR-5699-5p	2.26488545	8.198822764	10.92950416	0.000286339	-4.268755543
hsa-miR-6500-5p	5.676127683	4.972984375	10.92067658	0.000287295	-4.272851334
hsa-miR-4665-3p	4.615487128	9.105809542	10.91731262	0.00028766	-4.274412941
hsa-miR-6849-5p	3.447601472	5.933692708	10.9047621	0.000289029	-4.280243074
hsa-miR-510-3p	4.791487728	5.201627647	10.88159182	0.000291577	-4.291022862
hsa-miR-144-5p	-1.714221228	3.179037869	-10.87300617	0.000292528	-4.295022697
hsa-miR-4487	-1.921951817	2.880756636	-10.8611904	0.000293843	-4.300532167
hsa-miR-5010-5p	-3.477297583	3.724617786	-10.85742256	0.000294264	-4.302290212
hsa-miR-4721	-8.497909006	8.023690297	-10.83232262	0.000297086	-4.314016157
hsa-miR-99a-5p	4.235862622	8.157697922	10.77470619	0.000303693	-4.341028694
hsa-miR-4281	-5.656378633	12.63842999	-10.76585417	0.000304724	-4.345190719
hsa-miR-4513	-3.377142678	3.534499456	-10.74698727	0.000306935	-4.354072133
hsa-miR-6790-3p	5.088246944	5.312796961	10.697402	0.000312842	-4.377483171
hsa-miR-6746-5p	-3.647884978	4.118813189	-10.67446577	0.000315622	-4.388346317
hsa-miR-6763-3p	4.135986678	5.930861572	10.58715573	0.000326484	-4.429897528
hsa-miR-1260a	-4.339358267	4.8399939	-10.57562293	0.000327953	-4.435409773
hsa-miR-378c	2.172121133	3.634620261	10.50226388	0.000337489	-4.470603597

hsa-miR-1914-3p	6.198880161	11.95713511	10.49644124	0.000338261	-4.473406737
hsa-miR-106b-5p	-5.687222906	5.265740636	-10.48467126	0.000339826	-4.479077449
hur_5	-6.577574944	5.836425356	-10.47309986	0.000341375	-4.484658236
hsa-miR-1306-3p	7.944906922	8.750746194	10.41263396	0.000349607	-4.513913527
hsa-miR-4478	-7.658305572	7.035569253	-10.4055371	0.000350589	-4.517357507
hsa-miR-3937	-1.555235361	6.142494108	-10.39121266	0.000352582	-4.524315519
hsa-miR-4793-3p	-2.890552389	3.346302239	-10.32787464	0.000361561	-4.555188218
hsa-miR-7152-3p	5.477226706	8.554258714	10.30215015	0.000365288	-4.567777004
hsa-miR-770-5p	2.918976372	3.491677553	10.28891291	0.000367224	-4.574266219
hsa-miR-1343-5p	2.261049694	7.569486719	10.26217791	0.000371173	-4.587395887
hsa-miR-6861-5p	2.231422333	6.591739622	10.21839231	0.000377754	-4.608967472
hsa-miR-206	-3.869800733	5.243982733	-10.19031518	0.00038205	-4.622844949
hsa-miR-6782-5p	4.104202056	4.7603624	10.15331056	0.000387803	-4.641188859
hsa-miR-3976	6.379941592	6.763185024	10.14648023	0.000388876	-4.644581514
hsa-miR-194-5p	-1.448772494	3.156996992	-10.07548937	0.000400252	-4.679968015
hsa-miR-6887-5p	-6.984856522	6.395211961	-10.00077532	0.00041267	-4.717458908
hsa-miR-7847-3p	-3.466319189	8.861315211	-9.992675734	0.000414044	-4.721538661
hsa-miR-3154	3.199023306	6.461647714	9.981946246	0.000415873	-4.726947773
hsa-miR-378i	-5.294816878	8.107833644	-9.952905292	0.000420875	-4.741615161
hsa-miR-6510-5p	2.362282156	12.97059673	9.947479091	0.000421817	-4.744360066
hsa-miR-6760-3p	5.453198989	4.9802347	9.931616404	0.000424588	-4.752392271
hsa-miR-3679-5p	-3.818635178	9.606731767	-9.92870485	0.000425099	-4.753867839
hsa-miR-6872-3p	-3.750267439	4.187210331	-9.878384949	0.000434049	-4.779432735
hsa-miR-3654	-5.067655222	4.428614261	-9.864550564	0.000436551	-4.78648218
hsa-miR-939-5p	4.104485706	3.883775269	9.861156328	0.000437167	-4.78821313
hsa-miR-1307-5p	2.938177917	4.123011219	9.830078363	0.00044286	-4.804087309
hsa-miR-140-5p	-7.6651035	10.58944753	-9.796990143	0.000449023	-4.821038892
hsa-miR-8485	-1.633001144	4.155781339	-9.786372507	0.000451022	-4.82648957
hsa-miR-6766-3p	2.737413328	7.945188331	9.711984818	0.000465342	-4.864829703
hsa-miR-6875-5p	5.038686622	6.898835083	9.651211761	0.000477457	-4.89635243
hsa-miR-362-5p	-1.7249617	3.394839639	-9.64582065	0.00047855	-4.899157521
hsa-miR-4481	4.345128856	8.803299517	9.634124823	0.000480933	-4.905247991
hsa-miR-365a-3p	-5.560761139	8.383117753	-9.630576258	0.000481658	-4.9070972
hsa-miR-4664-3p	4.499517089	5.420675272	9.61356919	0.000485155	-4.915968471
hsa-miR-4515	-4.724300083	4.306038508	-9.596628224	0.000488669	-4.924819504
hsa-miR-4306	-7.555773944	6.895968728	-9.58865272	0.000490335	-4.928991345
hsa-miR-4485-3p	-2.87383005	5.963677992	-9.587817566	0.000490509	-4.929428382
hsa-miR-6747-5p	-4.180917822	4.573870078	-9.582343483	0.000491657	-4.932293834
hsa-miR-127-3p	-7.2799689	6.716023822	-9.577297001	0.000492718	-4.934936778
hsa-miR-6743-5p	3.773241117	5.762549736	9.547329361	0.000499075	-4.950657623
hsa-miR-663a	-4.499030078	4.691250356	-9.543291127	0.000499939	-4.952779493
hsa-miR-513b-5p	-5.994026761	5.595981903	-9.514774665	0.000506095	-4.967786641
hsa-miR-1288-3p	4.223639089	5.818048106	9.511561532	0.000506795	-4.969480159
hsa-miR-23a-3p	-14.74406994	11.95392383	-9.492205856	0.000511033	-4.979692816
hsa-miR-1587	4.106275928	14.35755204	9.488857449	0.000511771	-4.981461463
hsa-miR-4787-3p	-3.355420133	3.577468489	-9.445810717	0.000521372	-5.00424968
hsa-miR-7977	3.772167083	5.832769908	9.39826307	0.000532234	-5.029530597
hsa-miR-150-5p	-3.670413761	5.883567008	-9.376734586	0.000537244	-5.041015447
hsa-miR-3934-5p	-2.86166565	5.976586925	-9.368862561	0.00053909	-5.045220933
hsa-miR-4488	2.192710867	3.736546367	9.368569718	0.000539159	-5.04537744
hsa-miR-1275	4.831042833	13.51216758	9.361398574	0.000540847	-5.049211394
hsa-miR-4317	-3.444839706	5.595824086	-9.358745054	0.000541474	-5.050630738
hsa-miR-6129	3.75996905	5.889200575	9.349870339	0.000543576	-5.055380397

hsa-miR-6511b-5p	3.714851467	4.923044861	9.311754769	0.000552718	-5.075826082
hsa-miR-204-3p	4.734130722	5.49783675	9.305738529	0.000554178	-5.079060203
hsa-miR-194-3p	5.030205433	5.351124122	9.301241724	0.000555273	-5.081478766
hsa-miR-550a-5p	4.010490028	6.190930086	9.291039085	0.000557766	-5.08697009
hsa-miR-34b-5p	-2.358342178	3.822460983	-9.219272643	0.000575696	-5.12575171
hsa-miR-4701-5p	-2.26445675	2.912647531	-9.198776253	0.000580946	-5.136877846
hsa-miR-6075	-2.082700278	2.915037733	-9.187159155	0.000583948	-5.14319396
hsa-miR-572	2.839758289	6.318410856	9.181212168	0.000585492	-5.14643008
hsa-miR-1229-5p	2.947924783	12.97848966	9.17922324	0.00058601	-5.1475128
hsa-miR-181b-5p	-4.229699617	6.994059003	-9.161114264	0.000590747	-5.15738062
hsa-miR-920	-3.462143517	3.906523286	-9.157962671	0.000591576	-5.159099765
hsa-miR-6806-5p	-1.608167294	2.655458886	-9.068882197	0.000615618	-5.207914091
hsa-miR-543	3.364395217	4.401153908	9.027250109	0.000627264	-5.23087612
hsa-miR-3648	-2.242601322	7.197992544	-9.024533404	0.000628033	-5.232377824
hsa-miR-1260b	-4.272524789	4.424093917	-9.015198743	0.000630685	-5.237540817
hsa-miR-5684	3.918908717	5.751188092	9.010313195	0.000632079	-5.240244929
hsa-miR-20a-5p	-3.760302156	5.597861872	-9.009708214	0.000632252	-5.240579873
hsa-miR-642a-3p	1.429648622	15.82526261	8.965430584	0.000645059	-5.265149053
hsa-miR-497-5p	-6.000204056	8.740377878	-8.901118124	0.000664233	-5.301030438
hsa-miR-6076	2.977084172	9.865209092	8.866082223	0.000674972	-5.320675883
hsa-miR-6752-5p	2.174390756	10.53499644	8.818014049	0.000690055	-5.347742484
hsa-miR-6865-5p	-1.734662606	5.477110747	-8.816730359	0.000690463	-5.348467126
hsa-miR-6512-5p	1.9593823	7.198999928	8.784122599	0.000700937	-5.366905965
hsa-miR-1229-3p	-1.865327272	2.879091847	-8.748758365	0.000712518	-5.38697294
hsa-miR-6758-5p	4.198691133	5.86117195	8.744202395	0.000714027	-5.389563442
hsa-miR-1208	-2.826734603	3.295373274	-8.66528206	0.000740796	-5.434629763
hsa-miR-4539	-3.077379189	3.735566839	-8.647192989	0.000747104	-5.445010902
hsa-miR-660-5p	-4.23646435	4.000563392	-8.643195119	0.000748507	-5.447307855
hsa-miR-6085	1.850760544	14.20077481	8.617710704	0.000757526	-5.461972098
hsa-miR-1-3p	-3.903586272	4.302102919	-8.606804215	0.000761427	-5.468259731
hsa-miR-670-5p	-2.513209333	3.076861122	-8.551468503	0.000781601	-5.500270803
hsa-miR-502-3p	-2.727154031	3.221100554	-8.516919473	0.000794529	-5.520350664
hsa-miR-4304	-2.149098044	2.967382767	-8.497648643	0.000801853	-5.531582347
hsa-miR-4743-5p	-3.8598675	4.242587972	-8.483838211	0.000807153	-5.539645464
hsa-miR-4451	-1.351534211	3.8469176	-8.460895427	0.000816053	-5.553066248
hsa-miR-6858-3p	4.019195256	4.02546355	8.447602165	0.000821265	-5.560857169
hsa-miR-374b-5p	-4.161189839	6.473641031	-8.433070473	0.000827008	-5.569386369
hsa-miR-6850-5p	2.394785672	9.652436992	8.398224059	0.000840983	-5.589892287
hsa-miR-4535	2.1191365	5.919388572	8.384088716	0.000846735	-5.598231961
hsa-miR-376a-3p	-3.834484522	6.498385639	-8.373599115	0.000851034	-5.60442875
hsa-miR-345-5p	5.303864978	7.395036222	8.36441609	0.000854819	-5.609859319
hsa-miR-513c-5p	-5.729763356	5.452084278	-8.34759103	0.000861809	-5.619822872
hsa-miR-6086	2.506614328	9.574056253	8.323409924	0.000871977	-5.634173739
hsa-miR-145-5p	-4.396319578	8.323963794	-8.312481911	0.00087662	-5.640671337
hsa-miR-4419b	4.447541239	7.741635425	8.312095572	0.000876785	-5.640901185
hsa-miR-5190	-3.184887528	3.413617636	-8.30734857	0.000878811	-5.643726136
hsa-miR-574-5p	-7.351145783	6.996403853	-8.282425112	0.000889548	-5.658581588
hsa-miR-31-5p	-4.181668583	6.486330386	-8.274061476	0.000893187	-5.663575525
hsa-miR-4485-5p	-4.515510978	8.3010595	-8.258666756	0.000899932	-5.672779396
hsa-let-7f-1-3p	2.741813661	4.642704392	8.237536973	0.000909292	-5.685436714
hsa-miR-6856-5p	2.336099861	5.157507853	8.231049706	0.00091219	-5.689328512
hsa-miR-299-5p	-1.696986369	3.357276035	-8.221608535	0.000916427	-5.694997231
hsa-miR-4640-5p	2.637753483	4.004666681	8.212252333	0.00092065	-5.700620599

hsa-miR-6728-5p	-2.937759322	8.044359089	-8.16376891	0.000942918	-5.729851372
hsa-miR-6812-5p	2.616424472	11.03101505	8.148903644	0.000949877	-5.738844293
hsa-miR-23c	3.754610411	5.023080217	8.129202462	0.000959197	-5.750785021
hsa-miR-152-3p	-5.366644456	7.901215822	-8.124207676	0.000961578	-5.753816364
hsa-miR-3132	3.005238922	6.324771922	8.117302774	0.000964881	-5.758009658
hsa-miR-6886-3p	2.515036617	3.039802825	8.110933958	0.00096794	-5.761880171
hsa-miR-6767-5p	3.649155556	7.693237694	8.104578551	0.000971004	-5.765745198
hsa-miR-125b-1-3p	8.101040472	7.962385858	8.096098935	0.000975111	-5.770906206
hsa-miR-363-3p	-2.871219725	4.554193115	-8.084182668	0.000980918	-5.778166916
hsa-miR-331-3p	-3.481406728	5.897047853	-8.071093773	0.000987346	-5.786152956
hsa-miR-619-5p	-3.402530906	3.585361925	-8.070394035	0.000987691	-5.786580213
hsa-miR-7152-5p	2.710723244	3.351957367	8.047477862	0.000999074	-5.800590727
hsa-miR-4773	2.208130183	5.939154264	8.024785399	0.001010504	-5.814498985
hsa-miR-2467-3p	-1.732825978	2.769293817	-8.015772549	0.001015088	-5.820032544
hsa-miR-3180-3p	3.761328278	5.891304506	7.986292533	0.001030261	-5.838170328
hsa-miR-4433a-5p	4.436023256	8.537746156	7.962727319	0.001042589	-5.852711177
hsa-miR-92a-3p	2.653037772	5.476405725	7.956725392	0.001045757	-5.856420661
hsa-miR-6734-5p	2.37795975	8.156764342	7.945603035	0.001051661	-5.863301294
hsa-miR-3161	2.457567583	4.054566858	7.939041972	0.001055162	-5.867364117
hsa-miR-1225-5p	2.830414078	15.15955157	7.916668483	0.00106721	-5.881240598
hsa-miR-936	4.565164883	4.847012519	7.915456767	0.001067867	-5.881993105
hsa-miR-6732-5p	-1.93373095	2.919572192	-7.894234368	0.001079459	-5.895189099
hsa-miR-15b-5p	-7.4728635	10.60530807	-7.882496309	0.001085937	-5.902501071
hsa-miR-5008-5p	-3.735214311	4.051248383	-7.8725302	0.001091474	-5.9087167
hsa-miR-483-5p	-3.658746189	10.6110784	-7.851941738	0.001103022	-5.921578993
hsa-miR-21-3p	-1.690117761	3.419279403	-7.829491122	0.001115786	-5.935638159
hsa-miR-4430	-1.760804106	6.386114164	-7.795247547	0.001135604	-5.957150099
hsa-miR-6797-5p	-1.542767017	3.980630414	-7.787663385	0.001140051	-5.96192561
hsa-miR-143-3p	-2.558538	3.261973294	-7.782052333	0.001143355	-5.965461316
hsa-miR-451b	-1.128419267	3.390172544	-7.741171932	0.001167782	-5.99128843
hsa-miR-6883-5p	-4.43662585	4.499167419	-7.686118431	0.001201695	-6.026257305
hsa-miR-181c-5p	-5.975134372	4.699503236	-7.672240907	0.001210432	-6.035106261
hsa-miR-584-5p	-3.596854811	3.660258856	-7.629821368	0.001237626	-6.062241043
hsa-miR-381-3p	-2.397615922	4.640325761	-7.618992339	0.001244687	-6.069189007
hsa-miR-1227-5p	2.879127489	8.670252217	7.580373988	0.001270273	-6.09403636
hsa-miR-4800-5p	4.401130706	13.14687387	7.552932598	0.001288844	-6.1117588
hsa-miR-28-5p	-2.831242669	4.643977949	-7.551649053	0.001289721	-6.112589108
hsa-miR-6769a-5p	3.595697333	7.556864861	7.543775924	0.001295115	-6.117684789
hsa-miR-933	2.671463556	4.775925989	7.53420969	0.001301706	-6.123882454
hsa-miR-2861	-3.804778167	9.562150567	-7.533848952	0.001301955	-6.124116297
hsa-miR-376c-3p	-4.708504289	7.436883217	-7.530281774	0.001304424	-6.12642919
hsa-miR-30a-5p	-5.044048011	8.378275917	-7.523879725	0.001308869	-6.130582527
hsa-miR-6795-3p	5.268610206	5.204356153	7.504500016	0.001322438	-6.143173692
hsa-miR-1246	5.908224511	16.39398816	7.48364272	0.001337234	-6.156756127
hsa-miR-4444	2.955729772	4.586963775	7.482430109	0.0013381	-6.157546788
hsa-miR-550a-3-5p	3.645485844	6.118821972	7.481591781	0.001338699	-6.15809347
hsa-miR-23a-5p	-1.483940011	4.207013861	-7.480687598	0.001339346	-6.158683155
hsa-miR-19b-3p	-5.130085539	7.957249536	-7.476133631	0.00134261	-6.161654067
hsa-miR-187-5p	1.971193028	3.696453758	7.454745045	0.001358069	-6.175628347
hsa-miR-6798-5p	-3.523095717	4.015157019	-7.448902784	0.001362329	-6.179451383
hsa-miR-1304-3p	3.834673844	6.042140022	7.412674808	0.001389114	-6.203215668
hsa-miR-30b-5p	-4.687357517	6.959878219	-7.379863209	0.00141393	-6.22482493
hsa-miR-495-3p	-2.768144914	4.662560504	-7.351617659	0.001435727	-6.243492974

hsa-miR-29b-3p	-3.614460994	5.839012197	-7.330269168	0.001452474	-6.257643314
hsa-miR-5196-5p	4.013469994	5.964384564	7.283985859	0.001489608	-6.288442247
hsa-miR-4324	-4.122601361	6.574924542	-7.271188483	0.001500079	-6.296987605
hsa-miR-1271-5p	-3.057933239	3.332735242	-7.240653602	0.001525429	-6.317428875
hsa-miR-4689	-1.428731444	5.744567711	-7.240453145	0.001525597	-6.317563311
hsa-miR-4484	-2.239304039	6.219839081	-7.239944599	0.001526024	-6.31790438
hsa-miR-6795-5p	-2.749253817	3.218703947	-7.124975748	0.001626329	-6.395538087
hsa-miR-223-3p	-5.561533161	8.395994853	-7.106889084	0.001642832	-6.407847682
hsa-miR-148b-3p	-3.844370844	6.175864122	-7.098657163	0.001650411	-6.413459008
hsa-miR-622	3.847635228	5.922856225	7.093550138	0.001655134	-6.416942998
hsa-miR-6716-3p	-1.637434911	4.067386233	-7.083567376	0.001664414	-6.423759314
hsa-miR-3124-5p	4.720545317	5.461360003	7.075571548	0.001671893	-6.429224782
hsa-miR-4442	3.623615767	13.13949067	7.013660809	0.001731209	-6.471720353
hsa-miR-342-3p	-6.296293317	9.264991314	-7.010864433	0.001733949	-6.473647229
hsa-miR-4463	-1.66615435	5.800761897	-6.983726654	0.001760808	-6.492380476
hsa-miR-324-5p	-3.233792569	5.240083554	-6.96392014	0.001780732	-6.50609157
hsa-miR-1238-3p	3.462483883	6.380602731	6.945787149	0.001799214	-6.518672879
hsa-miR-2276-3p	-1.995853144	6.64627325	-6.93999902	0.001805163	-6.522694685
hsa-miR-890	-4.008272111	4.390357606	-6.904799212	0.001841859	-6.547213471
hsa-miR-125a-5p	-6.363909944	6.357697572	-6.903409461	0.001843327	-6.548183661
hsa-miR-3665	-3.631872161	8.385000331	-6.889177329	0.001858436	-6.55812857
hsa-miR-4659a-3p	-3.6771337	4.202489411	-6.847037408	0.001904068	-6.587675412
hsa-miR-4313	-3.226446367	3.664581556	-6.816229562	0.001938294	-6.609372765
hsa-miR-1273g-3p	2.2617997	15.97363265	6.782479518	0.001976652	-6.633236142
hsa-miR-4769-3p	1.85264805	5.577823231	6.77916703	0.001980466	-6.635583593
hsa-miR-3907	-1.590443278	4.321762456	-6.706629376	0.002066287	-6.687228777
hsa-miR-128-3p	-3.2456183	5.1598385	-6.6454379	0.002142225	-6.731156425
hsa-miR-4649-3p	-2.288110828	2.870283331	-6.615493648	0.002180623	-6.752774277
hsa-miR-4286	-3.240850561	5.901473292	-6.568182348	0.002243012	-6.787094682
hsa-miR-151a-3p	-4.3826325	7.323538789	-6.550386417	0.00226704	-6.800056698
hsa-miR-4291	-4.422882828	7.472527186	-6.542530873	0.002277746	-6.805787626
hsa-miR-1321	2.527418483	4.007679058	6.527300744	0.002298678	-6.816914682
hsa-miR-6894-5p	2.338494272	3.426464086	6.526852925	0.002299297	-6.817242178
hsa-miR-8071	-4.325874239	4.767967397	-6.522430832	0.002305421	-6.8204771
hsa-miR-4730	-1.365354506	3.658206503	-6.520865411	0.002307594	-6.821622693
hsa-miR-6741-5p	1.570577311	6.707410039	6.480668974	0.002364241	-6.851116205
hsa-miR-22-5p	-2.264044414	4.204078504	-6.450311242	0.002408146	-6.873489868
hsa-miR-3926	-2.403354733	4.693272228	-6.435730702	0.002429586	-6.884266251
hsa-miR-6826-5p	2.567889122	8.79592175	6.433758498	0.002432504	-6.88572542
hsa-miR-7159-5p	-2.657159789	5.206341878	-6.426932589	0.002442635	-6.890778497
hsa-miR-4314	-1.398312844	4.609642522	-6.412493115	0.002464237	-6.901482137
hsa-miR-3194-5p	2.332331939	8.072184442	6.394370824	0.002491678	-6.914943512
hsa-miR-5787	2.335644017	16.07184377	6.381018212	0.002512134	-6.924881763
hsa-miR-2116-3p	3.048383906	5.005944786	6.349877943	0.002560641	-6.948124911
hsa-miR-4698	6.0583609	12.03170438	6.306002789	0.002630931	-6.981030407
hsa-miR-6880-3p	3.159991006	3.829323931	6.297407301	0.002644974	-6.987498499
hsa-miR-6877-3p	-3.925536794	4.194468869	-6.271457655	0.002687928	-7.00706884
hsa-miR-4476	-1.575072672	5.593398736	-6.270572769	0.002689408	-7.00773734
hsa-miR-139-3p	2.312266322	7.6770941	6.205660196	0.002800698	-7.056984569
hsa-miR-6889-3p	2.7372668	5.732444117	6.203928544	0.002803743	-7.058303971
hsa-miR-500a-5p	2.644969522	4.837920956	6.202314606	0.002806584	-7.05953395
hsa-miR-4749-3p	3.165628617	6.137547792	6.19398215	0.002821309	-7.065888175
hsa-miR-3202	-1.547337611	6.107620072	-6.188093087	0.002831772	-7.070383228

hsa-miR-1469	-2.166731811	4.87443645	-6.150577251	0.002899548	-7.09909921
hsa-miR-659-3p	2.53972875	7.065876236	6.101627658	0.002990966	-7.136777863
hsa-miR-4259	1.9618606	4.450242189	6.089202042	0.003014726	-7.146380673
hsa-miR-4499	-2.3933176	7.561340122	-6.08210009	0.003028409	-7.151876224
hsa-miR-1234-3p	2.556783022	7.413774744	6.076256216	0.003039725	-7.156402085
hsa-miR-590-5p	-1.264340122	1.936572294	-6.074223399	0.003043674	-7.157977236
hsa-miR-3667-5p	-1.653156017	6.967652292	-6.067325944	0.003057117	-7.163324923
hsa-miR-505-3p	-2.890101189	4.930996994	-6.008904699	0.003173919	-7.208813844
hsa-miR-6794-5p	-2.659165278	8.165538233	-5.990215587	0.003212422	-7.223439649
hsa-miR-6515-3p	1.654980239	6.971232286	5.97370427	0.003246911	-7.236391102
hsa-miR-1228-3p	2.685580589	8.104062989	5.972169975	0.003250139	-7.237596032
hsa-miR-6780a-5p	-2.584579267	4.455939111	-5.971815145	0.003250886	-7.237874726
hsa-miR-638	2.241683533	11.20852044	5.942719643	0.003312852	-7.26077162
hsa-miR-4455	-2.995554044	5.999282983	-5.919148196	0.003364106	-7.279385962
hsa-miR-151a-5p	-7.715610772	6.440191992	-5.890382687	0.003427963	-7.302180903
hsa-miR-3131	-1.880130761	6.902290553	-5.844234456	0.003533507	-7.338932986
hsa-miR-3653-3p	-4.444676556	7.943800683	-5.83593399	0.003552906	-7.345567398
hsa-miR-196b-5p	-3.858799039	5.961596586	-5.831235537	0.003563945	-7.349326042
hsa-miR-6870-3p	2.47703475	4.017819725	5.816643415	0.003598492	-7.361014404
hsa-miR-361-5p	-4.220615394	6.828201375	-5.814254639	0.003604186	-7.362929996
hsa-miR-6737-3p	-2.756251089	3.316294261	-5.809263468	0.003616119	-7.366934456
hsa-miR-30c-5p	-4.733178456	7.307846106	-5.797343098	0.003644811	-7.376509089
hsa-miR-602	4.676586128	5.436119492	5.79372482	0.003653575	-7.37941837
hsa-let-7b-3p	1.833142589	4.420899206	5.766809077	0.003719575	-7.401104299
hsa-miR-4660	4.117063694	5.575329864	5.720121515	0.003837518	-7.438906577
hsa-miR-1290	-7.887376111	7.143363478	-5.687724029	0.003922027	-7.465278322
hsa-miR-3117-3p	3.7644817	4.949428867	5.682962351	0.003934637	-7.469164078
hsa-miR-487b-3p	-2.642409017	4.698992647	-5.629922263	0.004078472	-7.512616901
hsa-miR-30e-3p	-2.537795564	4.556333818	-5.626479817	0.004088025	-7.51544791
hsa-miR-4672	-2.233703022	4.973028744	-5.621787299	0.004101091	-7.519309087
hsa-miR-345-3p	-1.879358183	5.743348514	-5.581167859	0.004216332	-7.552835324
hsa-miR-328-5p	-2.980734689	9.576005289	-5.572481472	0.004241482	-7.560028921
hsa-miR-6727-5p	2.366385733	11.55044831	5.506296524	0.004439189	-7.615120613
hsa-miR-324-3p	-4.019502522	7.3941582	-5.478063282	0.004526908	-7.63877391
hsa-miR-6812-3p	-1.975218561	2.918255319	-5.473208417	0.004542202	-7.642850467
hsa-miR-451a	-14.09717549	16.64376933	-5.46552263	0.004566542	-7.649309665
hsa-miR-4710	-2.556633144	3.243998839	-5.46177799	0.004578458	-7.652459163
hsa-miR-6786-5p	-2.735295367	8.101795028	-5.416531431	0.004725452	-7.690643133
hsa-miR-449c-3p	2.599015217	4.149152392	5.413624936	0.004735088	-7.693104094
hsa-miR-197-3p	-2.356793028	4.255936731	-5.408891995	0.00475083	-7.69711364
hsa-miR-452-5p	-1.893786239	2.942615914	-5.407393084	0.004755829	-7.698384
hsa-miR-382-5p	-2.212881883	4.631820925	-5.305892441	0.005109601	-7.785023782
hsa-miR-7110-5p	3.265157622	8.427292972	5.304105761	0.005116108	-7.786559822
hsa-miR-193a-5p	-2.753911011	5.60531625	-5.303454681	0.005118481	-7.787119662
hsa-miR-5096	3.236178294	5.184998442	5.275076082	0.005223241	-7.811570728
hsa-miR-6083	2.631975956	5.408261794	5.271854428	0.005235297	-7.814352626
hsa-miR-601	-1.867453456	6.285614828	-5.246360297	0.005331891	-7.83641093
hsa-miR-4422	-1.328185106	4.810721014	-5.201901791	0.005505535	-7.875066231
hsa-miR-3180-5p	2.160104661	4.981372047	5.18171964	0.005586609	-7.892693535
hsa-miR-7106-5p	-1.788496744	6.231876939	-5.155609459	0.005693643	-7.915572606
hsa-miR-6724-5p	2.650883717	11.01223938	5.149418074	0.005719385	-7.92101013
hsa-miR-889-3p	1.905028411	3.631703194	5.144235606	0.00574104	-7.92556522
hsa-miR-664b-5p	-4.626627622	4.788223233	-5.13526707	0.005778749	-7.933455894

hsa-miR-4687-3p	2.87932605	16.9908712	5.101869032	0.005921827	-7.962927837
hsa-miR-7107-5p	-5.565481111	13.15830403	-5.098981643	0.005934396	-7.96548232
hsa-miR-4433a-3p	-1.436901756	6.419946461	-5.062662174	0.006095274	-7.997703316
hsa-miR-5093	3.771261017	4.992890031	5.060724878	0.006104002	-7.999426645
hsa-miR-3190-5p	2.227554728	4.356744814	5.059159058	0.006111068	-8.000819872
hsa-miR-6808-5p	2.308457211	6.784275772	5.04696215	0.006166442	-8.011682933
hsa-miR-181a-3p	-2.581273975	4.420406557	-5.030689735	0.006241262	-8.026205029
hsa-let-7b-5p	-25.01169358	17.23864665	-4.990859936	0.006429062	-8.061892188
hsa-miR-144-3p	-2.246704806	3.737475047	-4.968566469	0.006537145	-8.08195517
hsa-miR-1273c	5.084719078	10.84299931	4.956087887	0.006598598	-8.093213037
hsa-miR-4446-3p	-2.051152083	4.320952797	-4.954526215	0.006606338	-8.094623347
hsa-miR-218-5p	-2.920309808	5.026689924	-4.953062279	0.006613603	-8.095945677
hsa-miR-361-3p	-2.564381192	4.667834443	-4.938209501	0.006687859	-8.109377344
hsa-miR-30e-5p	-2.858724717	5.161911408	-4.935722699	0.00670039	-8.111628986
hsa-miR-4644	-1.367306933	4.830503444	-4.927553846	0.006741751	-8.119030983
hsa-miR-766-3p	-1.295584378	3.686855133	-4.915973155	0.006800914	-8.129539306
hsa-miR-1183	1.78077025	6.953131564	4.912988422	0.006816263	-8.132250466
hsa-miR-4522	-4.762532189	4.980638211	-4.912146723	0.0068206	-8.133015226
hsa-miR-6833-5p	2.607737022	10.22766053	4.889554144	0.006938236	-8.153576983
hsa-miR-664b-3p	-1.071556828	2.307673019	-4.849738266	0.007151548	-8.189975754
hsa-miR-6749-5p	1.207120278	17.22676758	4.833222751	0.007242337	-8.205134792
hsa-miR-132-3p	-3.373475364	3.922907065	-4.832095671	0.007248583	-8.206170608
hsa-miR-4668-5p	-2.69812	5.375500667	-4.775753062	0.007569239	-8.258164821
hsa-miR-494-3p	-13.11455442	11.39698272	-4.707036796	0.007983681	-8.32214995
hsa-miR-4788	2.862576067	13.47102072	4.695812271	0.00805394	-8.332661929
hsa-miR-424-3p	-1.420793033	3.479106833	-4.672979919	0.008199159	-8.354097422
hsa-miR-4270	-4.103599172	11.02256299	-4.665420188	0.008247932	-8.361210228
hsa-miR-423-3p	1.846381894	3.828318225	4.653823304	0.008323428	-8.372136607
hsa-miR-29a-3p	-7.710169639	12.23644271	-4.651678144	0.008337484	-8.374159746
hsa-miR-3162-3p	-1.429072	3.985087267	-4.629845718	0.008482168	-8.394785981
hsa-miR-30c-2-3p	-1.0980853	4.620770333	-4.608395716	0.008627259	-8.415114399
hsa-miR-6516-5p	-1.067664767	2.288814406	-4.581876618	0.008810771	-8.440334206
hsa-miR-4261	-1.278118106	3.297993997	-4.575203337	0.008857685	-8.446695792
hsa-miR-3591-3p	-1.124131622	3.115778633	-4.573338642	0.008870847	-8.44847449
hsa-miR-6800-3p	-2.152709822	3.035208672	-4.57017227	0.008893251	-8.451495934
hsa-miR-6847-5p	1.829883406	6.758352692	4.50844521	0.009343853	-8.510675492
hsa-miR-4497	-1.973455661	6.526016286	-4.444005606	0.009843731	-8.573023755
hsa-miR-4769-5p	-1.63143385	5.140056925	-4.440766454	0.009869692	-8.576173215
hsa-miR-3125	-1.938152606	5.007831097	-4.427052074	0.009980519	-8.589524286
hsa-miR-4778-5p	-2.976306711	8.990021728	-4.417513899	0.010058475	-8.598825491
hsa-miR-3652	-2.619478894	6.890469814	-4.381831221	0.010356643	-8.633736302
hsa-miR-484	-1.440945144	3.765328828	-4.376039007	0.010406034	-8.639420339
hsa-miR-6824-5p	1.848392706	6.601857658	4.34669054	0.01066066	-8.668294441
hsa-miR-6124	2.926752444	14.86435028	4.333318929	0.010779135	-8.681490884
hsa-miR-6870-5p	3.848571383	4.873067069	4.319580744	0.010902499	-8.695075883
hsa-miR-6784-5p	-1.510995244	5.368835456	-4.290903687	0.011165483	-8.723520906
hsa-miR-330-3p	-1.428404447	3.327217435	-4.280770972	0.011260211	-8.733600074
hsa-miR-8064	-1.507798022	5.281317756	-4.255862901	0.011497174	-8.758439837
hsa-miR-378d	-2.581632911	4.1285823	-4.253290345	0.011521985	-8.761010476
hsa-miR-3135b	-4.114437928	7.739811581	-4.218130434	0.011867571	-8.796240918
hsa-miR-6788-5p	1.795543056	6.591606972	4.208255317	0.011966847	-8.806168344
hsa-miR-6738-5p	1.605576761	6.099870219	4.191324814	0.012139363	-8.82322179
hsa-miR-4758-5p	6.610437183	11.81201393	4.158590213	0.012481396	-8.856313581

hsa-miR-142-3p	-2.005220017	3.569225108	-4.155911664	0.012509888	-8.859028346
hsa-miR-30a-3p	-2.510662619	4.762867529	-4.135492385	0.012729653	-8.879758539
hsa-miR-6087	1.451093611	16.72157425	4.107105698	0.013042852	-8.90868001
hsa-miR-6730-3p	-1.058727539	2.308057803	-4.092298203	0.013209863	-8.923813942
hsa-miR-188-5p	5.184627283	13.86146491	4.065371054	0.013520134	-8.951418371
hsa-miR-3190-3p	-1.435745894	2.283817631	-4.065306864	0.013520884	-8.951484304
hsa-miR-377-3p	-2.083955044	4.202727592	-4.022971542	0.014026434	-8.9951039
hsa-miR-4428	4.179939356	14.26152966	4.020058215	0.014062042	-8.99811548
hsa-miR-374c-5p	-2.066745369	3.961800957	-4.017577493	0.014092447	-9.00068087
hsa-miR-19a-3p	-2.335140461	4.202983111	-4.016350124	0.01410752	-9.00195047
hsa-miR-6817-5p	-3.088458606	3.500372514	-3.999732461	0.014313484	-9.0191622
hsa-miR-4755-3p	-6.871539383	5.877333247	-3.973947992	0.014640178	-9.045950706
hsa-miR-642a-5p	-1.114709622	2.329688211	-3.917784659	0.015382914	-9.104648523
hsa-miR-501-5p	-1.921258017	2.751539064	-3.891213212	0.015749837	-9.132585643
hsa-miR-1973	-2.9098195	5.195290206	-3.875392798	0.015973225	-9.149270142
hsa-miR-425-3p	1.869361661	4.405451003	3.866200665	0.016104744	-9.158981836
hsa-let-7d-5p	1.549151417	13.51216104	3.86562018	0.016113092	-9.159595564
hsa-miR-410-3p	-2.243542983	4.234315283	-3.836727556	0.01653517	-9.190207712
hsa-miR-23b-5p	-2.273415017	4.382759903	-3.797465867	0.017129887	-9.232010446
hsa-miR-6509-5p	-1.128971878	2.400487889	-3.788347616	0.017271604	-9.241752599
hsa-miR-6891-3p	2.16510215	3.323264975	3.772770599	0.017516925	-9.258424933
hsa-miR-6511a-5p	-1.259049772	2.430842475	-3.771923168	0.017530389	-9.25933302
hsa-miR-193b-3p	-4.598413106	7.956054275	-3.731917874	0.018180129	-9.302327192
hsa-miR-630	2.479757983	11.64148037	3.706499273	0.018607746	-9.32977269
hsa-miR-340-5p	-2.299785889	4.297553231	-3.672241743	0.019202988	-9.366919318
hsa-miR-6757-3p	-1.155791278	2.371295006	-3.670087273	0.019241168	-9.369261528
hsa-miR-6736-5p	2.106782128	6.747389197	3.666342424	0.019307744	-9.373334407
hsa-miR-654-3p	2.309317728	7.718457764	3.660763436	0.019407433	-9.379406095
hsa-miR-4450	-1.286299539	3.791109086	-3.637862813	0.019823044	-9.404379448
hsa-miR-551b-5p	-1.251230306	2.435135081	-3.61604408	0.020228809	-9.428248262
hsa-miR-4436b-5p	-1.180232556	3.291158911	-3.572685173	0.021064559	-9.475899288
hsa-miR-96-5p	-3.69065925	4.187105419	-3.571192168	0.021094053	-9.477545256
hsa-miR-335-5p	-1.477778272	3.832791456	-3.537705085	0.021768521	-9.514553634
hsa-miR-1237-3p	2.182917722	5.735564228	3.535306806	0.021817791	-9.517210745
hsa-miR-3960	1.185793	19.1435595	3.531954873	0.021886872	-9.52092592
hsa-miR-656-3p	-1.219747181	2.427832332	-3.485723766	0.022866374	-9.57234389
hsa-miR-4417	-2.346914806	7.039087031	-3.427715967	0.024169105	-9.637325952
hsa-miR-202-3p	-2.395268633	5.206322489	-3.404233513	0.02472108	-9.663778946
hsa-miR-4725-5p	-1.801985128	2.865900925	-3.383420689	0.025222677	-9.687295401
hsa-miR-1207-5p	2.153687428	16.1452939	3.356769968	0.025882465	-9.717505084
hsa-miR-432-5p	-2.450913169	4.441391507	-3.326161126	0.026665299	-9.752335525
hsa-miR-221-5p	-2.139316344	3.670202606	-3.224817002	0.029461829	-9.868674128
hsa-miR-6765-3p	-1.74084065	2.767008781	-3.215063599	0.029748578	-9.879952496
hsa-miR-4682	-1.265143194	2.412292547	-3.209126118	0.02992473	-9.886825312
hsa-miR-4310	-1.420109589	2.560464761	-3.182641901	0.030725392	-9.917545938
hsa-miR-4290	-1.174063383	3.550186536	-3.174906815	0.030963919	-9.92653811
hsa-miR-154-5p	-2.390419978	4.522560969	-3.160803989	0.031404372	-9.942955831
hsa-miR-378f	-1.063864244	3.356661378	-3.12245027	0.03263952	-9.987754327
hsa-miR-424-5p	-2.868822222	4.797729778	-3.090068873	0.033726387	-10.02574574
hsa-miR-149-5p	-3.016498922	4.963047194	-3.071345095	0.034373971	-10.04778336
hsa-miR-6777-5p	-1.51615835	2.592336303	-3.03209209	0.035778895	-10.09414865
hsa-miR-4732-5p	-2.479504394	5.212653481	-3.012592482	0.036501449	-10.11726385
hsa-miR-197-5p	3.200023933	17.68067836	2.947495688	0.039038588	-10.19482151

hsa-miR-3666	-1.888857728	2.738170219	-2.925443942	0.03994375	-10.22122894
hsa-miR-539-5p	-1.664203706	2.639061081	-2.903559529	0.040866019	-10.24750227
hsa-miR-6745	-1.230238917	5.241725303	-2.876700052	0.042031626	-10.27983771
hsa-miR-5739	1.477240394	14.16584814	2.868797806	0.042381799	-10.28936958
hsa-miR-2277-3p	2.691674811	5.564641206	2.849871849	0.043234174	-10.3122325
hsa-miR-6751-3p	-1.089597356	2.22472185	-2.849672203	0.04324327	-10.31247394
hsa-miR-3605-5p	-1.589472933	4.880747022	-2.829366162	0.044179984	-10.33705745
hsa-miR-4746-5p	-3.153144906	5.738181831	-2.815317654	0.044841661	-10.35409701
hsa-miR-4534	-2.07352585	7.698543869	-2.793283511	0.045902459	-10.38087407
hsa-miR-135a-3p	3.745214756	10.99279318	2.776294949	0.046739981	-10.40156208
hsa-miR-3193	-1.160931406	2.332693853	-2.771104361	0.046999349	-10.40789031
hsa-miR-665	-1.51720975	4.125984103	-2.765581579	0.047277125	-10.41462728
hsa-miR-133b	-3.5049179	5.421413728	-2.747528909	0.048198286	-10.43667558
hsa-miR-4274	-1.456881906	4.905821597	-2.721036192	0.049587468	-10.46910526
hsa-miR-493-5p	-2.287147097	3.773569549	-2.719308382	0.049679644	-10.47122326
hsa-miR-16-5p	2.170767889	16.88339511	2.71062409	0.050145903	-10.48187421
hsa-miR-8055	2.135725294	4.263581103	2.696785863	0.050899222	-10.49886509
hsa-miR-181d-5p	-2.538257378	4.297525461	-2.637709622	0.054263433	-10.57165425
hsa-miR-103a-3p	1.973244744	13.41424119	2.590079669	0.057160346	-10.63062878
hsa-miR-3651	-3.67000075	6.337825503	-2.560085307	0.059074429	-10.66789321
hsa-miR-4673	2.095693339	4.945821564	2.546132394	0.059989452	-10.68526004
hsa-miR-1181	2.500142967	9.137710539	2.536424112	0.06063556	-10.6973554
hsa-miR-516a-5p	-2.193805356	7.681020067	-2.519153463	0.061804467	-10.71889589
hsa-miR-4284	-2.6820805	5.497560433	-2.510445514	0.062403458	-10.72976785
hsa-miR-204-5p	-3.561982417	3.289321575	-2.509711772	0.062454227	-10.73068427
hsa-miR-548u	-1.012854836	2.229566338	-2.507717443	0.062592456	-10.73317538
hsa-miR-6769b-5p	1.540029972	14.47720531	2.409843401	0.069818432	-10.85587722
hsa-miR-422a	-1.073480717	4.595438336	-2.356813148	0.074121095	-10.92269287
hsa-miR-3149	-2.069564789	4.341081994	-2.339551607	0.075584852	-10.94448618
hsa-miR-2392	-2.290728111	7.962027267	-2.285088386	0.080418287	-11.01337587
hsa-miR-6740-5p	1.822643206	13.53871809	2.281825499	0.080718571	-11.01750874
hsa-miR-1180-3p	-1.113218481	2.320411126	-2.278804976	0.080997658	-11.02133515
hsa-miR-378b	-1.271860039	3.699160247	-2.246940016	0.084007906	-11.06173158
hsa-miR-1972	-1.089730417	2.337734308	-2.210451559	0.087607745	-11.10804943
hsa-miR-4459	-12.04747893	17.61263984	-2.201728992	0.088493271	-11.11913004
hsa-miR-128-1-5p	-1.039824617	2.328149569	-2.154078257	0.093508018	-11.17970923
hsa-miR-1237-5p	-1.134691422	2.409096411	-2.13900335	0.095158823	-11.1988878
hsa-miR-1299	1.673350683	5.998179881	2.136457386	0.095440762	-11.20212734
hsa-miR-450a-5p	-1.278348167	2.979851356	-2.091323956	0.100593497	-11.25957481
hsa-miR-374a-5p	-4.091135425	5.822914799	-2.057435296	0.104661794	-11.30272263
hsa-miR-4489	-1.0722421	3.638283589	-2.043546694	0.106380488	-11.32040618
hsa-miR-122-5p	-1.068968772	2.359760264	-2.04214067	0.106556181	-11.32219634
hsa-miR-4745-5p	-1.4850441	5.379973283	-2.041643236	0.106618415	-11.32282967
hsa-miR-505-5p	-1.100862881	2.948720318	-2.015653201	0.109925447	-11.35591732
hsa-miR-6837-5p	-1.201963161	2.384091281	-1.999792636	0.111998003	-11.37610489
hsa-miR-3162-5p	1.529572433	14.27594134	1.981661545	0.114419074	-11.39917639
hsa-miR-6845-3p	-1.138672064	2.396086726	-1.922426777	0.122729437	-11.47448279
hsa-miR-3610	-1.2827429	5.554903556	-1.91698441	0.123524823	-11.4813948
hsa-miR-10a-3p	-1.251296283	2.434279231	-1.883559663	0.128532131	-11.52381208
hsa-miR-126-5p	-1.682893611	2.443331139	-1.865299298	0.13135869	-11.54695748
hsa-miR-6777-3p	-1.067819178	4.390580522	-1.854769719	0.133018562	-11.56029385
hsa-miR-379-5p	-1.225177617	3.015208492	-1.843843106	0.13476458	-11.57412465
hsa-miR-32-3p	-1.757429122	3.927906278	-1.736344348	0.153289788	-11.70962446

hsa-miR-6508-5p	-1.002711267	3.5252919	-1.718777424	0.156562423	-11.73164701
hsa-miR-6774-5p	-1.594938033	5.6418678	-1.705225568	0.159136738	-11.74860862
hsa-miR-769-5p	-1.03932995	3.033634064	-1.640570776	0.172036255	-11.82916236
hsa-miR-3120-3p	-1.798294083	3.663352353	-1.606728871	0.179212781	-11.87105183
hsa-miR-193a-3p	-1.549934411	3.407354261	-1.586902964	0.183558591	-11.89549382
hsa-miR-489-3p	-2.056781278	3.611053239	-1.531321554	0.196321657	-11.96358648
hsa-miR-1202	1.866189883	17.58024339	1.495825193	0.204937462	-12.00670749
hsa-miR-500a-3p	-1.589328222	3.659917583	-1.489357461	0.206547675	-12.01453122
hsa-miR-17-5p	-2.907048831	4.910890368	-1.448007195	0.217144453	-12.06429173
hsa-miR-136-3p	-1.209584278	3.166167211	-1.403092026	0.229265062	-12.11779697
hsa-miR-342-5p	-2.003067897	4.160990346	-1.354877049	0.243013866	-12.17453602
hsa-miR-29c-5p	-2.074076878	4.4129574	-1.308088276	0.257117553	-12.22883666
hsa-miR-224-5p	-2.021261167	4.045015606	-1.289228286	0.263021721	-12.25049649
hsa-miR-508-5p	1.828468589	5.090002594	1.143687394	0.313061066	-12.41261675
hsa-miR-24-1-5p	-1.014525606	2.876472814	-1.016050585	0.363916257	-12.54614835
hsa-miR-7-1-3p	-1.175208756	2.970157611	-1.006559584	0.367972258	-12.55570522

Supplementary Table 3. The gene expression matrix of mRNAs

id	logFC	AveExpr	t	P.Value	B
GATA6	1.903094593	3.340851891	7.969930474	2.35E-07	6.579997149
ZNF185	-1.469973082	3.331197489	-7.324262344	7.75E-07	5.608391396
AOX1	-1.2017309	3.645737962	-6.501876635	3.84E-06	4.268489614
LMO2	1.920074763	6.225314063	6.191701683	7.18E-06	3.733362727
IL12RB2	-0.930468359	2.76538608	-5.970447523	1.13E-05	3.341926735
MUC1	-0.732111776	3.167682214	-5.905143644	1.30E-05	3.224879216
ZNF680	0.851353904	6.970636241	5.886755619	1.35E-05	3.191798642
ASPH	1.212288754	5.717972044	5.654258938	2.19E-05	2.768959016
ENPP2	-1.518038678	6.879514277	-5.594358486	2.49E-05	2.658673045
FOXF2	-1.394098208	6.345102329	-5.560832119	2.67E-05	2.596710839
PLA2G4A	0.77664466	9.748431096	5.505099112	3.01E-05	2.493338484
IGFBP3	3.236412271	6.258409648	5.384045205	3.89E-05	2.267254314
DUSP16	-0.617759676	4.911885093	-5.353736226	4.15E-05	2.210321443
EPS8	0.662737053	11.69527957	5.281178903	4.85E-05	2.073509572
LOC100288911	-0.914019794	2.571436637	-5.240368711	5.30E-05	1.996242793
EMILIN1	1.581173198	6.549549436	5.225754502	5.47E-05	1.968518905
SLC19A3	-0.765771452	3.333502242	-5.21698268	5.58E-05	1.951864631
DMKN	-1.30106913	5.557724541	-5.197447968	5.82E-05	1.914739133
MGST1	1.362571367	10.61045628	5.174093496	6.12E-05	1.870288241
PLAG1	0.792315951	3.979603924	5.165705078	6.23E-05	1.854305031
SCGB2A2	-2.179377349	3.409877616	-5.133119733	6.69E-05	1.792130721
AHR	1.096324214	7.728881368	5.124946848	6.80E-05	1.776515058
ZCCHC3	-0.743173968	5.861091428	-5.016796319	8.61E-05	1.569084653
HYAL1	-1.635539649	4.514924363	-4.970820876	9.52E-05	1.480471274
VAMP8	-1.572016478	5.804236351	-4.954329312	9.87E-05	1.448624205
PTRHD1	-0.898576282	7.075959654	-4.941475965	0.000101474	1.423780883
SGCE	-1.195489241	7.689809927	-4.91252922	0.000108109	1.36776183
ENPP4	0.69054358	2.490025318	4.903102576	0.000110364	1.349498266
MT1G	-1.937295532	10.67090267	-4.892632831	0.000112925	1.329201958
CYP1B1	1.515119454	11.21241374	4.874839444	0.000117416	1.294680024
C6orf52	-0.731391736	2.896657937	-4.845026068	0.000125354	1.236758717
FAM43B	-0.900995896	3.648344225	-4.827695575	0.000130217	1.203044441
TEX14	-1.189145873	3.098585964	-4.761986205	0.000150467	1.074924775
ZMAT3	0.724865542	5.449903817	4.75246771	0.000153656	1.056328437
CCT6B	-0.97279088	3.69385396	-4.715246299	0.000166797	0.983520894
B3GNT7	0.631789417	6.260100602	4.709801379	0.000168813	0.972858705
CYB5R2	-0.988509054	3.859760161	-4.66457853	0.000186543	0.884192538
TMEM27	-1.62963952	5.171874434	-4.66397295	0.000186793	0.883003881
OSCP1	-0.786596915	4.618541725	-4.635591164	0.000198891	0.827256512
CYB5D2	-0.767571088	5.01027136	-4.608731891	0.000211073	0.774431557
SMIM3	2.303470985	5.115883098	4.604924017	0.00021286	0.766937238
PSME1	0.876930278	5.051976138	4.603264431	0.000213644	0.763670584
ZNF260	0.850730936	5.117638999	4.570889355	0.000229532	0.699896554
SERTAD4-AS1	-0.809361466	5.469621265	-4.568500114	0.000230751	0.695186509
ITM2C	-0.83816313	8.393561115	-4.551637327	0.000239543	0.661930117
ATAD2	0.620744405	5.660868435	4.545158996	0.000243011	0.649147334
CLDN11	-0.667447116	2.846593544	-4.529124078	0.000251813	0.617492888
NMRAL1	0.903491496	4.34206308	4.525061117	0.000254095	0.609468863
DLX3	1.147831995	2.921977177	4.505410738	0.000265427	0.570642089
GSTZ1	-0.746465967	4.08769536	-4.495700833	0.000271215	0.551445121
TFPI	2.055735683	6.303031304	4.487722459	0.000276065	0.535665935

RCL1	-0.86389911	6.802186619	-4.486251099	0.00027697	0.53275542
C4BPA	-0.710256809	2.555553045	-4.484185287	0.000278244	0.528668728
SULF1	1.096191063	3.357431949	4.479494172	0.00028116	0.519387307
HAS2	-1.435044745	5.476003776	-4.455146309	0.000296801	0.471187882
TMEM123	0.622984972	9.273380927	4.452490578	0.000298559	0.46592786
EMILIN3	-0.716321298	3.691391593	-4.448947117	0.000300922	0.458908762
RGS3	-0.862447377	5.780314293	-4.433957873	0.000311127	0.429206998
PXDN	1.143151734	7.285699592	4.431008541	0.000313176	0.423360863
LAMA5	0.638495968	3.037974256	4.429122637	0.000314493	0.419622317
DMXL2	1.210445757	6.394658425	4.381727324	0.000349503	0.325585864
EDEM3	0.695535825	7.333429143	4.368653221	0.000359837	0.299618915
PRSS16	-0.720213168	2.612179112	-4.367006077	0.000361161	0.296346669
CLIP4	0.609162154	6.330061417	4.359944712	0.000366891	0.282316443
FOXQ1	-1.382805735	3.795466383	-4.356488882	0.000369729	0.275448888
LGALSL	-0.830803009	8.646890843	-4.355952496	0.000370171	0.274382893
SMKR1	-0.707675957	2.931565755	-4.338171581	0.000385144	0.239035461
CRYL1	-0.943154181	4.342029627	-4.331641272	0.000390796	0.226048654
GSTO2	-1.275553892	5.069111616	-4.32599555	0.000395749	0.21481893
OARD1	-0.61029659	7.488903295	-4.308913292	0.000411126	0.180829539
LDLR	-1.601830469	6.498533763	-4.308726774	0.000411297	0.18045832
FOXN3-AS1	-0.620650533	3.604855852	-4.306455901	0.000413387	0.175938536
PMVK	-0.751117013	4.75560239	-4.28295039	0.00043566	0.12913751
MMD	0.986515093	6.773428182	4.282127129	0.000436462	0.127497781
KANK2	0.787309607	6.435535046	4.279688375	0.000438845	0.122640174
LINC00467	-1.123047279	3.987824244	-4.268638975	0.00044981	0.100627479
SOWAHC	-0.733746155	6.721463715	-4.263125213	0.000455384	0.089640465
PALLD	-1.196309679	8.968735741	-4.22952349	0.000490895	0.022649854
PROK2	-0.831881703	2.931377475	-4.208907667	0.00051405	-0.018478814
TALDO1	0.727592381	11.56850607	4.208860129	0.000514104	-0.018573675
MB21D2	-0.664049048	5.443786023	-4.191049183	0.000534996	-0.054122524
QDPR	-1.111407796	5.629826859	-4.132101382	0.000610431	-0.17187214
SERPINA1	-1.037830855	10.54819322	-4.12069571	0.000626219	-0.194670704
HHLA3	-0.805205187	3.281369075	-4.115504119	0.000633541	-0.205049586
TOX	-0.619500885	3.033791587	-4.106342543	0.000646672	-0.223367397
SCGB1D2	-2.249564886	4.040611384	-4.101678474	0.000653461	-0.232693887
PQLC3	-1.120211688	7.284127016	-4.095615855	0.000662394	-0.244818037
HIPK2	0.940516225	5.904706979	4.083653785	0.000680381	-0.268743412
HOXA7	0.751639757	5.669707464	4.078520733	0.000688249	-0.279011376
ZFPM2	-1.118446022	2.833526222	-4.071296002	0.000699478	-0.29346476
CCND1	1.484930221	5.383347417	4.068437932	0.000703971	-0.299182864
MT1F	-1.389473761	9.445713973	-4.065228443	0.000709051	-0.305604316
PXDC1	-0.661975445	9.313190931	-4.063751905	0.0007114	-0.308558626
APOL3	1.178278516	3.981459552	4.056936279	0.000722346	-0.322196339
NRXN3	-0.668143808	2.375468624	-4.039631881	0.000750904	-0.356826936
PSMB10	0.643442806	4.289046688	4.037393705	0.000754679	-0.361306645
HEXA	-0.762568223	8.093617119	-4.030524564	0.000766386	-0.375055965
BCKDHB	-0.657115631	3.814088624	-4.021707161	0.000781681	-0.392706501
NSUN7	-0.633217259	2.333650758	-4.018922376	0.000786575	-0.398281388
ELL2	0.703455087	7.88099808	3.987515641	0.000843942	-0.461165378
GSTA4	-0.836004034	8.400376547	-3.9778359	0.000862455	-0.480550082
PPIA	-1.074146726	6.903511656	-3.971539253	0.000874715	-0.493160548
IBSP	-2.414626547	5.037774163	-3.963653638	0.000890317	-0.508954072
LGALS8	0.72962475	5.460544746	3.959743456	0.000898156	-0.516785793

KIAA1804	-1.162453439	4.115684986	-3.939679259	0.000939482	-0.556975262
CFL2	-0.810621109	7.818416262	-3.939296668	0.000940288	-0.557741649
PER2	-0.655290065	7.10895946	-3.932625127	0.000954459	-0.571105962
LYVE1	-1.50362957	5.520369528	-3.926259744	0.000968179	-0.583857331
SLC40A1	1.240892822	9.247796652	3.91578337	0.000991192	-0.604844624
PRKCH	-0.817705032	4.5596521	-3.913998342	0.000995167	-0.608420633
TMTC1	1.184674002	4.797732684	3.892650237	0.001043964	-0.65118917
C1orf94	-0.710933189	3.647451483	-3.89043405	0.001049165	-0.655629138
PIR	0.902292136	7.878022385	3.888342103	0.001054098	-0.659820207
MPV17	-0.836499594	5.310620861	-3.887495319	0.001056101	-0.661516683
BEX1	-1.0963424	3.272515718	-3.885943691	0.001059782	-0.664625267
LOC100505501	-0.757881875	2.641498149	-3.879621543	0.001074913	-0.677291298
TRAM2	0.733661681	6.381830081	3.873947794	0.001088676	-0.688658312
PHF11	-0.705403662	9.59360558	-3.873598771	0.001089528	-0.689357558
MAP3K8	1.091357104	7.768777897	3.862666941	0.001116566	-0.711258728
KLHDC9	-0.611866939	3.028644284	-3.858276357	0.001127613	-0.720054889
CADPS2	0.948045077	3.873103292	3.847888493	0.001154187	-0.740865829
MAN2B2	-0.759883845	7.978233739	-3.838498489	0.001178746	-0.759677226
ASB13	0.604637553	4.546233154	3.828960709	0.001204226	-0.7787841
INPP1	-0.60237031	8.680361305	-3.824892103	0.001215262	-0.78693446
ACTC1	-1.459980616	5.191829927	-3.81170525	0.001251729	-0.813349763
SLC44A3	-0.696341752	2.493332347	-3.805280062	0.001269891	-0.826219759
MAGI2	-0.671960367	4.250458518	-3.804763871	0.001271362	-0.827253697
CHMP4C	-1.713229267	6.48442426	-3.800907196	0.001282403	-0.834978572
ZEB2	1.650922664	3.328605033	3.794310981	0.001301508	-0.84819028
CPAMD8	-1.05330911	4.086387208	-3.792132119	0.001307881	-0.852554246
FBLN7	-0.946794383	4.211085262	-3.790923783	0.001311429	-0.854974351
INPP4B	1.065970152	5.017798055	3.775771937	0.001356739	-0.885319254
PRKCZ	-0.840429017	8.846121142	-3.771964463	0.001368369	-0.892943984
SCRN1	-1.123175195	5.226131399	-3.754975126	0.001421483	-0.926963131
ARMC9	0.733791378	4.615177651	3.750573483	0.001435576	-0.935775991
ZFP36L1	1.528745549	6.237822506	3.740984238	0.001466763	-0.954973924
NUDT11	-1.007698068	4.979426127	-3.724977709	0.001520331	-0.987014763
ARHGAP5-AS1	-0.997366442	5.476551818	-3.714774692	0.00155549	-1.007435182
GMPR	-0.915642413	3.742683462	-3.713764248	0.001559015	-1.009457344
IDI1	-0.796638244	10.02945216	-3.702899803	0.00159743	-1.031198155
ACAN	-0.952948861	11.95187531	-3.691728793	0.00163791	-1.053548855
USE1	-0.663816157	7.337341261	-3.689707157	0.001645345	-1.057593293
SERPINA5	-1.088421812	9.500406385	-3.688916927	0.00164826	-1.059174173
SDR16C5	-1.661894689	2.958608809	-3.682555512	0.001671914	-1.071899665
FAT4	1.446208783	5.739799555	3.676853567	0.001693403	-1.083304819
PLP2	-0.642081735	9.360060615	-3.659516243	0.001760444	-1.117976461
C4orf32	-1.15013865	5.164801307	-3.658906589	0.001762849	-1.119195467
C2orf42	-0.636351714	5.395077878	-3.648546585	0.001804219	-1.13990823
PTGDS	1.222862019	3.660024227	3.64613461	0.001813988	-1.144729905
WSCD2	-0.79566772	3.251190349	-3.62396658	0.001906272	-1.18903406
RAB3B	-0.846355282	2.217928685	-3.6211663	0.001918257	-1.194629122
PRPH2	-0.690719331	3.209676541	-3.615838784	0.001941264	-1.205272743
SMYD3	-1.029804487	4.146429416	-3.61378962	0.001950186	-1.209366344
AKR1C3	2.304257176	5.640682569	3.600760863	0.002007873	-1.235389322
C9orf69	0.663879311	5.301337275	3.596857092	0.002025486	-1.243184966
CXXC5	0.643056312	8.637667515	3.572005	0.002141258	-1.292795705
RBM20	1.091198716	3.354748787	3.568235649	0.002159381	-1.300317455

GPRC5A	-0.922428662	10.01075098	-3.568114097	0.002159968	-1.300559998
CSRNP1	-0.730468473	7.337897945	-3.565950365	0.002170443	-1.304877374
CDCA7L	-0.904035756	6.13026015	-3.565412504	0.002173054	-1.305950547
SMYD2	-0.618818952	6.967317007	-3.563145774	0.002184095	-1.3104731
DPAGT1	0.990600266	4.198789278	3.562487407	0.002187312	-1.311786613
STK38L	0.665955596	8.432575353	3.559835376	0.002200319	-1.317077462
S100B	-0.707299106	8.360231181	-3.555787707	0.002220319	-1.325151882
SCGB2A1	-0.630457334	2.026928952	-3.545114819	0.002273922	-1.346438073
TAF13	1.061927181	6.026862185	3.539689039	0.002301662	-1.357256824
ELMO1	0.877572592	3.098668931	3.534546663	0.002328262	-1.367508873
ZBED5-AS1	-0.716471577	3.071604035	-3.529539594	0.002354455	-1.377489645
HOXB7	0.675396675	5.649150745	3.522812448	0.002390106	-1.390896693
TRAM2-AS1	-0.63634862	5.532961887	-3.513896689	0.00243818	-1.408661239
PTPRM	0.659943795	8.49253701	3.510837678	0.002454894	-1.414755117
CENPM	-1.140325337	3.485422656	-3.506378197	0.002479463	-1.423637793
ALKBH3	-0.625434855	7.017850927	-3.506068197	0.00248118	-1.424255223
FGFBP1	-0.662230533	2.664055376	-3.497584481	0.002528627	-1.441149836
KCTD4	-0.946390999	2.752047063	-3.485925349	0.002595299	-1.464360096
SLC16A6	1.239865427	2.633485903	3.484596195	0.00260301	-1.467005498
TM4SF1	-0.822867833	11.2660856	-3.482720923	0.002613926	-1.470737621
CBX6	0.62681379	7.031852205	3.476651785	0.002649569	-1.482814576
BID	0.870837575	2.62537771	3.467606323	0.002703584	-1.50080921
TRIM29	-1.333900743	6.463062935	-3.462518125	0.002734445	-1.510928815
CRIP2	-0.784597802	4.88003918	-3.458920389	0.002756476	-1.518082972
GLRX	0.971094881	7.603206253	3.453810979	0.002788065	-1.528241444
DCXR	-1.2723576	6.037859998	-3.451813197	0.002800514	-1.532212875
THEMIS2	0.916298097	2.625909443	3.447554733	0.002827232	-1.540677338
SHISA2	-1.485773996	5.451235636	-3.445357866	0.002841114	-1.545043458
EFNB2	-0.987226688	3.452939343	-3.444516024	0.002846451	-1.546716459
TTLL7	-0.627080112	3.753887774	-3.443204116	0.002854789	-1.54932352
LAG3	-1.033518393	4.427973857	-3.442594496	0.002858671	-1.550534929
FLJ20021	-0.768795905	6.819656742	-3.428221822	0.002951731	-1.579087092
SUMF1	-0.852445062	7.095766298	-3.426149912	0.002965391	-1.583201693
PEX13	0.78040727	6.278725927	3.416075057	0.003032705	-1.603204281
C4orf48	0.872314356	4.562804121	3.412960746	0.003053817	-1.609385713
ABLIM1	-1.378942766	4.873276625	-3.406987185	0.003094717	-1.621240021
MAP4K4	0.641784236	6.304198949	3.406972239	0.00309482	-1.621269677
NAPRT	0.697342702	3.29936969	3.399335302	0.003147899	-1.636420421
MTMR11	-0.649557195	3.777747502	-3.39200567	0.003199686	-1.650956721
ZNF420	0.789362508	4.883756725	3.388961584	0.00322144	-1.656992425
UBLCP1	0.679289747	7.053941648	3.38771661	0.003230379	-1.659460676
SLC25A45	-0.704061215	3.60679391	-3.384742137	0.003251834	-1.66535722
SLITRK4	1.151402046	6.209819448	3.380983496	0.003279147	-1.672807142
JPH1	-0.763033078	4.113861487	-3.379275435	0.003291633	-1.676192226
SPAG4	-0.827553261	3.891579485	-3.374944039	0.003323508	-1.684775113
NQO1	1.881865811	8.195438003	3.37463865	0.003325767	-1.685380194
ZNF365	-0.600825651	3.349608683	-3.371230969	0.003351074	-1.692131376
PLCG2	-0.964075341	4.074544129	-3.360536798	0.003431733	-1.713311236
MAOB	-1.021982737	6.506881474	-3.359282739	0.003441316	-1.715794194
SERPINI1	-0.977408927	8.619239878	-3.358146615	0.00345002	-1.718043519
NUDT12	0.87033414	4.959068349	3.347910281	0.00352943	-1.738304025
BTN3A2	0.623124059	3.65656398	3.338681583	0.003602562	-1.756561324
UACA	0.717914029	4.584353359	3.335133179	0.003631077	-1.763578931

LOC100129550	-0.789619326	6.408226406	-3.330665138	0.003667297	-1.772413476
COL9A3	-0.944523369	10.64463918	-3.329720838	0.003674998	-1.774280356
DACT1	-0.661516584	3.061756601	-3.324684661	0.003716335	-1.784235332
AHCY	-0.778504421	6.468162552	-3.323367278	0.003727224	-1.786838961
ATP6V1E2	-0.632571523	3.302376026	-3.319824477	0.003756661	-1.793839937
LNP1	-0.923126589	6.107077489	-3.319764096	0.003757165	-1.793959246
EBF1	1.498717114	4.643660186	3.319596565	0.003758563	-1.794290272
CKB	-1.252384516	7.147777472	-3.31686431	0.003781433	-1.79968856
ADIRF-AS1	-0.960721619	3.222144899	-3.313359033	0.003810974	-1.806613004
MX1	-0.603691626	2.717125987	-3.309664613	0.003842355	-1.813909681
MAP3K7CL	-0.738957457	3.930045471	-3.299235849	0.003932314	-1.834499162
TXLNB	-0.605436243	2.577166172	-3.28918271	0.004020991	-1.854335837
GDF15	1.548320939	8.476730381	3.285824864	0.004051045	-1.860958988
ABCC1	0.603936018	8.522166932	3.284709721	0.004061075	-1.863158262
PHLPP2	-0.621141525	5.613753904	-3.275774282	0.004142326	-1.880775588
SCAMP1-AS1	-0.658298578	4.226448703	-3.267161859	0.00422215	-1.897747424
SOAT1	0.89939503	6.732318871	3.260746212	0.004282592	-1.910384655
CCPG1	0.744404693	9.291911507	3.259671702	0.004292797	-1.912500702
CCNB1IP1	-0.65121661	9.983941347	-3.259435731	0.004295042	-1.912965386
ZNF124	-0.771012784	5.986489127	-3.251441037	0.004371767	-1.92870493
ZBTB41	0.662206266	7.551148689	3.246919102	0.004415757	-1.937604105
KYNU	0.740482777	2.591201334	3.241943314	0.004464665	-1.947393612
RPTN	-0.69450586	2.100161918	-3.239101267	0.004492837	-1.952983786
IP6K3	0.709917148	3.316331541	3.22720873	0.004612626	-1.976365059
CHST1	-0.78182034	8.511758003	-3.219068853	0.004696415	-1.992358249
SYBU	-0.75466722	7.878398385	-3.21301901	0.004759653	-2.004239505
POPDC3	0.922691257	8.35922584	3.211165329	0.004779195	-2.007879003
SLC2A12	0.977291047	4.182860088	3.205907606	0.004835054	-2.018199541
UST	0.724796404	4.27248804	3.204497421	0.004850144	-2.020967023
RNF19B	-0.72745751	8.595037322	-3.198361397	0.004916344	-2.033005922
GGTA1P	-0.949566838	4.597427089	-3.181304248	0.005105047	-2.06644585
SPIDR	0.742056723	4.826679924	3.169700217	0.005237452	-2.089172613
LINC00545	-0.91471143	3.455545184	-3.169154718	0.005243758	-2.090240529
MMP1	2.082072425	3.539864452	3.15596188	0.005398552	-2.116055308
C5	-0.715278617	4.46860192	-3.154083143	0.005420956	-2.119729488
SRPR	0.637846152	7.522281695	3.14461837	0.00553521	-2.138231733
BFSP1	-0.886293217	3.764645996	-3.143548594	0.005548271	-2.140322178
TMEM17	-0.722076361	4.363559945	-3.142039861	0.005566742	-2.143270106
THNSL2	-0.751542761	5.114755765	-3.138110204	0.005615135	-2.150946743
SLC43A3	0.601850157	4.601715048	3.13643798	0.005635852	-2.154212772
PRTFDC1	-0.915099735	4.94748922	-3.132414432	0.005686006	-2.162069501
LOC100132891	0.803824275	2.374992791	3.12733081	0.005749997	-2.171992803
PPP2R2B	-1.00327367	4.611766247	-3.126496962	0.00576056	-2.173620122
SPR	0.604355346	4.713239903	3.126295654	0.005763113	-2.174012974
SYTL2	-0.727154728	3.213271417	-3.12509835	0.00577832	-2.176349387
TDRD6	0.643461602	3.360950484	3.122260552	0.005814519	-2.181886192
C1GALT1C1	0.837475995	6.242703535	3.121214574	0.005827918	-2.183926689
ALAS1	0.696407104	7.445336037	3.117994802	0.00586935	-2.190206793
VWDE	-0.757385573	2.15234636	-3.10983288	0.005975671	-2.206119431
LOC440028	-1.161364513	3.323256581	-3.105137547	0.006037683	-2.215268933
MAMLD1	-0.729747245	2.673678469	-3.102225185	0.006076461	-2.220942364
HSD11B1	1.48908376	3.700757865	3.101023057	0.006092538	-2.223283789
DSE	0.79732973	10.2416086	3.100694533	0.006096939	-2.223923627

RPN1	0.619627856	10.26000839	3.084347433	0.006319878	-2.255740134
LOC100505841	-0.95550577	3.470656207	-3.08341812	0.006332788	-2.257547597
NEDD4	0.723417456	5.658467138	3.078455932	0.006402156	-2.267196438
PAAF1	-0.610626022	6.449310884	-3.076254746	0.006433164	-2.27147532
SELENBP1	-1.029867981	3.920620668	-3.075788007	0.006439757	-2.272382513
CHCHD1	0.624832811	8.059609859	3.075677818	0.006441315	-2.27259668
VRK1	-0.639764987	6.689823324	-3.07012929	0.006520223	-2.283378463
FAM189A2	-0.935628222	3.459941457	-3.06690153	0.006566558	-2.289648275
CAB39L	-0.818915686	6.403934471	-3.065822669	0.006582117	-2.291743545
FNDC4	-0.794548817	4.404680721	-3.062103661	0.006636025	-2.298964816
IRX3	1.533096142	5.343164189	3.05641481	0.00671932	-2.310006555
MYOT	-1.091409078	4.705620441	-3.053817273	0.006757689	-2.315046443
ZNF135	0.805848404	3.645297385	3.053454337	0.006763067	-2.315750541
THAP2	0.982142682	4.29277674	3.053326046	0.006764969	-2.315999422
COL11A2	-1.323588541	7.128506568	-3.047043329	0.006858756	-2.32818432
YAE1D1	-0.664354948	6.792051668	-3.045935204	0.006875428	-2.330332764
EYA4	0.631531725	2.800199368	3.045046972	0.006888821	-2.332054727
ATF3	-0.745762403	4.800462716	-3.042746894	0.006923618	-2.336513134
C7orf55	-0.747318184	4.633592389	-3.04094845	0.006950946	-2.33999856
IRX5	1.229607855	6.271473006	3.032728725	0.007077185	-2.355921558
AGT	-1.155619629	7.089124996	-3.032165488	0.007085916	-2.357012219
TBC1D4	-0.682413288	4.786653298	-3.028268643	0.007146612	-2.364556631
TSPAN1	-0.765351489	3.553136407	-3.026388546	0.007176076	-2.368195614
CLIC2	0.829862911	1.985534571	3.022677949	0.007234574	-2.375375785
SERPINB2	-0.714041626	2.475431272	-3.018993727	0.007293114	-2.382502536
PTPLAD2	-0.8587409	3.614889153	-3.013322562	0.007384125	-2.393468161
TLR1	0.727430762	3.674691409	3.001765772	0.007573014	-2.415796399
PELI1	-0.660040213	10.73661497	-2.99751056	0.007643737	-2.424011622
CD1D	1.52116075	4.544262427	2.99728311	0.007647535	-2.424450649
SAMD9	0.704304979	2.789411926	2.996204186	0.007665577	-2.426533083
IGFBP1	2.647170398	4.272732721	2.99379623	0.007705993	-2.431179921
MGAT4B	0.621547468	7.627927321	2.977635219	0.007982641	-2.462339691
LIFR	0.841809182	6.188001502	2.972655536	0.008069807	-2.471931175
TNFAIP6	1.376896967	9.096467527	2.972381007	0.00807464	-2.472459816
SEMA3E	1.104952489	7.324327478	2.964811525	0.008208985	-2.487030256
LY75	-1.165628423	3.35271454	-2.961834433	0.008262415	-2.492757865
IRF1	0.720661263	4.972827435	2.949422276	0.008488824	-2.51661927
TMEM71	-1.082129159	5.147770292	-2.949140819	0.008494027	-2.517160006
AGA	-0.838661135	5.717320682	-2.942258966	0.00862221	-2.530376625
CDS1	-0.746298557	4.000046707	-2.938237117	0.008697986	-2.538096311
COL8A2	0.709129262	5.306878631	2.934148152	0.008775686	-2.545941564
CDNF	-0.604508158	3.032433167	-2.933650375	0.008785191	-2.546896393
SPG20	0.666221242	8.362890468	2.930056907	0.008854102	-2.55378788
MCM2	-0.728844283	4.539784535	-2.928140928	0.008891057	-2.557461259
LIAS	-0.756713783	5.583693431	-2.928056896	0.008892681	-2.557622351
COL6A2	-1.093370373	7.042299064	-2.924176918	0.008967989	-2.565058868
RUNX1-IT1	0.837427505	3.303534135	2.915304752	0.009142514	-2.58205224
ARG2	-0.916475243	6.038988003	-2.914193176	0.00916461	-2.584180185
ADTRP	-1.166435665	5.793621094	-2.91141303	0.009220099	-2.589501256
LINC00936	-1.036675771	5.071345185	-2.910917197	0.00923003	-2.590450091
RORC	-0.633759942	2.82976178	-2.910821651	0.009231945	-2.590632925
ZEB1-AS1	-0.730968955	6.364071211	-2.903920895	0.009371257	-2.603832991
TUBA4A	0.681927391	7.412539034	2.903884065	0.009372006	-2.603903416

SFRP2	0.790524941	2.503760536	2.90204535	0.00940947	-2.607418924
S100A3	-1.021420408	3.715356777	-2.896441566	0.009524541	-2.618128704
CCDC91	0.639555781	8.277661094	2.894420919	0.009566366	-2.621988903
MYH10	-0.661169034	6.625798327	-2.893250532	0.009590673	-2.624224396
MFAP4	1.043602922	3.041192889	2.892465766	0.009607005	-2.625723173
ARHGDIB	0.625916397	3.427256803	2.890710138	0.009643638	-2.629075679
CLEC3A	-1.708521224	6.906481576	-2.883709786	0.009791052	-2.642436947
RBPMS	0.622012453	3.568750982	2.881656235	0.009834707	-2.646354511
FRMD4B	0.679216271	8.484521586	2.875288263	0.009971275	-2.658497014
CASP4	0.757790126	4.795051744	2.873471841	0.010010564	-2.661958997
GREM1	1.224310377	8.941100263	2.872238257	0.010037331	-2.664309726
RAB31	0.85367014	8.094756785	2.869359017	0.010100076	-2.669795162
MRAP2	-0.778228497	3.258449388	-2.864941164	0.010197086	-2.678208442
SLC6A12	-0.906169909	3.848409953	-2.860583529	0.010293653	-2.686502903
CLCF1	-0.880415304	6.488410856	-2.859394373	0.010320157	-2.688765665
DKK1	-1.142102848	3.852638831	-2.856847308	0.01037715	-2.693611257
PTGES	0.842145296	4.080253811	2.854941309	0.010419996	-2.697236343
FAM46B	-0.756798409	3.530223533	-2.851588483	0.010495779	-2.703611268
GALNT7	0.666066728	7.82476127	2.847863434	0.010580599	-2.710691019
LRP8	0.623427123	4.392763687	2.847070493	0.010598739	-2.712197671
DEFB1	1.395979913	9.277511895	2.842894717	0.010694764	-2.720129681
ST6GALNAC2	1.028035475	4.805333874	2.838262452	0.010802266	-2.728924265
LOC100289098	-0.959489017	4.626879836	-2.836067817	0.010853559	-2.733089214
GPR126	-1.15647527	3.926504346	-2.832348233	0.01094103	-2.740145719
TMEM56	-0.676537449	2.631560528	-2.822920935	0.011165771	-2.758016454
LOC93622	-0.688138138	7.035725417	-2.822212976	0.011182826	-2.759357671
FGF1	-0.797535354	3.022585756	-2.81991079	0.011238461	-2.763718337
MGAT1	0.761081167	7.684046923	2.805791162	0.011585539	-2.790436244
HIST1H4H	-1.339850798	5.023922142	-2.798846342	0.011760006	-2.803560617
BAG2	0.673915949	6.7212887	2.797755073	0.01178765	-2.805621879
CNIH3	1.001700646	4.990434765	2.796071729	0.011830413	-2.808800941
LPXN	-0.760503889	4.954604036	-2.795969848	0.011833006	-2.808993325
DET1	-0.654607415	5.195660895	-2.792638407	0.011918094	-2.815282835
LECT1	-1.827436654	9.201031944	-2.792257636	0.011927857	-2.816001535
TNFRSF12A	-0.762858872	8.72459529	-2.791870729	0.011937785	-2.816731782
PDLIM1	-1.278680771	4.631781509	-2.791788034	0.011939908	-2.816887855
CRISPLD2	-1.233301817	7.976496818	-2.788100946	0.012034931	-2.823844994
GLA	0.888278748	8.390742012	2.787788294	0.012043022	-2.824434787
LOC100130992	-0.73160574	3.87624378	-2.781995438	0.012193878	-2.835358317
GSR	0.75030522	5.862921101	2.776211517	0.012346309	-2.846256977
VPS13A	0.697001146	4.475970019	2.774523765	0.012391132	-2.849435695
FOXP1-IT1	0.653582829	5.69320112	2.768080649	0.012563685	-2.86156434
PTMS	-0.637334492	4.814432751	-2.760052727	0.012781904	-2.876662097
TPPP3	-0.910285175	3.714823913	-2.757375717	0.012855475	-2.881693117
MATN1-AS1	-0.732229137	4.75161769	-2.755451483	0.012908609	-2.885308321
COL9A2	-1.157207849	6.591245491	-2.754975567	0.012921782	-2.88620232
ZNF474	-0.797052649	3.447326167	-2.747630412	0.013126743	-2.89999292
SH3TC2	-0.637961388	2.999825594	-2.739822842	0.013348016	-2.914636955
AGAP1	-0.67281462	5.053700277	-2.736997595	0.013428962	-2.919932273
ITGB8	0.68601229	4.266559282	2.736041703	0.013456456	-2.921723431
IER5L	0.701921879	6.01726206	2.734038281	0.013514253	-2.92547671
CDKN3	-0.741015055	4.397913605	-2.733856744	0.013519502	-2.925816756
MT4	-0.66036999	8.811256773	-2.731785711	0.013579521	-2.929695536

IGFBP5	0.620333841	3.273211817	2.73073199	0.013610156	-2.931668602
ABHD4	0.657860778	4.271914632	2.722814144	0.013842476	-2.946485518
CAMTA2	-0.663007565	4.970172326	-2.72176712	0.01387348	-2.948443647
HOPX	-1.531716454	2.989948635	-2.717554233	0.013998899	-2.956319673
TIAM1	-0.811768901	4.378384763	-2.715781346	0.014052002	-2.959632733
TTC13	0.689068489	4.97037834	2.712801569	0.014141688	-2.965199319
CHST10	-1.103610816	6.571357852	-2.712614706	0.01414733	-2.965548325
DUSP10	0.702061137	5.413983771	2.711262329	0.01418823	-2.968073905
SOX9-AS1	-0.817548137	9.254661568	-2.710553315	0.014209718	-2.969397807
RAB11FIP1	-0.757419279	3.860422617	-2.705303445	0.014369794	-2.979196518
ALDH3A2	1.041097534	7.428783591	2.702195838	0.014465358	-2.984993371
HIST1H2AE	-1.478890701	3.691297953	-2.696881411	0.014630194	-2.994900889
STK19	-0.718730111	5.338177367	-2.69506768	0.014686859	-2.998280466
SPATA7	-0.832271709	5.071590014	-2.694974854	0.014689765	-2.998453408
IFNAR1	0.608943478	3.74226408	2.692883343	0.014755381	-3.002349447
RAPGEF5	-1.172797805	6.358016776	-2.692168369	0.014777875	-3.003681025
ZBED9	-0.940872631	3.434692516	-2.690933511	0.014816803	-3.005980523
TTLL4	0.60291541	4.48470535	2.681583629	0.015114743	-3.023378267
FLNC	0.861158166	3.947421776	2.676341244	0.015284285	-3.033122749
CYFIP2	0.626737244	3.280275814	2.668101107	0.015554444	-3.04842439
NT5DC3	-0.81839505	4.700437865	-2.66677797	0.015598246	-3.050879688
NOVA1	0.762176079	8.792959438	2.666290406	0.015614416	-3.051784322
PDGFRB	0.974676826	3.977907331	2.655166506	0.015987707	-3.072406091
PCK2	-0.609387889	7.549826921	-2.651311584	0.016119039	-3.079544483
CHUK	0.68211056	6.495209977	2.642568055	0.016420735	-3.095720087
LDOC1	-0.608976703	6.829587615	-2.639538766	0.016526507	-3.101319311
ADNP2	-0.824169954	6.998263308	-2.636929762	0.016618123	-3.10613963
PPP1R15A	-0.809298205	8.045945148	-2.630838171	0.016833916	-3.117386792
MVP	0.630117677	5.092293079	2.630735897	0.016837561	-3.117575535
IFIT3	1.224422011	6.912638555	2.628401776	0.016920967	-3.121882267
DNAJC12	0.874576962	5.431019937	2.619528754	0.017241626	-3.138239878
LPL	-0.978083949	6.412331484	-2.615809832	0.017377731	-3.145089081
DYNLRB2	-0.636937905	3.896131692	-2.610126366	0.017587707	-3.155548712
SPTSSB	-1.26286057	4.778213976	-2.60676585	0.017712991	-3.161728872
CAPG	-0.656010351	6.21717943	-2.606459078	0.01772447	-3.162292878
ZNF367	-0.722179422	5.20621653	-2.602565552	0.017870775	-3.169448814
NR4A3	-0.782194416	4.142330089	-2.591604959	0.018288809	-3.189569503
IFNAR2	0.64268632	6.934409028	2.58672675	0.018477827	-3.198513181
IL17RD	-0.754909315	3.431781744	-2.583349359	0.018609776	-3.204701128
SLC25A33	-0.630471325	8.396677557	-2.583064865	0.018620931	-3.205222214
TOLLIP-AS1	-0.641235097	3.214765146	-2.578804769	0.018788734	-3.213022213
ZNF204P	-0.72968916	3.338433344	-2.576206277	0.018891788	-3.21777724
MAN1A1	1.277828753	5.022894089	2.573818936	0.018986941	-3.22214409
MYD88	0.603613138	5.123652749	2.568024843	0.019219766	-3.232735324
ADH1B	0.925103006	2.05486445	2.565911497	0.019305357	-3.236595859
GPR137B	0.63935783	8.334779742	2.561501685	0.019485118	-3.244647072
SSPN	-0.605219542	6.406272727	-2.557689097	0.01964181	-3.251603128
CD80	0.848999477	3.40375302	2.557186799	0.019662542	-3.252519238
MRPL34	0.645785075	6.345029036	2.555990337	0.019712009	-3.254701079
NUDT9P1	-0.697850007	3.454489114	-2.555202402	0.01974465	-3.2561377
ACTR1A	0.625753741	5.513426905	2.547613689	0.020061646	-3.269964226
NIPSNAP3B	-0.833296142	4.142700462	-2.546269061	0.020118313	-3.272412266
MT1M	-1.212321266	8.214867469	-2.545514517	0.020150178	-3.27378575

PNPLA4	-0.692569932	4.664336686	-2.545043908	0.020170077	-3.274642302
ZMYND12	-0.770229474	3.059192909	-2.542734069	0.020268011	-3.278845428
TCP11L2	0.611949588	3.349496897	2.542072578	0.020296141	-3.280048813
CXCL8	1.883541952	5.631364715	2.541545377	0.020318586	-3.2810078
ILF3-AS1	-0.770241378	8.056315848	-2.53087641	0.020777873	-3.300396186
S100A2	-1.563529121	10.67226982	-2.530461753	0.02079592	-3.301149009
TLR2	1.049678477	3.800613384	2.525448594	0.021015278	-3.310246281
MSMO1	-0.765032231	11.11390671	-2.518619869	0.021317592	-3.322625438
SOCS2	-0.994970431	4.876902117	-2.517487809	0.021368104	-3.324676214
GBP1	1.03638574	4.763902126	2.507994338	0.021796161	-3.341857932
CDH11	1.113361817	4.572775378	2.505315541	0.0219184	-3.346700912
SALL3	-0.747463015	2.415252279	-2.503083518	0.022020746	-3.350734407
LIMA1	0.708474823	6.719701918	2.496746901	0.022313757	-3.362176542
ST8SIA1	-0.691859396	2.327267794	-2.493805046	0.022451036	-3.36748427
EBP	-0.639630132	4.131719168	-2.490691711	0.022597181	-3.37309831
GDAP1	-0.660893631	3.422345212	-2.490210769	0.022619837	-3.373965272
ITGBL1	1.037623677	6.413080305	2.488669331	0.022692595	-3.376743415
SOX4	1.31424054	6.203557844	2.481093618	0.023053386	-3.390385836
ARSI	0.72296102	3.584384927	2.476658813	0.023267089	-3.398363299
ST6GALNAC4	0.646916844	4.957470309	2.469231318	0.023629178	-3.411709495
LXN	-0.677328198	5.265312325	-2.466651365	0.023756184	-3.416341018
PTHLH	0.916276265	3.364521179	2.459032176	0.024135015	-3.430005942
RIN2	1.33630252	8.525354169	2.458395056	0.024166948	-3.431147725
PRKD1	-0.750476475	7.712391413	-2.457069709	0.024233504	-3.433522442
GGH	-0.686338886	3.664010962	-2.450632941	0.024559197	-3.445047197
CFB	-0.949588012	8.19427599	-2.4499915	0.024591878	-3.446194901
ADRB2	-0.649528608	6.511622085	-2.436002065	0.025314837	-3.471190676
PWAR6	0.730460559	4.301220665	2.434041275	0.025417746	-3.474688776
T	-1.110196968	3.303458428	-2.430401385	0.02560982	-3.481178915
AKR1B1	0.703648946	10.15379566	2.429687732	0.025647638	-3.482450863
PPP1R3B	-0.682472263	3.762350094	-2.429354681	0.025665305	-3.483044402
TEX9	-0.697446364	5.191342719	-2.417024226	0.026327458	-3.504991669
RARB	0.677047506	3.496219123	2.414351397	0.026473084	-3.509742093
MFSD6	0.659882802	4.241377684	2.410476765	0.026685526	-3.516624031
SETD6	0.836178424	5.599965637	2.406662625	0.026896207	-3.523393356
POGLUT1	0.719927233	5.343333667	2.405396765	0.026966473	-3.525638864
HCG4	-1.067704671	5.88368378	-2.402896491	0.027105762	-3.53007243
TMEM158	-0.665782824	3.124162256	-2.402198838	0.027144748	-3.531309135
GCLM	0.69453052	10.17731161	2.401700088	0.027172651	-3.532193145
TMPRSS4	-1.003004733	4.284404656	-2.399706998	0.027284423	-3.53572492
SERTAD1	-0.815909338	7.738225585	-2.398771231	0.027337049	-3.537382621
LPAR4	0.620010754	3.967442531	2.39394458	0.027610001	-3.545928015
C2orf82	-0.973139072	9.66575813	-2.393322722	0.027645353	-3.547028384
KCNN4	1.026644575	5.42941119	2.391070637	0.027773732	-3.55101226
ALCAM	1.001591531	6.761156895	2.388868401	0.027899809	-3.554906195
PEG10	-0.838044139	3.807839231	-2.386149351	0.028056209	-3.559711539
CCDC151	-0.658612039	5.363220352	-2.383782405	0.028193022	-3.563892446
SDC4	-0.656148987	10.95827336	-2.373080248	0.028819428	-3.582771114
WIF1	-1.715006608	8.34271622	-2.371269508	0.028926687	-3.585961149
KRT19	-1.69930153	3.266810402	-2.370812444	0.02895382	-3.586766184
DLGAP1-AS2	-0.851582677	4.553687296	-2.36587377	0.029248513	-3.595459875
COL4A1	1.354341619	4.236660092	2.365396101	0.029277163	-3.596300256
LINC00847	-0.667552481	4.994823898	-2.362565295	0.029447492	-3.601278877

MAP3K5	1.006143517	5.996563382	2.361202602	0.029529813	-3.603674433
SFT2D2	0.624485061	5.301756589	2.357331055	0.029764867	-3.61047671
GLDN	-0.856739093	7.252431799	-2.356805378	0.029796917	-3.611399894
HMGCR	-0.761316296	9.054546469	-2.354116888	0.029961332	-3.616119766
PLEKHG4	-0.774570837	3.836470354	-2.352727625	0.030046623	-3.618557684
NANOS1	1.252369519	5.373884289	2.348606294	0.030300975	-3.625785696
CALR	0.629276801	6.401017725	2.342970827	0.030652017	-3.635658956
CYP4X1	-0.601336866	3.298271676	-2.341104183	0.030769125	-3.63892667
RAP1A	1.098144622	10.0031597	2.329705914	0.031493287	-3.658851844
MRC2	-0.749423329	6.787448288	-2.328366741	0.0315794	-3.661189613
PRMT6	0.93043048	5.671687135	2.32712231	0.031659616	-3.663361383
CTSO	-0.786147848	5.246573993	-2.326255384	0.031715611	-3.664873988
ERICH1	-0.74165731	7.525949983	-2.321257891	0.032040192	-3.673587991
PSRC1	0.837189332	3.457604019	2.312473991	0.032618179	-3.688881128
MGLL	-0.644756712	7.493849041	-2.311313137	0.032695283	-3.690900008
TTC32	-0.67655601	7.32501152	-2.310406718	0.032755605	-3.69247603
LAYN	-0.682890901	6.670144699	-2.298037088	0.033589181	-3.713951805
CHAC2	1.230877027	6.83995027	2.293410694	0.033905966	-3.721968732
RAB38	-0.61373157	3.241618285	-2.29239111	0.033976151	-3.723734412
SCO2	0.682870414	5.525654479	2.288467245	0.034247516	-3.730525826
MICB	0.753092434	3.759730666	2.287347354	0.034325332	-3.732463022
ZNF329	0.675116368	3.53209718	2.286521293	0.034382835	-3.733891633
LY96	0.625991448	4.150975691	2.283962909	0.034561496	-3.738314464
GUSBP11	0.675040372	3.973502238	2.275935186	0.035127688	-3.752175693
PTGR2	0.788738507	4.833441385	2.274234932	0.035248701	-3.755108196
PCSK1	-0.705530066	4.422808089	-2.265714218	0.035860975	-3.769786906
WBP1L	0.601894932	5.518600641	2.258769625	0.036367242	-3.781728936
AKNAD1	-0.800868386	4.215921636	-2.257215462	0.036481441	-3.784398844
DUSP5	1.00414603	7.814128073	2.25704395	0.036494064	-3.784693427
NTN4	0.875262725	9.21080194	2.254644413	0.036671088	-3.78881353
HLF	-0.74724514	4.167907102	-2.25422416	0.036702173	-3.789534883
SPARCL1	2.001784532	3.691416945	2.236879955	0.03800648	-3.819243179
GADD45G	-0.716677607	5.489179051	-2.219486309	0.039357255	-3.848912312
RERG	1.024758116	3.742576386	2.219302008	0.039371802	-3.849226012
VEGFC	0.974780245	2.387305088	2.211770983	0.03997044	-3.862032522
SLC26A6	0.615416533	3.285801324	2.206941524	0.040358721	-3.870232538
MMP2	2.159729649	3.138074124	2.206114252	0.040425579	-3.871636195
WARS	0.692057236	8.328529825	2.205670961	0.040461446	-3.872388221
TGFB2	0.878193779	2.904148765	2.197041865	0.041165484	-3.887010658
ZNF33B	-0.631264428	3.555384751	-2.195530731	0.041289927	-3.889568113
JARID2	0.820190904	5.535795949	2.190445955	0.041711197	-3.898166508
PDGFA	0.615082998	3.910405822	2.19021362	0.041730539	-3.898559126
TSLP	-1.304916385	6.38596644	-2.190108097	0.041739327	-3.898737438
RFTN1	0.891740977	2.777841836	2.18968155	0.041774867	-3.899458171
CPVL	-1.497938496	3.348890963	-2.180411216	0.042554149	-3.915102992
BIRC3	0.930350197	6.23224317	2.170165861	0.043430864	-3.932350363
KLF3-AS1	-0.929494849	5.413994425	-2.160432341	0.044279057	-3.948694034
SESN3	0.967629831	4.396013691	2.159943993	0.044322009	-3.94951294
FAM110C	0.732406743	6.311995436	2.158318523	0.044465248	-3.952237923
MXRA8	0.81567749	6.061506528	2.157118748	0.044571246	-3.95424852
ITGA5	0.776464284	7.450091967	2.155896878	0.044679433	-3.956295496
TRIM22	0.927204938	2.81784448	2.144092507	0.045737033	-3.97603741
THBS1	0.891974197	7.715623398	2.143638137	0.045778195	-3.976796082

COL4A2	0.957525746	2.841571169	2.137911878	0.046299846	-3.986349534
ADAMTS6	0.618311361	2.292504944	2.136812694	0.046400598	-3.988181705
BANK1	-0.916547129	4.781557977	-2.133296104	0.046724273	-3.994039709
CYTL1	1.120473104	10.25093291	2.13212611	0.046832418	-3.995987489
NUDT7	-0.748413119	3.524870553	-2.131327552	0.046906361	-3.99731656
TXNIP	0.745181624	9.816409075	2.128116597	0.047204756	-4.002657802
FLJ38717	-0.683259755	7.548562844	-2.126552047	0.047350776	-4.005258674
CHURC1	-0.961747139	7.49569332	-2.114851153	0.04845593	-4.024675131
HMGCS1	-0.703017141	6.319842625	-2.109808726	0.048939383	-4.033023495
CA9	-0.969804714	5.221236282	-2.106843294	0.049225743	-4.037927756
STEAP1	0.719695812	6.108703305	2.100641368	0.049829568	-4.048171629
CA12	-0.921574562	5.523197057	-2.098824098	0.050007769	-4.051169937
CPM	0.748499621	5.769458789	2.092939585	0.050588779	-4.060868396
WFDC21P	0.615124748	4.514516434	2.09070113	0.050811398	-4.064553496
GATA3	0.675335671	2.465536105	2.085215736	0.051360692	-4.073574176
KCNK6	0.600072133	5.03123157	2.084105011	0.051472571	-4.075399064
SOD2	0.616225932	11.33518612	2.073101977	0.05259285	-4.093445814
ANPEP	0.815558458	2.978158167	2.072347823	0.052670437	-4.094680685
SLC30A1	-0.671869884	7.10994136	-2.07133415	0.052774887	-4.096340084
PPL	-0.679926214	3.665830867	-2.071024002	0.052806883	-4.096847706
NDNF	-0.645495629	3.99234836	-2.068763661	0.053040594	-4.100545862
PON3	-0.729296038	4.82169351	-2.067784496	0.053142126	-4.102147137
CMAHP	0.691458583	7.816022562	2.064136909	0.053521904	-4.108108261
SLC7A2	0.634810732	10.14951375	2.05593991	0.054384325	-4.121481517
MANF	0.649189878	10.03092987	2.050741699	0.054937726	-4.129945878
PRC1	-0.616572389	4.131022728	-2.049179677	0.055105009	-4.13248685
JUN	-0.657667437	10.02559697	-2.048595991	0.055167636	-4.133436048
INHBA	-0.750644755	10.33232053	-2.042429198	0.055833246	-4.143454663
C5orf34	0.716818878	4.355913493	2.040971601	0.055991625	-4.145820039
RP2	0.695483436	7.0857732	2.037381293	0.056383472	-4.151642029
ZNF165	-1.341815051	5.893896719	-2.032842509	0.056882372	-4.15899322
C12orf66	-0.629695888	3.817954447	-2.032249384	0.056947861	-4.159953137
SEMA3C	0.720913216	7.644521497	2.029389357	0.057264601	-4.164579453
NEFM	-1.383725998	3.786927988	-2.028140029	0.057403458	-4.166599103
CD151	0.66444258	5.474846302	2.021727909	0.058120919	-4.176953011
APCDD1L	0.934299364	3.362981658	2.019319545	0.058392472	-4.180836754
LINC01207	-0.638573114	2.86051803	-2.012856975	0.059126803	-4.19124442
CBLB	0.765218655	9.368922984	2.012000022	0.059224798	-4.192622977
TCIRG1	0.628075926	4.361403756	2.009385771	0.059524649	-4.196826236
FICD	0.886852444	6.706343039	2.0074717	0.059745053	-4.19990161
COL10A1	1.932913349	8.489563227	2.005019847	0.060028455	-4.203838431
CHADL	-0.630115409	6.112223472	-2.001893307	0.060391592	-4.20885429
LTBP1	0.926148712	6.023971462	1.999660378	0.060652146	-4.212433603
GULP1	0.677995909	9.38967272	1.997651498	0.060887417	-4.215651677
SQLE	-0.76041121	7.203009278	-1.989146761	0.061892552	-4.229253565
SBF2-AS1	-0.641143088	3.637086904	-1.98911611	0.061896202	-4.229302523
HYOU1	0.766832636	7.625758506	1.986432228	0.062216488	-4.233587465
DHCR7	-0.640851976	5.148917633	-1.985589057	0.062317416	-4.234932888
CHRDL2	-1.542476353	7.880595402	-1.983836154	0.062527708	-4.237728812
BEND6	0.649324673	2.843722531	1.978424196	0.063180985	-4.246351371
BMP6	-0.733402542	10.26337414	-1.9731159	0.063827679	-4.254794559
FDPS	-0.702142228	8.790030277	-1.971725429	0.063998051	-4.25700386
SLC47A1	0.65681352	3.638844012	1.971009745	0.064085902	-4.258140622

ULBP2	-0.692333623	7.24096976	-1.965540567	0.064760813	-4.266819138
AKR1C1	0.969042257	10.67837144	1.954348575	0.066161764	-4.284531583
FZD10	1.169980341	6.490733904	1.951739074	0.066492271	-4.288652249
HSF2BP	-0.728803275	3.750662299	-1.949324464	0.066799405	-4.292462076
SPAG5-AS1	-0.635082761	5.073647212	-1.945187399	0.067328574	-4.298982721
SGK223	-0.674644091	3.745275862	-1.943345973	0.067565309	-4.301882277
RCAN1	-0.956223093	5.508761752	-1.940373518	0.067949015	-4.306559117
NPR3	0.821358864	3.700003739	1.935061239	0.0686396	-4.314906123
C10orf10	1.12899078	6.63472512	1.932962715	0.06891412	-4.318199465
RDH10	0.8734151	6.195993085	1.931900637	0.069053429	-4.319865383
HTRA1	-0.673254778	11.23011457	-1.92957367	0.069359525	-4.323513304
CFD	0.830344012	6.068912969	1.924852754	0.069984239	-4.330905547
COL2A1	-0.696273174	11.17707598	-1.924268748	0.070061866	-4.33181921
GPR56	0.649150697	5.334007023	1.92084756	0.070518158	-4.33716802
ADAMTS5	1.28388547	4.023819124	1.920209546	0.070603544	-4.338164842
MOXD1	-0.799079215	5.076741852	-1.917688598	0.07094182	-4.342101454
RBPMS2	-0.636834953	6.727615442	-1.915364332	0.071254978	-4.345728002
IFITM2	1.01210015	7.461920203	1.909574635	0.072040392	-4.354749388
NAP1L5	-0.758565007	6.717886741	-1.907667563	0.072300776	-4.357717107
GPR133	-0.819014878	5.570151218	-1.905533062	0.072593203	-4.36103648
PCDHB16	0.779864329	6.366669226	1.901273678	0.073179874	-4.367653114
FXYD6	-0.679808751	8.494103155	-1.900594691	0.073273782	-4.368706986
CYR61	-0.625980472	9.005881409	-1.892636238	0.074382473	-4.381041355
LRP12	0.705354033	6.466673754	1.880940069	0.076038808	-4.399107648
PCOLCE	-0.789230958	4.756184776	-1.88032562	0.076126716	-4.400054732
DPT	-0.95804974	4.582316946	-1.876192565	0.076720366	-4.406419988
DSC2	-0.792575928	4.916929099	-1.874469482	0.076969065	-4.40907098
F13A1	0.674457757	10.74985972	1.864259779	0.078457321	-4.424746048
PTGER2	0.730492374	5.542352339	1.863445246	0.078577141	-4.42599419
TSIX	-1.013860489	2.891868057	-1.854388245	0.079920348	-4.439848403
HOTAIRM1	0.756048061	4.954982219	1.850499183	0.080503296	-4.445783713
SLC26A9	0.876068972	3.0927986	1.83684548	0.082579627	-4.466555974
SERPING1	-0.790840869	9.144305563	-1.83636754	0.082653152	-4.467281245
CHI3L1	1.04702941	10.16905298	1.832342485	0.083274641	-4.473384241
FOS	-0.73298083	10.50980034	-1.828011445	0.083947953	-4.479941212
PNMAL1	-0.862913886	5.285443935	-1.826722331	0.08414928	-4.481890862
PLD6	-0.648316746	4.136457328	-1.820402553	0.08514241	-4.491435544
ECM1	-0.695657071	3.980817774	-1.814370778	0.086099841	-4.500524561
LOC100507557	-0.661932532	4.909905465	-1.803596275	0.087833552	-4.516709632
RGS16	-0.894682326	6.96045809	-1.793109981	0.089550098	-4.532399158
NEFH	-0.602797325	2.530969723	-1.768905122	0.093624365	-4.568376241
SDF2L1	0.788145212	5.731750285	1.762282958	0.094766739	-4.578160764
P4HA3	-0.60061747	4.588384553	-1.750195247	0.096883179	-4.595955604
VCAM1	1.00613448	1.981728171	1.743383894	0.098093728	-4.605945551
ACAT2	-0.606430447	8.105795313	-1.73870722	0.098932451	-4.612788987
CIDEA	-1.157833577	4.694817322	-1.735537246	0.099504479	-4.617420379
LNX1	0.652474837	5.826488453	1.735374057	0.099534004	-4.617658641
SPHK1	0.664246694	4.831613915	1.726408508	0.101167773	-4.6307247
GADD45B	-0.60748828	10.56449483	-1.721828887	0.1020112	-4.63738061
COL1A1	1.561899014	4.413434314	1.721798022	0.102016905	-4.637425427
TCEA3	-0.624307496	6.288904458	-1.720587274	0.10224091	-4.639183006
C15orf48	0.873224733	3.01078111	1.719266519	0.102485751	-4.641099289
LIPG	-0.6224129	2.498899733	-1.718244014	0.102675649	-4.642582131

DNAJB6	0.604636661	8.213520664	1.7096079	0.104291665	-4.65508154
GPR158	-0.896824418	3.392508502	-1.704056233	0.105342029	-4.663093271
TREM1	1.132566215	6.531866671	1.703707578	0.105408296	-4.663595809
PGD	0.790790511	7.113332759	1.693926985	0.107281901	-4.677663531
MCOLN3	0.690854307	6.198549745	1.686985685	0.108628878	-4.68761256
GALNT3	-0.893574203	5.00829656	-1.686404758	0.108742263	-4.688443892
COL12A1	0.724529537	3.06367415	1.685884746	0.108843844	-4.689187878
C3orf14	-0.696886959	7.14521368	-1.684620303	0.109091186	-4.69099625
ABCA1	0.721459629	4.054811049	1.683949502	0.109222599	-4.691955218
HTR2A	0.633295763	3.076335484	1.676227976	0.110745068	-4.702974201
AKAP12	-0.735264442	5.085072905	-1.676149258	0.110760682	-4.703086349
SLCO4A1	0.7803145	4.257542262	1.674936591	0.111001458	-4.70481354
CST3	-0.600317146	9.246796716	-1.669191213	0.112148297	-4.712984473
OGN	-0.8235482	10.72987023	-1.661569689	0.113685245	-4.723792543
FRZB	-0.725953103	9.710007442	-1.65914217	0.114178535	-4.727227542
SRGN	1.24578231	7.337322479	1.649667901	0.116121275	-4.740599239
IFIT1	0.896930185	4.17978396	1.642791658	0.117548842	-4.750269514
H1F0	-0.809126068	8.474223268	-1.633937776	0.119408937	-4.762677875
WISP2	-0.767048191	3.68961009	-1.631234023	0.119981921	-4.766457365
PDGFRA	0.718276129	6.881485687	1.628831711	0.120492978	-4.769811658
S100A4	-0.918014506	9.890372442	-1.620048588	0.122377199	-4.782044639
SERPINF1	0.911183564	2.773237997	1.614887398	0.12349601	-4.789210502
TMEM140	0.775747069	4.238138417	1.605444013	0.125565487	-4.802278407
NOV	-0.872307573	5.7157124	-1.598303393	0.127149696	-4.81212228
BEX5	-0.663806559	8.489763292	-1.594080136	0.12809457	-4.817929126
F5	-0.869858669	3.59286688	-1.582649935	0.130681564	-4.833588282
CILP	-1.282928999	10.93487455	-1.571422753	0.133265218	-4.848887849
MEOX2	0.788794905	2.36591769	1.565669531	0.134605694	-4.856696464
LOC643733	0.611392825	2.178495284	1.559618257	0.136027782	-4.864886523
CD14	0.930312482	5.830440883	1.557150801	0.136611247	-4.868219273
GPX3	0.631663555	11.94040155	1.545123433	0.139485317	-4.88440772
TGFBI	-0.998025778	9.248750478	-1.540645295	0.140568224	-4.890411021
GCA	-0.734279161	5.801770727	-1.534488581	0.142068473	-4.898643127
IFIT2	0.90988601	4.358912579	1.533372022	0.142341976	-4.900133406
ATP8B1	0.621042142	3.122909117	1.525825836	0.144201925	-4.910183813
ELTD1	0.850126076	2.002360633	1.522199895	0.145102785	-4.914999662
KCNK5	0.689635766	8.536153971	1.507166846	0.148887673	-4.934873036
TWIST1	1.218011882	4.774403321	1.503026796	0.149944249	-4.940319669
SFN	-0.712891366	9.802057948	-1.500920316	0.150484215	-4.94308655
PMAIP1	1.125070008	5.462375861	1.499346856	0.150888596	-4.945151366
RSPO3	0.999312087	4.492803702	1.487545375	0.153950269	-4.960585229
BLM	-0.691988303	6.05098946	-1.4858561	0.154392678	-4.96278678
FRMD6-AS1	-0.632020041	5.358616867	-1.467946266	0.159147781	-4.986009097
DUSP4	0.729761022	5.331767513	1.462045408	0.160740536	-4.993612563
SPINK1	-0.895545196	4.226659066	-1.461878823	0.16078569	-4.993826869
PRSS23	-0.688082114	8.001956368	-1.459231592	0.161504622	-4.997229896
TNFAIP3	0.816330263	4.735620665	1.455703166	0.162466957	-5.001758255
TF	1.148324759	5.834584753	1.453833063	0.162978902	-5.004154876
MAMDC2	0.859841288	2.824469981	1.451183406	0.163706508	-5.007546423
S100A8	0.801524528	2.371766442	1.442874045	0.166005506	-5.018151071
ORM1	0.760501199	10.07472243	1.440126421	0.166771472	-5.021647205
OLFML2A	-0.624998921	2.634443024	-1.417993943	0.173046868	-5.049618322
ABCB4	-0.729041974	4.261912971	-1.3965101	0.17931998	-5.076442666

ECHDC3	-0.610102389	3.624662281	-1.390306691	0.181165048	-5.0841277
NPTX2	1.025810715	5.708976578	1.37966759	0.184364936	-5.097244408
ACVR1C	-0.674516869	4.058362309	-1.365105233	0.188818118	-5.115067425
NUP62CL	-0.640393618	5.508595751	-1.3567832	0.191401354	-5.125184717
SPOCK1	-0.687837487	6.536665631	-1.351999834	0.192898861	-5.130977445
HBD	0.962478241	2.713908188	1.348692712	0.193939651	-5.134972781
ENO2	-0.69249002	7.052139171	-1.343363863	0.195626095	-5.141393954
MMP13	0.803193923	2.431184909	1.327560179	0.200696092	-5.160316109
SGCG	-0.735159353	4.215024694	-1.319611573	0.203285091	-5.169764457
CYP1A1	0.704437875	3.703156002	1.296928923	0.210818183	-5.196472163
MAFB	0.685257313	9.001363568	1.287416639	0.214041724	-5.207559457
HBB	1.715659644	6.045222103	1.287035605	0.214171648	-5.208002185
XIST	-2.153036302	5.572347819	-1.28110053	0.216203339	-5.21488428
COCH	-0.877505296	3.194703815	-1.279956599	0.216596652	-5.216207728
CSRP2	-0.60451921	6.853436888	-1.277866737	0.217316641	-5.218623036
DDX3Y	1.650926795	6.173892428	1.266968366	0.221101549	-5.231165739
CAPN8	-0.698371916	3.429126217	-1.263726605	0.222237213	-5.234879465
EFEMP1	0.709831138	2.923384874	1.257078708	0.224580277	-5.242470551
ASPN	-1.281810393	7.229044445	-1.246827217	0.228230867	-5.254111302
PTX3	0.628749122	9.081778302	1.207900584	0.242510498	-5.297586915
PSMD5-AS1	-0.604654797	4.446621638	-1.198382852	0.246103544	-5.308040499
MAOA	-0.844258958	3.933539367	-1.163851325	0.259479152	-5.345378735
LAMB3	-0.854947648	5.837784052	-1.139902175	0.269071439	-5.370727378
MXRA5	-0.691795937	4.958971456	-1.139007716	0.269434742	-5.371665356
GPNMB	0.853281917	3.930335782	1.135315329	0.270938346	-5.3755307
TAC1	-0.920534623	6.089479029	-1.094415975	0.28801121	-5.417620724
THBD	0.621734241	6.694403231	1.094177378	0.288113069	-5.417862343
EPYC	1.068359299	6.958993118	1.074742291	0.296498638	-5.43738942
COL15A1	0.881012439	5.303107181	1.029913415	0.316511535	-5.481260181
EIF1AY	1.327561734	5.501764709	1.017677087	0.322137632	-5.49294867
FABP4	0.744000157	3.381845807	1.010666005	0.325392934	-5.499590096
POSTN	1.176463468	4.245419841	0.992036553	0.334155137	-5.517039075
RPS4Y1	1.733505767	7.98573024	0.985671534	0.337186347	-5.522934497
KDM5D	1.046614283	4.718077983	0.951531943	0.353770852	-5.553975409
USP9Y	0.77481378	3.656179028	0.919395961	0.369884819	-5.582294084
PAX8-AS1	-0.621137587	5.392151753	-0.885678382	0.3873155	-5.611057476
AGR2	0.684029639	5.914248162	0.840887816	0.411296438	-5.647746538
CCL20	0.658580958	6.44383656	0.745352418	0.465553269	-5.720105722
PRG4	0.801002282	9.763376807	0.738371004	0.46968127	-5.725074374

Supplementary Table 4. The regulatory network of

miRNA	mRNA	Target	mirnaLogFC	mrnaLogFC
hsa-miR-93-5p	ZFPM2	target	4.022878217	-1.118446022
hsa-miR-378h	ZFPM2	target	2.142129164	-1.118446022
hsa-miR-378c	ZFPM2	target	2.172121133	-1.118446022
hsa-miR-301a-3p	ZFPM2	target	4.377116122	-1.118446022
hsa-miR-103a-3p	ZFPM2	target	1.973244744	-1.118446022
hsa-miR-96-5p	ZFP36L1	target	-3.69065925	1.528745549
hsa-miR-377-3p	ZFP36L1	target	-2.083955044	1.528745549
hsa-miR-29b-3p	ZFP36L1	target	-3.614460994	1.528745549
hsa-miR-29a-3p	ZFP36L1	target	-7.710169639	1.528745549
hsa-miR-27b-3p	ZFP36L1	target	-8.0156531	1.528745549
hsa-miR-27a-3p	ZFP36L1	target	-7.027874406	1.528745549
hsa-miR-181d-5p	ZFP36L1	target	-2.538257378	1.528745549
hsa-miR-181c-5p	ZFP36L1	target	-5.975134372	1.528745549
hsa-miR-181b-5p	ZFP36L1	target	-4.229699617	1.528745549
hsa-miR-181a-5p	ZFP36L1	target	-8.086162172	1.528745549
hsa-miR-128-3p	ZFP36L1	target	-3.2456183	1.528745549
hsa-miR-1271-5p	ZFP36L1	target	-3.057933239	1.528745549
hsa-miR-532-3p	ZEB2	target	-3.840000894	1.650922664
hsa-miR-502-3p	ZEB2	target	-2.727154031	1.650922664
hsa-miR-377-3p	ZEB2	target	-2.083955044	1.650922664
hsa-miR-16-5p	WIF1	target	2.170767889	-1.715006608
hsa-miR-16-5p	VAMP8	target	2.170767889	-1.572016478
hsa-miR-103a-3p	VAMP8	target	1.973244744	-1.572016478
hsa-miR-96-5p	TWIST1	target	-3.69065925	1.218011882
hsa-miR-214-5p	TWIST1	target	-3.476266878	1.218011882
hsa-miR-151a-3p	TWIST1	target	-4.3826325	1.218011882
hsa-miR-1271-5p	TWIST1	target	-3.057933239	1.218011882
hsa-miR-23b-3p	TNFAIP6	target	-13.60579603	1.376896967
hsa-miR-23a-3p	TNFAIP6	target	-14.74406994	1.376896967
hsa-miR-27b-3p	TFPI	target	-8.0156531	2.055735683
hsa-miR-27a-3p	TFPI	target	-7.027874406	2.055735683
hsa-miR-374c-5p	TAF13	target	-2.066745369	1.061927181
hsa-miR-454-3p	SULF1	target	-1.793108858	1.096191063
hsa-miR-19a-3p	SULF1	target	-2.335140461	1.096191063
hsa-miR-199b-5p	SULF1	target	-6.704986694	1.096191063
hsa-miR-199a-5p	SULF1	target	-7.464223567	1.096191063
hsa-miR-152-3p	SULF1	target	-5.366644456	1.096191063
hsa-miR-148b-3p	SULF1	target	-3.844370844	1.096191063
hsa-miR-148a-3p	SULF1	target	-6.9178194	1.096191063
hsa-miR-23c	SPTSSB	target	3.754610411	-1.26286057
hsa-miR-182-5p	SPTSSB	target	2.937748733	-1.26286057
hsa-miR-454-3p	SOX4	target	-1.793108858	1.31424054
hsa-miR-3666	SOX4	target	-1.888857728	1.31424054
hsa-miR-214-5p	SOX4	target	-3.476266878	1.31424054
hsa-miR-20b-5p	SOX4	target	-2.910447375	1.31424054
hsa-miR-20a-5p	SOX4	target	-3.760302156	1.31424054
hsa-miR-19b-3p	SOX4	target	-5.130085539	1.31424054
hsa-miR-19a-3p	SOX4	target	-2.335140461	1.31424054
hsa-miR-17-5p	SOX4	target	-2.907048831	1.31424054
hsa-miR-140-5p	SOX4	target	-7.6651035	1.31424054
hsa-miR-132-3p	SOX4	target	-3.373475364	1.31424054

hsa-miR-130b-3p	SOX4	target	-5.001102139	1.31424054
hsa-miR-130a-3p	SOX4	target	-7.083430956	1.31424054
hsa-miR-106b-5p	SOX4	target	-5.687222906	1.31424054
hsa-miR-98-5p	SMIM3	target	-5.885407408	2.303470985
hsa-let-7i-5p	SMIM3	target	-10.44359602	2.303470985
hsa-let-7g-5p	SMIM3	target	-9.970715511	2.303470985
hsa-let-7f-5p	SMIM3	target	-12.84678967	2.303470985
hsa-let-7e-5p	SMIM3	target	-8.750119272	2.303470985
hsa-let-7c-5p	SMIM3	target	-13.21630578	2.303470985
hsa-let-7b-5p	SMIM3	target	-25.01169358	2.303470985
hsa-let-7a-5p	SMIM3	target	-13.68176841	2.303470985
hsa-miR-324-5p	SLITRK4	target	-3.233792569	1.151402046
hsa-miR-145-5p	SLITRK4	target	-4.396319578	1.151402046
hsa-miR-194-5p	SLC40A1	target	-1.448772494	1.240892822
hsa-miR-222-3p	SLC16A6	target	-4.5975339	1.239865427
hsa-miR-221-3p	SLC16A6	target	-7.122492578	1.239865427
hsa-miR-20b-5p	SLC16A6	target	-2.910447375	1.239865427
hsa-miR-20a-5p	SLC16A6	target	-3.760302156	1.239865427
hsa-miR-193b-3p	SLC16A6	target	-4.598413106	1.239865427
hsa-miR-193a-3p	SLC16A6	target	-1.549934411	1.239865427
hsa-miR-17-5p	SLC16A6	target	-2.907048831	1.239865427
hsa-miR-152-3p	SLC16A6	target	-5.366644456	1.239865427
hsa-miR-148b-3p	SLC16A6	target	-3.844370844	1.239865427
hsa-miR-148a-3p	SLC16A6	target	-6.9178194	1.239865427
hsa-miR-106b-5p	SLC16A6	target	-5.687222906	1.239865427
hsa-miR-92a-3p	S100A2	target	2.653037772	-1.563529121
hsa-miR-19b-3p	RIN2	target	-5.130085539	1.33630252
hsa-miR-19a-3p	RIN2	target	-2.335140461	1.33630252
hsa-miR-144-3p	RIN2	target	-2.246704806	1.33630252
hsa-miR-223-3p	RERG	target	-5.561533161	1.024758116
hsa-miR-182-5p	RAPGEF5	target	2.937748733	-1.172797805
hsa-miR-502-3p	RAP1A	target	-2.727154031	1.098144622
hsa-miR-320d	RAP1A	target	-6.863046828	1.098144622
hsa-miR-320b	RAP1A	target	-6.736763533	1.098144622
hsa-miR-320a	RAP1A	target	-5.854823167	1.098144622
hsa-miR-24-3p	RAP1A	target	-8.852555428	1.098144622
hsa-miR-19b-3p	RAP1A	target	-5.130085539	1.098144622
hsa-miR-19a-3p	RAP1A	target	-2.335140461	1.098144622
hsa-miR-149-5p	RAP1A	target	-3.016498922	1.098144622
hsa-miR-454-3p	PXDN	target	-1.793108858	1.143151734
hsa-miR-29b-3p	PXDN	target	-3.614460994	1.143151734
hsa-miR-29a-3p	PXDN	target	-7.710169639	1.143151734
hsa-miR-19b-3p	PXDN	target	-5.130085539	1.143151734
hsa-miR-19a-3p	PXDN	target	-2.335140461	1.143151734
hsa-miR-19b-3p	POSTN	target	-5.130085539	1.176463468
hsa-miR-19a-3p	POSTN	target	-2.335140461	1.176463468
hsa-miR-98-5p	PMAIP1	target	-5.885407408	1.125070008
hsa-miR-23b-3p	PMAIP1	target	-13.60579603	1.125070008
hsa-miR-23a-3p	PMAIP1	target	-14.74406994	1.125070008
hsa-miR-144-3p	PMAIP1	target	-2.246704806	1.125070008
hsa-miR-142-3p	PMAIP1	target	-2.005220017	1.125070008
hsa-let-7i-5p	PMAIP1	target	-10.44359602	1.125070008
hsa-let-7g-5p	PMAIP1	target	-9.970715511	1.125070008

hsa-let-7f-5p	PMAIP1	target	-12.84678967	1.125070008
hsa-let-7e-5p	PMAIP1	target	-8.750119272	1.125070008
hsa-let-7c-5p	PMAIP1	target	-13.21630578	1.125070008
hsa-let-7b-5p	PMAIP1	target	-25.01169358	1.125070008
hsa-let-7a-5p	PMAIP1	target	-13.68176841	1.125070008
hsa-miR-92a-3p	PALLD	target	2.653037772	-1.196309679
hsa-miR-34a-5p	PALLD	target	17.52203323	-1.196309679
hsa-miR-182-5p	PALLD	target	2.937748733	-1.196309679
hsa-miR-29c-3p	NUDT11	target	21.99464321	-1.007698068
hsa-miR-1271-5p	NPTX2	target	-3.057933239	1.025810715
hsa-miR-92a-3p	NEFM	target	2.653037772	-1.383725998
hsa-let-7d-5p	NEFM	target	1.549151417	-1.383725998
hsa-miR-96-5p	NANOS1	target	-3.69065925	1.252369519
hsa-miR-708-5p	NANOS1	target	-4.858929633	1.252369519
hsa-miR-29b-3p	NANOS1	target	-3.614460994	1.252369519
hsa-miR-29a-3p	NANOS1	target	-7.710169639	1.252369519
hsa-miR-28-5p	NANOS1	target	-2.831242669	1.252369519
hsa-miR-20b-5p	NANOS1	target	-2.910447375	1.252369519
hsa-miR-20a-5p	NANOS1	target	-3.760302156	1.252369519
hsa-miR-17-5p	NANOS1	target	-2.907048831	1.252369519
hsa-miR-1271-5p	NANOS1	target	-3.057933239	1.252369519
hsa-miR-106b-5p	NANOS1	target	-5.687222906	1.252369519
hsa-miR-20b-5p	MMP2	target	-2.910447375	2.159729649
hsa-miR-20a-5p	MMP2	target	-3.760302156	2.159729649
hsa-miR-17-5p	MMP2	target	-2.907048831	2.159729649
hsa-miR-106b-5p	MMP2	target	-5.687222906	2.159729649
hsa-miR-20b-5p	MAP3K8	target	-2.910447375	1.091357104
hsa-miR-20a-5p	MAP3K8	target	-3.760302156	1.091357104
hsa-miR-17-5p	MAP3K8	target	-2.907048831	1.091357104
hsa-miR-144-3p	MAP3K8	target	-2.246704806	1.091357104
hsa-miR-106b-5p	MAP3K8	target	-5.687222906	1.091357104
hsa-miR-425-5p	MAP3K5	target	-3.864192222	1.006143517
hsa-miR-20b-5p	MAP3K5	target	-2.910447375	1.006143517
hsa-miR-20a-5p	MAP3K5	target	-3.760302156	1.006143517
hsa-miR-199a-3p	MAP3K5	target	-8.798220544	1.006143517
hsa-miR-17-5p	MAP3K5	target	-2.907048831	1.006143517
hsa-miR-106b-5p	MAP3K5	target	-5.687222906	1.006143517
hsa-miR-5195-3p	LYVE1	target	6.534320478	-1.50362957
hsa-miR-339-5p	LYVE1	target	5.250190517	-1.50362957
hsa-let-7d-5p	LYVE1	target	1.549151417	-1.50362957
hsa-miR-223-3p	LMO2	target	-5.561533161	1.920074763
hsa-miR-301a-3p	LDLR	target	4.377116122	-1.601830469
hsa-miR-497-5p	KCNN4	target	-6.000204056	1.026644575
hsa-miR-424-5p	KCNN4	target	-2.868822222	1.026644575
hsa-miR-195-5p	KCNN4	target	-7.895415794	1.026644575
hsa-miR-15b-5p	KCNN4	target	-7.4728635	1.026644575
hsa-miR-15a-5p	KCNN4	target	-7.474686522	1.026644575
hsa-miR-374c-5p	IRX5	target	-2.066745369	1.229607855
hsa-miR-222-3p	IRX5	target	-4.5975339	1.229607855
hsa-miR-221-3p	IRX5	target	-7.122492578	1.229607855
hsa-miR-493-5p	IRX3	target	-2.287147097	1.533096142
hsa-miR-377-3p	IRX3	target	-2.083955044	1.533096142
hsa-miR-340-5p	IGFBP3	target	-2.299785889	3.236412271

hsa-miR-19b-3p	IGFBP3	target	-5.130085539	3.236412271
hsa-miR-19a-3p	IGFBP3	target	-2.335140461	3.236412271
hsa-miR-877-5p	HSD11B1	target	-3.979460428	1.48908376
hsa-miR-340-5p	HSD11B1	target	-2.299785889	1.48908376
hsa-miR-132-3p	HSD11B1	target	-3.373475364	1.48908376
hsa-miR-324-5p	hsa_circ_100906	target	-3.233792569	1.03217136
hsa-miR-182-5p	hsa_circ_100896	target	2.937748733	-1.27638136
hsa-miR-339-5p	hsa_circ_100893	target	5.250190517	-1.0679431
hsa-miR-339-5p	hsa_circ_100892	target	5.250190517	-1.00594502
hsa-miR-98-5p	hsa_circ_100882	target	-5.885407408	1.4391607
hsa-miR-665	hsa_circ_100882	target	-1.51720975	1.4391607
hsa-let-7i-5p	hsa_circ_100882	target	-10.44359602	1.4391607
hsa-let-7g-5p	hsa_circ_100882	target	-9.970715511	1.4391607
hsa-let-7f-5p	hsa_circ_100882	target	-12.84678967	1.4391607
hsa-let-7e-5p	hsa_circ_100882	target	-8.750119272	1.4391607
hsa-let-7c-5p	hsa_circ_100882	target	-13.21630578	1.4391607
hsa-let-7b-5p	hsa_circ_100882	target	-25.01169358	1.4391607
hsa-let-7a-5p	hsa_circ_100882	target	-13.68176841	1.4391607
hsa-miR-665	hsa_circ_100876	target	-1.51720975	1.91686344
hsa-miR-320c	hsa_circ_100844	target	1.871100867	-2.38715834
hsa-miR-16-5p	hsa_circ_100844	target	2.170767889	-2.38715834
hsa-miR-103a-3p	hsa_circ_100844	target	1.973244744	-2.38715834
hsa-miR-182-5p	hsa_circ_100836	target	2.937748733	-1.42659808
hsa-miR-520b	hsa_circ_100823	target	7.073403739	-1.22774796
hsa-miR-708-5p	hsa_circ_100815	target	-4.858929633	1.06539044
hsa-miR-29b-3p	hsa_circ_100815	target	-3.614460994	1.06539044
hsa-miR-29a-3p	hsa_circ_100815	target	-7.710169639	1.06539044
hsa-miR-28-5p	hsa_circ_100815	target	-2.831242669	1.06539044
hsa-miR-28-5p	hsa_circ_100815	target	-2.831242669	1.06539044
hsa-miR-665	hsa_circ_100802	target	-1.51720975	1.62299234
hsa-miR-425-5p	hsa_circ_100802	target	-3.864192222	1.62299234
hsa-miR-182-5p	hsa_circ_100772	target	2.937748733	-2.6984196
hsa-miR-16-5p	hsa_circ_100772	target	2.170767889	-2.6984196
hsa-miR-103a-3p	hsa_circ_100772	target	1.973244744	-2.6984196
hsa-miR-760	hsa_circ_100723	target	3.333884622	-1.88863978
hsa-miR-5195-3p	hsa_circ_100723	target	6.534320478	-1.88863978
hsa-miR-455-5p	hsa_circ_100723	target	17.33461372	-1.88863978
hsa-miR-376b-3p	hsa_circ_100723	target	3.651956789	-1.88863978
hsa-miR-301a-3p	hsa_circ_100723	target	4.377116122	-1.88863978
hsa-miR-103a-3p	hsa_circ_100723	target	1.973244744	-1.88863978
hsa-miR-5195-3p	hsa_circ_100701	target	6.534320478	-1.98306782
hsa-miR-134-5p	hsa_circ_100701	target	4.379412022	-1.98306782
hsa-miR-5195-3p	hsa_circ_100698	target	6.534320478	-2.41655144
hsa-miR-134-5p	hsa_circ_100698	target	4.379412022	-2.41655144
hsa-miR-376c-3p	hsa_circ_100688	target	-4.708504289	1.02484966
hsa-miR-98-5p	hsa_circ_100685	target	-5.885407408	1.04794418
hsa-miR-532-5p	hsa_circ_100685	target	-5.295597783	1.04794418
hsa-miR-532-5p	hsa_circ_100685	target	-5.295597783	1.04794418
hsa-miR-377-3p	hsa_circ_100685	target	-2.083955044	1.04794418
hsa-miR-29b-3p	hsa_circ_100685	target	-3.614460994	1.04794418
hsa-miR-29a-3p	hsa_circ_100685	target	-7.710169639	1.04794418
hsa-miR-199a-3p	hsa_circ_100685	target	-8.798220544	1.04794418
hsa-miR-193b-3p	hsa_circ_100685	target	-4.598413106	1.04794418

hsa-miR-193a-3p	hsa_circ_100685	target	-1.549934411	1.04794418
hsa-let-7i-5p	hsa_circ_100685	target	-10.44359602	1.04794418
hsa-let-7g-5p	hsa_circ_100685	target	-9.970715511	1.04794418
hsa-let-7f-5p	hsa_circ_100685	target	-12.84678967	1.04794418
hsa-let-7e-5p	hsa_circ_100685	target	-8.750119272	1.04794418
hsa-let-7c-5p	hsa_circ_100685	target	-13.21630578	1.04794418
hsa-let-7b-5p	hsa_circ_100685	target	-25.01169358	1.04794418
hsa-let-7a-5p	hsa_circ_100685	target	-13.68176841	1.04794418
hsa-miR-98-5p	hsa_circ_100668	target	-5.885407408	1.59573632
hsa-miR-96-5p	hsa_circ_100668	target	-3.69065925	1.59573632
hsa-miR-374c-5p	hsa_circ_100668	target	-2.066745369	1.59573632
hsa-miR-149-5p	hsa_circ_100668	target	-3.016498922	1.59573632
hsa-miR-149-5p	hsa_circ_100668	target	-3.016498922	1.59573632
hsa-miR-143-3p	hsa_circ_100668	target	-2.558538	1.59573632
hsa-miR-132-3p	hsa_circ_100668	target	-3.373475364	1.59573632
hsa-miR-1271-5p	hsa_circ_100668	target	-3.057933239	1.59573632
hsa-miR-101-3p	hsa_circ_100668	target	-4.824183106	1.59573632
hsa-let-7i-5p	hsa_circ_100668	target	-10.44359602	1.59573632
hsa-let-7g-5p	hsa_circ_100668	target	-9.970715511	1.59573632
hsa-let-7f-5p	hsa_circ_100668	target	-12.84678967	1.59573632
hsa-let-7e-5p	hsa_circ_100668	target	-8.750119272	1.59573632
hsa-let-7c-5p	hsa_circ_100668	target	-13.21630578	1.59573632
hsa-let-7b-5p	hsa_circ_100668	target	-25.01169358	1.59573632
hsa-let-7a-5p	hsa_circ_100668	target	-13.68176841	1.59573632
hsa-miR-320d	hsa_circ_100646	target	-6.863046828	2.04021004
hsa-miR-520b	hsa_circ_100640	target	7.073403739	-1.50197352
hsa-miR-5195-3p	hsa_circ_100633	target	6.534320478	-1.81514382
hsa-miR-27b-3p	hsa_circ_100604	target	-8.0156531	2.67199762
hsa-miR-27a-3p	hsa_circ_100604	target	-7.027874406	2.67199762
hsa-miR-144-3p	hsa_circ_100604	target	-2.246704806	2.67199762
hsa-miR-708-5p	hsa_circ_100600	target	-4.858929633	1.03440484
hsa-miR-497-5p	hsa_circ_100600	target	-6.000204056	1.03440484
hsa-miR-424-5p	hsa_circ_100600	target	-2.868822222	1.03440484
hsa-miR-28-5p	hsa_circ_100600	target	-2.831242669	1.03440484
hsa-miR-223-3p	hsa_circ_100600	target	-5.561533161	1.03440484
hsa-miR-195-5p	hsa_circ_100600	target	-7.895415794	1.03440484
hsa-miR-194-5p	hsa_circ_100600	target	-1.448772494	1.03440484
hsa-miR-15b-5p	hsa_circ_100600	target	-7.4728635	1.03440484
hsa-miR-15a-5p	hsa_circ_100600	target	-7.474686522	1.03440484
hsa-miR-383-5p	hsa_circ_100579	target	2.153888914	-1.60890002
hsa-miR-502-3p	hsa_circ_100571	target	-2.727154031	2.00809222
hsa-miR-29b-3p	hsa_circ_100525	target	-3.614460994	1.41262602
hsa-miR-29a-3p	hsa_circ_100525	target	-7.710169639	1.41262602
hsa-miR-134-5p	hsa_circ_100477	target	4.379412022	-1.18776898
hsa-miR-320c	hsa_circ_100476	target	1.871100867	-2.162316
hsa-miR-134-5p	hsa_circ_100476	target	4.379412022	-2.162316
hsa-miR-497-5p	hsa_circ_100470	target	-6.000204056	1.22561234
hsa-miR-424-5p	hsa_circ_100470	target	-2.868822222	1.22561234
hsa-miR-195-5p	hsa_circ_100470	target	-7.895415794	1.22561234
hsa-miR-15b-5p	hsa_circ_100470	target	-7.4728635	1.22561234
hsa-miR-15a-5p	hsa_circ_100470	target	-7.474686522	1.22561234
hsa-miR-152-3p	hsa_circ_100453	target	-5.366644456	1.09279654
hsa-miR-148b-3p	hsa_circ_100453	target	-3.844370844	1.09279654

hsa-miR-148a-3p	hsa_circ_100453	target	-6.9178194	1.09279654
hsa-miR-182-5p	hsa_circ_100443	target	2.937748733	-1.01819448
hsa-miR-374c-5p	hsa_circ_100422	target	-2.066745369	1.57102538
hsa-miR-374c-5p	hsa_circ_100421	target	-2.066745369	1.50703614
hsa-miR-103a-3p	hsa_circ_100420	target	1.973244744	-1.65018748
hsa-miR-103a-3p	hsa_circ_100420	target	1.973244744	-1.65018748
hsa-miR-454-3p	hsa_circ_100411	target	-1.793108858	1.31800384
hsa-miR-3666	hsa_circ_100411	target	-1.888857728	1.31800384
hsa-miR-19b-3p	hsa_circ_100411	target	-5.130085539	1.31800384
hsa-miR-19a-3p	hsa_circ_100411	target	-2.335140461	1.31800384
hsa-miR-152-3p	hsa_circ_100411	target	-5.366644456	1.31800384
hsa-miR-148b-3p	hsa_circ_100411	target	-3.844370844	1.31800384
hsa-miR-148a-3p	hsa_circ_100411	target	-6.9178194	1.31800384
hsa-miR-130b-3p	hsa_circ_100411	target	-5.001102139	1.31800384
hsa-miR-130a-3p	hsa_circ_100411	target	-7.083430956	1.31800384
hsa-miR-93-5p	hsa_circ_100407	target	4.022878217	-1.40093756
hsa-miR-301a-3p	hsa_circ_100407	target	4.377116122	-1.40093756
hsa-miR-181d-5p	hsa_circ_100374	target	-2.538257378	2.11558444
hsa-miR-181c-5p	hsa_circ_100374	target	-5.975134372	2.11558444
hsa-miR-181b-5p	hsa_circ_100374	target	-4.229699617	2.11558444
hsa-miR-181a-5p	hsa_circ_100374	target	-8.086162172	2.11558444
hsa-miR-196b-5p	hsa_circ_100350	target	-3.858799039	1.10611924
hsa-miR-196a-5p	hsa_circ_100350	target	-6.248512556	1.10611924
hsa-miR-212-3p	hsa_circ_100332	target	4.252929717	-1.285155
hsa-miR-23b-3p	hsa_circ_100329	target	-13.60579603	1.45547144
hsa-miR-23a-3p	hsa_circ_100329	target	-14.74406994	1.45547144
hsa-miR-455-5p	hsa_circ_100290	target	17.33461372	-2.1012706
hsa-miR-29c-3p	hsa_circ_100290	target	21.99464321	-2.1012706
hsa-miR-29c-3p	hsa_circ_100290	target	21.99464321	-2.1012706
hsa-miR-383-5p	hsa_circ_100283	target	2.153888914	-1.17878776
hsa-miR-34a-5p	hsa_circ_100270	target	17.52203323	-1.4706532
hsa-miR-378h	hsa_circ_100266	target	2.142129164	-1.0576901
hsa-miR-378c	hsa_circ_100266	target	2.172121133	-1.0576901
hsa-miR-877-5p	hsa_circ_100236	target	-3.979460428	1.52880272
hsa-miR-143-3p	hsa_circ_100236	target	-2.558538	1.52880272
hsa-miR-24-3p	hsa_circ_100227	target	-8.852555428	1.87763952
hsa-miR-24-3p	hsa_circ_100227	target	-8.852555428	1.87763952
hsa-miR-24-3p	hsa_circ_100226	target	-8.852555428	1.5606671
hsa-miR-24-3p	hsa_circ_100226	target	-8.852555428	1.5606671
hsa-miR-199a-3p	hsa_circ_100226	target	-8.798220544	1.5606671
hsa-miR-194-5p	hsa_circ_100226	target	-1.448772494	1.5606671
hsa-miR-378h	hsa_circ_100219	target	2.142129164	-1.2632643
hsa-miR-378c	hsa_circ_100219	target	2.172121133	-1.2632643
hsa-miR-182-5p	hsa_circ_100219	target	2.937748733	-1.2632643
hsa-miR-378h	hsa_circ_100213	target	2.142129164	-1.63603092
hsa-miR-378c	hsa_circ_100213	target	2.172121133	-1.63603092
hsa-miR-34a-5p	hsa_circ_100213	target	17.52203323	-1.63603092
hsa-miR-320c	hsa_circ_100213	target	1.871100867	-1.63603092
hsa-miR-128-3p	hsa_circ_100202	target	-3.2456183	1.63754254
hsa-miR-760	hsa_circ_100188	target	3.333884622	-1.29563954
hsa-miR-378h	hsa_circ_100188	target	2.142129164	-1.29563954
hsa-miR-378c	hsa_circ_100188	target	2.172121133	-1.29563954
hsa-miR-301a-3p	hsa_circ_100188	target	4.377116122	-1.29563954

hsa-miR-29c-3p	hsa_circ_100188	target	21.99464321	-1.29563954
hsa-miR-29c-3p	hsa_circ_100188	target	21.99464321	-1.29563954
hsa-miR-16-5p	hsa_circ_100188	target	2.170767889	-1.29563954
hsa-miR-134-5p	hsa_circ_100188	target	4.379412022	-1.29563954
hsa-miR-103a-3p	hsa_circ_100188	target	1.973244744	-1.29563954
hsa-miR-96-5p	hsa_circ_100177	target	-3.69065925	1.39822884
hsa-miR-1271-5p	hsa_circ_100177	target	-3.057933239	1.39822884
hsa-miR-98-5p	hsa_circ_100117	target	-5.885407408	1.66873586
hsa-miR-196b-5p	hsa_circ_100117	target	-3.858799039	1.66873586
hsa-miR-196a-5p	hsa_circ_100117	target	-6.248512556	1.66873586
hsa-miR-142-3p	hsa_circ_100117	target	-2.005220017	1.66873586
hsa-let-7i-5p	hsa_circ_100117	target	-10.44359602	1.66873586
hsa-let-7g-5p	hsa_circ_100117	target	-9.970715511	1.66873586
hsa-let-7f-5p	hsa_circ_100117	target	-12.84678967	1.66873586
hsa-let-7e-5p	hsa_circ_100117	target	-8.750119272	1.66873586
hsa-let-7c-5p	hsa_circ_100117	target	-13.21630578	1.66873586
hsa-let-7b-5p	hsa_circ_100117	target	-25.01169358	1.66873586
hsa-let-7a-5p	hsa_circ_100117	target	-13.68176841	1.66873586
hsa-miR-151a-3p	hsa_circ_100086	target	-4.3826325	2.23862012
hsa-miR-151a-3p	hsa_circ_100085	target	-4.3826325	2.0592209
hsa-miR-497-5p	hsa_circ_100045	target	-6.000204056	1.2146054
hsa-miR-493-5p	hsa_circ_100045	target	-2.287147097	1.2146054
hsa-miR-424-5p	hsa_circ_100045	target	-2.868822222	1.2146054
hsa-miR-195-5p	hsa_circ_100045	target	-7.895415794	1.2146054
hsa-miR-15b-5p	hsa_circ_100045	target	-7.4728635	1.2146054
hsa-miR-15a-5p	hsa_circ_100045	target	-7.474686522	1.2146054
hsa-miR-16-5p	hsa_circ_100037	target	2.170767889	-1.43572602
hsa-miR-16-5p	hsa_circ_100037	target	2.170767889	-1.43572602
hsa-miR-103a-3p	hsa_circ_100037	target	1.973244744	-1.43572602
hsa-miR-16-5p	hsa_circ_100036	target	2.170767889	-1.16736316
hsa-miR-16-5p	hsa_circ_100036	target	2.170767889	-1.16736316
hsa-miR-103a-3p	hsa_circ_100036	target	1.973244744	-1.16736316
hsa-miR-16-5p	hsa_circ_100035	target	2.170767889	-1.51137716
hsa-miR-16-5p	hsa_circ_100035	target	2.170767889	-1.51137716
hsa-miR-103a-3p	hsa_circ_100035	target	1.973244744	-1.51137716
hsa-miR-16-5p	hsa_circ_100034	target	2.170767889	-1.56709236
hsa-miR-16-5p	hsa_circ_100034	target	2.170767889	-1.56709236
hsa-miR-103a-3p	hsa_circ_100034	target	1.973244744	-1.56709236
hsa-miR-16-5p	hsa_circ_100033	target	2.170767889	-1.31053668
hsa-miR-760	hsa_circ_100004	target	3.333884622	-1.17333056
hsa-miR-520b	hsa_circ_100004	target	7.073403739	-1.17333056
hsa-miR-103a-3p	hsa_circ_002106	target	1.973244744	-1.03718066
hsa-miR-93-5p	hsa_circ_002086	target	4.022878217	-2.11371662
hsa-miR-520b	hsa_circ_002086	target	7.073403739	-2.11371662
hsa-miR-5195-3p	hsa_circ_002086	target	6.534320478	-2.11371662
hsa-miR-16-5p	hsa_circ_002086	target	2.170767889	-2.11371662
hsa-miR-92a-3p	hsa_circ_001769	target	2.653037772	-1.1074031
hsa-miR-16-5p	hsa_circ_001769	target	2.170767889	-1.1074031
hsa-miR-377-3p	hsa_circ_001653	target	-2.083955044	2.22151978
hsa-miR-152-3p	hsa_circ_001653	target	-5.366644456	2.22151978
hsa-miR-148b-3p	hsa_circ_001653	target	-3.844370844	2.22151978
hsa-miR-148a-3p	hsa_circ_001653	target	-6.9178194	2.22151978
hsa-miR-502-3p	hsa_circ_001405	target	-2.727154031	1.49576896

hsa-miR-377-3p	hsa_circ_001405	target	-2.083955044	1.49576896
hsa-miR-532-3p	hsa_circ_001175	target	-3.840000894	2.28857146
hsa-miR-497-5p	hsa_circ_001175	target	-6.000204056	2.28857146
hsa-miR-497-5p	hsa_circ_001175	target	-6.000204056	2.28857146
hsa-miR-424-5p	hsa_circ_001175	target	-2.868822222	2.28857146
hsa-miR-424-5p	hsa_circ_001175	target	-2.868822222	2.28857146
hsa-miR-376c-3p	hsa_circ_001175	target	-4.708504289	2.28857146
hsa-miR-374c-5p	hsa_circ_001175	target	-2.066745369	2.28857146
hsa-miR-374c-5p	hsa_circ_001175	target	-2.066745369	2.28857146
hsa-miR-340-5p	hsa_circ_001175	target	-2.299785889	2.28857146
hsa-miR-320d	hsa_circ_001175	target	-6.863046828	2.28857146
hsa-miR-320b	hsa_circ_001175	target	-6.736763533	2.28857146
hsa-miR-320a	hsa_circ_001175	target	-5.854823167	2.28857146
hsa-miR-27b-3p	hsa_circ_001175	target	-8.0156531	2.28857146
hsa-miR-27a-3p	hsa_circ_001175	target	-7.027874406	2.28857146
hsa-miR-222-3p	hsa_circ_001175	target	-4.5975339	2.28857146
hsa-miR-221-3p	hsa_circ_001175	target	-7.122492578	2.28857146
hsa-miR-218-5p	hsa_circ_001175	target	-2.920309808	2.28857146
hsa-miR-214-5p	hsa_circ_001175	target	-3.476266878	2.28857146
hsa-miR-20b-5p	hsa_circ_001175	target	-2.910447375	2.28857146
hsa-miR-20a-5p	hsa_circ_001175	target	-3.760302156	2.28857146
hsa-miR-19b-3p	hsa_circ_001175	target	-5.130085539	2.28857146
hsa-miR-19b-3p	hsa_circ_001175	target	-5.130085539	2.28857146
hsa-miR-19a-3p	hsa_circ_001175	target	-2.335140461	2.28857146
hsa-miR-19a-3p	hsa_circ_001175	target	-2.335140461	2.28857146
hsa-miR-199b-5p	hsa_circ_001175	target	-6.704986694	2.28857146
hsa-miR-199b-5p	hsa_circ_001175	target	-6.704986694	2.28857146
hsa-miR-199a-5p	hsa_circ_001175	target	-7.464223567	2.28857146
hsa-miR-199a-5p	hsa_circ_001175	target	-7.464223567	2.28857146
hsa-miR-195-5p	hsa_circ_001175	target	-7.895415794	2.28857146
hsa-miR-195-5p	hsa_circ_001175	target	-7.895415794	2.28857146
hsa-miR-194-5p	hsa_circ_001175	target	-1.448772494	2.28857146
hsa-miR-181d-5p	hsa_circ_001175	target	-2.538257378	2.28857146
hsa-miR-181d-5p	hsa_circ_001175	target	-2.538257378	2.28857146
hsa-miR-181c-5p	hsa_circ_001175	target	-5.975134372	2.28857146
hsa-miR-181c-5p	hsa_circ_001175	target	-5.975134372	2.28857146
hsa-miR-181b-5p	hsa_circ_001175	target	-4.229699617	2.28857146
hsa-miR-181b-5p	hsa_circ_001175	target	-4.229699617	2.28857146
hsa-miR-181a-5p	hsa_circ_001175	target	-8.086162172	2.28857146
hsa-miR-181a-5p	hsa_circ_001175	target	-8.086162172	2.28857146
hsa-miR-17-5p	hsa_circ_001175	target	-2.907048831	2.28857146
hsa-miR-15b-5p	hsa_circ_001175	target	-7.4728635	2.28857146
hsa-miR-15b-5p	hsa_circ_001175	target	-7.4728635	2.28857146
hsa-miR-15a-5p	hsa_circ_001175	target	-7.474686522	2.28857146
hsa-miR-15a-5p	hsa_circ_001175	target	-7.474686522	2.28857146
hsa-miR-151a-3p	hsa_circ_001175	target	-4.3826325	2.28857146
hsa-miR-145-5p	hsa_circ_001175	target	-4.396319578	2.28857146
hsa-miR-143-3p	hsa_circ_001175	target	-2.558538	2.28857146
hsa-miR-142-3p	hsa_circ_001175	target	-2.005220017	2.28857146
hsa-miR-128-3p	hsa_circ_001175	target	-3.2456183	2.28857146
hsa-miR-106b-5p	hsa_circ_001175	target	-5.687222906	2.28857146
hsa-miR-331-3p	hsa_circ_001109	target	-3.481406728	1.1582763
hsa-miR-206	hsa_circ_001109	target	-3.869800733	1.1582763

hsa-miR-1-3p	hsa_circ_001109	target	-3.903586272	1.1582763
hsa-miR-324-5p	hsa_circ_001100	target	-3.233792569	1.2243097
hsa-miR-23c	hsa_circ_001072	target	3.754610411	-1.28896542
hsa-miR-199b-5p	hsa_circ_001046	target	-6.704986694	1.3837194
hsa-miR-199a-5p	hsa_circ_001046	target	-7.464223567	1.3837194
hsa-miR-143-3p	hsa_circ_001046	target	-2.558538	1.3837194
hsa-miR-383-5p	hsa_circ_000943	target	2.153888914	-2.56591412
hsa-miR-16-5p	hsa_circ_000941	target	2.170767889	-2.26248584
hsa-miR-320d	hsa_circ_000926	target	-6.863046828	1.08593422
hsa-miR-320d	hsa_circ_000926	target	-6.863046828	1.08593422
hsa-miR-320b	hsa_circ_000926	target	-6.736763533	1.08593422
hsa-miR-320b	hsa_circ_000926	target	-6.736763533	1.08593422
hsa-miR-320a	hsa_circ_000926	target	-5.854823167	1.08593422
hsa-miR-320a	hsa_circ_000926	target	-5.854823167	1.08593422
hsa-miR-222-3p	hsa_circ_000926	target	-4.5975339	1.08593422
hsa-miR-221-3p	hsa_circ_000926	target	-7.122492578	1.08593422
hsa-miR-140-5p	hsa_circ_000926	target	-7.6651035	1.08593422
hsa-miR-149-5p	hsa_circ_000881	target	-3.016498922	2.02632116
hsa-miR-493-5p	hsa_circ_000791	target	-2.287147097	1.116836
hsa-miR-425-5p	hsa_circ_000791	target	-3.864192222	1.116836
hsa-miR-376c-3p	hsa_circ_000791	target	-4.708504289	1.116836
hsa-miR-340-5p	hsa_circ_000791	target	-2.299785889	1.116836
hsa-miR-23c	hsa_circ_000750	target	3.754610411	-2.3494982
hsa-miR-98-5p	hsa_circ_000684	target	-5.885407408	1.38981146
hsa-let-7i-5p	hsa_circ_000684	target	-10.44359602	1.38981146
hsa-let-7g-5p	hsa_circ_000684	target	-9.970715511	1.38981146
hsa-let-7f-5p	hsa_circ_000684	target	-12.84678967	1.38981146
hsa-let-7e-5p	hsa_circ_000684	target	-8.750119272	1.38981146
hsa-let-7c-5p	hsa_circ_000684	target	-13.21630578	1.38981146
hsa-let-7b-5p	hsa_circ_000684	target	-25.01169358	1.38981146
hsa-let-7a-5p	hsa_circ_000684	target	-13.68176841	1.38981146
hsa-miR-222-3p	hsa_circ_000200	target	-4.5975339	2.48946402
hsa-miR-221-3p	hsa_circ_000200	target	-7.122492578	2.48946402
hsa-miR-132-3p	hsa_circ_000200	target	-3.373475364	2.48946402
hsa-miR-708-5p	hsa_circ_000178	target	-4.858929633	2.45118972
hsa-miR-497-5p	hsa_circ_000178	target	-6.000204056	2.45118972
hsa-miR-424-5p	hsa_circ_000178	target	-2.868822222	2.45118972
hsa-miR-28-5p	hsa_circ_000178	target	-2.831242669	2.45118972
hsa-miR-195-5p	hsa_circ_000178	target	-7.895415794	2.45118972
hsa-miR-15b-5p	hsa_circ_000178	target	-7.4728635	2.45118972
hsa-miR-15a-5p	hsa_circ_000178	target	-7.474686522	2.45118972
hsa-miR-149-5p	hsa_circ_000178	target	-3.016498922	2.45118972
hsa-let-7d-5p	hsa_circ_000094	target	1.549151417	-1.7381442
hsa-let-7d-5p	hsa_circ_000094	target	1.549151417	-1.7381442
hsa-miR-760	HIST1H2AE	target	3.333884622	-1.478890701
hsa-miR-93-5p	HAS2	target	4.022878217	-1.435044745
hsa-miR-92a-3p	HAS2	target	2.653037772	-1.435044745
hsa-miR-455-5p	HAS2	target	17.33461372	-1.435044745
hsa-miR-376b-3p	HAS2	target	3.651956789	-1.435044745
hsa-miR-23c	HAS2	target	3.754610411	-1.435044745
hsa-miR-212-3p	HAS2	target	4.252929717	-1.435044745
hsa-miR-182-5p	HAS2	target	2.937748733	-1.435044745
hsa-miR-16-5p	HAS2	target	2.170767889	-1.435044745

hsa-miR-134-5p	HAS2	target	4.379412022	-1.435044745
hsa-let-7d-5p	HAS2	target	1.549151417	-1.435044745
hsa-miR-218-5p	GREM1	target	-2.920309808	1.224310377
hsa-miR-199a-3p	GREM1	target	-8.798220544	1.224310377
hsa-miR-193b-3p	GREM1	target	-4.598413106	1.224310377
hsa-miR-193a-3p	GREM1	target	-1.549934411	1.224310377
hsa-miR-142-3p	GREM1	target	-2.005220017	1.224310377
hsa-miR-128-3p	GREM1	target	-3.2456183	1.224310377
hsa-miR-532-5p	GBP1	target	-5.295597783	1.03638574
hsa-miR-27b-3p	GATA6	target	-8.0156531	1.903094593
hsa-miR-27a-3p	GATA6	target	-7.027874406	1.903094593
hsa-miR-196b-5p	GATA6	target	-3.858799039	1.903094593
hsa-miR-196a-5p	GATA6	target	-6.248512556	1.903094593
hsa-miR-181d-5p	GATA6	target	-2.538257378	1.903094593
hsa-miR-181c-5p	GATA6	target	-5.975134372	1.903094593
hsa-miR-181b-5p	GATA6	target	-4.229699617	1.903094593
hsa-miR-181a-5p	GATA6	target	-8.086162172	1.903094593
hsa-miR-128-3p	GATA6	target	-3.2456183	1.903094593
hsa-miR-93-5p	FOXQ1	target	4.022878217	-1.382805735
hsa-miR-320c	FOXQ1	target	1.871100867	-1.382805735
hsa-miR-182-5p	FOXQ1	target	2.937748733	-1.382805735
hsa-miR-520b	FOXF2	target	7.073403739	-1.394098208
hsa-miR-301a-3p	FOXF2	target	4.377116122	-1.394098208
hsa-miR-182-5p	FOXF2	target	2.937748733	-1.394098208
hsa-miR-497-5p	FAT4	target	-6.000204056	1.446208783
hsa-miR-424-5p	FAT4	target	-2.868822222	1.446208783
hsa-miR-195-5p	FAT4	target	-7.895415794	1.446208783
hsa-miR-193b-3p	FAT4	target	-4.598413106	1.446208783
hsa-miR-193a-3p	FAT4	target	-1.549934411	1.446208783
hsa-miR-15b-5p	FAT4	target	-7.4728635	1.446208783
hsa-miR-15a-5p	FAT4	target	-7.474686522	1.446208783
hsa-miR-144-3p	FAT4	target	-2.246704806	1.446208783
hsa-miR-29c-3p	ENPP2	target	21.99464321	-1.518038678
hsa-miR-196b-5p	EBF1	target	-3.858799039	1.498717114
hsa-miR-196a-5p	EBF1	target	-6.248512556	1.498717114
hsa-miR-331-3p	DUSP5	target	-3.481406728	1.00414603
hsa-miR-27b-3p	DUSP5	target	-8.0156531	1.00414603
hsa-miR-27a-3p	DUSP5	target	-7.027874406	1.00414603
hsa-miR-23b-3p	DUSP5	target	-13.60579603	1.00414603
hsa-miR-23a-3p	DUSP5	target	-14.74406994	1.00414603
hsa-miR-218-5p	DUSP5	target	-2.920309808	1.00414603
hsa-miR-199a-3p	DUSP5	target	-8.798220544	1.00414603
hsa-miR-128-3p	DUSP5	target	-3.2456183	1.00414603
hsa-miR-19b-3p	DMXL2	target	-5.130085539	1.210445757
hsa-miR-19a-3p	DMXL2	target	-2.335140461	1.210445757
hsa-miR-665	DLX3	target	-1.51720975	1.147831995
hsa-miR-19b-3p	DLX3	target	-5.130085539	1.147831995
hsa-miR-19a-3p	DLX3	target	-2.335140461	1.147831995
hsa-miR-520b	DKK1	target	7.073403739	-1.142102848
hsa-miR-103a-3p	DKK1	target	1.973244744	-1.142102848
hsa-miR-497-5p	DDX3Y	target	-6.000204056	1.650926795
hsa-miR-424-5p	DDX3Y	target	-2.868822222	1.650926795
hsa-miR-29b-3p	DDX3Y	target	-3.614460994	1.650926795

hsa-miR-29a-3p	DDX3Y	target	-7.710169639	1.650926795
hsa-miR-19b-3p	DDX3Y	target	-5.130085539	1.650926795
hsa-miR-19a-3p	DDX3Y	target	-2.335140461	1.650926795
hsa-miR-199b-5p	DDX3Y	target	-6.704986694	1.650926795
hsa-miR-199a-5p	DDX3Y	target	-7.464223567	1.650926795
hsa-miR-195-5p	DDX3Y	target	-7.895415794	1.650926795
hsa-miR-15b-5p	DDX3Y	target	-7.4728635	1.650926795
hsa-miR-15a-5p	DDX3Y	target	-7.474686522	1.650926795
hsa-miR-101-3p	DDX3Y	target	-4.824183106	1.650926795
hsa-miR-5195-3p	CRISPLD2	target	6.534320478	-1.233301817
hsa-miR-376b-3p	CRISPLD2	target	3.651956789	-1.233301817
hsa-miR-29c-3p	COL6A2	target	21.99464321	-1.093370373
hsa-miR-98-5p	COL4A1	target	-5.885407408	1.354341619
hsa-miR-29b-3p	COL4A1	target	-3.614460994	1.354341619
hsa-miR-29a-3p	COL4A1	target	-7.710169639	1.354341619
hsa-miR-152-3p	COL4A1	target	-5.366644456	1.354341619
hsa-miR-148b-3p	COL4A1	target	-3.844370844	1.354341619
hsa-miR-148a-3p	COL4A1	target	-6.9178194	1.354341619
hsa-let-7f-5p	COL4A1	target	-12.84678967	1.354341619
hsa-let-7e-5p	COL4A1	target	-8.750119272	1.354341619
hsa-let-7c-5p	COL4A1	target	-13.21630578	1.354341619
hsa-let-7a-5p	COL4A1	target	-13.68176841	1.354341619
hsa-miR-98-5p	COL1A1	target	-5.885407408	1.561899014
hsa-miR-29b-3p	COL1A1	target	-3.614460994	1.561899014
hsa-miR-29a-3p	COL1A1	target	-7.710169639	1.561899014
hsa-miR-218-5p	COL1A1	target	-2.920309808	1.561899014
hsa-miR-196b-5p	COL1A1	target	-3.858799039	1.561899014
hsa-miR-196a-5p	COL1A1	target	-6.248512556	1.561899014
hsa-miR-143-3p	COL1A1	target	-2.558538	1.561899014
hsa-let-7i-5p	COL1A1	target	-10.44359602	1.561899014
hsa-let-7g-5p	COL1A1	target	-9.970715511	1.561899014
hsa-let-7f-5p	COL1A1	target	-12.84678967	1.561899014
hsa-let-7e-5p	COL1A1	target	-8.750119272	1.561899014
hsa-let-7c-5p	COL1A1	target	-13.21630578	1.561899014
hsa-let-7b-5p	COL1A1	target	-25.01169358	1.561899014
hsa-let-7a-5p	COL1A1	target	-13.68176841	1.561899014
hsa-miR-502-3p	COL10A1	target	-2.727154031	1.932913349
hsa-miR-101-3p	COL10A1	target	-4.824183106	1.932913349
hsa-miR-497-5p	CNIH3	target	-6.000204056	1.001700646
hsa-miR-424-5p	CNIH3	target	-2.868822222	1.001700646
hsa-miR-195-5p	CNIH3	target	-7.895415794	1.001700646
hsa-miR-15b-5p	CNIH3	target	-7.4728635	1.001700646
hsa-miR-15a-5p	CNIH3	target	-7.474686522	1.001700646
hsa-miR-383-5p	CLEC3A	target	2.153888914	-1.708521224
hsa-miR-23c	CHST10	target	3.754610411	-1.103610816
hsa-miR-24-3p	CHI3L1	target	-8.852555428	1.04702941
hsa-miR-27b-3p	CDH11	target	-8.0156531	1.113361817
hsa-miR-27a-3p	CDH11	target	-7.027874406	1.113361817
hsa-miR-144-3p	CDH11	target	-2.246704806	1.113361817
hsa-miR-128-3p	CDH11	target	-3.2456183	1.113361817
hsa-miR-101-3p	CDH11	target	-4.824183106	1.113361817
hsa-miR-497-5p	CCND1	target	-6.000204056	1.484930221
hsa-miR-424-5p	CCND1	target	-2.868822222	1.484930221

hsa-miR-20b-5p	CCND1	target	-2.910447375	1.484930221
hsa-miR-20a-5p	CCND1	target	-3.760302156	1.484930221
hsa-miR-19b-3p	CCND1	target	-5.130085539	1.484930221
hsa-miR-19a-3p	CCND1	target	-2.335140461	1.484930221
hsa-miR-195-5p	CCND1	target	-7.895415794	1.484930221
hsa-miR-193b-3p	CCND1	target	-4.598413106	1.484930221
hsa-miR-193a-3p	CCND1	target	-1.549934411	1.484930221
hsa-miR-17-5p	CCND1	target	-2.907048831	1.484930221
hsa-miR-15b-5p	CCND1	target	-7.4728635	1.484930221
hsa-miR-15a-5p	CCND1	target	-7.474686522	1.484930221
hsa-miR-142-3p	CCND1	target	-2.005220017	1.484930221
hsa-miR-106b-5p	CCND1	target	-5.687222906	1.484930221
hsa-let-7i-5p	CCND1	target	-10.44359602	1.484930221
hsa-let-7b-5p	CCND1	target	-25.01169358	1.484930221
hsa-miR-29c-3p	C4orf32	target	21.99464321	-1.15013865
hsa-miR-92a-3p	ASPN	target	2.653037772	-1.281810393
hsa-miR-222-3p	ASPH	target	-4.5975339	1.212288754
hsa-miR-221-3p	ASPH	target	-7.122492578	1.212288754
hsa-miR-206	ASPH	target	-3.869800733	1.212288754
hsa-miR-152-3p	ASPH	target	-5.366644456	1.212288754
hsa-miR-148b-3p	ASPH	target	-3.844370844	1.212288754
hsa-miR-148a-3p	ASPH	target	-6.9178194	1.212288754
hsa-miR-142-3p	ASPH	target	-2.005220017	1.212288754
hsa-miR-1-3p	ASPH	target	-3.903586272	1.212288754
hsa-miR-376c-3p	ALCAM	target	-4.708504289	1.001591531
hsa-miR-152-3p	ALCAM	target	-5.366644456	1.001591531
hsa-miR-148b-3p	ALCAM	target	-3.844370844	1.001591531
hsa-miR-148a-3p	ALCAM	target	-6.9178194	1.001591531
hsa-miR-142-3p	ALCAM	target	-2.005220017	1.001591531
hsa-miR-96-5p	AHR	target	-3.69065925	1.096324214
hsa-miR-502-3p	AHR	target	-2.727154031	1.096324214
hsa-miR-29b-3p	AHR	target	-3.614460994	1.096324214
hsa-miR-29a-3p	AHR	target	-7.710169639	1.096324214
hsa-miR-1271-5p	AHR	target	-3.057933239	1.096324214
hsa-miR-98-5p	ADAMTS5	target	-5.885407408	1.28388547
hsa-miR-497-5p	ADAMTS5	target	-6.000204056	1.28388547
hsa-miR-424-5p	ADAMTS5	target	-2.868822222	1.28388547
hsa-miR-195-5p	ADAMTS5	target	-7.895415794	1.28388547
hsa-miR-181d-5p	ADAMTS5	target	-2.538257378	1.28388547
hsa-miR-181c-5p	ADAMTS5	target	-5.975134372	1.28388547
hsa-miR-181b-5p	ADAMTS5	target	-4.229699617	1.28388547
hsa-miR-181a-5p	ADAMTS5	target	-8.086162172	1.28388547
hsa-miR-15b-5p	ADAMTS5	target	-7.4728635	1.28388547
hsa-miR-15a-5p	ADAMTS5	target	-7.474686522	1.28388547
hsa-miR-152-3p	ADAMTS5	target	-5.366644456	1.28388547
hsa-miR-148b-3p	ADAMTS5	target	-3.844370844	1.28388547
hsa-miR-148a-3p	ADAMTS5	target	-6.9178194	1.28388547
hsa-miR-140-5p	ADAMTS5	target	-7.6651035	1.28388547
hsa-let-7i-5p	ADAMTS5	target	-10.44359602	1.28388547
hsa-let-7g-5p	ADAMTS5	target	-9.970715511	1.28388547
hsa-let-7f-5p	ADAMTS5	target	-12.84678967	1.28388547
hsa-let-7e-5p	ADAMTS5	target	-8.750119272	1.28388547
hsa-let-7c-5p	ADAMTS5	target	-13.21630578	1.28388547

hsa-let-7b-5p	ADAMTS5	target	-25.01169358	1.28388547
hsa-let-7a-5p	ADAMTS5	target	-13.68176841	1.28388547
hsa-miR-92a-3p	ACTC1	target	2.653037772	-1.459980616

Supplementary Table 5. Patients' infomation

Case Number	Gender	Age (Years)	Diagnosis	MRI Grade	Segment	Operation Method
1	Male	63	Lumbar Spinal Stenosis	IV	L5/S1	Transforaminal lumbar interbody fusion
2	Male	78	Lumbar disc herniation	III	L5/S1	Percutaneous lumbar intervertebral foramen microscopic resection
3	Male	85	Lumbar Spinal Stenosis	IV	L5/S1	Transforaminal lumbar interbody fusion
4	Male	61	Lumbar disc herniation	III	L4/5	Percutaneous lumbar intervertebral foramen microscopic resection
5	Male	75	Lumbar disc herniation	III	L4/5	Percutaneous lumbar intervertebral foramen microscopic resection
6	Male	87	Lumbar disc herniation	III	L5/S1	Percutaneous lumbar intervertebral foramen microscopic resection
7	Male	49	Lumbar Spinal Stenosis	IV	L4/5	Transforaminal lumbar interbody fusion
8	Female	52	Lumbar Spinal Stenosis	III	L4/5	Transforaminal lumbar interbody fusion
9	Male	83	Lumbar disc herniation	IV	L5/S1	Percutaneous lumbar intervertebral foramen microscopic resection
10	Male	62	Lumbar Spinal Stenosis	III	L4/5	Transforaminal lumbar interbody fusion
11	Female	86	Lumbar Spinal Stenosis	IV	L4/5	Transforaminal lumbar interbody fusion
12	Male	53	Lumbar Spinal Stenosis	IV	L4/5	Transforaminal lumbar interbody fusion
13	Female	57	Lumbar Spinal Stenosis	IV	L4/5	Transforaminal lumbar interbody fusion
14	Male	81	Lumbar disc herniation	IV	L5/S1	Percutaneous lumbar intervertebral foramen microscopic resection
15	Female	49	Lumbar Spinal Stenosis	IV	L5/S1	Transforaminal lumbar interbody fusion
16	Female	54	Lumbar Spinal Stenosis	IV	L4/5	Transforaminal lumbar interbody fusion
17	Female	63	Lumbar Spinal Stenosis	IV	L4/5	Transforaminal lumbar interbody fusion
18	Male	49	Lumbar disc herniation	III	L5/S1	Percutaneous lumbar intervertebral foramen microscopic resection
19	Male	66	Lumbar disc herniation	III	L5/S1	Percutaneous lumbar intervertebral foramen microscopic resection
20	Female	49	Lumbar Spinal Stenosis	IV	L5/S1	Transforaminal lumbar interbody fusion
21	Female	73	Lumbar Spinal Stenosis	V	L5/S1	Transforaminal lumbar interbody fusion
22	Female	69	Lumbar Spinal Stenosis	V	L4/5	Transforaminal lumbar interbody fusion
23	Male	64	Lumbar Spinal Stenosis	V	L5/S1	Transforaminal lumbar interbody fusion
24	Male	59	Lumbar Spinal Stenosis	IV	L4/5	Transforaminal lumbar interbody fusion
25	Male	82	Lumbar Spinal Stenosis	III	L5/S1	Transforaminal lumbar interbody fusion
26	Male	83	Lumbar disc herniation	III	L5/S1	Percutaneous lumbar intervertebral foramen microscopic resection
27	Male	63	Lumbar disc herniation	IV	L4/5	Percutaneous lumbar intervertebral foramen microscopic resection
28	Male	63	Lumbar Spinal Stenosis	IV	L4/5	Transforaminal lumbar interbody fusion
29	Male	68	Lumbar Spinal Stenosis	IV	L5/S1	Transforaminal lumbar interbody fusion
30	Female	67	Lumbar disc herniation	III	L5/S1	Percutaneous lumbar intervertebral foramen microscopic resection
31	Male	69	Lumbar Spinal Stenosis	III	L4/5	Transforaminal lumbar interbody fusion
32	Female	69	Lumbar Spinal Stenosis	III	L5/S1	Transforaminal lumbar interbody fusion
33	Male	73	Lumbar disc herniation	IV	L5/S1	Percutaneous lumbar intervertebral foramen microscopic resection
34	Male	25	Lumbar disc herniation	II	L5/S1	Percutaneous lumbar intervertebral foramen microscopic resection
35	Male	32	Lumbar disc herniation	II	L4/5	Percutaneous lumbar intervertebral foramen microscopic resection
36	Female	36	Lumbar disc herniation	II	L4/5	Percutaneous lumbar intervertebral foramen microscopic resection
37	Female	34	Lumbar disc herniation	II	L4/5	Percutaneous lumbar intervertebral foramen microscopic resection
38	Female	27	Lumbar disc herniation	I	L5/S1	Percutaneous lumbar intervertebral foramen microscopic resection
39	Male	42	Lumbar disc herniation	II	L4/5	Percutaneous lumbar intervertebral foramen microscopic resection

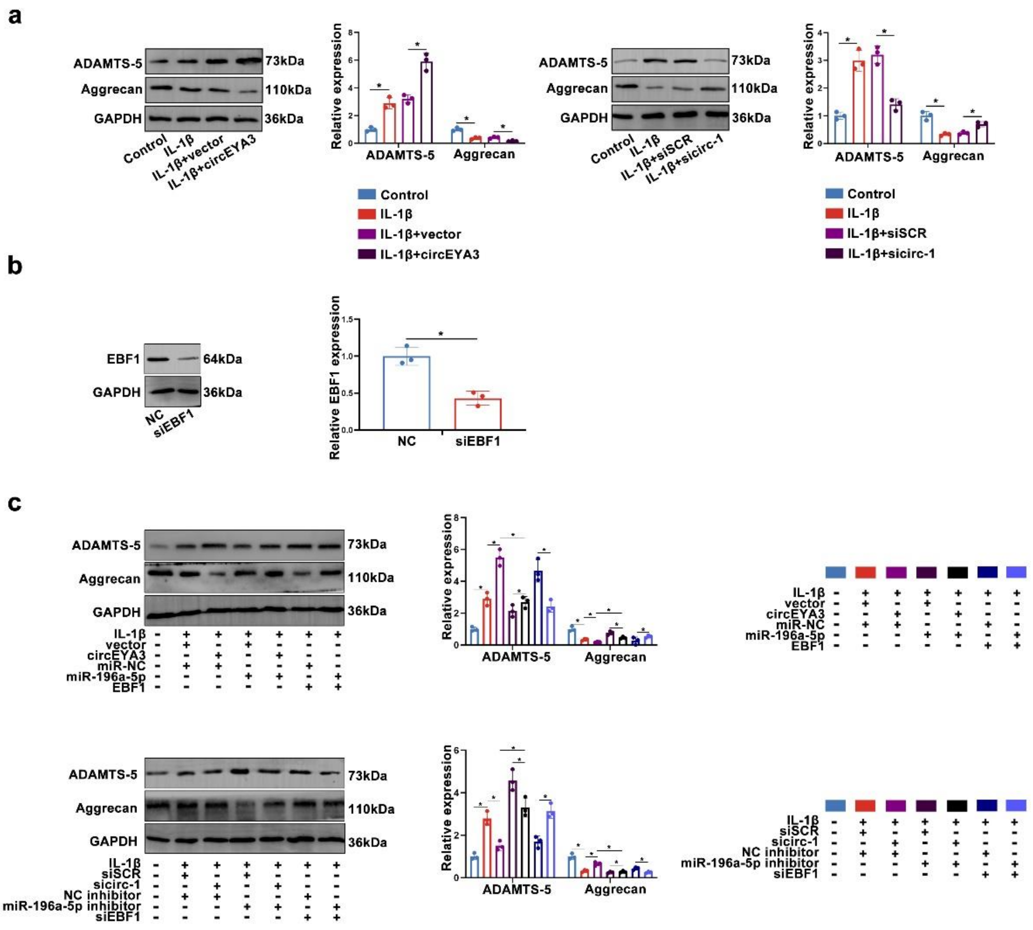

Supplementary Figure 1. CircEYA3/mir-196a-5p/EBF1 axis mediates ECM degradation of NPC.

(a) Expression levels of ADAMTS-5 and Aggrecan in treated NPCs. (b) Expression level of EBF1 in treated NPCs. (c) Expression levels of ADAMTS-5 and Aggrecan in treated NPCs. Data were means ± SD of three independent assays (*P<0.05).

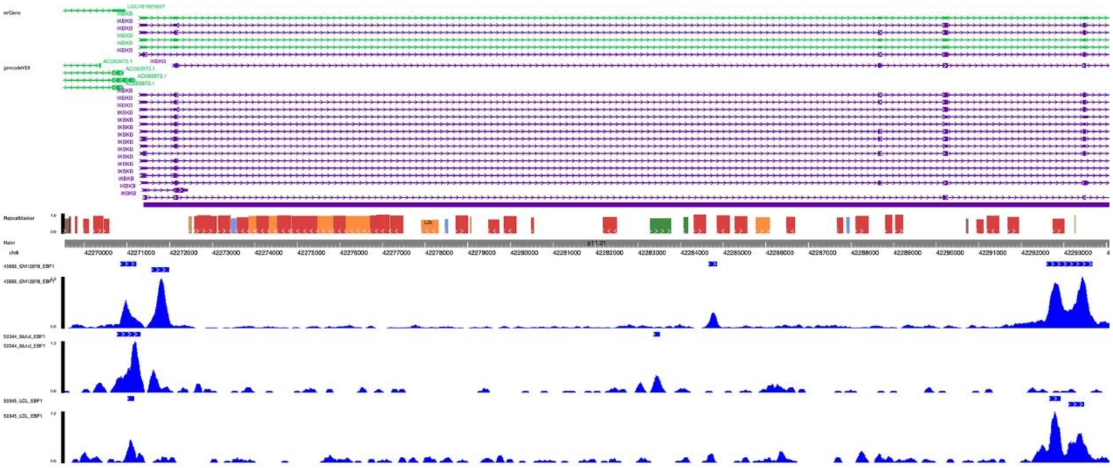

Supplementary Figure 2. The ChIP-seq data of EBF1 from Cistrome Data Browser.

Supplementary Figure 3. Unedited and uncropped Western blots of all the indicated figures in main figures and supplementary figures.

Figure 1h

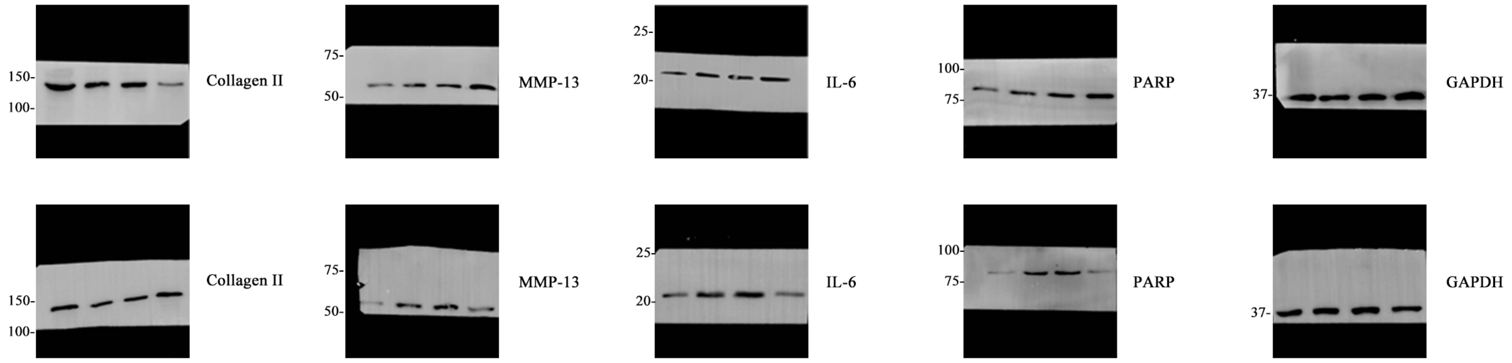

Figure 3k

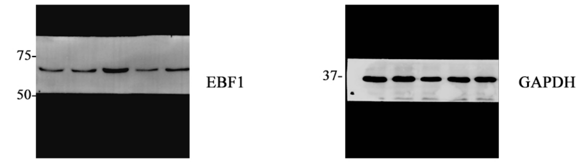

Figure 3l

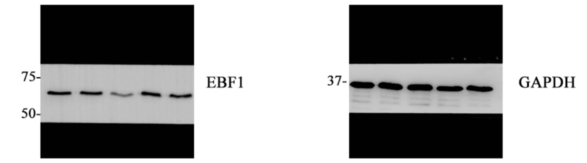

Figure 4a

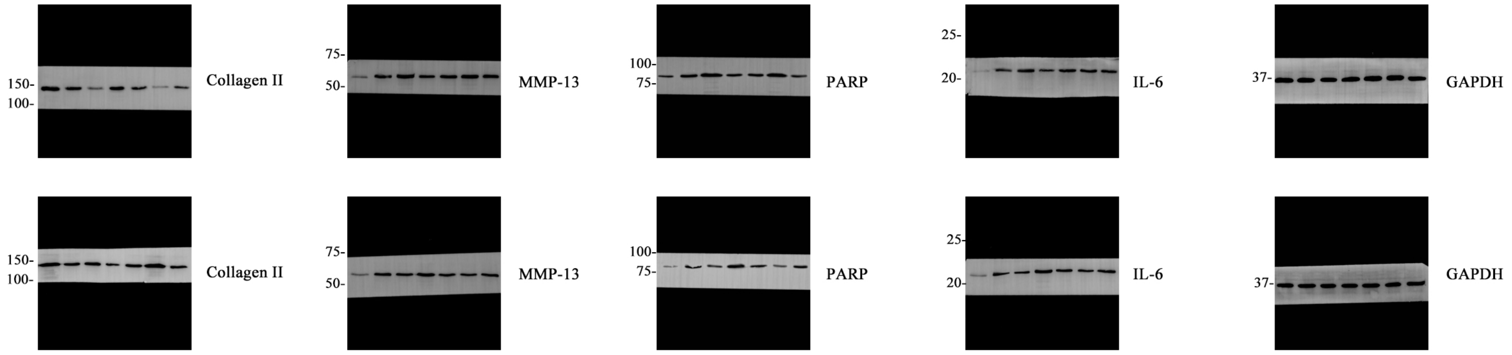

Figure 6a

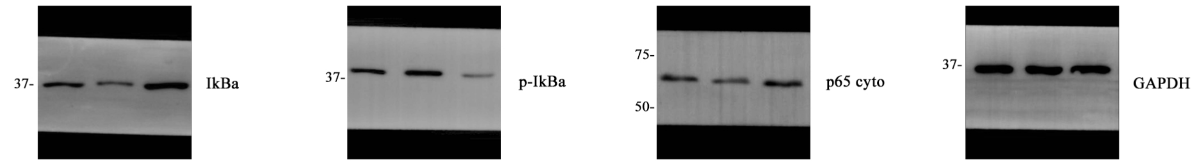

Figure 6b

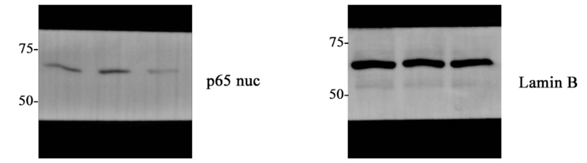

Figure 6i

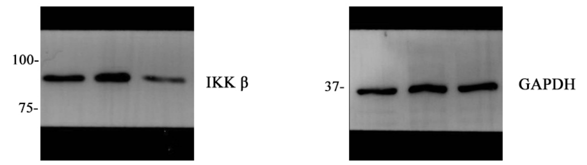

Figure 6b

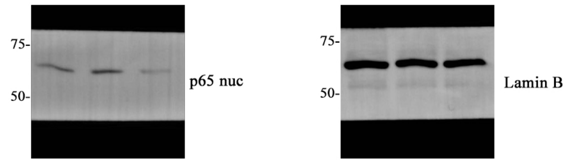

Figure 6i

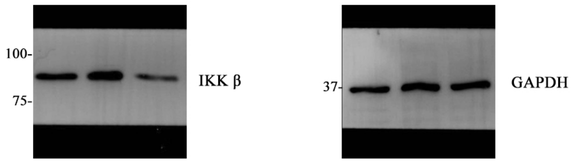

Figure 6j

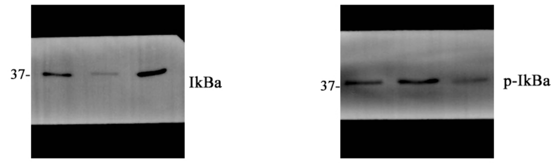

Figure 6k

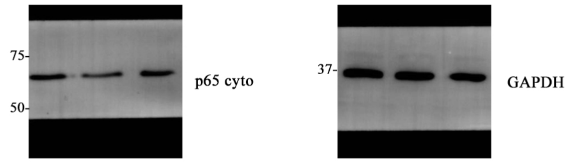

Figure 6l

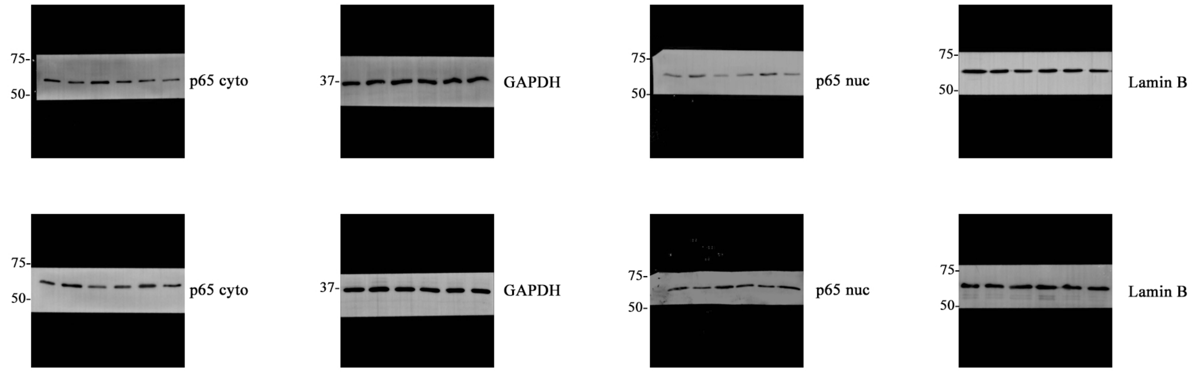

Supplementary Figure 1a

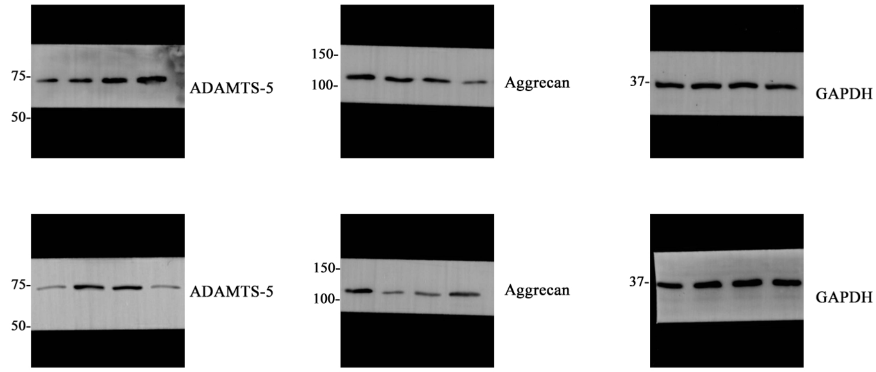

Supplementary Figure 1b

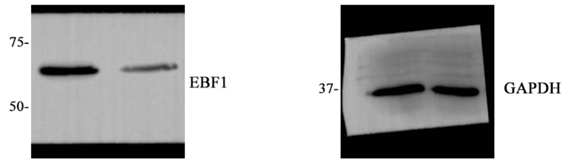

Supplementary Figure 1c

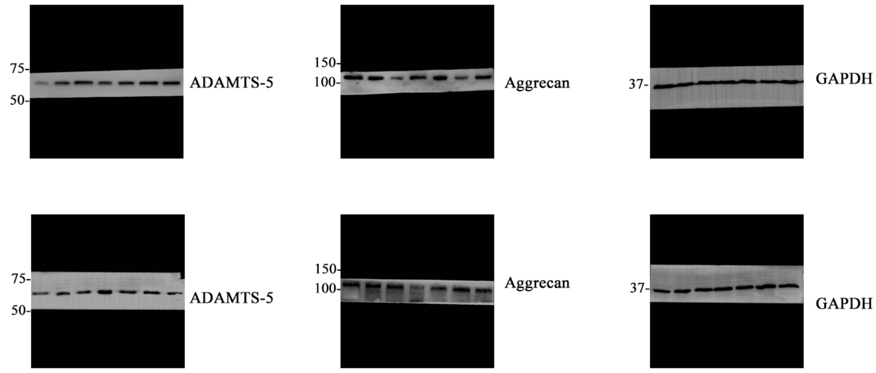

Supplement: Supplementary file 1 — Supplementary Information [file 42003_2024_6055_MOESM1_ESM.pdf]
