# Supplementary material for: CircEYA3 aggravates intervertebral disc degeneration through the miR-196a-5p/EBF1 axis and NF-κB signaling
Source: Commun Biol. 2024 Mar 30;7:390. doi: 10.1038/s42003-024-06055-2 (PMC10981674; doi:10.1038/s42003-024-06055-2)
Supplement: Supplementary file 2 — Description of Supplementary Materials [file 42003_2024_6055_MOESM2_ESM.pdf]

Description of Additional Supplementary Files

File name: Supplementary Data 1

Description: Source data for graphs

File name: Supplementary Data 2

Description: The gene expression matrix of circRNAs

File name: Supplementary Data 3

Description: The gene expression matrix of miRNAs

File name: Supplementary Data 4

Description: The gene expression matrix of mRNAs

File name: Supplementary Data 5

Description: The regulatory network
