# Supplementary material for: CircEYA3 aggravates intervertebral disc degeneration through the miR-196a-5p/EBF1 axis and NF-κB signaling
Source: Commun Biol. 2024 Mar 30;7:390. doi: 10.1038/s42003-024-06055-2 (PMC10981674; doi:10.1038/s42003-024-06055-2)
Supplement: Supplementary file 4 — Supplementary Data 2 [file 42003_2024_6055_MOESM4_ESM.pdf]

Supplementary data 2. The gene expression matrix of circRNAs

id	logFC	AveExpr	t	P.Value	B
hsa_circ_000178	2.45118972	8.59736988	210.4181487	3.82E-19	34.66045582
hsa_circ_100646	2.04021004	12.46600778	192.5930067	9.04E-19	33.85086049
hsa_circ_000094	-1.7381442	12.953486	-183.5410947	1.44E-18	33.40203875
hsa_circ_100790	-1.6490793	13.24564825	-174.1361965	2.41E-18	32.9053814
hsa_circ_100018	2.57126848	12.80710734	163.8254308	4.37E-18	32.32154013
hsa_circ_001175	2.28857146	10.54738051	154.2115299	7.87E-18	31.7357697
hsa_circ_100319	-2.1792524	11.88554452	-151.7916319	9.18E-18	31.58146084
hsa_circ_000864	1.77543696	9.40277206	146.1514128	1.33E-17	31.21027961
hsa_circ_100227	1.87763952	8.8926687	140.3665111	1.97E-17	30.81179723
hsa_circ_100723	-1.88863978	7.43045043	-133.0191002	3.32E-17	30.27746386
hsa_circ_100604	2.67199762	11.74985539	132.2309533	3.52E-17	30.21814779
hsa_circ_100273	-2.08731664	11.5244404	-131.5268205	3.71E-17	30.16481318
hsa_circ_100236	1.52880272	13.74598334	129.0608938	4.46E-17	29.97544185
hsa_circ_100772	-2.6984196	8.59185784	-128.9869215	4.48E-17	29.9696979
hsa_circ_100684	-2.38677888	10.03441654	-127.4334222	5.04E-17	29.84820057
hsa_circ_100422	1.57102538	8.29801329	127.136405	5.16E-17	29.82478066
hsa_circ_100876	1.91686344	10.43978712	122.1288938	7.63E-17	29.42040238
hsa_circ_000750	-2.3494982	10.72759014	-120.6968944	8.56E-17	29.30133577
hsa_circ_000791	1.116836	13.7170995	117.933427	1.07E-16	29.06704143
hsa_circ_100637	-2.13558324	8.05461754	-117.6851329	1.09E-16	29.04569185
hsa_circ_100329	1.45547144	8.41234392	112.6693855	1.67E-16	28.60339903
hsa_circ_000881	2.02632116	10.88736208	108.4964546	2.41E-16	28.21855807
hsa_circ_100272	-1.54717282	12.08083349	-106.6081275	2.86E-16	28.03904529
hsa_circ_100420	-1.65018748	11.61666386	-102.2025193	4.32E-16	27.60639162
hsa_circ_100085	2.0592209	10.59952591	99.89306154	5.40E-16	27.37143043
hsa_circ_000046	1.51798832	8.17655924	92.23706746	1.17E-15	26.54846585
hsa_circ_100845	1.3355756	11.24255804	90.44652385	1.42E-15	26.34542802
hsa_circ_000911	1.55309534	11.21024543	90.23938803	1.45E-15	26.3216638
hsa_circ_100086	2.23862012	8.21801448	89.1693113	1.63E-15	26.197964
hsa_circ_001067	1.0692433	12.45337159	88.12755986	1.83E-15	26.07601248
hsa_circ_001405	1.49576896	8.3511731	87.83149766	1.89E-15	26.04107468
hsa_circ_100754	-1.17357174	9.09261267	-87.42277378	1.97E-15	25.99263589
hsa_circ_001401	-1.55259906	12.52729707	-85.70545865	2.40E-15	25.78645498
hsa_circ_100035	-1.51137716	7.79545584	-85.45428237	2.46E-15	25.75593235
hsa_circ_001108	1.31896692	11.17098522	84.76850307	2.67E-15	25.67211153
hsa_circ_100034	-1.56709236	8.60465648	-84.32397158	2.81E-15	25.61739387
hsa_circ_100040	1.71433716	6.48578432	83.75766369	3.00E-15	25.54724361
hsa_circ_100051	-1.39212314	9.14940261	-83.03265128	3.26E-15	25.45669976
hsa_circ_100525	1.41262602	8.20413407	82.74972209	3.37E-15	25.42113944
hsa_circ_001046	1.3837194	9.5915602	81.32879787	3.99E-15	25.24058936
hsa_circ_100411	1.31800384	10.47217824	80.32553088	4.50E-15	25.11109793
hsa_circ_100438	0.93936936	11.81524342	78.47537619	5.65E-15	24.86778096
hsa_circ_000963	1.31472582	7.81189843	78.33765785	5.74E-15	24.84942934
hsa_circ_100205	1.04341994	9.46955699	77.83477147	6.11E-15	24.78212899
hsa_circ_100823	-1.22774796	6.1726551	-77.58610534	6.31E-15	24.74868197
hsa_circ_000200	2.48946402	7.60727927	77.14830412	6.66E-15	24.68952151
hsa_circ_100836	-1.42659808	9.12767482	-76.51995228	7.22E-15	24.60399493
hsa_circ_000638	1.38585508	7.68966904	76.07600929	7.64E-15	24.54312477
hsa_circ_000941	-2.26248584	8.46632492	-75.39183335	8.34E-15	24.44858502
hsa_circ_001583	-1.1081408	8.59382118	-75.08566176	8.67E-15	24.40598749
hsa_circ_000662	1.4290372	8.14743368	74.4377413	9.44E-15	24.31524174

hsa_circ_100192	-1.1517077	12.22282515	-74.29585344	9.61E-15	24.29525939
hsa_circ_000671	1.26898056	8.6138709	74.24561041	9.68E-15	24.28817402
hsa_circ_100290	-2.1012706	8.23330774	-73.75221483	1.03E-14	24.21832771
hsa_circ_100508	1.59004516	13.02753552	73.67865767	1.04E-14	24.20787307
hsa_circ_000166	2.16922376	6.96415	72.17072167	1.27E-14	23.99112235
hsa_circ_100640	-1.50197352	8.64699864	-72.07016805	1.29E-14	23.97650157
hsa_circ_100571	2.00809222	8.90012861	72.02133645	1.30E-14	23.96939366
hsa_circ_100427	-2.84656613	7.073818815	-71.81402707	1.34E-14	23.93916189
hsa_circ_001153	1.2897528	9.44752942	71.63806006	1.37E-14	23.91342949
hsa_circ_100045	1.2146054	9.26524766	71.3894239	1.42E-14	23.87695816
hsa_circ_100606	1.58940508	7.2679467	71.18468306	1.46E-14	23.84682642
hsa_circ_001654	2.12861288	9.10859654	70.79894459	1.54E-14	23.78981188
hsa_circ_100476	-2.162316	8.724251	-68.69545266	2.06E-14	23.47312018
hsa_circ_100844	-2.38715834	9.97152331	-67.8825809	2.31E-14	23.34803791
hsa_circ_100593	-0.87421242	10.88284529	-67.59979312	2.41E-14	23.30415965
hsa_circ_001059	-1.46923822	11.27169815	-67.50968828	2.44E-14	23.2901388
hsa_circ_100579	-1.60890002	8.12827113	-67.02744098	2.62E-14	23.21476784
hsa_circ_100202	1.63754254	11.37879823	66.01306643	3.03E-14	23.05438444
hsa_circ_100117	1.66873586	8.17857433	65.53492658	3.26E-14	22.9779009
hsa_circ_001950	1.29487692	6.8385217	65.52567083	3.26E-14	22.97641467
hsa_circ_100412	0.80484324	9.19605542	65.4807503	3.28E-14	22.96919854
hsa_circ_002086	-2.11371662	10.77186431	-65.25282955	3.40E-14	22.93250598
hsa_circ_100213	-1.63603092	10.98319524	-65.06382836	3.49E-14	22.90197863
hsa_circ_000780	1.8686227	8.07333315	65.02219815	3.51E-14	22.89524225
hsa_circ_100257	-1.52222976	10.24247028	-64.48764704	3.81E-14	22.80834687
hsa_circ_001754	-1.5191623	8.42620851	-63.50149737	4.42E-14	22.64607571
hsa_circ_001409	1.72689272	7.54703046	63.10816477	4.70E-14	22.58062781
hsa_circ_100188	-1.29563954	7.11243081	-62.91629382	4.84E-14	22.54854933
hsa_circ_001264	1.13788016	7.33494424	62.66435835	5.03E-14	22.50627566
hsa_circ_100004	-1.17333056	8.82267152	-62.59193554	5.09E-14	22.49409109
hsa_circ_000684	1.38981146	9.18909817	62.16301828	5.44E-14	22.42163069
hsa_circ_100270	-1.4706532	7.93125118	-61.87529073	5.69E-14	22.37273407
hsa_circ_000598	1.30106672	9.91299204	61.49512994	6.04E-14	22.30776991
hsa_circ_001302	-0.75481284	11.33194536	-61.47157468	6.07E-14	22.30373108
hsa_circ_100226	1.5606671	7.48159913	61.20879142	6.32E-14	22.25856579
hsa_circ_100090	1.22659068	11.21526448	60.81160529	6.74E-14	22.18992115
hsa_circ_100882	1.4391607	7.80225149	60.01128241	7.66E-14	22.05019534
hsa_circ_100616	-1.43464798	7.48413809	-59.95327526	7.74E-14	22.03999391
hsa_circ_000711	1.51091378	7.46078127	59.69577405	8.07E-14	21.99458599
hsa_circ_001100	1.2243097	10.37800627	59.06922863	8.94E-14	21.88325703
hsa_circ_001594	-0.66963986	11.07497123	-59.03620387	8.99E-14	21.87735545
hsa_circ_100219	-1.2632643	7.72023849	-58.05215228	1.06E-13	21.69993591
hsa_circ_001216	1.00565562	9.55740661	57.67803792	1.13E-13	21.63167652
hsa_circ_100332	-1.285155	8.52189858	-56.97614971	1.27E-13	21.50238299
hsa_circ_100685	1.04794418	11.35396649	56.81673221	1.30E-13	21.47279024
hsa_circ_000956	-1.06885542	13.05191179	-56.03682615	1.49E-13	21.32678305
hsa_circ_100701	-1.98306782	5.71951711	-55.28857098	1.70E-13	21.18473897
hsa_circ_100893	-1.0679431	8.33520623	-55.27461726	1.70E-13	21.1820715
hsa_circ_100477	-1.18776898	8.95398659	-54.38097928	2.00E-13	21.00979364
hsa_circ_100283	-1.17878776	8.43381654	-54.27670228	2.03E-13	20.9895032
hsa_circ_100108	1.12849542	10.86209037	54.18493723	2.07E-13	20.97161448
hsa_circ_100851	0.85000344	9.16463826	54.10139053	2.10E-13	20.95530101
hsa_circ_100223	1.10901524	13.03183188	53.90749636	2.17E-13	20.91734185

hsa_circ_100883	1.92324206	7.38746705	53.7404466	2.24E-13	20.88452647
hsa_circ_100698	-2.41655144	6.2012452	-53.30967221	2.42E-13	20.79942349
hsa_circ_100528	-0.7105696	7.90069864	-53.03479294	2.55E-13	20.74475243
hsa_circ_000872	-0.8051739	11.26231555	-52.97584426	2.57E-13	20.73299053
hsa_circ_100033	-1.31053668	9.61702286	-52.84736307	2.63E-13	20.70730879
hsa_circ_100084	-0.98354126	9.02917599	-52.10134921	3.02E-13	20.55692454
hsa_circ_100620	-1.06034624	10.2168556	-51.98545356	3.09E-13	20.53336567
hsa_circ_100533	-1.11518278	11.11426209	-51.69488197	3.26E-13	20.47406394
hsa_circ_100735	-1.04504212	7.04980538	-51.47715445	3.40E-13	20.42940645
hsa_circ_100770	-1.7176014	10.17852398	-50.52064484	4.08E-13	20.23092136
hsa_circ_001012	-0.7409334	9.10897098	-49.92955737	4.57E-13	20.10635044
hsa_circ_100806	-0.71952262	9.60782749	-49.85531363	4.64E-13	20.0905981
hsa_circ_100395	0.86120276	9.77763214	49.42176957	5.05E-13	19.99813552
hsa_circ_100498	-0.7112877	9.45742225	-49.29229239	5.18E-13	19.97036235
hsa_circ_100374	2.11558444	10.1379365	49.24763334	5.23E-13	19.96076574
hsa_circ_001653	2.22151978	11.63910189	48.96166554	5.53E-13	19.89910558
hsa_circ_100079	-2.28266352	10.4807411	-48.62993584	5.91E-13	19.82711991
hsa_circ_100180	-1.09638402	7.08563063	-48.2786728	6.34E-13	19.75035183
hsa_circ_100464	-1.0795639	7.14144717	-48.03284585	6.66E-13	19.69628986
hsa_circ_001040	1.61164237	6.869397575	47.67944253	7.15E-13	19.61807762
hsa_circ_100802	1.62299234	7.08581567	47.17480129	7.93E-13	19.50537295
hsa_circ_100269	-0.87123226	8.89183747	-47.13427364	8.00E-13	19.49626887
hsa_circ_100478	-0.93538688	8.24800646	-46.78231314	8.60E-13	19.41687086
hsa_circ_100879	-0.8122987	8.36382225	-46.61010998	8.91E-13	19.37780388
hsa_circ_001363	1.12083162	6.37814885	46.60304922	8.93E-13	19.37619893
hsa_circ_000950	-1.50967756	7.92760874	-45.92747396	1.03E-12	19.2214905
hsa_circ_001072	-1.28896542	6.82326711	-45.79587506	1.06E-12	19.19108696
hsa_circ_100350	1.10611924	6.83032784	45.62916588	1.10E-12	19.15244506
hsa_circ_001729	-1.68015529	5.747324995	-45.58781315	1.11E-12	19.14283778
hsa_circ_000585	-0.67000574	7.65530993	-45.36962447	1.16E-12	19.09200111
hsa_circ_000481	-0.84648114	11.42880931	-44.92206734	1.27E-12	18.98694724
hsa_circ_001389	1.49677202	7.77453993	44.91403163	1.28E-12	18.98505141
hsa_circ_100433	-1.89530152	13.66628122	-44.8550516	1.29E-12	18.97112608
hsa_circ_100445	-0.79149278	10.97211551	-44.76814816	1.32E-12	18.95057432
hsa_circ_000943	-2.56591412	10.16033372	-44.64273037	1.35E-12	18.92084334
hsa_circ_001225	1.11023206	8.24813391	44.4630894	1.41E-12	18.87811159
hsa_circ_000676	-1.27696896	13.60724394	-43.7426003	1.65E-12	18.70496205
hsa_circ_100892	-1.00594502	7.96127781	-43.6781013	1.67E-12	18.68932178
hsa_circ_100453	1.09279654	8.62362099	43.35857055	1.80E-12	18.61149493
hsa_circ_100351	-0.80997772	9.54775768	-43.23202418	1.85E-12	18.58051294
hsa_circ_100688	1.02484966	6.35771087	42.7581118	2.06E-12	18.46367062
hsa_circ_100817	-1.2147678	6.97935174	-42.62096045	2.12E-12	18.42961325
hsa_circ_001296	-0.9259799	7.30513705	-42.5357296	2.16E-12	18.40839323
hsa_circ_100668	1.59573632	7.19926162	42.5300019	2.17E-12	18.40696567
hsa_circ_100175	0.97280588	7.69686286	42.074401	2.41E-12	18.29278929
hsa_circ_100230	1.46204079	6.785059205	41.97427209	2.46E-12	18.26752998
hsa_circ_100530	-0.64283744	6.71612182	-41.90561381	2.50E-12	18.25017471
hsa_circ_001396	-1.23204966	9.18653341	-41.89593369	2.51E-12	18.2477255
hsa_circ_100037	-1.43572602	6.80883501	-41.68074339	2.64E-12	18.19313201
hsa_circ_100808	-1.07095042	10.23786301	-41.40383938	2.81E-12	18.12246415
hsa_circ_000645	-0.72546028	9.81833956	-41.36853015	2.83E-12	18.11341891
hsa_circ_001724	1.05159374	6.89077349	41.08110141	3.03E-12	18.03949828
hsa_circ_100449	-0.79624654	8.07985223	-40.97256885	3.11E-12	18.01145098

hsa_circ_100759	0.91637092	9.31109046	40.68855916	3.33E-12	17.93770238
hsa_circ_100322	-0.6841298	6.45340102	-40.66742202	3.34E-12	17.93219309
hsa_circ_100629	-0.77896462	6.33530601	-40.10203453	3.83E-12	17.78375298
hsa_circ_002106	-1.03718066	7.34481851	-39.92251928	4.00E-12	17.73618318
hsa_circ_001304	-1.30897735	6.131369165	-39.47451575	4.46E-12	17.61652599
hsa_circ_100696	0.61790562	13.79506561	39.36914997	4.58E-12	17.58818643
hsa_circ_002143	0.84933926	6.32632009	39.36342915	4.59E-12	17.58664557
hsa_circ_100361	-0.96254472	7.60775968	-39.08417137	4.91E-12	17.51115566
hsa_circ_001240	1.60280134	6.82447159	38.83549904	5.23E-12	17.44347815
hsa_circ_100896	-1.27638136	9.93404124	-38.21075326	6.12E-12	17.27151983
hsa_circ_100750	0.98513102	11.39696051	37.8583011	6.69E-12	17.17326514
hsa_circ_100266	-1.0576901	6.27543595	-37.74365135	6.89E-12	17.14110667
hsa_circ_000868	-0.88781754	7.61136199	-37.69528914	6.98E-12	17.12751213
hsa_circ_100229	1.15445158	7.48051373	37.48722645	7.36E-12	17.06882678
hsa_circ_100861	0.96008134	10.80916689	37.37052938	7.59E-12	17.03576916
hsa_circ_100745	-1.27463816	5.9804166	-36.60349799	9.27E-12	16.8158891
hsa_circ_001038	-0.95127656	11.77434638	-36.17401383	1.04E-11	16.69075627
hsa_circ_100373	1.03950884	10.8357246	35.92557533	1.11E-11	16.61769457
hsa_circ_001486	0.96611814	5.84277937	35.31704274	1.31E-11	16.43658769
hsa_circ_100655	-0.96976206	9.05027879	-35.29846297	1.32E-11	16.43100938
hsa_circ_100714	0.99051948	8.53406486	35.18778023	1.36E-11	16.39771776
hsa_circ_100531	-0.9136769	6.20223051	-34.84672893	1.49E-11	16.29447594
hsa_circ_100421	1.50703614	7.74471713	34.84010077	1.49E-11	16.29245955
hsa_circ_100499	0.91380882	6.76935527	34.78997407	1.52E-11	16.27719783
hsa_circ_100832	0.91138036	7.71574328	34.34278786	1.72E-11	16.14007072
hsa_circ_100439	-0.74705484	7.10046274	-33.95379188	1.92E-11	16.01933648
hsa_circ_100072	1.10010334	7.13679171	33.73913529	2.04E-11	15.9521231
hsa_circ_100389	-0.80034552	7.35457118	-33.15007029	2.42E-11	15.76547096
hsa_circ_100850	0.60739534	9.64973967	32.65117764	2.80E-11	15.60479981
hsa_circ_100849	0.76020616	9.16600224	32.23711483	3.17E-11	15.46959004
hsa_circ_000441	-1.01660484	6.36911402	-31.7807893	3.63E-11	15.31857302
hsa_circ_000629	0.90864142	14.61068189	31.76093181	3.66E-11	15.31195272
hsa_circ_100179	0.82226674	5.67462505	31.69855071	3.73E-11	15.29112878
hsa_circ_100641	0.81211784	8.8032765	31.67538751	3.75E-11	15.28338616
hsa_circ_100751	1.4488197	12.03660011	31.48048738	3.98E-11	15.21801537
hsa_circ_100633	-1.81514382	13.62869961	-31.40694093	4.07E-11	15.19324333
hsa_circ_001769	-1.1074031	6.34114505	-31.30095661	4.21E-11	15.15744443
hsa_circ_100881	0.82006118	8.36321503	31.0627871	4.53E-11	15.07655742
hsa_circ_100147	0.78611994	6.36882247	30.86413615	4.82E-11	15.0086215
hsa_circ_100069	0.72292428	9.5912844	30.78403409	4.94E-11	14.98110538
hsa_circ_000996	0.9079796	8.9016227	30.67200872	5.12E-11	14.94250435
hsa_circ_100815	1.06539044	6.17557174	30.50006258	5.40E-11	14.88298448
hsa_circ_100365	-0.73870582	6.51041951	-30.48475467	5.43E-11	14.87766954
hsa_circ_001350	1.61125082	8.62815033	30.36537118	5.64E-11	14.83612865
hsa_circ_100017	-0.6813063	6.87419521	-29.84473949	6.66E-11	14.65306458
hsa_circ_001800	0.80421008	6.22699784	29.76117216	6.85E-11	14.62338767
hsa_circ_100407	-1.40093756	7.62629172	-29.36632332	7.79E-11	14.48204656
hsa_circ_100827	1.2179772	6.7771812	29.23298446	8.14E-11	14.43389303
hsa_circ_100647	1.10998246	5.43012173	28.82456004	9.32E-11	14.28503886
hsa_circ_100053	0.6387845	12.18317417	28.75490426	9.54E-11	14.25944512
hsa_circ_100413	0.9589644	5.86815702	28.65557902	9.86E-11	14.22284418
hsa_circ_000926	1.08593422	6.95932569	28.57769283	1.01E-10	14.19405606
hsa_circ_100891	0.84088412	7.17401674	28.54980462	1.02E-10	14.18372931

hsa_circ_100443	-1.01819448	6.5540979	-28.35673492	1.09E-10	14.1119642
hsa_circ_002172	0.9018417	5.85458213	28.08308524	1.20E-10	14.00941997
hsa_circ_100008	0.81826162	6.53118621	27.75185872	1.34E-10	13.88397891
hsa_circ_100693	0.9472488	6.61445094	27.34539106	1.55E-10	13.72801903
hsa_circ_000162	0.65161268	5.97917774	27.32836601	1.56E-10	13.72143708
hsa_circ_100870	-0.80497202	5.22673171	-27.19735163	1.63E-10	13.67065144
hsa_circ_100748	1.128857	7.14759948	27.13247864	1.67E-10	13.64541561
hsa_circ_001830	-0.70968266	7.51574129	-27.05192947	1.72E-10	13.61399926
hsa_circ_100302	0.80074496	6.42507744	26.78126215	1.89E-10	13.50775608
hsa_circ_100840	-0.60286564	8.1987215	-26.0941828	2.43E-10	13.23326733
hsa_circ_001915	-0.85255006	8.17878085	-25.97308774	2.54E-10	13.18415802
hsa_circ_100600	1.03440484	6.24393942	25.95151052	2.56E-10	13.17538404
hsa_circ_001838	0.74915307	5.481824815	25.18806056	3.41E-10	12.86026526
hsa_circ_100821	-0.87033672	12.11807786	-24.53762763	4.39E-10	12.58435737
hsa_circ_000031	1.02720825	5.913059335	24.44283212	4.55E-10	12.54355245
hsa_circ_100403	-0.6961789	8.66224683	-24.42720819	4.58E-10	12.53681232
hsa_circ_100112	0.68063574	7.95760401	24.41105903	4.61E-10	12.52984119
hsa_circ_100146	-0.67087006	7.47907429	-24.06074013	5.29E-10	12.37750428
hsa_circ_001109	1.1582763	9.40980431	23.98255835	5.46E-10	12.34321257
hsa_circ_100695	-0.8700856	5.86082794	-23.81160524	5.85E-10	12.26785001
hsa_circ_100300	0.84753485	5.854050535	23.72825807	6.05E-10	12.2309167
hsa_circ_100583	-0.81343056	6.2641466	-23.66219346	6.21E-10	12.20155215
hsa_circ_100005	-0.68682784	6.85489034	-23.29643477	7.22E-10	12.03752549
hsa_circ_002144	1.73060314	6.80733501	23.24557023	7.37E-10	12.01451744
hsa_circ_100875	-0.86865484	7.63686874	-23.19995316	7.51E-10	11.99384148
hsa_circ_001671	-0.67956872	8.0347035	-23.06751691	7.93E-10	11.93359074
hsa_circ_100075	1.16660692	7.26169276	23.05146009	7.98E-10	11.92626306
hsa_circ_100106	0.61193903	5.368265565	22.92426939	8.42E-10	11.86804314
hsa_circ_100303	-0.8887566	5.5673047	-22.72562384	9.15E-10	11.77648716
hsa_circ_100068	0.67155182	6.71185239	22.64359851	9.47E-10	11.73845551
hsa_circ_100465	-1.1002799	6.21843007	-22.31247623	1.09E-09	11.58356185
hsa_circ_001288	0.60564615	5.817666745	22.00268741	1.25E-09	11.43662402
hsa_circ_100488	-1.01855696	6.78787638	-21.98347478	1.26E-09	11.42744553
hsa_circ_100156	-0.67854831	5.198036555	-21.56184456	1.51E-09	11.22404761
hsa_circ_100331	-1.0498519	6.82781333	-21.53261428	1.53E-09	11.20980499
hsa_circ_000993	1.48485164	8.61267374	21.31842056	1.69E-09	11.10486646
hsa_circ_100409	-0.6386725	6.43627925	-21.14690108	1.82E-09	11.02010115
hsa_circ_100036	-1.16736316	6.01934508	-21.12519601	1.84E-09	11.00932738
hsa_circ_100719	0.75871234	6.90280067	21.08428467	1.87E-09	10.98899121
hsa_circ_002178	-0.91284898	8.79076161	-21.0522065	1.90E-09	10.97301928
hsa_circ_100660	0.64624225	5.841950995	20.44274811	2.52E-09	10.66504312
hsa_circ_001369	0.66946624	5.48605694	20.41641284	2.55E-09	10.65153758
hsa_circ_100298	-0.62373632	5.95373924	-20.3444523	2.64E-09	10.61454874
hsa_circ_100308	0.69777434	6.20698741	20.13907353	2.90E-09	10.50828781
hsa_circ_100470	1.22561234	6.01993435	19.95400459	3.17E-09	10.41164329
hsa_circ_000815	0.99278802	6.15204395	19.27571847	4.41E-09	10.04996631
hsa_circ_100013	-0.7416012	5.52431494	-19.14018209	4.71E-09	9.976243478
hsa_circ_100385	-0.89317763	5.981162035	-19.01666296	5.01E-09	9.908623402
hsa_circ_000987	-0.75775262	11.45966949	-18.88152426	5.36E-09	9.834162272
hsa_circ_100651	0.69972992	6.20615516	18.60574886	6.17E-09	9.68062747
hsa_circ_100868	1.40284112	6.07126454	18.08337528	8.08E-09	9.38379591
hsa_circ_100852	0.92795272	5.81689484	17.98084852	8.53E-09	9.324585674
hsa_circ_000620	-0.73378086	6.48712499	-17.59065659	1.05E-08	9.096293882

hsa_circ_100357	-0.70751866	5.75957177	-17.25647437	1.26E-08	8.896948558
hsa_circ_100627	-0.7724521	5.58501173	-17.15961623	1.33E-08	8.838492747
hsa_circ_100510	-0.60363982	7.69321571	-16.9118349	1.52E-08	8.687531188
hsa_circ_000446	-0.86544474	6.34332729	-16.72850456	1.69E-08	8.574497091
hsa_circ_100907	-0.7392281	5.49979827	-16.71016267	1.71E-08	8.563124521
hsa_circ_100659	0.7302851	6.11220341	16.55297122	1.87E-08	8.465179465
hsa_circ_000942	0.72109792	6.19230612	16.39201099	2.05E-08	8.363982051
hsa_circ_001689	-0.83435386	6.11976023	-16.32740384	2.12E-08	8.323101995
hsa_circ_100547	-2.03177404	6.53483538	-16.28354192	2.18E-08	8.295262455
hsa_circ_100252	-0.94765136	6.3965294	-16.18084303	2.31E-08	8.229804422
hsa_circ_000526	0.81141526	5.53914417	16.12371503	2.39E-08	8.19322473
hsa_circ_100895	0.60409118	5.43765079	15.43053555	3.61E-08	7.739523289
hsa_circ_100830	-0.7019625	5.54994595	-15.27385035	3.98E-08	7.634360181
hsa_circ_100833	0.63787429	5.298796105	14.95646835	4.84E-08	7.418260494
hsa_circ_100552	-0.66703968	5.77454062	-14.51191486	6.42E-08	7.108384808
hsa_circ_100177	1.39822884	10.44189104	14.41549576	6.83E-08	7.040031485
hsa_circ_100612	0.62254288	7.2996452	13.60909536	1.17E-07	6.45149992
hsa_circ_000082	0.97885796	5.87527974	13.13276112	1.63E-07	6.088888695
hsa_circ_100339	0.65794238	5.82622459	13.10120695	1.66E-07	6.064453522
hsa_circ_100115	0.60675094	6.71560895	12.98587698	1.80E-07	5.974693664
hsa_circ_100906	1.03217136	5.97236976	12.13826201	3.36E-07	5.292387251
hsa_circ_100471	0.62319041	5.489965345	11.98588979	3.78E-07	5.165299956
hsa_circ_100136	0.61107388	5.54607096	11.75838414	4.50E-07	4.972894578
hsa_circ_100296	0.68134984	5.38400472	11.4739779	5.63E-07	4.727764055
hsa_circ_100104	0.72846394	5.97685301	11.02908117	8.07E-07	4.333592046
hsa_circ_100566	-0.62153796	6.07614204	-9.658743415	2.66E-06	3.028862271
hsa_circ_100107	1.19228874	6.23301025	8.981766606	5.06E-06	2.327474387
hsa_circ_100526	-0.72797432	5.50609692	-8.950999623	5.21E-06	2.294613505
hsa_circ_100009	-0.61199438	10.08602687	-8.950954113	5.21E-06	2.294564833
hsa_circ_100029	-0.62888442	6.35047651	-7.574803108	2.20E-05	0.72797872
hsa_circ_001459	1.26707614	6.18350239	7.491329699	2.41E-05	0.626423657
hsa_circ_100129	-0.657674	5.98545808	-7.394461898	2.69E-05	0.507568648
hsa_circ_001241	1.04059416	7.64722924	6.689999128	6.16E-05	-0.390491612
hsa_circ_100657	0.61995338	6.13705287	2.632600795	0.02556943	-6.750696087
hsa_circ_000617	0.7498601	5.16097187	2.452510344	0.034703814	-7.054092426
