# Supplementary material for: CircEYA3 aggravates intervertebral disc degeneration through the miR-196a-5p/EBF1 axis and NF-κB signaling
Source: Commun Biol. 2024 Mar 30;7:390. doi: 10.1038/s42003-024-06055-2 (PMC10981674; doi:10.1038/s42003-024-06055-2)
Supplement: Supplementary file 5 — Supplementary Data 3 [file 42003_2024_6055_MOESM5_ESM.pdf]

Supplementary data 3. The gene expression matrix of miRNAs

id	logFC	AveExpr	t	P.Value	B
hsa-miR-32-5p	-1.1363987	1.75372875	-17783.60474	6.87E-18	30.85181771
hsa-miR-3160-3p	2.959943578	3.801899889	4776.571531	1.83E-15	27.04995892
hsa-miR-3155b	2.9598338	3.801954778	4707.370925	1.95E-15	26.98663059
hsa-miR-3922-5p	2.9598338	3.801954778	4707.370925	1.95E-15	26.98663059
hsa-miR-4296	2.9598338	3.801954778	4707.370925	1.95E-15	26.98663059
hsa-miR-3183	3.640370856	4.142113528	2005.235256	7.33E-14	22.90333899
hur_2	-26.97642237	26.56937332	-599.5138063	1.24E-11	16.65301886
hsa-miR-1301-5p	4.3605878	4.502222	573.3838889	1.50E-11	16.41983489
hsa-miR-1915-5p	4.3605878	4.502222	573.3838889	1.50E-11	16.41983489
hsa-miR-1262	4.360478022	4.502276889	573.3132332	1.50E-11	16.41918997
hsa-miR-200a-5p	4.360478022	4.502276889	573.3132332	1.50E-11	16.41918997
hsa-miR-200b-5p	3.628946972	4.193321981	435.0038201	4.84E-11	14.97336256
hsa-miR-2682-3p	5.227993194	4.935924697	383.5861574	8.27E-11	14.31407534
hsa-miR-1261	3.651956789	4.148016272	383.0566215	8.32E-11	14.30683366
hsa-miR-186-3p	3.651956789	4.148016272	383.0566215	8.32E-11	14.30683366
hsa-miR-18b-5p	3.651956789	4.148016272	383.0566215	8.32E-11	14.30683366
hsa-miR-203a-3p	3.651956789	4.148016272	383.0566215	8.32E-11	14.30683366
hsa-miR-376b-3p	3.651956789	4.148016272	383.0566215	8.32E-11	14.30683366
hsa-miR-129-5p	2.142238942	3.393047571	339.3855229	1.39E-10	13.67219882
hsa-miR-3939	2.142238942	3.393047571	339.3855229	1.39E-10	13.67219882
hsa-miR-1250-5p	2.142129164	3.39310246	339.3198305	1.39E-10	13.67118375
hsa-miR-378h	2.142129164	3.39310246	339.3198305	1.39E-10	13.67118375
hsa-miR-4715-5p	5.257492417	4.950784086	338.7656547	1.40E-10	13.66261285
hsa-miR-492	5.257492417	4.950784086	338.7656547	1.40E-10	13.66261285
hsa-miR-6084	3.664963028	4.154409614	323.7403657	1.70E-10	13.42471457
hsa-miR-1204	4.382537344	4.513196772	319.9829759	1.79E-10	13.3634937
hsa-miR-4255	4.399175444	4.521515822	296.0592899	2.49E-10	12.95594698
hsa-miR-1258	2.971385733	3.807730744	280.5289182	3.13E-10	12.67332413
hsa-miR-1298-3p	2.971385733	3.807730744	280.5289182	3.13E-10	12.67332413
hsa-miR-29b-2-5p	2.971385733	3.807730744	280.5289182	3.13E-10	12.67332413
hsa-miR-30d-3p	2.971385733	3.807730744	280.5289182	3.13E-10	12.67332413
hsa-miR-3605-3p	2.971385733	3.807730744	280.5289182	3.13E-10	12.67332413
hsa-miR-369-3p	2.971385733	3.807730744	280.5289182	3.13E-10	12.67332413
hsa-miR-3912-5p	2.971385733	3.807730744	280.5289182	3.13E-10	12.67332413
hsa-miR-3927-5p	4.400500972	4.522288364	271.9681731	3.56E-10	12.51075879
hsa-miR-15b-3p	6.255974744	5.488742889	266.0153969	3.92E-10	12.39466885
hsa-miR-4482-3p	5.324833311	4.984454533	264.8550799	3.99E-10	12.3717379
hsa-miR-187-3p	8.349468633	6.496662417	235.4092218	6.58E-10	11.7534524
hsa-miR-141-3p	8.349358856	6.496717306	235.4050653	6.58E-10	11.75335977
hsa-miR-4315	4.385442289	4.5283824	219.7337957	8.82E-10	11.39192376
hsa-miR-302c-5p	5.283757194	4.963806697	214.9484389	9.69E-10	11.27639918
hsa-miR-100-3p	4.377116122	4.510595939	209.9111438	1.07E-09	11.15197825
hsa-miR-143-5p	4.377116122	4.510595939	209.9111438	1.07E-09	11.15197825
hsa-miR-181b-3p	4.377116122	4.510595939	209.9111438	1.07E-09	11.15197825
hsa-miR-20a-3p	4.377116122	4.510595939	209.9111438	1.07E-09	11.15197825
hsa-miR-25-5p	4.377116122	4.510595939	209.9111438	1.07E-09	11.15197825
hsa-miR-301a-3p	4.377116122	4.510595939	209.9111438	1.07E-09	11.15197825
hsa-miR-132-5p	4.322042439	4.521494681	208.4751556	1.10E-09	11.11596183
hsa-miR-3185	2.987969272	3.815912736	207.861666	1.12E-09	11.10049899
hsa-miR-514a-5p	2.987969272	3.815912736	207.861666	1.12E-09	11.10049899
hsa-miR-19b-1-5p	5.20579835	4.947022119	205.3600663	1.18E-09	11.03697048

hsa-miR-1244	2.153888914	3.398982335	204.2038761	1.20E-09	11.00734676
hsa-miR-3064-5p	2.153888914	3.398982335	204.2038761	1.20E-09	11.00734676
hsa-miR-3200-3p	2.153888914	3.398982335	204.2038761	1.20E-09	11.00734676
hsa-miR-382-3p	2.153888914	3.398982335	204.2038761	1.20E-09	11.00734676
hsa-miR-383-5p	2.153888914	3.398982335	204.2038761	1.20E-09	11.00734676
hsa-miR-4316	2.153888914	3.398982335	204.2038761	1.20E-09	11.00734676
hsa-miR-127-5p	7.598674919	6.135108493	198.9069072	1.35E-09	10.86944722
hsa-miR-3130-5p	3.639424383	4.155483225	192.1692618	1.56E-09	10.68863348
hsa-miR-3678-3p	3.639424383	4.155483225	192.1692618	1.56E-09	10.68863348
hsa-miR-3197	3.639534161	4.155428336	191.7990316	1.57E-09	10.67851484
hsa-miR-411-3p	4.338392956	4.513319422	191.2313987	1.59E-09	10.66296305
hsa-miR-376a-5p	-1.082985689	1.780545033	-187.148834	1.75E-09	10.5497309
hsa-miR-410-5p	-1.076912806	1.783471697	-186.1309188	1.79E-09	10.52111358
hsa-miR-6894-3p	-5.889545011	5.169650111	-183.5535933	1.90E-09	10.44794958
hsa-miR-3687	4.363247444	4.539479822	183.0366539	1.92E-09	10.43315125
hsa-miR-1273h-3p	5.250190517	4.947133136	179.8780795	2.07E-09	10.34181296
hsa-miR-130b-5p	5.250190517	4.947133136	179.8780795	2.07E-09	10.34181296
hsa-miR-15a-3p	5.250190517	4.947133136	179.8780795	2.07E-09	10.34181296
hsa-miR-1911-5p	5.250190517	4.947133136	179.8780795	2.07E-09	10.34181296
hsa-miR-27a-5p	5.250190517	4.947133136	179.8780795	2.07E-09	10.34181296
hsa-miR-339-5p	5.250190517	4.947133136	179.8780795	2.07E-09	10.34181296
hsa-miR-379-3p	5.250190517	4.947133136	179.8780795	2.07E-09	10.34181296
hsa-miR-4695-3p	4.395189528	4.519632642	179.5257722	2.08E-09	10.33152579
hsa-miR-135b-3p	4.36468275	4.540197475	176.829558	2.22E-09	10.25212269
hsa-miR-300	2.94077255	3.839511097	170.9218158	2.57E-09	10.07382089
hsa-miR-3200-5p	5.247829194	4.981770697	170.1087586	2.62E-09	10.04880061
hsa-miR-3620-3p	5.247829194	4.981770697	170.1087586	2.62E-09	10.04880061
hsa-miR-1291	4.399344889	4.557528544	169.1027846	2.68E-09	10.0176776
hsa-miR-1296-5p	2.941297383	3.839248681	169.0466463	2.69E-09	10.01593533
hsa-miR-3130-3p	2.970381039	3.820961553	168.7038266	2.71E-09	10.00528325
hsa-miR-597-3p	6.447491044	5.593610733	168.0416795	2.76E-09	9.984647605
hsa-miR-3606-3p	5.236567139	4.953944825	163.2108444	3.12E-09	9.831588141
hsa-miR-3074-3p	2.158668164	3.401262182	162.7701758	3.16E-09	9.817401233
hsa-miR-3934-3p	2.158558386	3.401317071	162.7566498	3.16E-09	9.816965167
hsa-miR-5010-3p	4.514224189	4.628172933	160.0601854	3.39E-09	9.729302027
hsa-miR-4461	2.96532245	3.827236147	159.9433398	3.40E-09	9.725470016
hsa-miR-4303	2.972949811	3.822245939	158.5988559	3.53E-09	9.681174426
hsa-miR-3978	2.973059589	3.82219105	158.2879497	3.56E-09	9.670877801
hsa-miR-7855-5p	-3.134494036	3.750708882	-158.1745102	3.57E-09	9.667115859
hsa-miR-2114-3p	3.629871722	4.159058806	155.0990828	3.88E-09	9.564085358
hsa-miR-1301-3p	3.618475539	4.167093869	152.7972684	4.13E-09	9.485625998
hsa-miR-3663-5p	3.631372	4.1735421	152.1383312	4.21E-09	9.462947837
hsa-miR-4300	3.631372	4.1735421	152.1383312	4.21E-09	9.462947837
hsa-miR-296-3p	2.974126339	3.822834203	152.0193879	4.22E-09	9.45884379
hsa-miR-3619-5p	2.961397878	3.816469972	146.9861306	4.87E-09	9.282165738
hsa-miR-1288-5p	5.254148194	4.949002197	146.9656826	4.87E-09	9.281435699
hsa-miR-1247-5p	2.974887461	3.845299831	145.9633797	5.02E-09	9.245526138
hsa-miR-223-5p	7.594413278	6.119244517	143.4497984	5.40E-09	9.154375994
hsa-miR-138-2-3p	4.336063383	4.532557614	141.8642306	5.66E-09	9.096053263
hsa-miR-3164	5.276455294	4.960155747	141.2679666	5.76E-09	9.07395178
hsa-miR-516a-3p	5.276455294	4.960155747	141.2679666	5.76E-09	9.07395178
hsa-miR-4521	3.632208694	4.160227292	140.3035735	5.94E-09	9.038006786
hsa-miR-182-5p	2.937748733	3.812997311	137.3874687	6.49E-09	8.927794966

hsa-miR-3680-3p	2.161354183	3.449433469	136.9277327	6.58E-09	8.910206436
hsa-miR-3923	2.148213497	3.406489515	128.6641548	8.57E-09	8.583571923
hsa-miR-3622b-3p	2.140265536	3.405794024	125.9477997	9.39E-09	8.471605069
hsa-miR-6731-3p	4.658037483	4.690582036	124.3565823	9.91E-09	8.404889004
hsa-miR-1273h-5p	5.227101278	4.969458361	121.3762067	1.10E-08	8.277600101
hsa-miR-1199-5p	2.19107445	3.431308258	121.3380956	1.10E-08	8.275952256
hsa-miR-377-5p	2.950477194	3.819361542	118.748552	1.21E-08	8.162756439
hsa-miR-3714	2.187940906	3.429741486	118.6565284	1.21E-08	8.158688578
hsa-miR-3177-3p	3.654371194	4.181335264	115.8352285	1.34E-08	8.032419372
hsa-miR-193b-5p	7.565595739	11.50211767	115.5190998	1.36E-08	8.0180797
hsa-miR-95-3p	-5.722889022	5.183371767	-113.9990898	1.43E-08	7.948579349
hsa-miR-939-3p	3.328729906	4.022221053	111.3404443	1.59E-08	7.824759248
hsa-miR-1295b-3p	2.987442544	3.81575915	110.238371	1.65E-08	7.772564052
hsa-miR-4434	5.509303256	5.112507728	99.16504876	2.59E-08	7.217132222
hsa-miR-4260	2.991433239	3.853572719	98.17537912	2.71E-08	7.164507039
hsa-miR-335-3p	2.103570636	3.412381724	95.98477194	2.98E-08	7.046111973
hsa-miR-4725-3p	4.4369455	4.586854072	93.10422109	3.39E-08	6.886250078
hsa-miR-136-5p	2.122354025	3.419419251	92.20847445	3.53E-08	6.835530169
hsa-miR-4746-3p	-5.243067867	5.410965456	-87.01979622	4.52E-08	6.531683997
hsa-miR-4466	4.008294983	12.90572712	85.54936191	4.85E-08	6.442279428
hsa-miR-205-5p	2.136473319	3.412359604	84.9353984	5.01E-08	6.404494353
hsa-miR-365b-5p	2.136473319	3.412359604	84.9353984	5.01E-08	6.404494353
hsa-miR-3685	2.178115011	3.410985606	83.18903648	5.47E-08	6.295507519
hsa-miR-4506	2.972415589	3.884656611	81.79333823	5.87E-08	6.206748931
hsa-miR-513b-3p	4.408545189	4.53993385	81.75542588	5.89E-08	6.204316866
hsa-miR-4642	3.780330139	4.262770464	81.67038875	5.91E-08	6.198857661
hsa-miR-7845-5p	-5.3893196	5.604780694	-80.27201254	6.36E-08	6.108261245
hsa-miR-6840-3p	-4.218612317	4.636336014	-78.84397283	6.87E-08	6.0141014
hsa-miR-5703	3.878082589	12.16873804	78.32700077	7.06E-08	5.979593598
hsa-miR-3613-3p	2.137248261	3.436088453	77.70147538	7.31E-08	5.937534459
hsa-miR-421	2.114944142	3.406694971	77.68845057	7.31E-08	5.936655106
hsa-miR-6776-3p	5.810980994	5.378776086	76.4947735	7.81E-08	5.855434048
hsa-miR-6885-3p	2.546367728	3.631039964	75.09991695	8.44E-08	5.758904973
hsa-miR-2110	2.164381856	3.417852183	73.67374192	9.16E-08	5.658339479
hsa-miR-4667-3p	4.569829828	4.642771014	73.32557098	9.34E-08	5.633493312
hsa-miR-7641	-13.53809602	10.02512963	-73.15248407	9.44E-08	5.621097615
hsa-let-7f-5p	-12.84678967	10.32601466	-71.42487682	1.04E-07	5.495744282
hsa-miR-3940-3p	2.146856483	3.431284342	68.17526334	1.27E-07	5.251530389
hsa-miR-431-5p	2.146856483	3.431284342	68.17526334	1.27E-07	5.251530389
hsa-miR-4425	2.146856483	3.431284342	68.17526334	1.27E-07	5.251530389
hsa-miR-8082	3.112232989	3.878044594	66.6817553	1.40E-07	5.135366189
hsa-miR-1285-3p	2.171598731	3.42992231	66.22270313	1.44E-07	5.099139107
hsa-miR-6785-3p	5.775158761	5.349725936	65.22619805	1.54E-07	5.019627401
hsa-miR-22-3p	-13.39614562	10.54159297	-61.22307428	2.01E-07	4.68750804
hsa-miR-6728-3p	5.673185811	5.18071585	60.45424639	2.12E-07	4.621247239
hsa-miR-509-5p	4.425380733	4.570546467	60.45151934	2.12E-07	4.621010717
hsa-miR-937-3p	-5.0882498	4.845615006	-60.44201301	2.12E-07	4.620186133
hsa-miR-154-3p	2.922310839	3.832268192	59.78484723	2.22E-07	4.562867286
hsa-miR-98-3p	-5.517885628	5.339726075	-59.61990831	2.25E-07	4.548382414
hsa-miR-6810-3p	5.439003528	5.077357864	58.13947696	2.50E-07	4.416552434
hsa-miR-520b	7.073403739	5.936640581	58.03590488	2.52E-07	4.407204553
hsa-miR-4697-3p	5.53444835	5.125080275	57.44872276	2.63E-07	4.353891881
hsa-miR-4651	5.103394883	10.30894746	57.09722976	2.70E-07	4.3217177

hsa-miR-486-5p	-11.59599494	7.653207156	-56.79744707	2.76E-07	4.294120372
hsa-miR-509-3-5p	3.711737961	4.213725081	56.41231848	2.84E-07	4.258452295
hsa-miR-7154-5p	-5.083209978	4.766305844	-55.27631789	3.10E-07	4.151811053
hsa-miR-4787-5p	4.761749761	10.00617949	54.97028484	3.18E-07	4.122708591
hsa-miR-373-3p	5.3337113	5.002516906	54.61297803	3.26E-07	4.088525063
hsa-miR-6857-5p	-3.479000933	3.549635956	-54.49497602	3.29E-07	4.077186809
hsa-miR-3187-3p	5.3740517	5.04488195	52.59080266	3.83E-07	3.890759296
hsa-miR-6850-3p	4.675961522	4.695836861	52.24627101	3.94E-07	3.856310166
hsa-miR-21-5p	21.83345567	29.14085667	51.88969739	4.06E-07	3.820417666
hsa-let-7e-5p	-8.750119272	8.177032747	-50.48745	4.56E-07	3.676842625
hsa-miR-3944-5p	2.172499833	3.477139767	50.38380463	4.60E-07	3.666073208
hsa-miR-4648	5.49261865	5.298117458	49.5608903	4.93E-07	3.579775185
hsa-miR-338-3p	8.101539289	6.433048744	49.51739839	4.95E-07	3.575174595
hsa-miR-1236-5p	2.511245344	8.8871882	48.61334045	5.35E-07	3.478620109
hsa-let-7a-5p	-13.68176841	12.01849557	-47.74708447	5.77E-07	3.384409418
hsa-miR-7157-3p	2.113182906	3.392362486	47.72525665	5.78E-07	3.38201357
hsa-miR-5585-3p	-5.107324572	5.215514575	-46.81688036	6.28E-07	3.281328704
hsa-miR-4636	-2.819733228	5.337275792	-46.14024661	6.68E-07	3.205057412
hsa-miR-4472	1.167733239	2.941722719	45.80609209	6.88E-07	3.166979013
hsa-miR-431-3p	5.407113061	5.104904536	44.5106159	7.78E-07	3.016691374
hsa-miR-942-3p	-4.704901886	4.693128215	-44.50326445	7.78E-07	3.015826164
hsa-miR-8069	-21.19788627	15.93854798	-44.03488122	8.14E-07	2.960405587
hsa-miR-4695-5p	5.479270778	9.539629794	43.62675567	8.47E-07	2.911634244
hsa-let-7i-5p	-10.44359602	9.611664944	-43.35585694	8.69E-07	2.879010154
hsa-miR-557	-1.803635917	5.803136281	-43.28324037	8.75E-07	2.870230514
hsa-miR-6791-5p	2.563423483	9.077080392	43.13743822	8.88E-07	2.852558072
hsa-miR-8063	4.120156533	8.283197072	42.89884781	9.09E-07	2.823510251
hsa-miR-4733-5p	3.731435133	4.2014886	42.84977705	9.14E-07	2.817516066
hsa-miR-3196	-2.329912994	7.154178592	-42.56138844	9.40E-07	2.782149597
hsa-miR-6875-3p	3.200409022	3.922242389	41.54418401	1.04E-06	2.655472047
hsa-miR-211-3p	5.102910644	9.368358267	41.47591309	1.05E-06	2.646859718
hsa-miR-6511b-3p	5.656653839	5.186183019	40.41978495	1.17E-06	2.511802442
hsa-miR-4793-5p	6.652628589	11.18783351	40.20428595	1.20E-06	2.48381356
hsa-miR-455-5p	17.33461372	16.88492123	39.84554108	1.24E-06	2.436887298
hsa-miR-146a-5p	15.41758108	15.90812926	39.44702577	1.30E-06	2.384263524
hsa-miR-4443	4.879239	15.45993389	39.40139069	1.30E-06	2.378203721
hsa-miR-6799-3p	5.675486772	5.195599486	39.14210939	1.34E-06	2.343641149
hsa-miR-192-5p	9.469256328	8.894915414	38.84621913	1.38E-06	2.303919281
hsa-miR-4646-5p	4.254309894	9.861698097	38.70830394	1.41E-06	2.285301904
hsa-miR-6831-3p	4.643090928	4.657206719	38.52476211	1.43E-06	2.260422757
hsa-miR-6759-3p	5.683478011	5.199595106	37.88342775	1.54E-06	2.172554727
hsa-miR-767-3p	-4.612865006	4.708893647	-37.81448876	1.55E-06	2.163021638
hsa-miR-378a-5p	-2.874885031	2.827410882	-36.91644018	1.72E-06	2.037233609
hsa-miR-1305	6.715402656	10.30753223	36.35842712	1.83E-06	1.957532533
hsa-miR-518b	5.636353833	5.15394795	36.33174734	1.84E-06	1.953691472
hsa-miR-4674	5.511575617	5.137330558	35.99295382	1.91E-06	1.904670483
hsa-miR-765	-5.744570656	5.400993033	-35.87284251	1.94E-06	1.887181096
hsa-miR-874-5p	3.191369194	3.917722475	35.55833889	2.01E-06	1.841109232
hsa-miR-151b	-5.508910372	5.404745831	-35.18345033	2.11E-06	1.785659786
hsa-miR-6855-5p	3.899556956	4.271816356	35.17874468	2.11E-06	1.784960053
hsa-miR-7846-3p	7.2511047	6.473328756	34.69788211	2.24E-06	1.712961074
hsa-miR-9500	-7.010851456	6.274582478	-34.4508	2.30E-06	1.675579464
hsa-miR-6874-5p	2.53952285	3.591799303	34.30118903	2.35E-06	1.652814872

hsa-miR-6867-5p	5.715970994	6.538666453	34.12174882	2.40E-06	1.625381127
hsa-miR-582-5p	-3.329699428	3.620742814	-33.6700189	2.54E-06	1.555679169
hsa-miR-10b-5p	-8.213593006	7.477264414	-33.50531981	2.59E-06	1.530034945
hsa-miR-222-3p	-4.5975339	7.877437578	-33.32710594	2.65E-06	1.502145112
hsa-miR-6803-5p	-11.49950901	8.935376056	-33.3167598	2.65E-06	1.500521436
hsa-miR-3195	2.821831033	12.69686382	33.26525709	2.67E-06	1.492431381
hsa-miR-1185-1-3p	3.908054639	9.745836681	33.11349247	2.72E-06	1.468519688
hsa-miR-8052	-4.813386311	4.690555511	-32.99513104	2.77E-06	1.449795326
hsa-miR-6810-5p	4.609745611	4.626910683	32.76229254	2.85E-06	1.412765788
hsa-miR-3198	-9.679587511	7.913530733	-32.70287898	2.87E-06	1.403275122
hsa-miR-6127	-11.29518238	8.812550789	-32.6978197	2.87E-06	1.402466167
hsa-miR-6861-3p	3.677205372	4.337413781	32.65765994	2.89E-06	1.396040404
hsa-miR-4533	3.060353189	3.86583785	32.50420494	2.95E-06	1.371414415
hsa-miR-7975	4.306209939	9.225330536	32.42064397	2.98E-06	1.357956261
hsa-miR-6089	-15.89154959	12.01059665	-32.31071291	3.02E-06	1.340198491
hsa-miR-6807-5p	-5.524870372	5.194777392	-31.86809387	3.20E-06	1.268088202
hsa-miR-6789-5p	-5.759959417	5.330195775	-31.86347671	3.21E-06	1.267330763
miRNABrightCorner30	-20.39726732	14.0633889	-31.8519077	3.21E-06	1.265432406
hsa-miR-3174	2.912887972	3.914259458	31.75691271	3.25E-06	1.249818784
hsa-miR-6891-5p	6.429423733	9.014119961	31.65042704	3.30E-06	1.232261373
hsa-miR-1273g-5p	4.487739417	4.580267714	31.63449504	3.31E-06	1.229629462
hsa-miR-184	5.583761322	5.414568528	31.24026044	3.49E-06	1.164081166
hsa-miR-518e-5p	5.769247772	5.242479986	31.07423713	3.57E-06	1.136231186
hsa-miR-129-1-3p	4.439147722	4.714547083	30.96494292	3.62E-06	1.117816742
hsa-miR-3156-5p	3.382335811	9.751526772	30.90959674	3.65E-06	1.108467149
hsa-miR-3911	-2.254236317	6.311346514	-30.89044786	3.66E-06	1.105228477
hsa-miR-92a-1-5p	2.4292683	3.53872475	30.80758954	3.70E-06	1.091191584
hsa-miR-1295a	3.6462381	4.259934733	30.63679088	3.79E-06	1.062138423
hsa-miR-373-5p	4.617189394	4.685657303	30.25317777	3.99E-06	0.996295699
hsa-miR-125b-5p	-14.49668563	11.12559471	-30.19405207	4.03E-06	0.986073945
hsa-miR-4634	5.360401156	12.43260029	30.11819585	4.07E-06	0.972930708
hsa-miR-4538	-3.191891711	3.827730739	-29.92929829	4.18E-06	0.940058306
hsa-miR-610	8.580824772	6.894421919	29.86081592	4.22E-06	0.928090058
hsa-miR-7-2-3p	4.626021883	4.635048819	29.61368475	4.37E-06	0.884673202
hsa-miR-718	-9.296765294	8.443457086	-28.98192482	4.79E-06	0.772031308
hsa-miR-6880-5p	-5.443667067	5.199008117	-28.91962167	4.83E-06	0.76079134
hsa-miR-1185-2-3p	3.069868739	8.409250719	28.59137458	5.07E-06	0.701174384
hsa-miR-4666b	5.44454425	5.251191164	28.53883977	5.11E-06	0.691570056
hsa-miR-4505	-10.25107851	9.438697814	-28.53342827	5.12E-06	0.69057974
hsa-miR-6846-3p	3.853027978	4.248551867	28.04360625	5.51E-06	0.600162738
hsa-miR-563	7.868320228	6.503152308	27.42201622	6.05E-06	0.483146395
hsa-miR-874-3p	-9.001030183	8.427734408	-27.38065452	6.09E-06	0.475267099
hsa-miR-6881-3p	-3.485005739	3.961840931	-27.35995321	6.11E-06	0.471319134
hsa-miR-99a-3p	2.4083897	3.548317794	27.19634044	6.27E-06	0.44001208
hsa-miR-615-3p	5.040709206	5.085330692	27.18095896	6.28E-06	0.437059287
hsa-miR-7111-3p	-5.748054761	5.171605569	-27.15094555	6.31E-06	0.431292835
hsa-miR-4532	4.041395439	10.1992596	27.04693145	6.42E-06	0.411259819
hsa-miR-150-3p	5.69367555	10.142553	26.92213755	6.54E-06	0.387123898
hsa-miR-625-3p	5.207761967	5.150862239	26.90104251	6.56E-06	0.383033063
hsa-miR-4728-5p	-5.658001844	5.432632628	-26.89515099	6.57E-06	0.381889989
hsa-miR-3682-3p	3.947404867	5.866677861	26.89489201	6.57E-06	0.381839736
hsa-miR-6775-5p	-7.768993189	7.11968525	-26.89478111	6.57E-06	0.381818215
hsa-miR-1267	4.405240878	4.738667267	26.7915768	6.68E-06	0.361753984

hsa-miR-6871-5p	-3.594573489	4.125336544	-26.74946443	6.72E-06	0.353544852
hsa-miR-5001-5p	-5.328256772	4.926947919	-26.74127209	6.73E-06	0.351946403
hsa-miR-6778-5p	-5.356787689	5.178488961	-26.63089173	6.85E-06	0.330362216
hsa-miR-8075	-4.64373455	4.770940336	-26.61356787	6.87E-06	0.32696662
hsa-miR-129-2-3p	3.748094817	4.366209214	26.51149915	6.98E-06	0.306916002
hsa-miR-6892-5p	2.9497235	3.958159317	26.48237257	7.01E-06	0.301180318
hsa-miR-1249-5p	8.074942039	11.24318485	26.48225919	7.01E-06	0.301157981
hsa-miR-24-3p	-8.852555428	13.53755906	-26.25981632	7.27E-06	0.257147105
hsa-miR-6790-5p	5.251900111	5.085623394	26.24834275	7.28E-06	0.254867052
hsa-miR-4653-3p	5.882898194	9.815743897	26.13767611	7.41E-06	0.232824482
hsa-let-7g-5p	-9.970715511	8.253857806	-25.7800535	7.86E-06	0.160957759
hsa-miR-1273e	-3.029982783	3.611009553	-25.75025668	7.90E-06	0.154925526
hsa-miR-3137	5.775062272	10.06142454	25.71769413	7.94E-06	0.148325499
hsa-miR-181a-5p	-8.086162172	12.11566614	-25.58337406	8.12E-06	0.121012989
hur_6	6.103431478	9.216849939	25.47668514	8.26E-06	0.09921791
hsa-miR-574-3p	-8.47308405	7.323574358	-25.37754823	8.40E-06	0.078884706
hsa-miR-627-5p	5.709221794	5.224163942	25.31788672	8.48E-06	0.066610178
hsa-miR-320b	-6.736763533	11.28833822	-25.21445782	8.63E-06	0.045263326
hsa-miR-99b-5p	3.634678189	4.302983422	25.10483603	8.79E-06	0.022543788
hsa-miR-6819-3p	3.1941102	6.127957972	24.98862554	8.97E-06	-0.00164841
hsa-miR-130a-3p	-7.083430956	6.090075594	-24.91651309	9.08E-06	-0.016716339
hsa-miR-4441	5.663590044	7.711872539	24.72353723	9.38E-06	-0.057251287
hsa-miR-5696	5.474550383	5.211134853	24.57194794	9.63E-06	-0.089312335
hsa-miR-6132	4.928871239	8.109420592	24.39429875	9.93E-06	-0.127134174
hsa-miR-1268a	2.267965222	11.01767912	24.39329054	9.93E-06	-0.127349598
hsa-miR-3127-5p	5.4033506	8.853673878	24.22165477	1.02E-05	-0.164151459
hsa-miR-6716-5p	4.326394039	4.536990692	24.14950242	1.04E-05	-0.17969905
hsa-miR-3163	6.677264994	8.122047142	24.04280503	1.06E-05	-0.202774609
hsa-miR-1249-3p	5.640854439	10.85045301	23.93032238	1.08E-05	-0.227210875
hsa-miR-6872-5p	-5.844866594	5.294280814	-23.88534902	1.08E-05	-0.237012788
hsa-miR-6895-5p	-4.843333378	4.830122428	-23.71636432	1.12E-05	-0.274006108
hsa-miR-664a-5p	-4.83378695	4.871420508	-23.65930622	1.13E-05	-0.286555654
hsa-miR-5581-5p	3.073898072	8.500927503	23.56658067	1.15E-05	-0.307013737
hsa-miR-8077	-4.854382369	4.646705996	-23.50002517	1.16E-05	-0.32174684
hsa-miR-6831-5p	-2.302288428	7.374562597	-23.49787137	1.16E-05	-0.322224302
hsa-miR-1268b	12.04697941	14.89588841	23.48528078	1.17E-05	-0.325016301
hsa-miR-8059	2.410413539	3.540868025	23.4743199	1.17E-05	-0.327448107
mr_1	-13.29588234	11.36929683	-23.43940019	1.17E-05	-0.335202934
hsa-miR-195-5p	-7.895415794	11.00497768	-23.41207189	1.18E-05	-0.341279831
hsa-miR-155-5p	-7.055342872	5.988672225	-22.97509403	1.28E-05	-0.439409264
hsa-miR-7155-5p	-3.859861706	4.200196753	-22.9728987	1.28E-05	-0.43990688
hsa-miR-199a-3p	-8.798220544	12.69286117	-22.87220006	1.30E-05	-0.462782655
hsa-miR-6893-5p	6.628698172	9.059638969	22.85146106	1.31E-05	-0.46750625
hsa-miR-6869-5p	-14.22105093	11.43780398	-22.80003254	1.32E-05	-0.479238034
hsa-miR-3940-5p	-1.895968878	6.629683856	-22.78682047	1.32E-05	-0.482256152
hsa-miR-199a-5p	-7.464223567	11.11799591	-22.75516709	1.33E-05	-0.489493944
hsa-miR-371a-3p	5.135575606	5.087619997	22.69746172	1.35E-05	-0.502714252
hsa-miR-4750-3p	5.395408183	5.231232631	22.68319911	1.35E-05	-0.505986913
hsa-miR-7108-5p	5.050102411	6.876918078	22.34311122	1.44E-05	-0.584627978
hsa-miR-7515	-8.5164112	6.986881194	-22.33596511	1.44E-05	-0.586293024
hsa-miR-4514	6.255378911	6.988667422	22.30268414	1.45E-05	-0.5940544
hsa-miR-4269	5.104754544	6.912560306	22.130029	1.50E-05	-0.634502524
hsa-miR-125a-3p	5.388786833	12.41208445	21.88838657	1.57E-05	-0.691636244

hsa-miR-6776-5p	5.475592411	5.112604044	21.87900174	1.57E-05	-0.693867667
hsa-miR-6126	3.3626295	8.483941728	21.86679848	1.57E-05	-0.696770633
hsa-miR-29c-3p	21.99464321	23.91166331	21.78174327	1.60E-05	-0.717048254
hsa-miR-6781-5p	-3.576088489	3.737767283	-21.72580186	1.62E-05	-0.730427433
hsa-miR-6877-5p	-7.307548456	6.778786856	-21.59135049	1.66E-05	-0.762722262
hsa-miR-133a-3p	-2.639515594	3.258485642	-21.51219707	1.69E-05	-0.781827162
hsa-miR-6801-3p	5.967272872	5.116502519	21.46617687	1.70E-05	-0.792966603
hsa-miR-6125	-9.339678456	8.4526323	-21.46486609	1.70E-05	-0.793284229
hsa-miR-4685-5p	6.790178433	7.47921735	21.42056597	1.72E-05	-0.804030126
hsa-miR-6722-3p	4.78692075	8.324587508	21.35423538	1.74E-05	-0.820160823
hsa-miR-26b-5p	-6.376560789	8.87696095	-21.01045346	1.86E-05	-0.904559819
hsa-miR-937-5p	3.46368515	9.802696736	20.88359081	1.91E-05	-0.936047447
hsa-miR-6073	-3.249752372	3.537108892	-20.87644037	1.91E-05	-0.937827785
hsa-miR-3170	2.256597811	3.464069939	20.82241537	1.94E-05	-0.951298448
hsa-miR-623	-2.461594661	3.063660919	-20.6910211	1.99E-05	-0.984204031
hsa-miR-5195-3p	6.534320478	10.26594906	20.63299015	2.01E-05	-0.99880222
hsa-miR-6873-5p	-3.401917817	3.930439986	-20.54220506	2.05E-05	-1.021720924
hsa-miR-6886-5p	-6.444763472	6.574810975	-20.52681815	2.06E-05	-1.025615188
hsa-miR-6131	-5.054806439	4.591455319	-20.51248091	2.06E-05	-1.029246365
hsa-miR-708-5p	-4.858929633	4.654342422	-20.50863355	2.06E-05	-1.030221207
hsa-miR-6723-5p	-4.478883494	4.666076619	-20.44773677	2.09E-05	-1.045675085
hsa-miR-320d	-6.863046828	11.59077273	-20.42991753	2.10E-05	-1.050205635
hsa-miR-3189-3p	2.282803883	3.499258042	20.401328	2.11E-05	-1.057482629
hsa-miR-744-3p	2.402366344	3.52322105	20.35284389	2.13E-05	-1.069846335
hsa-miR-642b-3p	3.487137428	9.701777036	20.34541063	2.13E-05	-1.071744406
hsa-miR-34a-5p	17.52203323	18.26910602	20.25191999	2.18E-05	-1.09567527
hsa-miR-6756-5p	3.871342894	9.474314625	20.23260596	2.19E-05	-1.100632582
hsa-miR-101-3p	-4.824183106	4.838413553	-20.13574218	2.23E-05	-1.125564622
hsa-miR-4707-3p	3.63321425	4.299556281	20.09916535	2.25E-05	-1.135009796
hsa-miR-7157-5p	2.4548069	3.658992111	20.09492548	2.25E-05	-1.136105739
hsa-miR-125b-2-3p	-4.860159778	4.524744706	-20.04884625	2.27E-05	-1.148031154
hsa-miR-23b-3p	-13.60579603	10.66059976	-19.88016202	2.35E-05	-1.191917139
hsa-miR-4713-3p	2.450688044	8.976376606	19.77885717	2.40E-05	-1.218448774
hsa-miR-6858-5p	-5.447667489	5.708288367	-19.68568334	2.45E-05	-1.242968559
hsa-miR-583	5.351997311	5.449252167	19.67801681	2.46E-05	-1.244991149
hsa-miR-8087	-5.494385972	5.143330103	-19.66472901	2.46E-05	-1.248498575
hsa-miR-454-3p	-1.793108858	3.298821746	-19.62484163	2.48E-05	-1.259041123
hsa-miR-1273f	5.854265011	9.287551494	19.6106653	2.49E-05	-1.262793085
hsa-miR-6829-5p	-2.131398428	6.284644997	-19.55582905	2.52E-05	-1.277331289
hsa-miR-762	3.821965522	9.380320939	19.54666076	2.53E-05	-1.279765879
hsa-miR-100-5p	-12.73368183	10.12352969	-19.53635225	2.53E-05	-1.28250458
hsa-miR-671-5p	2.747671272	9.477722714	19.53204607	2.53E-05	-1.283649041
hsa-miR-4257	-4.4113491	4.653352611	-19.43270218	2.59E-05	-1.310120519
hsa-miR-6827-3p	5.558048572	4.826661008	19.32927227	2.65E-05	-1.337821733
hsa-miR-6815-5p	-3.2494525	3.564893539	-19.3199209	2.65E-05	-1.340333413
hsa-miR-6851-5p	-3.726064939	3.737577886	-19.29088324	2.67E-05	-1.348140199
hsa-miR-518a-5p	7.931686222	6.763518017	19.24405622	2.70E-05	-1.360753892
hsa-miR-1273d	4.376267267	4.695019739	19.17400623	2.74E-05	-1.379679215
hsa-miR-4690-5p	-4.151011294	4.369761892	-19.04776203	2.82E-05	-1.413957693
hsa-miR-4667-5p	3.845583761	9.540002292	19.0315926	2.83E-05	-1.418364126
hsa-miR-6792-5p	3.903691372	5.852080875	19.02528878	2.83E-05	-1.42008301
hsa-miR-6869-3p	-4.194677239	4.324211269	-19.00638121	2.84E-05	-1.425241939
hsa-miR-371b-5p	4.3795039	9.02362785	18.86984039	2.93E-05	-1.462646579

hsa-miR-6507-3p	6.379618594	4.883993931	18.80883414	2.97E-05	-1.479444419
hsa-miR-6851-3p	4.536057611	4.895077906	18.70173513	3.04E-05	-1.509062791
hsa-miR-196a-5p	-6.248512556	8.194120361	-18.68376988	3.06E-05	-1.514047314
hsa-miR-6820-5p	3.321553678	7.76572475	18.57118283	3.13E-05	-1.5453919
hsa-miR-4656	-2.849422333	6.023894139	-18.44976957	3.22E-05	-1.579402186
hsa-miR-1915-3p	5.85795345	13.52102483	18.41801066	3.25E-05	-1.588334514
hsa-miR-139-5p	8.5453454	7.106524389	18.33664148	3.31E-05	-1.611288665
hsa-miR-1471	6.821587878	10.64598853	18.32548377	3.31E-05	-1.614443984
hsa-miR-6890-5p	-4.670922367	4.724749372	-18.3048885	3.33E-05	-1.620273096
hur_1	3.009973267	11.62794577	18.28815984	3.34E-05	-1.625012545
hsa-miR-766-5p	-4.775670617	4.617240664	-18.26403772	3.36E-05	-1.631854104
hsa-miR-208a-5p	3.916176656	7.357602806	18.2595133	3.37E-05	-1.633138308
hsa-miR-6876-5p	-5.014693117	4.742363336	-18.12939744	3.47E-05	-1.670203552
hsa-miR-892b	-4.7761889	4.748944572	-18.11114586	3.48E-05	-1.675423482
hsa-miR-3614-5p	5.171426761	6.830340764	17.8699993	3.68E-05	-1.744876436
hsa-miR-5100	-7.867248144	7.629962872	-17.83310304	3.72E-05	-1.755583418
hsa-miR-4311	2.731176644	3.87542095	17.79006153	3.75E-05	-1.768100988
hsa-miR-532-5p	-5.295597783	5.053690031	-17.70614437	3.83E-05	-1.792591248
hsa-miR-6779-5p	2.421543978	9.027278522	17.58792329	3.94E-05	-1.827285066
hsa-miR-4462	5.349908128	8.123946486	17.56497921	3.96E-05	-1.834044649
hsa-miR-6839-5p	5.048421911	4.348244933	17.50801516	4.02E-05	-1.850864101
hsa-miR-4658	3.39698155	4.249843486	17.46703363	4.06E-05	-1.86299744
hsa-miR-4516	3.339489711	16.74675276	17.40006369	4.12E-05	-1.882884838
hsa-miR-6800-5p	2.047948267	13.49026983	17.39245869	4.13E-05	-1.885147924
hsa-miR-99b-3p	-5.583314267	5.556867383	-17.28962466	4.23E-05	-1.915843951
hsa-miR-4470	3.868393456	5.970161017	17.2834482	4.24E-05	-1.917693274
hsa-miR-28-3p	4.284790761	4.760008019	17.21624815	4.31E-05	-1.937855574
hsa-miR-6763-5p	2.328271889	11.00181498	17.14657469	4.38E-05	-1.958840889
hsa-miR-6889-5p	-6.188757117	5.606768431	-17.12940502	4.40E-05	-1.964025036
hsa-miR-1908-3p	5.496666894	6.711351414	17.08874036	4.45E-05	-1.976323334
hsa-miR-4465	-5.728071867	5.281137739	-16.93620373	4.62E-05	-2.022709967
hsa-miR-6799-5p	-4.44439025	4.632719892	-16.90653661	4.65E-05	-2.031778888
hsa-miR-6793-5p	5.329046667	5.489461239	16.89029009	4.67E-05	-2.036751821
hsa-miR-4298	-2.858647433	7.632513561	-16.76208681	4.82E-05	-2.076157508
hsa-miR-5194	-4.479316344	4.613711294	-16.73179502	4.86E-05	-2.085510967
hsa-miR-652-3p	5.160146978	7.750272811	16.69130289	4.91E-05	-2.098039759
hsa-miR-1224-5p	2.3455628	9.022875883	16.65589596	4.95E-05	-2.109019311
hsa-miR-4669	-3.563877439	8.835167708	-16.57442919	5.05E-05	-2.13436804
hsa-miR-409-3p	-3.334154089	5.599872261	-16.56693897	5.06E-05	-2.136704714
hsa-miR-212-3p	4.252929717	4.735729064	16.5374721	5.10E-05	-2.145907249
hsa-miR-6887-3p	-3.337919278	3.884991206	-16.51173523	5.13E-05	-2.153957915
hsa-miR-4419a	3.555982789	9.175007911	16.44630855	5.22E-05	-2.174478713
hsa-miR-4688	-3.548992556	3.994527967	-16.44245966	5.23E-05	-2.175688364
hsa-miR-575	2.191979083	13.1779488	16.44031762	5.23E-05	-2.176361697
hsa-miR-10a-5p	-5.642922356	5.169860133	-16.40586162	5.28E-05	-2.18720433
hsa-miR-7851-3p	-8.305966428	7.337619458	-16.34737418	5.35E-05	-2.205659801
hsa-miR-6796-5p	-3.885008561	4.372512153	-16.33679901	5.37E-05	-2.209003578
hsa-miR-6841-3p	4.865746083	4.519116453	16.31581901	5.40E-05	-2.215643488
hsa-miR-518c-5p	3.287564422	4.347831489	16.3043384	5.41E-05	-2.21928046
hsa-miR-5088-5p	-4.281716783	4.621381019	-16.29788824	5.42E-05	-2.22132491
hsa-miR-7113-5p	-3.5353949	3.987212261	-16.21598114	5.54E-05	-2.247354659
hsa-miR-4776-5p	6.875297583	8.760381814	16.16476393	5.61E-05	-2.263696052
hsa-miR-4496	6.958580428	10.18690784	16.1247034	5.67E-05	-2.276512759

hsa-miR-4734	4.253188344	4.698392578	16.11049403	5.69E-05	-2.281066217
hsa-miR-887-3p	-6.364294394	6.304269997	-16.06192046	5.77E-05	-2.29666125
hsa-miR-4486	5.120692628	8.245134819	16.04837163	5.79E-05	-2.30101938
hsa-miR-875-3p	2.400557767	3.535940139	16.0152727	5.84E-05	-2.311680997
hsa-miR-25-3p	-8.261238533	7.958467683	-15.98912535	5.88E-05	-2.3201185
hsa-miR-6090	3.226735111	16.87812444	15.90946037	6.00E-05	-2.345908154
hsa-miR-6757-5p	3.413976567	8.991545794	15.87048561	6.06E-05	-2.358570842
hsa-miR-512-3p	-2.593642956	3.017245022	-15.86356443	6.07E-05	-2.360822638
hsa-miR-6081	4.858621339	5.145789631	15.84872411	6.10E-05	-2.365654119
hsa-miR-877-5p	-3.979460428	4.188279358	-15.84852084	6.10E-05	-2.365720327
hsa-miR-519e-5p	5.736725278	8.831761022	15.71501884	6.32E-05	-2.409382052
hsa-miR-7151-3p	2.802394033	4.015326478	15.65784917	6.41E-05	-2.428189052
hsa-miR-378a-3p	-4.964257378	8.1848621	-15.64475423	6.44E-05	-2.432506209
hsa-miR-6717-5p	-4.604257606	4.975796619	-15.62983324	6.46E-05	-2.437429631
hsa-miR-27b-3p	-8.0156531	11.34901386	-15.54492436	6.61E-05	-2.465533407
hsa-let-7c-5p	-13.21630578	11.44584194	-15.53092309	6.64E-05	-2.470181883
hsa-miR-4280	2.114209583	3.549469308	15.50596351	6.68E-05	-2.478478601
hsa-miR-550b-2-5p	4.274030389	5.015780039	15.3940015	6.89E-05	-2.515854769
hsa-miR-7108-3p	2.345527433	3.690961428	15.35629241	6.96E-05	-2.528502225
hsa-miR-4741	4.013833161	12.80648225	15.29856832	7.07E-05	-2.547920769
hsa-miR-6845-5p	-4.512890156	4.854927217	-15.15011635	7.36E-05	-2.598186746
hsa-miR-548ai	5.165357978	4.250703767	15.08415836	7.50E-05	-2.620672673
hsa-miR-4767	-3.310252156	3.873274244	-15.05232324	7.57E-05	-2.631559589
hsa-miR-532-3p	-3.840000894	3.767426775	-15.05158883	7.57E-05	-2.631811004
hsa-miR-185-5p	-5.277619667	5.311766517	-14.92428263	7.84E-05	-2.675571797
hsa-miR-6765-5p	-1.469800956	3.9190872	-14.84940313	8.01E-05	-2.701479349
hsa-miR-7856-5p	-3.688818439	4.069286825	-14.83693736	8.04E-05	-2.705804584
hsa-miR-6775-3p	5.878315069	5.161876576	14.81623055	8.08E-05	-2.712996933
hsa-miR-6785-5p	-10.82280041	8.431269975	-14.7885217	8.15E-05	-2.722636529
hsa-miR-6772-5p	-5.567124078	5.174962206	-14.73465966	8.27E-05	-2.741424336
hsa-miR-3147	3.478552878	5.092889839	14.69934616	8.36E-05	-2.753778043
hsa-miR-126-3p	-6.930241006	6.466485819	-14.69037361	8.38E-05	-2.756921451
hsa-miR-10b-3p	-4.764221289	4.969423906	-14.67875381	8.41E-05	-2.76099503
hsa-miR-4271	-2.3160515	7.121186956	-14.55053727	8.72E-05	-2.806151134
hsa-miR-425-5p	-3.864192222	6.16596225	-14.54511735	8.73E-05	-2.808068367
hsa-miR-4530	-5.306786922	13.40560767	-14.54308347	8.74E-05	-2.808788004
hsa-miR-6864-3p	-4.906899794	4.820922275	-14.41765055	9.06E-05	-2.8533572
hsa-miR-186-5p	-3.5183031	6.295220328	-14.38096348	9.16E-05	-2.866463332
hsa-miR-6820-3p	4.7792641	4.495968944	14.30619685	9.36E-05	-2.893272681
hsa-miR-4327	-1.70960425	6.339104825	-14.26306674	9.48E-05	-2.908799202
hsa-miR-370-3p	-2.885163261	6.524147647	-14.26222226	9.48E-05	-2.909103657
hsa-miR-214-3p	-7.264414194	10.97574959	-14.22862475	9.57E-05	-2.921230437
hsa-miR-130b-3p	-5.001102139	5.449459042	-14.15277527	9.79E-05	-2.948709032
hsa-miR-6809-5p	3.364820517	9.873659986	14.14355769	9.82E-05	-2.952057973
hsa-miR-6821-5p	1.7265935	13.48190872	14.12280479	9.88E-05	-2.9596056
hsa-miR-4647	-4.963075078	4.961370644	-14.11237581	9.91E-05	-2.963402534
hsa-miR-134-5p	4.379412022	10.75980983	14.0999367	9.94E-05	-2.967934822
hsa-miR-3917	4.27059655	8.054323147	14.08649864	9.98E-05	-2.97283539
hsa-miR-1233-5p	1.9545666	5.677409517	14.08388039	9.99E-05	-2.973790731
hsa-miR-6830-5p	5.680194378	5.018056811	14.06890715	0.000100352	-2.9792574
hsa-miR-6069	3.092414983	6.616925653	14.04463652	0.000101078	-2.988130361
hsa-miR-320e	-6.966401144	11.23008534	-14.02586825	0.000101644	-2.995001819
hsa-miR-6754-5p	5.112570453	5.291411179	13.99113212	0.000102701	-3.007742714

hsa-miR-8089	-5.847969072	5.993178281	-13.96438165	0.000103525	-3.017575199
hsa-miR-513a-5p	-5.859622622	5.538052022	-13.92762366	0.00010467	-3.031115536
hsa-miR-664a-3p	-3.980411772	3.943859781	-13.92661655	0.000104701	-3.031486999
hsa-miR-8072	5.126619567	4.348553983	13.90214614	0.000105472	-3.040520631
hsa-miR-514b-5p	-1.877431072	5.766478525	-13.84015781	0.000107457	-3.063472856
hsa-miR-6797-3p	3.008750594	6.612861092	13.75456476	0.000110274	-3.095327283
hsa-miR-5006-5p	5.482845561	12.6219798	13.75188942	0.000110364	-3.096325995
hsa-miR-33b-3p	5.872766428	7.162638025	13.73977069	0.00011077	-3.100852269
hsa-miR-107	-8.674325711	7.472460539	-13.65833269	0.000113551	-3.131368243
hsa-miR-30d-5p	-5.242261483	8.877193642	-13.56202541	0.000116951	-3.167681103
hsa-miR-4763-3p	-3.123912522	9.739559783	-13.52439036	0.000118314	-3.181938434
hsa-miR-6813-3p	4.238238461	5.988281481	13.43883706	0.000121485	-3.214490098
hsa-miR-7111-5p	-4.534796911	4.786481439	-13.40322882	0.000122835	-3.228096771
hsa-miR-4716-3p	-1.887184872	5.801638503	-13.39219794	0.000123257	-3.232318903
hsa-miR-7109-5p	5.033922883	6.313378875	13.38452551	0.000123552	-3.235257525
hsa-miR-6863	-4.472493167	4.558174683	-13.31282533	0.000126348	-3.262797366
hsa-miR-4738-3p	-2.50031875	5.378998514	-13.28453415	0.000127473	-3.273702848
hsa-miR-5195-5p	-2.786821439	3.291410914	-13.28411256	0.00012749	-3.273865528
hsa-miR-605-5p	4.335717133	5.179704733	13.25107001	0.000128819	-3.286630976
hsa-miR-6737-5p	5.060471611	4.229131428	13.18897799	0.000131364	-3.310701462
hsa-miR-1825	2.44698365	5.536680003	13.17382281	0.000131994	-3.31659288
hsa-miR-1247-3p	-3.55655225	4.030945736	-13.1269857	0.000133966	-3.334841188
hsa-miR-760	3.333884622	4.456847578	13.10733925	0.000134804	-3.342514144
hsa-miR-93-3p	-3.349425236	3.849819276	-13.05987178	0.000136855	-3.361097907
hsa-miR-6768-5p	3.447849778	7.700196339	13.04052234	0.000137703	-3.368691759
hur_4	3.442494156	9.713286206	13.03424887	0.000137979	-3.371156135
hsa-miR-6752-3p	4.362172128	6.006480486	13.02954295	0.000138186	-3.373005478
hsa-miR-3692-5p	3.477391622	7.682967506	13.01604246	0.000138784	-3.378314465
hsa-miR-1539	6.009022861	6.927491392	12.99891172	0.000139547	-3.38505857
hsa-miR-455-3p	-8.018121789	10.76620213	-12.90992412	0.000143593	-3.420228126
hsa-miR-3616-3p	2.07802945	3.550077625	12.89792078	0.00014415	-3.424989694
hsa-miR-6068	2.94864905	8.531363858	12.87644149	0.000145153	-3.433520769
hsa-miR-6855-3p	3.996399322	4.650635378	12.83487376	0.000147119	-3.450068967
hsa-miR-7150	5.975402611	6.395051089	12.77212754	0.000150148	-3.475145018
hsa-miR-3138	-1.697725367	7.325027889	-12.57464322	0.000160197	-3.554838135
hsa-miR-634	5.65978765	4.811984053	12.48540134	0.000165009	-3.591240875
hsa-miR-6780b-5p	2.270447806	10.53852685	12.44138888	0.000167448	-3.609284867
hsa-miR-221-3p	-7.122492578	6.794790539	-12.43473237	0.00016782	-3.612019125
hsa-miR-423-5p	-7.40609135	7.037062814	-12.4117925	0.000169112	-3.621452639
hsa-miR-148a-3p	-6.9178194	9.634935522	-12.3676003	0.000171636	-3.6396723
hsa-miR-6857-3p	3.526099344	3.854267056	12.3368259	0.000173421	-3.652396457
hsa-miR-26a-5p	-7.344052117	11.63921855	-12.3275602	0.000173963	-3.656233381
hsa-miR-199b-5p	-6.704986694	7.311248081	-12.31792312	0.000174529	-3.660226991
hsa-miR-1182	2.2109183	5.894434433	12.27392204	0.000177141	-3.678498673
hsa-miR-1470	4.040280017	5.184568269	12.18836798	0.00018236	-3.714203333
hsa-miR-214-5p	-3.476266878	5.599481661	-12.16484982	0.000183827	-3.724059754
hsa-miR-15a-5p	-7.474686522	6.7937208	-12.1584258	0.000184231	-3.726755179
hsa-miR-31-3p	-2.653487053	4.268254346	-12.13353628	0.000185804	-3.73721117
hsa-miR-6133	3.511352106	6.117101964	12.12513639	0.000186339	-3.740744497
hsa-miR-3663-3p	2.363254583	12.04103113	12.09365343	0.000188359	-3.754008081
hsa-miR-4294	2.819070967	4.023345794	12.02610888	0.000192787	-3.782574455
hsa-miR-8085	-6.274605233	6.284848483	-11.98468404	0.000195565	-3.800169055
hsa-miR-320c	1.871100867	16.24273179	11.85678856	0.00020446	-3.854854681

hsa-miR-93-5p	4.022878217	7.426022886	11.84211074	0.000205512	-3.861166127
hsa-miR-1281	-1.62446585	4.251420808	-11.82060508	0.000207066	-3.870426839
hsa-miR-296-5p	7.468313222	9.988892572	11.81467835	0.000207497	-3.872981781
hsa-miR-1226-5p	3.118623517	7.273930769	11.80857592	0.000207942	-3.875613728
hsa-miR-4739	4.020891183	13.1800232	11.78085614	0.000209977	-3.887585241
hsa-miR-27a-3p	-7.027874406	10.22518986	-11.78074426	0.000209985	-3.887633614
hsa-miR-564	-4.199784672	3.978881947	-11.76874606	0.000210873	-3.892823615
hsa-miR-4701-3p	6.602850683	8.817822364	11.72041936	0.000214499	-3.913778569
hsa-miR-6879-5p	4.483871328	6.007777697	11.69033143	0.000216795	-3.926866067
hsa-miR-8060	3.430216017	5.821555108	11.68961542	0.00021685	-3.927177895
hsa-miR-4665-5p	4.831235889	9.144041222	11.63715188	0.000220927	-3.950075443
hsa-miR-3141	5.484513233	12.40808994	11.62344292	0.000222008	-3.956074653
hsa-miR-320a	-5.854823167	9.741194328	-11.62285854	0.000222054	-3.956330529
hsa-miR-4468	-2.736077372	3.363908269	-11.52379213	0.000230063	-3.999883752
hsa-miR-625-5p	-4.125041689	3.950719767	-11.51173109	0.000231063	-4.005210197
hsa-miR-98-5p	-5.885407408	5.939730296	-11.45990495	0.000235417	-4.028157578
hsa-miR-6753-5p	5.554359328	5.166566614	11.44517973	0.000236673	-4.0346953
hsa-miR-6165	-3.783964311	9.759254911	-11.443307	0.000236833	-4.035527324
hsa-miR-7704	3.324316922	7.092294856	11.4240251	0.000238491	-4.044101377
hsa-miR-6819-5p	2.63096795	8.852250569	11.41692149	0.000239106	-4.047263546
hsa-miR-6862-5p	3.348693061	3.464746592	11.39744062	0.000240801	-4.055944914
hsa-miR-4507	-8.308022344	7.879164567	-11.37491349	0.000242779	-4.066001147
hsa-miR-6088	2.739043222	14.96013	11.34155432	0.000245746	-4.080927174
hsa-miR-146b-5p	-5.318359211	5.118285244	-11.33625135	0.000246222	-4.083303689
hsa-miR-20b-5p	-2.910447375	4.473077085	-11.2996272	0.000249538	-4.099745181
hsa-miR-4697-5p	-4.685659922	6.085638672	-11.23218502	0.00025579	-4.130152505
hsa-miR-4299	-6.035472589	10.15890923	-11.20037589	0.000258806	-4.14455342
hsa-miR-3656	-3.290973167	8.745631906	-11.15083864	0.00026359	-4.167056644
hsa-miR-7114-5p	3.617387972	5.697358108	11.14593846	0.000264069	-4.169287719
hsa-miR-1238-5p	4.260528356	5.288257461	11.11078578	0.000267537	-4.185319777
hsa-miR-210-3p	-3.869260494	6.571073036	-11.07939343	0.000270682	-4.199676937
hsa-miR-7114-3p	-3.575289078	4.012224783	-11.07302551	0.000271325	-4.202593912
hsa-miR-6884-3p	-4.3238237	4.663725661	-11.0646898	0.00027217	-4.206414643
hsa-miR-940	-3.010369917	6.089559008	-11.04402512	0.000274279	-4.215898032
hsa-miR-5571-5p	4.886849822	5.547419561	10.95680996	0.000283405	-4.256105766
hsa-miR-4312	4.8580208	5.494862411	10.94623434	0.000284537	-4.261001577
hsa-miR-5699-5p	2.26488545	8.198822764	10.92950416	0.000286339	-4.268755543
hsa-miR-6500-5p	5.676127683	4.972984375	10.92067658	0.000287295	-4.272851334
hsa-miR-4665-3p	4.615487128	9.105809542	10.91731262	0.00028766	-4.274412941
hsa-miR-6849-5p	3.447601472	5.933692708	10.9047621	0.000289029	-4.280243074
hsa-miR-510-3p	4.791487728	5.201627647	10.88159182	0.000291577	-4.291022862
hsa-miR-144-5p	-1.714221228	3.179037869	-10.87300617	0.000292528	-4.295022697
hsa-miR-4487	-1.921951817	2.880756636	-10.8611904	0.000293843	-4.300532167
hsa-miR-5010-5p	-3.477297583	3.724617786	-10.85742256	0.000294264	-4.302290212
hsa-miR-4721	-8.497909006	8.023690297	-10.83232262	0.000297086	-4.314016157
hsa-miR-99a-5p	4.235862622	8.157697922	10.77470619	0.000303693	-4.341028694
hsa-miR-4281	-5.656378633	12.63842999	-10.76585417	0.000304724	-4.345190719
hsa-miR-4513	-3.377142678	3.534499456	-10.74698727	0.000306935	-4.354072133
hsa-miR-6790-3p	5.088246944	5.312796961	10.697402	0.000312842	-4.377483171
hsa-miR-6746-5p	-3.647884978	4.118813189	-10.67446577	0.000315622	-4.388346317
hsa-miR-6763-3p	4.135986678	5.930861572	10.58715573	0.000326484	-4.429897528
hsa-miR-1260a	-4.339358267	4.8399939	-10.57562293	0.000327953	-4.435409773
hsa-miR-378c	2.172121133	3.634620261	10.50226388	0.000337489	-4.470603597

hsa-miR-1914-3p	6.198880161	11.95713511	10.49644124	0.000338261	-4.473406737
hsa-miR-106b-5p	-5.687222906	5.265740636	-10.48467126	0.000339826	-4.479077449
hur_5	-6.577574944	5.836425356	-10.47309986	0.000341375	-4.484658236
hsa-miR-1306-3p	7.944906922	8.750746194	10.41263396	0.000349607	-4.513913527
hsa-miR-4478	-7.658305572	7.035569253	-10.4055371	0.000350589	-4.517357507
hsa-miR-3937	-1.555235361	6.142494108	-10.39121266	0.000352582	-4.524315519
hsa-miR-4793-3p	-2.890552389	3.346302239	-10.32787464	0.000361561	-4.555188218
hsa-miR-7152-3p	5.477226706	8.554258714	10.30215015	0.000365288	-4.567777004
hsa-miR-770-5p	2.918976372	3.491677553	10.28891291	0.000367224	-4.574266219
hsa-miR-1343-5p	2.261049694	7.569486719	10.26217791	0.000371173	-4.587395887
hsa-miR-6861-5p	2.231422333	6.591739622	10.21839231	0.000377754	-4.608967472
hsa-miR-206	-3.869800733	5.243982733	-10.19031518	0.00038205	-4.622844949
hsa-miR-6782-5p	4.104202056	4.7603624	10.15331056	0.000387803	-4.641188859
hsa-miR-3976	6.379941592	6.763185024	10.14648023	0.000388876	-4.644581514
hsa-miR-194-5p	-1.448772494	3.156996992	-10.07548937	0.000400252	-4.679968015
hsa-miR-6887-5p	-6.984856522	6.395211961	-10.00077532	0.00041267	-4.717458908
hsa-miR-7847-3p	-3.466319189	8.861315211	-9.992675734	0.000414044	-4.721538661
hsa-miR-3154	3.199023306	6.461647714	9.981946246	0.000415873	-4.726947773
hsa-miR-378i	-5.294816878	8.107833644	-9.952905292	0.000420875	-4.741615161
hsa-miR-6510-5p	2.362282156	12.97059673	9.947479091	0.000421817	-4.744360066
hsa-miR-6760-3p	5.453198989	4.9802347	9.931616404	0.000424588	-4.752392271
hsa-miR-3679-5p	-3.818635178	9.606731767	-9.92870485	0.000425099	-4.753867839
hsa-miR-6872-3p	-3.750267439	4.187210331	-9.878384949	0.000434049	-4.779432735
hsa-miR-3654	-5.067655222	4.428614261	-9.864550564	0.000436551	-4.78648218
hsa-miR-939-5p	4.104485706	3.883775269	9.861156328	0.000437167	-4.78821313
hsa-miR-1307-5p	2.938177917	4.123011219	9.830078363	0.00044286	-4.804087309
hsa-miR-140-5p	-7.6651035	10.58944753	-9.796990143	0.000449023	-4.821038892
hsa-miR-8485	-1.633001144	4.155781339	-9.786372507	0.000451022	-4.82648957
hsa-miR-6766-3p	2.737413328	7.945188331	9.711984818	0.000465342	-4.864829703
hsa-miR-6875-5p	5.038686622	6.898835083	9.651211761	0.000477457	-4.89635243
hsa-miR-362-5p	-1.7249617	3.394839639	-9.64582065	0.00047855	-4.899157521
hsa-miR-4481	4.345128856	8.803299517	9.634124823	0.000480933	-4.905247991
hsa-miR-365a-3p	-5.560761139	8.383117753	-9.630576258	0.000481658	-4.9070972
hsa-miR-4664-3p	4.499517089	5.420675272	9.61356919	0.000485155	-4.915968471
hsa-miR-4515	-4.724300083	4.306038508	-9.596628224	0.000488669	-4.924819504
hsa-miR-4306	-7.555773944	6.895968728	-9.58865272	0.000490335	-4.928991345
hsa-miR-4485-3p	-2.87383005	5.963677992	-9.587817566	0.000490509	-4.929428382
hsa-miR-6747-5p	-4.180917822	4.573870078	-9.582343483	0.000491657	-4.932293834
hsa-miR-127-3p	-7.2799689	6.716023822	-9.577297001	0.000492718	-4.934936778
hsa-miR-6743-5p	3.773241117	5.762549736	9.547329361	0.000499075	-4.950657623
hsa-miR-663a	-4.499030078	4.691250356	-9.543291127	0.000499939	-4.952779493
hsa-miR-513b-5p	-5.994026761	5.595981903	-9.514774665	0.000506095	-4.967786641
hsa-miR-1288-3p	4.223639089	5.818048106	9.511561532	0.000506795	-4.969480159
hsa-miR-23a-3p	-14.74406994	11.95392383	-9.492205856	0.000511033	-4.979692816
hsa-miR-1587	4.106275928	14.35755204	9.488857449	0.000511771	-4.981461463
hsa-miR-4787-3p	-3.355420133	3.577468489	-9.445810717	0.000521372	-5.00424968
hsa-miR-7977	3.772167083	5.832769908	9.39826307	0.000532234	-5.029530597
hsa-miR-150-5p	-3.670413761	5.883567008	-9.376734586	0.000537244	-5.041015447
hsa-miR-3934-5p	-2.86166565	5.976586925	-9.368862561	0.00053909	-5.045220933
hsa-miR-4488	2.192710867	3.736546367	9.368569718	0.000539159	-5.04537744
hsa-miR-1275	4.831042833	13.51216758	9.361398574	0.000540847	-5.049211394
hsa-miR-4317	-3.444839706	5.595824086	-9.358745054	0.000541474	-5.050630738
hsa-miR-6129	3.75996905	5.889200575	9.349870339	0.000543576	-5.055380397

hsa-miR-6511b-5p	3.714851467	4.923044861	9.311754769	0.000552718	-5.075826082
hsa-miR-204-3p	4.734130722	5.49783675	9.305738529	0.000554178	-5.079060203
hsa-miR-194-3p	5.030205433	5.351124122	9.301241724	0.000555273	-5.081478766
hsa-miR-550a-5p	4.010490028	6.190930086	9.291039085	0.000557766	-5.08697009
hsa-miR-34b-5p	-2.358342178	3.822460983	-9.219272643	0.000575696	-5.12575171
hsa-miR-4701-5p	-2.26445675	2.912647531	-9.198776253	0.000580946	-5.136877846
hsa-miR-6075	-2.082700278	2.915037733	-9.187159155	0.000583948	-5.14319396
hsa-miR-572	2.839758289	6.318410856	9.181212168	0.000585492	-5.14643008
hsa-miR-1229-5p	2.947924783	12.97848966	9.17922324	0.00058601	-5.1475128
hsa-miR-181b-5p	-4.229699617	6.994059003	-9.161114264	0.000590747	-5.15738062
hsa-miR-920	-3.462143517	3.906523286	-9.157962671	0.000591576	-5.159099765
hsa-miR-6806-5p	-1.608167294	2.655458886	-9.068882197	0.000615618	-5.207914091
hsa-miR-543	3.364395217	4.401153908	9.027250109	0.000627264	-5.23087612
hsa-miR-3648	-2.242601322	7.197992544	-9.024533404	0.000628033	-5.232377824
hsa-miR-1260b	-4.272524789	4.424093917	-9.015198743	0.000630685	-5.237540817
hsa-miR-5684	3.918908717	5.751188092	9.010313195	0.000632079	-5.240244929
hsa-miR-20a-5p	-3.760302156	5.597861872	-9.009708214	0.000632252	-5.240579873
hsa-miR-642a-3p	1.429648622	15.82526261	8.965430584	0.000645059	-5.265149053
hsa-miR-497-5p	-6.000204056	8.740377878	-8.901118124	0.000664233	-5.301030438
hsa-miR-6076	2.977084172	9.865209092	8.866082223	0.000674972	-5.320675883
hsa-miR-6752-5p	2.174390756	10.53499644	8.818014049	0.000690055	-5.347742484
hsa-miR-6865-5p	-1.734662606	5.477110747	-8.816730359	0.000690463	-5.348467126
hsa-miR-6512-5p	1.9593823	7.198999928	8.784122599	0.000700937	-5.366905965
hsa-miR-1229-3p	-1.865327272	2.879091847	-8.748758365	0.000712518	-5.38697294
hsa-miR-6758-5p	4.198691133	5.86117195	8.744202395	0.000714027	-5.389563442
hsa-miR-1208	-2.826734603	3.295373274	-8.66528206	0.000740796	-5.434629763
hsa-miR-4539	-3.077379189	3.735566839	-8.647192989	0.000747104	-5.445010902
hsa-miR-660-5p	-4.23646435	4.000563392	-8.643195119	0.000748507	-5.447307855
hsa-miR-6085	1.850760544	14.20077481	8.617710704	0.000757526	-5.461972098
hsa-miR-1-3p	-3.903586272	4.302102919	-8.606804215	0.000761427	-5.468259731
hsa-miR-670-5p	-2.513209333	3.076861122	-8.551468503	0.000781601	-5.500270803
hsa-miR-502-3p	-2.727154031	3.221100554	-8.516919473	0.000794529	-5.520350664
hsa-miR-4304	-2.149098044	2.967382767	-8.497648643	0.000801853	-5.531582347
hsa-miR-4743-5p	-3.8598675	4.242587972	-8.483838211	0.000807153	-5.539645464
hsa-miR-4451	-1.351534211	3.8469176	-8.460895427	0.000816053	-5.553066248
hsa-miR-6858-3p	4.019195256	4.02546355	8.447602165	0.000821265	-5.560857169
hsa-miR-374b-5p	-4.161189839	6.473641031	-8.433070473	0.000827008	-5.569386369
hsa-miR-6850-5p	2.394785672	9.652436992	8.398224059	0.000840983	-5.589892287
hsa-miR-4535	2.1191365	5.919388572	8.384088716	0.000846735	-5.598231961
hsa-miR-376a-3p	-3.834484522	6.498385639	-8.373599115	0.000851034	-5.60442875
hsa-miR-345-5p	5.303864978	7.395036222	8.36441609	0.000854819	-5.609859319
hsa-miR-513c-5p	-5.729763356	5.452084278	-8.34759103	0.000861809	-5.619822872
hsa-miR-6086	2.506614328	9.574056253	8.323409924	0.000871977	-5.634173739
hsa-miR-145-5p	-4.396319578	8.323963794	-8.312481911	0.00087662	-5.640671337
hsa-miR-4419b	4.447541239	7.741635425	8.312095572	0.000876785	-5.640901185
hsa-miR-5190	-3.184887528	3.413617636	-8.30734857	0.000878811	-5.643726136
hsa-miR-574-5p	-7.351145783	6.996403853	-8.282425112	0.000889548	-5.658581588
hsa-miR-31-5p	-4.181668583	6.486330386	-8.274061476	0.000893187	-5.663575525
hsa-miR-4485-5p	-4.515510978	8.3010595	-8.258666756	0.000899932	-5.672779396
hsa-let-7f-1-3p	2.741813661	4.642704392	8.237536973	0.000909292	-5.685436714
hsa-miR-6856-5p	2.336099861	5.157507853	8.231049706	0.00091219	-5.689328512
hsa-miR-299-5p	-1.696986369	3.357276035	-8.221608535	0.000916427	-5.694997231
hsa-miR-4640-5p	2.637753483	4.004666681	8.212252333	0.00092065	-5.700620599

hsa-miR-6728-5p	-2.937759322	8.044359089	-8.16376891	0.000942918	-5.729851372
hsa-miR-6812-5p	2.616424472	11.03101505	8.148903644	0.000949877	-5.738844293
hsa-miR-23c	3.754610411	5.023080217	8.129202462	0.000959197	-5.750785021
hsa-miR-152-3p	-5.366644456	7.901215822	-8.124207676	0.000961578	-5.753816364
hsa-miR-3132	3.005238922	6.324771922	8.117302774	0.000964881	-5.758009658
hsa-miR-6886-3p	2.515036617	3.039802825	8.110933958	0.00096794	-5.761880171
hsa-miR-6767-5p	3.649155556	7.693237694	8.104578551	0.000971004	-5.765745198
hsa-miR-125b-1-3p	8.101040472	7.962385858	8.096098935	0.000975111	-5.770906206
hsa-miR-363-3p	-2.871219725	4.554193115	-8.084182668	0.000980918	-5.778166916
hsa-miR-331-3p	-3.481406728	5.897047853	-8.071093773	0.000987346	-5.786152956
hsa-miR-619-5p	-3.402530906	3.585361925	-8.070394035	0.000987691	-5.786580213
hsa-miR-7152-5p	2.710723244	3.351957367	8.047477862	0.000999074	-5.800590727
hsa-miR-4773	2.208130183	5.939154264	8.024785399	0.001010504	-5.814498985
hsa-miR-2467-3p	-1.732825978	2.769293817	-8.015772549	0.001015088	-5.820032544
hsa-miR-3180-3p	3.761328278	5.891304506	7.986292533	0.001030261	-5.838170328
hsa-miR-4433a-5p	4.436023256	8.537746156	7.962727319	0.001042589	-5.852711177
hsa-miR-92a-3p	2.653037772	5.476405725	7.956725392	0.001045757	-5.856420661
hsa-miR-6734-5p	2.37795975	8.156764342	7.945603035	0.001051661	-5.863301294
hsa-miR-3161	2.457567583	4.054566858	7.939041972	0.001055162	-5.867364117
hsa-miR-1225-5p	2.830414078	15.15955157	7.916668483	0.00106721	-5.881240598
hsa-miR-936	4.565164883	4.847012519	7.915456767	0.001067867	-5.881993105
hsa-miR-6732-5p	-1.93373095	2.919572192	-7.894234368	0.001079459	-5.895189099
hsa-miR-15b-5p	-7.4728635	10.60530807	-7.882496309	0.001085937	-5.902501071
hsa-miR-5008-5p	-3.735214311	4.051248383	-7.8725302	0.001091474	-5.9087167
hsa-miR-483-5p	-3.658746189	10.6110784	-7.851941738	0.001103022	-5.921578993
hsa-miR-21-3p	-1.690117761	3.419279403	-7.829491122	0.001115786	-5.935638159
hsa-miR-4430	-1.760804106	6.386114164	-7.795247547	0.001135604	-5.957150099
hsa-miR-6797-5p	-1.542767017	3.980630414	-7.787663385	0.001140051	-5.96192561
hsa-miR-143-3p	-2.558538	3.261973294	-7.782052333	0.001143355	-5.965461316
hsa-miR-451b	-1.128419267	3.390172544	-7.741171932	0.001167782	-5.99128843
hsa-miR-6883-5p	-4.43662585	4.499167419	-7.686118431	0.001201695	-6.026257305
hsa-miR-181c-5p	-5.975134372	4.699503236	-7.672240907	0.001210432	-6.035106261
hsa-miR-584-5p	-3.596854811	3.660258856	-7.629821368	0.001237626	-6.062241043
hsa-miR-381-3p	-2.397615922	4.640325761	-7.618992339	0.001244687	-6.069189007
hsa-miR-1227-5p	2.879127489	8.670252217	7.580373988	0.001270273	-6.09403636
hsa-miR-4800-5p	4.401130706	13.14687387	7.552932598	0.001288844	-6.1117588
hsa-miR-28-5p	-2.831242669	4.643977949	-7.551649053	0.001289721	-6.112589108
hsa-miR-6769a-5p	3.595697333	7.556864861	7.543775924	0.001295115	-6.117684789
hsa-miR-933	2.671463556	4.775925989	7.53420969	0.001301706	-6.123882454
hsa-miR-2861	-3.804778167	9.562150567	-7.533848952	0.001301955	-6.124116297
hsa-miR-376c-3p	-4.708504289	7.436883217	-7.530281774	0.001304424	-6.12642919
hsa-miR-30a-5p	-5.044048011	8.378275917	-7.523879725	0.001308869	-6.130582527
hsa-miR-6795-3p	5.268610206	5.204356153	7.504500016	0.001322438	-6.143173692
hsa-miR-1246	5.908224511	16.39398816	7.48364272	0.001337234	-6.156756127
hsa-miR-4444	2.955729772	4.586963775	7.482430109	0.0013381	-6.157546788
hsa-miR-550a-3-5p	3.645485844	6.118821972	7.481591781	0.001338699	-6.15809347
hsa-miR-23a-5p	-1.483940011	4.207013861	-7.480687598	0.001339346	-6.158683155
hsa-miR-19b-3p	-5.130085539	7.957249536	-7.476133631	0.00134261	-6.161654067
hsa-miR-187-5p	1.971193028	3.696453758	7.454745045	0.001358069	-6.175628347
hsa-miR-6798-5p	-3.523095717	4.015157019	-7.448902784	0.001362329	-6.179451383
hsa-miR-1304-3p	3.834673844	6.042140022	7.412674808	0.001389114	-6.203215668
hsa-miR-30b-5p	-4.687357517	6.959878219	-7.379863209	0.00141393	-6.22482493
hsa-miR-495-3p	-2.768144914	4.662560504	-7.351617659	0.001435727	-6.243492974

hsa-miR-29b-3p	-3.614460994	5.839012197	-7.330269168	0.001452474	-6.257643314
hsa-miR-5196-5p	4.013469994	5.964384564	7.283985859	0.001489608	-6.288442247
hsa-miR-4324	-4.122601361	6.574924542	-7.271188483	0.001500079	-6.296987605
hsa-miR-1271-5p	-3.057933239	3.332735242	-7.240653602	0.001525429	-6.317428875
hsa-miR-4689	-1.428731444	5.744567711	-7.240453145	0.001525597	-6.317563311
hsa-miR-4484	-2.239304039	6.219839081	-7.239944599	0.001526024	-6.31790438
hsa-miR-6795-5p	-2.749253817	3.218703947	-7.124975748	0.001626329	-6.395538087
hsa-miR-223-3p	-5.561533161	8.395994853	-7.106889084	0.001642832	-6.407847682
hsa-miR-148b-3p	-3.844370844	6.175864122	-7.098657163	0.001650411	-6.413459008
hsa-miR-622	3.847635228	5.922856225	7.093550138	0.001655134	-6.416942998
hsa-miR-6716-3p	-1.637434911	4.067386233	-7.083567376	0.001664414	-6.423759314
hsa-miR-3124-5p	4.720545317	5.461360003	7.075571548	0.001671893	-6.429224782
hsa-miR-4442	3.623615767	13.13949067	7.013660809	0.001731209	-6.471720353
hsa-miR-342-3p	-6.296293317	9.264991314	-7.010864433	0.001733949	-6.473647229
hsa-miR-4463	-1.66615435	5.800761897	-6.983726654	0.001760808	-6.492380476
hsa-miR-324-5p	-3.233792569	5.240083554	-6.96392014	0.001780732	-6.50609157
hsa-miR-1238-3p	3.462483883	6.380602731	6.945787149	0.001799214	-6.518672879
hsa-miR-2276-3p	-1.995853144	6.64627325	-6.93999902	0.001805163	-6.522694685
hsa-miR-890	-4.008272111	4.390357606	-6.904799212	0.001841859	-6.547213471
hsa-miR-125a-5p	-6.363909944	6.357697572	-6.903409461	0.001843327	-6.548183661
hsa-miR-3665	-3.631872161	8.385000331	-6.889177329	0.001858436	-6.55812857
hsa-miR-4659a-3p	-3.6771337	4.202489411	-6.847037408	0.001904068	-6.587675412
hsa-miR-4313	-3.226446367	3.664581556	-6.816229562	0.001938294	-6.609372765
hsa-miR-1273g-3p	2.2617997	15.97363265	6.782479518	0.001976652	-6.633236142
hsa-miR-4769-3p	1.85264805	5.577823231	6.77916703	0.001980466	-6.635583593
hsa-miR-3907	-1.590443278	4.321762456	-6.706629376	0.002066287	-6.687228777
hsa-miR-128-3p	-3.2456183	5.1598385	-6.6454379	0.002142225	-6.731156425
hsa-miR-4649-3p	-2.288110828	2.870283331	-6.615493648	0.002180623	-6.752774277
hsa-miR-4286	-3.240850561	5.901473292	-6.568182348	0.002243012	-6.787094682
hsa-miR-151a-3p	-4.3826325	7.323538789	-6.550386417	0.00226704	-6.800056698
hsa-miR-4291	-4.422882828	7.472527186	-6.542530873	0.002277746	-6.805787626
hsa-miR-1321	2.527418483	4.007679058	6.527300744	0.002298678	-6.816914682
hsa-miR-6894-5p	2.338494272	3.426464086	6.526852925	0.002299297	-6.817242178
hsa-miR-8071	-4.325874239	4.767967397	-6.522430832	0.002305421	-6.8204771
hsa-miR-4730	-1.365354506	3.658206503	-6.520865411	0.002307594	-6.821622693
hsa-miR-6741-5p	1.570577311	6.707410039	6.480668974	0.002364241	-6.851116205
hsa-miR-22-5p	-2.264044414	4.204078504	-6.450311242	0.002408146	-6.873489868
hsa-miR-3926	-2.403354733	4.693272228	-6.435730702	0.002429586	-6.884266251
hsa-miR-6826-5p	2.567889122	8.79592175	6.433758498	0.002432504	-6.88572542
hsa-miR-7159-5p	-2.657159789	5.206341878	-6.426932589	0.002442635	-6.890778497
hsa-miR-4314	-1.398312844	4.609642522	-6.412493115	0.002464237	-6.901482137
hsa-miR-3194-5p	2.332331939	8.072184442	6.394370824	0.002491678	-6.914943512
hsa-miR-5787	2.335644017	16.07184377	6.381018212	0.002512134	-6.924881763
hsa-miR-2116-3p	3.048383906	5.005944786	6.349877943	0.002560641	-6.948124911
hsa-miR-4698	6.0583609	12.03170438	6.306002789	0.002630931	-6.981030407
hsa-miR-6880-3p	3.159991006	3.829323931	6.297407301	0.002644974	-6.987498499
hsa-miR-6877-3p	-3.925536794	4.194468869	-6.271457655	0.002687928	-7.00706884
hsa-miR-4476	-1.575072672	5.593398736	-6.270572769	0.002689408	-7.00773734
hsa-miR-139-3p	2.312266322	7.6770941	6.205660196	0.002800698	-7.056984569
hsa-miR-6889-3p	2.7372668	5.732444117	6.203928544	0.002803743	-7.058303971
hsa-miR-500a-5p	2.644969522	4.837920956	6.202314606	0.002806584	-7.05953395
hsa-miR-4749-3p	3.165628617	6.137547792	6.19398215	0.002821309	-7.065888175
hsa-miR-3202	-1.547337611	6.107620072	-6.188093087	0.002831772	-7.070383228

hsa-miR-1469	-2.166731811	4.87443645	-6.150577251	0.002899548	-7.09909921
hsa-miR-659-3p	2.53972875	7.065876236	6.101627658	0.002990966	-7.136777863
hsa-miR-4259	1.9618606	4.450242189	6.089202042	0.003014726	-7.146380673
hsa-miR-4499	-2.3933176	7.561340122	-6.08210009	0.003028409	-7.151876224
hsa-miR-1234-3p	2.556783022	7.413774744	6.076256216	0.003039725	-7.156402085
hsa-miR-590-5p	-1.264340122	1.936572294	-6.074223399	0.003043674	-7.157977236
hsa-miR-3667-5p	-1.653156017	6.967652292	-6.067325944	0.003057117	-7.163324923
hsa-miR-505-3p	-2.890101189	4.930996994	-6.008904699	0.003173919	-7.208813844
hsa-miR-6794-5p	-2.659165278	8.165538233	-5.990215587	0.003212422	-7.223439649
hsa-miR-6515-3p	1.654980239	6.971232286	5.97370427	0.003246911	-7.236391102
hsa-miR-1228-3p	2.685580589	8.104062989	5.972169975	0.003250139	-7.237596032
hsa-miR-6780a-5p	-2.584579267	4.455939111	-5.971815145	0.003250886	-7.237874726
hsa-miR-638	2.241683533	11.20852044	5.942719643	0.003312852	-7.26077162
hsa-miR-4455	-2.995554044	5.999282983	-5.919148196	0.003364106	-7.279385962
hsa-miR-151a-5p	-7.715610772	6.440191992	-5.890382687	0.003427963	-7.302180903
hsa-miR-3131	-1.880130761	6.902290553	-5.844234456	0.003533507	-7.338932986
hsa-miR-3653-3p	-4.444676556	7.943800683	-5.83593399	0.003552906	-7.345567398
hsa-miR-196b-5p	-3.858799039	5.961596586	-5.831235537	0.003563945	-7.349326042
hsa-miR-6870-3p	2.47703475	4.017819725	5.816643415	0.003598492	-7.361014404
hsa-miR-361-5p	-4.220615394	6.828201375	-5.814254639	0.003604186	-7.362929996
hsa-miR-6737-3p	-2.756251089	3.316294261	-5.809263468	0.003616119	-7.366934456
hsa-miR-30c-5p	-4.733178456	7.307846106	-5.797343098	0.003644811	-7.376509089
hsa-miR-602	4.676586128	5.436119492	5.79372482	0.003653575	-7.37941837
hsa-let-7b-3p	1.833142589	4.420899206	5.766809077	0.003719575	-7.401104299
hsa-miR-4660	4.117063694	5.575329864	5.720121515	0.003837518	-7.438906577
hsa-miR-1290	-7.887376111	7.143363478	-5.687724029	0.003922027	-7.465278322
hsa-miR-3117-3p	3.7644817	4.949428867	5.682962351	0.003934637	-7.469164078
hsa-miR-487b-3p	-2.642409017	4.698992647	-5.629922263	0.004078472	-7.512616901
hsa-miR-30e-3p	-2.537795564	4.556333818	-5.626479817	0.004088025	-7.51544791
hsa-miR-4672	-2.233703022	4.973028744	-5.621787299	0.004101091	-7.519309087
hsa-miR-345-3p	-1.879358183	5.743348514	-5.581167859	0.004216332	-7.552835324
hsa-miR-328-5p	-2.980734689	9.576005289	-5.572481472	0.004241482	-7.560028921
hsa-miR-6727-5p	2.366385733	11.55044831	5.506296524	0.004439189	-7.615120613
hsa-miR-324-3p	-4.019502522	7.3941582	-5.478063282	0.004526908	-7.63877391
hsa-miR-6812-3p	-1.975218561	2.918255319	-5.473208417	0.004542202	-7.642850467
hsa-miR-451a	-14.09717549	16.64376933	-5.46552263	0.004566542	-7.649309665
hsa-miR-4710	-2.556633144	3.243998839	-5.46177799	0.004578458	-7.652459163
hsa-miR-6786-5p	-2.735295367	8.101795028	-5.416531431	0.004725452	-7.690643133
hsa-miR-449c-3p	2.599015217	4.149152392	5.413624936	0.004735088	-7.693104094
hsa-miR-197-3p	-2.356793028	4.255936731	-5.408891995	0.00475083	-7.69711364
hsa-miR-452-5p	-1.893786239	2.942615914	-5.407393084	0.004755829	-7.698384
hsa-miR-382-5p	-2.212881883	4.631820925	-5.305892441	0.005109601	-7.785023782
hsa-miR-7110-5p	3.265157622	8.427292972	5.304105761	0.005116108	-7.786559822
hsa-miR-193a-5p	-2.753911011	5.60531625	-5.303454681	0.005118481	-7.787119662
hsa-miR-5096	3.236178294	5.184998442	5.275076082	0.005223241	-7.811570728
hsa-miR-6083	2.631975956	5.408261794	5.271854428	0.005235297	-7.814352626
hsa-miR-601	-1.867453456	6.285614828	-5.246360297	0.005331891	-7.83641093
hsa-miR-4422	-1.328185106	4.810721014	-5.201901791	0.005505535	-7.875066231
hsa-miR-3180-5p	2.160104661	4.981372047	5.18171964	0.005586609	-7.892693535
hsa-miR-7106-5p	-1.788496744	6.231876939	-5.155609459	0.005693643	-7.915572606
hsa-miR-6724-5p	2.650883717	11.01223938	5.149418074	0.005719385	-7.92101013
hsa-miR-889-3p	1.905028411	3.631703194	5.144235606	0.00574104	-7.92556522
hsa-miR-664b-5p	-4.626627622	4.788223233	-5.13526707	0.005778749	-7.933455894

hsa-miR-4687-3p	2.87932605	16.9908712	5.101869032	0.005921827	-7.962927837
hsa-miR-7107-5p	-5.565481111	13.15830403	-5.098981643	0.005934396	-7.96548232
hsa-miR-4433a-3p	-1.436901756	6.419946461	-5.062662174	0.006095274	-7.997703316
hsa-miR-5093	3.771261017	4.992890031	5.060724878	0.006104002	-7.999426645
hsa-miR-3190-5p	2.227554728	4.356744814	5.059159058	0.006111068	-8.000819872
hsa-miR-6808-5p	2.308457211	6.784275772	5.04696215	0.006166442	-8.011682933
hsa-miR-181a-3p	-2.581273975	4.420406557	-5.030689735	0.006241262	-8.026205029
hsa-let-7b-5p	-25.01169358	17.23864665	-4.990859936	0.006429062	-8.061892188
hsa-miR-144-3p	-2.246704806	3.737475047	-4.968566469	0.006537145	-8.08195517
hsa-miR-1273c	5.084719078	10.84299931	4.956087887	0.006598598	-8.093213037
hsa-miR-4446-3p	-2.051152083	4.320952797	-4.954526215	0.006606338	-8.094623347
hsa-miR-218-5p	-2.920309808	5.026689924	-4.953062279	0.006613603	-8.095945677
hsa-miR-361-3p	-2.564381192	4.667834443	-4.938209501	0.006687859	-8.109377344
hsa-miR-30e-5p	-2.858724717	5.161911408	-4.935722699	0.00670039	-8.111628986
hsa-miR-4644	-1.367306933	4.830503444	-4.927553846	0.006741751	-8.119030983
hsa-miR-766-3p	-1.295584378	3.686855133	-4.915973155	0.006800914	-8.129539306
hsa-miR-1183	1.78077025	6.953131564	4.912988422	0.006816263	-8.132250466
hsa-miR-4522	-4.762532189	4.980638211	-4.912146723	0.0068206	-8.133015226
hsa-miR-6833-5p	2.607737022	10.22766053	4.889554144	0.006938236	-8.153576983
hsa-miR-664b-3p	-1.071556828	2.307673019	-4.849738266	0.007151548	-8.189975754
hsa-miR-6749-5p	1.207120278	17.22676758	4.833222751	0.007242337	-8.205134792
hsa-miR-132-3p	-3.373475364	3.922907065	-4.832095671	0.007248583	-8.206170608
hsa-miR-4668-5p	-2.69812	5.375500667	-4.775753062	0.007569239	-8.258164821
hsa-miR-494-3p	-13.11455442	11.39698272	-4.707036796	0.007983681	-8.32214995
hsa-miR-4788	2.862576067	13.47102072	4.695812271	0.00805394	-8.332661929
hsa-miR-424-3p	-1.420793033	3.479106833	-4.672979919	0.008199159	-8.354097422
hsa-miR-4270	-4.103599172	11.02256299	-4.665420188	0.008247932	-8.361210228
hsa-miR-423-3p	1.846381894	3.828318225	4.653823304	0.008323428	-8.372136607
hsa-miR-29a-3p	-7.710169639	12.23644271	-4.651678144	0.008337484	-8.374159746
hsa-miR-3162-3p	-1.429072	3.985087267	-4.629845718	0.008482168	-8.394785981
hsa-miR-30c-2-3p	-1.0980853	4.620770333	-4.608395716	0.008627259	-8.415114399
hsa-miR-6516-5p	-1.067664767	2.288814406	-4.581876618	0.008810771	-8.440334206
hsa-miR-4261	-1.278118106	3.297993997	-4.575203337	0.008857685	-8.446695792
hsa-miR-3591-3p	-1.124131622	3.115778633	-4.573338642	0.008870847	-8.44847449
hsa-miR-6800-3p	-2.152709822	3.035208672	-4.57017227	0.008893251	-8.451495934
hsa-miR-6847-5p	1.829883406	6.758352692	4.50844521	0.009343853	-8.510675492
hsa-miR-4497	-1.973455661	6.526016286	-4.444005606	0.009843731	-8.573023755
hsa-miR-4769-5p	-1.63143385	5.140056925	-4.440766454	0.009869692	-8.576173215
hsa-miR-3125	-1.938152606	5.007831097	-4.427052074	0.009980519	-8.589524286
hsa-miR-4778-5p	-2.976306711	8.990021728	-4.417513899	0.010058475	-8.598825491
hsa-miR-3652	-2.619478894	6.890469814	-4.381831221	0.010356643	-8.633736302
hsa-miR-484	-1.440945144	3.765328828	-4.376039007	0.010406034	-8.639420339
hsa-miR-6824-5p	1.848392706	6.601857658	4.34669054	0.01066066	-8.668294441
hsa-miR-6124	2.926752444	14.86435028	4.333318929	0.010779135	-8.681490884
hsa-miR-6870-5p	3.848571383	4.873067069	4.319580744	0.010902499	-8.695075883
hsa-miR-6784-5p	-1.510995244	5.368835456	-4.290903687	0.011165483	-8.723520906
hsa-miR-330-3p	-1.428404447	3.327217435	-4.280770972	0.011260211	-8.733600074
hsa-miR-8064	-1.507798022	5.281317756	-4.255862901	0.011497174	-8.758439837
hsa-miR-378d	-2.581632911	4.1285823	-4.253290345	0.011521985	-8.761010476
hsa-miR-3135b	-4.114437928	7.739811581	-4.218130434	0.011867571	-8.796240918
hsa-miR-6788-5p	1.795543056	6.591606972	4.208255317	0.011966847	-8.806168344
hsa-miR-6738-5p	1.605576761	6.099870219	4.191324814	0.012139363	-8.82322179
hsa-miR-4758-5p	6.610437183	11.81201393	4.158590213	0.012481396	-8.856313581

hsa-miR-142-3p	-2.005220017	3.569225108	-4.155911664	0.012509888	-8.859028346
hsa-miR-30a-3p	-2.510662619	4.762867529	-4.135492385	0.012729653	-8.879758539
hsa-miR-6087	1.451093611	16.72157425	4.107105698	0.013042852	-8.90868001
hsa-miR-6730-3p	-1.058727539	2.308057803	-4.092298203	0.013209863	-8.923813942
hsa-miR-188-5p	5.184627283	13.86146491	4.065371054	0.013520134	-8.951418371
hsa-miR-3190-3p	-1.435745894	2.283817631	-4.065306864	0.013520884	-8.951484304
hsa-miR-377-3p	-2.083955044	4.202727592	-4.022971542	0.014026434	-8.9951039
hsa-miR-4428	4.179939356	14.26152966	4.020058215	0.014062042	-8.99811548
hsa-miR-374c-5p	-2.066745369	3.961800957	-4.017577493	0.014092447	-9.00068087
hsa-miR-19a-3p	-2.335140461	4.202983111	-4.016350124	0.01410752	-9.00195047
hsa-miR-6817-5p	-3.088458606	3.500372514	-3.999732461	0.014313484	-9.0191622
hsa-miR-4755-3p	-6.871539383	5.877333247	-3.973947992	0.014640178	-9.045950706
hsa-miR-642a-5p	-1.114709622	2.329688211	-3.917784659	0.015382914	-9.104648523
hsa-miR-501-5p	-1.921258017	2.751539064	-3.891213212	0.015749837	-9.132585643
hsa-miR-1973	-2.9098195	5.195290206	-3.875392798	0.015973225	-9.149270142
hsa-miR-425-3p	1.869361661	4.405451003	3.866200665	0.016104744	-9.158981836
hsa-let-7d-5p	1.549151417	13.51216104	3.86562018	0.016113092	-9.159595564
hsa-miR-410-3p	-2.243542983	4.234315283	-3.836727556	0.01653517	-9.190207712
hsa-miR-23b-5p	-2.273415017	4.382759903	-3.797465867	0.017129887	-9.232010446
hsa-miR-6509-5p	-1.128971878	2.400487889	-3.788347616	0.017271604	-9.241752599
hsa-miR-6891-3p	2.16510215	3.323264975	3.772770599	0.017516925	-9.258424933
hsa-miR-6511a-5p	-1.259049772	2.430842475	-3.771923168	0.017530389	-9.25933302
hsa-miR-193b-3p	-4.598413106	7.956054275	-3.731917874	0.018180129	-9.302327192
hsa-miR-630	2.479757983	11.64148037	3.706499273	0.018607746	-9.32977269
hsa-miR-340-5p	-2.299785889	4.297553231	-3.672241743	0.019202988	-9.366919318
hsa-miR-6757-3p	-1.155791278	2.371295006	-3.670087273	0.019241168	-9.369261528
hsa-miR-6736-5p	2.106782128	6.747389197	3.666342424	0.019307744	-9.373334407
hsa-miR-654-3p	2.309317728	7.718457764	3.660763436	0.019407433	-9.379406095
hsa-miR-4450	-1.286299539	3.791109086	-3.637862813	0.019823044	-9.404379448
hsa-miR-551b-5p	-1.251230306	2.435135081	-3.61604408	0.020228809	-9.428248262
hsa-miR-4436b-5p	-1.180232556	3.291158911	-3.572685173	0.021064559	-9.475899288
hsa-miR-96-5p	-3.69065925	4.187105419	-3.571192168	0.021094053	-9.477545256
hsa-miR-335-5p	-1.477778272	3.832791456	-3.537705085	0.021768521	-9.514553634
hsa-miR-1237-3p	2.182917722	5.735564228	3.535306806	0.021817791	-9.517210745
hsa-miR-3960	1.185793	19.1435595	3.531954873	0.021886872	-9.52092592
hsa-miR-656-3p	-1.219747181	2.427832332	-3.485723766	0.022866374	-9.57234389
hsa-miR-4417	-2.346914806	7.039087031	-3.427715967	0.024169105	-9.637325952
hsa-miR-202-3p	-2.395268633	5.206322489	-3.404233513	0.02472108	-9.663778946
hsa-miR-4725-5p	-1.801985128	2.865900925	-3.383420689	0.025222677	-9.687295401
hsa-miR-1207-5p	2.153687428	16.1452939	3.356769968	0.025882465	-9.717505084
hsa-miR-432-5p	-2.450913169	4.441391507	-3.326161126	0.026665299	-9.752335525
hsa-miR-221-5p	-2.139316344	3.670202606	-3.224817002	0.029461829	-9.868674128
hsa-miR-6765-3p	-1.74084065	2.767008781	-3.215063599	0.029748578	-9.879952496
hsa-miR-4682	-1.265143194	2.412292547	-3.209126118	0.02992473	-9.886825312
hsa-miR-4310	-1.420109589	2.560464761	-3.182641901	0.030725392	-9.917545938
hsa-miR-4290	-1.174063383	3.550186536	-3.174906815	0.030963919	-9.92653811
hsa-miR-154-5p	-2.390419978	4.522560969	-3.160803989	0.031404372	-9.942955831
hsa-miR-378f	-1.063864244	3.356661378	-3.12245027	0.03263952	-9.987754327
hsa-miR-424-5p	-2.868822222	4.797729778	-3.090068873	0.033726387	-10.02574574
hsa-miR-149-5p	-3.016498922	4.963047194	-3.071345095	0.034373971	-10.04778336
hsa-miR-6777-5p	-1.51615835	2.592336303	-3.03209209	0.035778895	-10.09414865
hsa-miR-4732-5p	-2.479504394	5.212653481	-3.012592482	0.036501449	-10.11726385
hsa-miR-197-5p	3.200023933	17.68067836	2.947495688	0.039038588	-10.19482151

hsa-miR-3666	-1.888857728	2.738170219	-2.925443942	0.03994375	-10.22122894
hsa-miR-539-5p	-1.664203706	2.639061081	-2.903559529	0.040866019	-10.24750227
hsa-miR-6745	-1.230238917	5.241725303	-2.876700052	0.042031626	-10.27983771
hsa-miR-5739	1.477240394	14.16584814	2.868797806	0.042381799	-10.28936958
hsa-miR-2277-3p	2.691674811	5.564641206	2.849871849	0.043234174	-10.3122325
hsa-miR-6751-3p	-1.089597356	2.22472185	-2.849672203	0.04324327	-10.31247394
hsa-miR-3605-5p	-1.589472933	4.880747022	-2.829366162	0.044179984	-10.33705745
hsa-miR-4746-5p	-3.153144906	5.738181831	-2.815317654	0.044841661	-10.35409701
hsa-miR-4534	-2.07352585	7.698543869	-2.793283511	0.045902459	-10.38087407
hsa-miR-135a-3p	3.745214756	10.99279318	2.776294949	0.046739981	-10.40156208
hsa-miR-3193	-1.160931406	2.332693853	-2.771104361	0.046999349	-10.40789031
hsa-miR-665	-1.51720975	4.125984103	-2.765581579	0.047277125	-10.41462728
hsa-miR-133b	-3.5049179	5.421413728	-2.747528909	0.048198286	-10.43667558
hsa-miR-4274	-1.456881906	4.905821597	-2.721036192	0.049587468	-10.46910526
hsa-miR-493-5p	-2.287147097	3.773569549	-2.719308382	0.049679644	-10.47122326
hsa-miR-16-5p	2.170767889	16.88339511	2.71062409	0.050145903	-10.48187421
hsa-miR-8055	2.135725294	4.263581103	2.696785863	0.050899222	-10.49886509
hsa-miR-181d-5p	-2.538257378	4.297525461	-2.637709622	0.054263433	-10.57165425
hsa-miR-103a-3p	1.973244744	13.41424119	2.590079669	0.057160346	-10.63062878
hsa-miR-3651	-3.67000075	6.337825503	-2.560085307	0.059074429	-10.66789321
hsa-miR-4673	2.095693339	4.945821564	2.546132394	0.059989452	-10.68526004
hsa-miR-1181	2.500142967	9.137710539	2.536424112	0.06063556	-10.6973554
hsa-miR-516a-5p	-2.193805356	7.681020067	-2.519153463	0.061804467	-10.71889589
hsa-miR-4284	-2.6820805	5.497560433	-2.510445514	0.062403458	-10.72976785
hsa-miR-204-5p	-3.561982417	3.289321575	-2.509711772	0.062454227	-10.73068427
hsa-miR-548u	-1.012854836	2.229566338	-2.507717443	0.062592456	-10.73317538
hsa-miR-6769b-5p	1.540029972	14.47720531	2.409843401	0.069818432	-10.85587722
hsa-miR-422a	-1.073480717	4.595438336	-2.356813148	0.074121095	-10.92269287
hsa-miR-3149	-2.069564789	4.341081994	-2.339551607	0.075584852	-10.94448618
hsa-miR-2392	-2.290728111	7.962027267	-2.285088386	0.080418287	-11.01337587
hsa-miR-6740-5p	1.822643206	13.53871809	2.281825499	0.080718571	-11.01750874
hsa-miR-1180-3p	-1.113218481	2.320411126	-2.278804976	0.080997658	-11.02133515
hsa-miR-378b	-1.271860039	3.699160247	-2.246940016	0.084007906	-11.06173158
hsa-miR-1972	-1.089730417	2.337734308	-2.210451559	0.087607745	-11.10804943
hsa-miR-4459	-12.04747893	17.61263984	-2.201728992	0.088493271	-11.11913004
hsa-miR-128-1-5p	-1.039824617	2.328149569	-2.154078257	0.093508018	-11.17970923
hsa-miR-1237-5p	-1.134691422	2.409096411	-2.13900335	0.095158823	-11.1988878
hsa-miR-1299	1.673350683	5.998179881	2.136457386	0.095440762	-11.20212734
hsa-miR-450a-5p	-1.278348167	2.979851356	-2.091323956	0.100593497	-11.25957481
hsa-miR-374a-5p	-4.091135425	5.822914799	-2.057435296	0.104661794	-11.30272263
hsa-miR-4489	-1.0722421	3.638283589	-2.043546694	0.106380488	-11.32040618
hsa-miR-122-5p	-1.068968772	2.359760264	-2.04214067	0.106556181	-11.32219634
hsa-miR-4745-5p	-1.4850441	5.379973283	-2.041643236	0.106618415	-11.32282967
hsa-miR-505-5p	-1.100862881	2.948720318	-2.015653201	0.109925447	-11.35591732
hsa-miR-6837-5p	-1.201963161	2.384091281	-1.999792636	0.111998003	-11.37610489
hsa-miR-3162-5p	1.529572433	14.27594134	1.981661545	0.114419074	-11.39917639
hsa-miR-6845-3p	-1.138672064	2.396086726	-1.922426777	0.122729437	-11.47448279
hsa-miR-3610	-1.2827429	5.554903556	-1.91698441	0.123524823	-11.4813948
hsa-miR-10a-3p	-1.251296283	2.434279231	-1.883559663	0.128532131	-11.52381208
hsa-miR-126-5p	-1.682893611	2.443331139	-1.865299298	0.13135869	-11.54695748
hsa-miR-6777-3p	-1.067819178	4.390580522	-1.854769719	0.133018562	-11.56029385
hsa-miR-379-5p	-1.225177617	3.015208492	-1.843843106	0.13476458	-11.57412465
hsa-miR-32-3p	-1.757429122	3.927906278	-1.736344348	0.153289788	-11.70962446

hsa-miR-6508-5p	-1.002711267	3.5252919	-1.718777424	0.156562423	-11.73164701
hsa-miR-6774-5p	-1.594938033	5.6418678	-1.705225568	0.159136738	-11.74860862
hsa-miR-769-5p	-1.03932995	3.033634064	-1.640570776	0.172036255	-11.82916236
hsa-miR-3120-3p	-1.798294083	3.663352353	-1.606728871	0.179212781	-11.87105183
hsa-miR-193a-3p	-1.549934411	3.407354261	-1.586902964	0.183558591	-11.89549382
hsa-miR-489-3p	-2.056781278	3.611053239	-1.531321554	0.196321657	-11.96358648
hsa-miR-1202	1.866189883	17.58024339	1.495825193	0.204937462	-12.00670749
hsa-miR-500a-3p	-1.589328222	3.659917583	-1.489357461	0.206547675	-12.01453122
hsa-miR-17-5p	-2.907048831	4.910890368	-1.448007195	0.217144453	-12.06429173
hsa-miR-136-3p	-1.209584278	3.166167211	-1.403092026	0.229265062	-12.11779697
hsa-miR-342-5p	-2.003067897	4.160990346	-1.354877049	0.243013866	-12.17453602
hsa-miR-29c-5p	-2.074076878	4.4129574	-1.308088276	0.257117553	-12.22883666
hsa-miR-224-5p	-2.021261167	4.045015606	-1.289228286	0.263021721	-12.25049649
hsa-miR-508-5p	1.828468589	5.090002594	1.143687394	0.313061066	-12.41261675
hsa-miR-24-1-5p	-1.014525606	2.876472814	-1.016050585	0.363916257	-12.54614835
hsa-miR-7-1-3p	-1.175208756	2.970157611	-1.006559584	0.367972258	-12.55570522
