# Supplementary material for: CircEYA3 aggravates intervertebral disc degeneration through the miR-196a-5p/EBF1 axis and NF-κB signaling
Source: Commun Biol. 2024 Mar 30;7:390. doi: 10.1038/s42003-024-06055-2 (PMC10981674; doi:10.1038/s42003-024-06055-2)
Supplement: Supplementary file 6 — Supplementary Data 4 [file 42003_2024_6055_MOESM6_ESM.pdf]

Supplementary data 4. The gene expression matrix of mRNAs

id	logFC	AveExpr	t	P.Value	B
GATA6	1.903094593	3.340851891	7.969930474	2.35E-07	6.579997149
ZNF185	-1.469973082	3.331197489	-7.324262344	7.75E-07	5.608391396
AOX1	-1.2017309	3.645737962	-6.501876635	3.84E-06	4.268489614
LMO2	1.920074763	6.225314063	6.191701683	7.18E-06	3.733362727
IL12RB2	-0.930468359	2.76538608	-5.970447523	1.13E-05	3.341926735
MUC1	-0.732111776	3.167682214	-5.905143644	1.30E-05	3.224879216
ZNF680	0.851353904	6.970636241	5.886755619	1.35E-05	3.191798642
ASPH	1.212288754	5.717972044	5.654258938	2.19E-05	2.768959016
ENPP2	-1.518038678	6.879514277	-5.594358486	2.49E-05	2.658673045
FOXF2	-1.394098208	6.345102329	-5.560832119	2.67E-05	2.596710839
PLA2G4A	0.77664466	9.748431096	5.505099112	3.01E-05	2.493338484
IGFBP3	3.236412271	6.258409648	5.384045205	3.89E-05	2.267254314
DUSP16	-0.617759676	4.911885093	-5.353736226	4.15E-05	2.210321443
EPS8	0.662737053	11.69527957	5.281178903	4.85E-05	2.073509572
LOC100288911	-0.914019794	2.571436637	-5.240368711	5.30E-05	1.996242793
EMILIN1	1.581173198	6.549549436	5.225754502	5.47E-05	1.968518905
SLC19A3	-0.765771452	3.333502242	-5.21698268	5.58E-05	1.951864631
DMKN	-1.30106913	5.557724541	-5.197447968	5.82E-05	1.914739133
MGST1	1.362571367	10.61045628	5.174093496	6.12E-05	1.870288241
PLAG1	0.792315951	3.979603924	5.165705078	6.23E-05	1.854305031
SCGB2A2	-2.179377349	3.409877616	-5.133119733	6.69E-05	1.792130721
AHR	1.096324214	7.728881368	5.124946848	6.80E-05	1.776515058
ZCCHC3	-0.743173968	5.861091428	-5.016796319	8.61E-05	1.569084653
HYAL1	-1.635539649	4.514924363	-4.970820876	9.52E-05	1.480471274
VAMP8	-1.572016478	5.804236351	-4.954329312	9.87E-05	1.448624205
PTRHD1	-0.898576282	7.075959654	-4.941475965	0.000101474	1.423780883
SGCE	-1.195489241	7.689809927	-4.91252922	0.000108109	1.36776183
ENPP4	0.69054358	2.490025318	4.903102576	0.000110364	1.349498266
MT1G	-1.937295532	10.67090267	-4.892632831	0.000112925	1.329201958
CYP1B1	1.515119454	11.21241374	4.874839444	0.000117416	1.294680024
C6orf52	-0.731391736	2.896657937	-4.845026068	0.000125354	1.236758717
FAM43B	-0.900995896	3.648344225	-4.827695575	0.000130217	1.203044441
TEX14	-1.189145873	3.098585964	-4.761986205	0.000150467	1.074924775
ZMAT3	0.724865542	5.449903817	4.75246771	0.000153656	1.056328437
CCT6B	-0.97279088	3.69385396	-4.715246299	0.000166797	0.983520894
B3GNT7	0.631789417	6.260100602	4.709801379	0.000168813	0.972858705
CYB5R2	-0.988509054	3.859760161	-4.66457853	0.000186543	0.884192538
TMEM27	-1.62963952	5.171874434	-4.66397295	0.000186793	0.883003881
OSCP1	-0.786596915	4.618541725	-4.635591164	0.000198891	0.827256512
CYB5D2	-0.767571088	5.01027136	-4.608731891	0.000211073	0.774431557
SMIM3	2.303470985	5.115883098	4.604924017	0.00021286	0.766937238
PSME1	0.876930278	5.051976138	4.603264431	0.000213644	0.763670584
ZNF260	0.850730936	5.117638999	4.570889355	0.000229532	0.699896554
SERTAD4-AS1	-0.809361466	5.469621265	-4.568500114	0.000230751	0.695186509
ITM2C	-0.83816313	8.393561115	-4.551637327	0.000239543	0.661930117
ATAD2	0.620744405	5.660868435	4.545158996	0.000243011	0.649147334
CLDN11	-0.667447116	2.846593544	-4.529124078	0.000251813	0.617492888
NMRAL1	0.903491496	4.34206308	4.525061117	0.000254095	0.609468863
DLX3	1.147831995	2.921977177	4.505410738	0.000265427	0.570642089
GSTZ1	-0.746465967	4.08769536	-4.495700833	0.000271215	0.551445121
TFPI	2.055735683	6.303031304	4.487722459	0.000276065	0.535665935

RCL1	-0.86389911	6.802186619	-4.486251099	0.00027697	0.53275542
C4BPA	-0.710256809	2.555553045	-4.484185287	0.000278244	0.528668728
SULF1	1.096191063	3.357431949	4.479494172	0.00028116	0.519387307
HAS2	-1.435044745	5.476003776	-4.455146309	0.000296801	0.471187882
TMEM123	0.622984972	9.273380927	4.452490578	0.000298559	0.46592786
EMILIN3	-0.716321298	3.691391593	-4.448947117	0.000300922	0.458908762
RGS3	-0.862447377	5.780314293	-4.433957873	0.000311127	0.429206998
PXDN	1.143151734	7.285699592	4.431008541	0.000313176	0.423360863
LAMA5	0.638495968	3.037974256	4.429122637	0.000314493	0.419622317
DMXL2	1.210445757	6.394658425	4.381727324	0.000349503	0.325585864
EDEM3	0.695535825	7.333429143	4.368653221	0.000359837	0.299618915
PRSS16	-0.720213168	2.612179112	-4.367006077	0.000361161	0.296346669
CLIP4	0.609162154	6.330061417	4.359944712	0.000366891	0.282316443
FOXQ1	-1.382805735	3.795466383	-4.356488882	0.000369729	0.275448888
LGALSL	-0.830803009	8.646890843	-4.355952496	0.000370171	0.274382893
SMKR1	-0.707675957	2.931565755	-4.338171581	0.000385144	0.239035461
CRYL1	-0.943154181	4.342029627	-4.331641272	0.000390796	0.226048654
GSTO2	-1.275553892	5.069111616	-4.32599555	0.000395749	0.21481893
OARD1	-0.61029659	7.488903295	-4.308913292	0.000411126	0.180829539
LDLR	-1.601830469	6.498533763	-4.308726774	0.000411297	0.18045832
FOXN3-AS1	-0.620650533	3.604855852	-4.306455901	0.000413387	0.175938536
PMVK	-0.751117013	4.75560239	-4.28295039	0.00043566	0.12913751
MMD	0.986515093	6.773428182	4.282127129	0.000436462	0.127497781
KANK2	0.787309607	6.435535046	4.279688375	0.000438845	0.122640174
LINC00467	-1.123047279	3.987824244	-4.268638975	0.00044981	0.100627479
SOWAHC	-0.733746155	6.721463715	-4.263125213	0.000455384	0.089640465
PALLD	-1.196309679	8.968735741	-4.22952349	0.000490895	0.022649854
PROK2	-0.831881703	2.931377475	-4.208907667	0.00051405	-0.018478814
TALDO1	0.727592381	11.56850607	4.208860129	0.000514104	-0.018573675
MB21D2	-0.664049048	5.443786023	-4.191049183	0.000534996	-0.054122524
QDPR	-1.111407796	5.629826859	-4.132101382	0.000610431	-0.17187214
SERPINA1	-1.037830855	10.54819322	-4.12069571	0.000626219	-0.194670704
HHLA3	-0.805205187	3.281369075	-4.115504119	0.000633541	-0.205049586
TOX	-0.619500885	3.033791587	-4.106342543	0.000646672	-0.223367397
SCGB1D2	-2.249564886	4.040611384	-4.101678474	0.000653461	-0.232693887
PQLC3	-1.120211688	7.284127016	-4.095615855	0.000662394	-0.244818037
HIPK2	0.940516225	5.904706979	4.083653785	0.000680381	-0.268743412
HOXA7	0.751639757	5.669707464	4.078520733	0.000688249	-0.279011376
ZFPM2	-1.118446022	2.833526222	-4.071296002	0.000699478	-0.29346476
CCND1	1.484930221	5.383347417	4.068437932	0.000703971	-0.299182864
MT1F	-1.389473761	9.445713973	-4.065228443	0.000709051	-0.305604316
PXDC1	-0.661975445	9.313190931	-4.063751905	0.0007114	-0.308558626
APOL3	1.178278516	3.981459552	4.056936279	0.000722346	-0.322196339
NRXN3	-0.668143808	2.375468624	-4.039631881	0.000750904	-0.356826936
PSMB10	0.643442806	4.289046688	4.037393705	0.000754679	-0.361306645
HEXA	-0.762568223	8.093617119	-4.030524564	0.000766386	-0.375055965
BCKDHB	-0.657115631	3.814088624	-4.021707161	0.000781681	-0.392706501
NSUN7	-0.633217259	2.333650758	-4.018922376	0.000786575	-0.398281388
ELL2	0.703455087	7.88099808	3.987515641	0.000843942	-0.461165378
GSTA4	-0.836004034	8.400376547	-3.9778359	0.000862455	-0.480550082
PPIA	-1.074146726	6.903511656	-3.971539253	0.000874715	-0.493160548
IBSP	-2.414626547	5.037774163	-3.963653638	0.000890317	-0.508954072
LGALS8	0.72962475	5.460544746	3.959743456	0.000898156	-0.516785793

KIAA1804	-1.162453439	4.115684986	-3.939679259	0.000939482	-0.556975262
CFL2	-0.810621109	7.818416262	-3.939296668	0.000940288	-0.557741649
PER2	-0.655290065	7.10895946	-3.932625127	0.000954459	-0.571105962
LYVE1	-1.50362957	5.520369528	-3.926259744	0.000968179	-0.583857331
SLC40A1	1.240892822	9.247796652	3.91578337	0.000991192	-0.604844624
PRKCH	-0.817705032	4.5596521	-3.913998342	0.000995167	-0.608420633
TMTC1	1.184674002	4.797732684	3.892650237	0.001043964	-0.65118917
C1orf94	-0.710933189	3.647451483	-3.89043405	0.001049165	-0.655629138
PIR	0.902292136	7.878022385	3.888342103	0.001054098	-0.659820207
MPV17	-0.836499594	5.310620861	-3.887495319	0.001056101	-0.661516683
BEX1	-1.0963424	3.272515718	-3.885943691	0.001059782	-0.664625267
LOC100505501	-0.757881875	2.641498149	-3.879621543	0.001074913	-0.677291298
TRAM2	0.733661681	6.381830081	3.873947794	0.001088676	-0.688658312
PHF11	-0.705403662	9.59360558	-3.873598771	0.001089528	-0.689357558
MAP3K8	1.091357104	7.768777897	3.862666941	0.001116566	-0.711258728
KLHDC9	-0.611866939	3.028644284	-3.858276357	0.001127613	-0.720054889
CADPS2	0.948045077	3.873103292	3.847888493	0.001154187	-0.740865829
MAN2B2	-0.759883845	7.978233739	-3.838498489	0.001178746	-0.759677226
ASB13	0.604637553	4.546233154	3.828960709	0.001204226	-0.7787841
INPP1	-0.60237031	8.680361305	-3.824892103	0.001215262	-0.78693446
ACTC1	-1.459980616	5.191829927	-3.81170525	0.001251729	-0.813349763
SLC44A3	-0.696341752	2.493332347	-3.805280062	0.001269891	-0.826219759
MAGI2	-0.671960367	4.250458518	-3.804763871	0.001271362	-0.827253697
CHMP4C	-1.713229267	6.48442426	-3.800907196	0.001282403	-0.834978572
ZEB2	1.650922664	3.328605033	3.794310981	0.001301508	-0.84819028
CPAMD8	-1.05330911	4.086387208	-3.792132119	0.001307881	-0.852554246
FBLN7	-0.946794383	4.211085262	-3.790923783	0.001311429	-0.854974351
INPP4B	1.065970152	5.017798055	3.775771937	0.001356739	-0.885319254
PRKCZ	-0.840429017	8.846121142	-3.771964463	0.001368369	-0.892943984
SCRN1	-1.123175195	5.226131399	-3.754975126	0.001421483	-0.926963131
ARMC9	0.733791378	4.615177651	3.750573483	0.001435576	-0.935775991
ZFP36L1	1.528745549	6.237822506	3.740984238	0.001466763	-0.954973924
NUDT11	-1.007698068	4.979426127	-3.724977709	0.001520331	-0.987014763
ARHGAP5-AS1	-0.997366442	5.476551818	-3.714774692	0.00155549	-1.007435182
GMPR	-0.915642413	3.742683462	-3.713764248	0.001559015	-1.009457344
IDI1	-0.796638244	10.02945216	-3.702899803	0.00159743	-1.031198155
ACAN	-0.952948861	11.95187531	-3.691728793	0.00163791	-1.053548855
USE1	-0.663816157	7.337341261	-3.689707157	0.001645345	-1.057593293
SERPINA5	-1.088421812	9.500406385	-3.688916927	0.00164826	-1.059174173
SDR16C5	-1.661894689	2.958608809	-3.682555512	0.001671914	-1.071899665
FAT4	1.446208783	5.739799555	3.676853567	0.001693403	-1.083304819
PLP2	-0.642081735	9.360060615	-3.659516243	0.001760444	-1.117976461
C4orf32	-1.15013865	5.164801307	-3.658906589	0.001762849	-1.119195467
C2orf42	-0.636351714	5.395077878	-3.648546585	0.001804219	-1.13990823
PTGDS	1.222862019	3.660024227	3.64613461	0.001813988	-1.144729905
WSCD2	-0.79566772	3.251190349	-3.62396658	0.001906272	-1.18903406
RAB3B	-0.846355282	2.217928685	-3.6211663	0.001918257	-1.194629122
PRPH2	-0.690719331	3.209676541	-3.615838784	0.001941264	-1.205272743
SMYD3	-1.029804487	4.146429416	-3.61378962	0.001950186	-1.209366344
AKR1C3	2.304257176	5.640682569	3.600760863	0.002007873	-1.235389322
C9orf69	0.663879311	5.301337275	3.596857092	0.002025486	-1.243184966
CXXC5	0.643056312	8.637667515	3.572005	0.002141258	-1.292795705
RBM20	1.091198716	3.354748787	3.568235649	0.002159381	-1.300317455

GPRC5A	-0.922428662	10.01075098	-3.568114097	0.002159968	-1.300559998
CSRNP1	-0.730468473	7.337897945	-3.565950365	0.002170443	-1.304877374
CDCA7L	-0.904035756	6.13026015	-3.565412504	0.002173054	-1.305950547
SMYD2	-0.618818952	6.967317007	-3.563145774	0.002184095	-1.3104731
DPAGT1	0.990600266	4.198789278	3.562487407	0.002187312	-1.311786613
STK38L	0.665955596	8.432575353	3.559835376	0.002200319	-1.317077462
S100B	-0.707299106	8.360231181	-3.555787707	0.002220319	-1.325151882
SCGB2A1	-0.630457334	2.026928952	-3.545114819	0.002273922	-1.346438073
TAF13	1.061927181	6.026862185	3.539689039	0.002301662	-1.357256824
ELMO1	0.877572592	3.098668931	3.534546663	0.002328262	-1.367508873
ZBED5-AS1	-0.716471577	3.071604035	-3.529539594	0.002354455	-1.377489645
HOXB7	0.675396675	5.649150745	3.522812448	0.002390106	-1.390896693
TRAM2-AS1	-0.63634862	5.532961887	-3.513896689	0.00243818	-1.408661239
PTPRM	0.659943795	8.49253701	3.510837678	0.002454894	-1.414755117
CENPM	-1.140325337	3.485422656	-3.506378197	0.002479463	-1.423637793
ALKBH3	-0.625434855	7.017850927	-3.506068197	0.00248118	-1.424255223
FGFBP1	-0.662230533	2.664055376	-3.497584481	0.002528627	-1.441149836
KCTD4	-0.946390999	2.752047063	-3.485925349	0.002595299	-1.464360096
SLC16A6	1.239865427	2.633485903	3.484596195	0.00260301	-1.467005498
TM4SF1	-0.822867833	11.2660856	-3.482720923	0.002613926	-1.470737621
CBX6	0.62681379	7.031852205	3.476651785	0.002649569	-1.482814576
BID	0.870837575	2.62537771	3.467606323	0.002703584	-1.50080921
TRIM29	-1.333900743	6.463062935	-3.462518125	0.002734445	-1.510928815
CRIP2	-0.784597802	4.88003918	-3.458920389	0.002756476	-1.518082972
GLRX	0.971094881	7.603206253	3.453810979	0.002788065	-1.528241444
DCXR	-1.2723576	6.037859998	-3.451813197	0.002800514	-1.532212875
THEMIS2	0.916298097	2.625909443	3.447554733	0.002827232	-1.540677338
SHISA2	-1.485773996	5.451235636	-3.445357866	0.002841114	-1.545043458
EFNB2	-0.987226688	3.452939343	-3.444516024	0.002846451	-1.546716459
TTLL7	-0.627080112	3.753887774	-3.443204116	0.002854789	-1.54932352
LAG3	-1.033518393	4.427973857	-3.442594496	0.002858671	-1.550534929
FLJ20021	-0.768795905	6.819656742	-3.428221822	0.002951731	-1.579087092
SUMF1	-0.852445062	7.095766298	-3.426149912	0.002965391	-1.583201693
PEX13	0.78040727	6.278725927	3.416075057	0.003032705	-1.603204281
C4orf48	0.872314356	4.562804121	3.412960746	0.003053817	-1.609385713
ABLIM1	-1.378942766	4.873276625	-3.406987185	0.003094717	-1.621240021
MAP4K4	0.641784236	6.304198949	3.406972239	0.00309482	-1.621269677
NAPRT	0.697342702	3.29936969	3.399335302	0.003147899	-1.636420421
MTMR11	-0.649557195	3.777747502	-3.39200567	0.003199686	-1.650956721
ZNF420	0.789362508	4.883756725	3.388961584	0.00322144	-1.656992425
UBLCP1	0.679289747	7.053941648	3.38771661	0.003230379	-1.659460676
SLC25A45	-0.704061215	3.60679391	-3.384742137	0.003251834	-1.66535722
SLITRK4	1.151402046	6.209819448	3.380983496	0.003279147	-1.672807142
JPH1	-0.763033078	4.113861487	-3.379275435	0.003291633	-1.676192226
SPAG4	-0.827553261	3.891579485	-3.374944039	0.003323508	-1.684775113
NQO1	1.881865811	8.195438003	3.37463865	0.003325767	-1.685380194
ZNF365	-0.600825651	3.349608683	-3.371230969	0.003351074	-1.692131376
PLCG2	-0.964075341	4.074544129	-3.360536798	0.003431733	-1.713311236
MAOB	-1.021982737	6.506881474	-3.359282739	0.003441316	-1.715794194
SERPINI1	-0.977408927	8.619239878	-3.358146615	0.00345002	-1.718043519
NUDT12	0.87033414	4.959068349	3.347910281	0.00352943	-1.738304025
BTN3A2	0.623124059	3.65656398	3.338681583	0.003602562	-1.756561324
UACA	0.717914029	4.584353359	3.335133179	0.003631077	-1.763578931

LOC100129550	-0.789619326	6.408226406	-3.330665138	0.003667297	-1.772413476
COL9A3	-0.944523369	10.64463918	-3.329720838	0.003674998	-1.774280356
DACT1	-0.661516584	3.061756601	-3.324684661	0.003716335	-1.784235332
AHCY	-0.778504421	6.468162552	-3.323367278	0.003727224	-1.786838961
ATP6V1E2	-0.632571523	3.302376026	-3.319824477	0.003756661	-1.793839937
LNP1	-0.923126589	6.107077489	-3.319764096	0.003757165	-1.793959246
EBF1	1.498717114	4.643660186	3.319596565	0.003758563	-1.794290272
CKB	-1.252384516	7.147777472	-3.31686431	0.003781433	-1.79968856
ADIRF-AS1	-0.960721619	3.222144899	-3.313359033	0.003810974	-1.806613004
MX1	-0.603691626	2.717125987	-3.309664613	0.003842355	-1.813909681
MAP3K7CL	-0.738957457	3.930045471	-3.299235849	0.003932314	-1.834499162
TXLNB	-0.605436243	2.577166172	-3.28918271	0.004020991	-1.854335837
GDF15	1.548320939	8.476730381	3.285824864	0.004051045	-1.860958988
ABCC1	0.603936018	8.522166932	3.284709721	0.004061075	-1.863158262
PHLPP2	-0.621141525	5.613753904	-3.275774282	0.004142326	-1.880775588
SCAMP1-AS1	-0.658298578	4.226448703	-3.267161859	0.00422215	-1.897747424
SOAT1	0.89939503	6.732318871	3.260746212	0.004282592	-1.910384655
CCPG1	0.744404693	9.291911507	3.259671702	0.004292797	-1.912500702
CCNB1IP1	-0.65121661	9.983941347	-3.259435731	0.004295042	-1.912965386
ZNF124	-0.771012784	5.986489127	-3.251441037	0.004371767	-1.92870493
ZBTB41	0.662206266	7.551148689	3.246919102	0.004415757	-1.937604105
KYNU	0.740482777	2.591201334	3.241943314	0.004464665	-1.947393612
RPTN	-0.69450586	2.100161918	-3.239101267	0.004492837	-1.952983786
IP6K3	0.709917148	3.316331541	3.22720873	0.004612626	-1.976365059
CHST1	-0.78182034	8.511758003	-3.219068853	0.004696415	-1.992358249
SYBU	-0.75466722	7.878398385	-3.21301901	0.004759653	-2.004239505
POPDC3	0.922691257	8.35922584	3.211165329	0.004779195	-2.007879003
SLC2A12	0.977291047	4.182860088	3.205907606	0.004835054	-2.018199541
UST	0.724796404	4.27248804	3.204497421	0.004850144	-2.020967023
RNF19B	-0.72745751	8.595037322	-3.198361397	0.004916344	-2.033005922
GGTA1P	-0.949566838	4.597427089	-3.181304248	0.005105047	-2.06644585
SPIDR	0.742056723	4.826679924	3.169700217	0.005237452	-2.089172613
LINC00545	-0.91471143	3.455545184	-3.169154718	0.005243758	-2.090240529
MMP1	2.082072425	3.539864452	3.15596188	0.005398552	-2.116055308
C5	-0.715278617	4.46860192	-3.154083143	0.005420956	-2.119729488
SRPR	0.637846152	7.522281695	3.14461837	0.00553521	-2.138231733
BFSP1	-0.886293217	3.764645996	-3.143548594	0.005548271	-2.140322178
TMEM17	-0.722076361	4.363559945	-3.142039861	0.005566742	-2.143270106
THNSL2	-0.751542761	5.114755765	-3.138110204	0.005615135	-2.150946743
SLC43A3	0.601850157	4.601715048	3.13643798	0.005635852	-2.154212772
PRTFDC1	-0.915099735	4.94748922	-3.132414432	0.005686006	-2.162069501
LOC100132891	0.803824275	2.374992791	3.12733081	0.005749997	-2.171992803
PPP2R2B	-1.00327367	4.611766247	-3.126496962	0.00576056	-2.173620122
SPR	0.604355346	4.713239903	3.126295654	0.005763113	-2.174012974
SYTL2	-0.727154728	3.213271417	-3.12509835	0.00577832	-2.176349387
TDRD6	0.643461602	3.360950484	3.122260552	0.005814519	-2.181886192
C1GALT1C1	0.837475995	6.242703535	3.121214574	0.005827918	-2.183926689
ALAS1	0.696407104	7.445336037	3.117994802	0.00586935	-2.190206793
VWDE	-0.757385573	2.15234636	-3.10983288	0.005975671	-2.206119431
LOC440028	-1.161364513	3.323256581	-3.105137547	0.006037683	-2.215268933
MAMLD1	-0.729747245	2.673678469	-3.102225185	0.006076461	-2.220942364
HSD11B1	1.48908376	3.700757865	3.101023057	0.006092538	-2.223283789
DSE	0.79732973	10.2416086	3.100694533	0.006096939	-2.223923627

RPN1	0.619627856	10.26000839	3.084347433	0.006319878	-2.255740134
LOC100505841	-0.95550577	3.470656207	-3.08341812	0.006332788	-2.257547597
NEDD4	0.723417456	5.658467138	3.078455932	0.006402156	-2.267196438
PAAF1	-0.610626022	6.449310884	-3.076254746	0.006433164	-2.27147532
SELENBP1	-1.029867981	3.920620668	-3.075788007	0.006439757	-2.272382513
CHCHD1	0.624832811	8.059609859	3.075677818	0.006441315	-2.27259668
VRK1	-0.639764987	6.689823324	-3.07012929	0.006520223	-2.283378463
FAM189A2	-0.935628222	3.459941457	-3.06690153	0.006566558	-2.289648275
CAB39L	-0.818915686	6.403934471	-3.065822669	0.006582117	-2.291743545
FNDC4	-0.794548817	4.404680721	-3.062103661	0.006636025	-2.298964816
IRX3	1.533096142	5.343164189	3.05641481	0.00671932	-2.310006555
MYOT	-1.091409078	4.705620441	-3.053817273	0.006757689	-2.315046443
ZNF135	0.805848404	3.645297385	3.053454337	0.006763067	-2.315750541
THAP2	0.982142682	4.29277674	3.053326046	0.006764969	-2.315999422
COL11A2	-1.323588541	7.128506568	-3.047043329	0.006858756	-2.32818432
YAE1D1	-0.664354948	6.792051668	-3.045935204	0.006875428	-2.330332764
EYA4	0.631531725	2.800199368	3.045046972	0.006888821	-2.332054727
ATF3	-0.745762403	4.800462716	-3.042746894	0.006923618	-2.336513134
C7orf55	-0.747318184	4.633592389	-3.04094845	0.006950946	-2.33999856
IRX5	1.229607855	6.271473006	3.032728725	0.007077185	-2.355921558
AGT	-1.155619629	7.089124996	-3.032165488	0.007085916	-2.357012219
TBC1D4	-0.682413288	4.786653298	-3.028268643	0.007146612	-2.364556631
TSPAN1	-0.765351489	3.553136407	-3.026388546	0.007176076	-2.368195614
CLIC2	0.829862911	1.985534571	3.022677949	0.007234574	-2.375375785
SERPINB2	-0.714041626	2.475431272	-3.018993727	0.007293114	-2.382502536
PTPLAD2	-0.8587409	3.614889153	-3.013322562	0.007384125	-2.393468161
TLR1	0.727430762	3.674691409	3.001765772	0.007573014	-2.415796399
PELI1	-0.660040213	10.73661497	-2.99751056	0.007643737	-2.424011622
CD1D	1.52116075	4.544262427	2.99728311	0.007647535	-2.424450649
SAMD9	0.704304979	2.789411926	2.996204186	0.007665577	-2.426533083
IGFBP1	2.647170398	4.272732721	2.99379623	0.007705993	-2.431179921
MGAT4B	0.621547468	7.627927321	2.977635219	0.007982641	-2.462339691
LIFR	0.841809182	6.188001502	2.972655536	0.008069807	-2.471931175
TNFAIP6	1.376896967	9.096467527	2.972381007	0.00807464	-2.472459816
SEMA3E	1.104952489	7.324327478	2.964811525	0.008208985	-2.487030256
LY75	-1.165628423	3.35271454	-2.961834433	0.008262415	-2.492757865
IRF1	0.720661263	4.972827435	2.949422276	0.008488824	-2.51661927
TMEM71	-1.082129159	5.147770292	-2.949140819	0.008494027	-2.517160006
AGA	-0.838661135	5.717320682	-2.942258966	0.00862221	-2.530376625
CDS1	-0.746298557	4.000046707	-2.938237117	0.008697986	-2.538096311
COL8A2	0.709129262	5.306878631	2.934148152	0.008775686	-2.545941564
CDNF	-0.604508158	3.032433167	-2.933650375	0.008785191	-2.546896393
SPG20	0.666221242	8.362890468	2.930056907	0.008854102	-2.55378788
MCM2	-0.728844283	4.539784535	-2.928140928	0.008891057	-2.557461259
LIAS	-0.756713783	5.583693431	-2.928056896	0.008892681	-2.557622351
COL6A2	-1.093370373	7.042299064	-2.924176918	0.008967989	-2.565058868
RUNX1-IT1	0.837427505	3.303534135	2.915304752	0.009142514	-2.58205224
ARG2	-0.916475243	6.038988003	-2.914193176	0.00916461	-2.584180185
ADTRP	-1.166435665	5.793621094	-2.91141303	0.009220099	-2.589501256
LINC00936	-1.036675771	5.071345185	-2.910917197	0.00923003	-2.590450091
RORC	-0.633759942	2.82976178	-2.910821651	0.009231945	-2.590632925
ZEB1-AS1	-0.730968955	6.364071211	-2.903920895	0.009371257	-2.603832991
TUBA4A	0.681927391	7.412539034	2.903884065	0.009372006	-2.603903416

SFRP2	0.790524941	2.503760536	2.90204535	0.00940947	-2.607418924
S100A3	-1.021420408	3.715356777	-2.896441566	0.009524541	-2.618128704
CCDC91	0.639555781	8.277661094	2.894420919	0.009566366	-2.621988903
MYH10	-0.661169034	6.625798327	-2.893250532	0.009590673	-2.624224396
MFAP4	1.043602922	3.041192889	2.892465766	0.009607005	-2.625723173
ARHGDIB	0.625916397	3.427256803	2.890710138	0.009643638	-2.629075679
CLEC3A	-1.708521224	6.906481576	-2.883709786	0.009791052	-2.642436947
RBPMS	0.622012453	3.568750982	2.881656235	0.009834707	-2.646354511
FRMD4B	0.679216271	8.484521586	2.875288263	0.009971275	-2.658497014
CASP4	0.757790126	4.795051744	2.873471841	0.010010564	-2.661958997
GREM1	1.224310377	8.941100263	2.872238257	0.010037331	-2.664309726
RAB31	0.85367014	8.094756785	2.869359017	0.010100076	-2.669795162
MRAP2	-0.778228497	3.258449388	-2.864941164	0.010197086	-2.678208442
SLC6A12	-0.906169909	3.848409953	-2.860583529	0.010293653	-2.686502903
CLCF1	-0.880415304	6.488410856	-2.859394373	0.010320157	-2.688765665
DKK1	-1.142102848	3.852638831	-2.856847308	0.01037715	-2.693611257
PTGES	0.842145296	4.080253811	2.854941309	0.010419996	-2.697236343
FAM46B	-0.756798409	3.530223533	-2.851588483	0.010495779	-2.703611268
GALNT7	0.666066728	7.82476127	2.847863434	0.010580599	-2.710691019
LRP8	0.623427123	4.392763687	2.847070493	0.010598739	-2.712197671
DEFB1	1.395979913	9.277511895	2.842894717	0.010694764	-2.720129681
ST6GALNAC2	1.028035475	4.805333874	2.838262452	0.010802266	-2.728924265
LOC100289098	-0.959489017	4.626879836	-2.836067817	0.010853559	-2.733089214
GPR126	-1.15647527	3.926504346	-2.832348233	0.01094103	-2.740145719
TMEM56	-0.676537449	2.631560528	-2.822920935	0.011165771	-2.758016454
LOC93622	-0.688138138	7.035725417	-2.822212976	0.011182826	-2.759357671
FGF1	-0.797535354	3.022585756	-2.81991079	0.011238461	-2.763718337
MGAT1	0.761081167	7.684046923	2.805791162	0.011585539	-2.790436244
HIST1H4H	-1.339850798	5.023922142	-2.798846342	0.011760006	-2.803560617
BAG2	0.673915949	6.7212887	2.797755073	0.01178765	-2.805621879
CNIH3	1.001700646	4.990434765	2.796071729	0.011830413	-2.808800941
LPXN	-0.760503889	4.954604036	-2.795969848	0.011833006	-2.808993325
DET1	-0.654607415	5.195660895	-2.792638407	0.011918094	-2.815282835
LECT1	-1.827436654	9.201031944	-2.792257636	0.011927857	-2.816001535
TNFRSF12A	-0.762858872	8.72459529	-2.791870729	0.011937785	-2.816731782
PDLIM1	-1.278680771	4.631781509	-2.791788034	0.011939908	-2.816887855
CRISPLD2	-1.233301817	7.976496818	-2.788100946	0.012034931	-2.823844994
GLA	0.888278748	8.390742012	2.787788294	0.012043022	-2.824434787
LOC100130992	-0.73160574	3.87624378	-2.781995438	0.012193878	-2.835358317
GSR	0.75030522	5.862921101	2.776211517	0.012346309	-2.846256977
VPS13A	0.697001146	4.475970019	2.774523765	0.012391132	-2.849435695
FOXP1-IT1	0.653582829	5.69320112	2.768080649	0.012563685	-2.86156434
PTMS	-0.637334492	4.814432751	-2.760052727	0.012781904	-2.876662097
TPPP3	-0.910285175	3.714823913	-2.757375717	0.012855475	-2.881693117
MATN1-AS1	-0.732229137	4.75161769	-2.755451483	0.012908609	-2.885308321
COL9A2	-1.157207849	6.591245491	-2.754975567	0.012921782	-2.88620232
ZNF474	-0.797052649	3.447326167	-2.747630412	0.013126743	-2.89999292
SH3TC2	-0.637961388	2.999825594	-2.739822842	0.013348016	-2.914636955
AGAP1	-0.67281462	5.053700277	-2.736997595	0.013428962	-2.919932273
ITGB8	0.68601229	4.266559282	2.736041703	0.013456456	-2.921723431
IER5L	0.701921879	6.01726206	2.734038281	0.013514253	-2.92547671
CDKN3	-0.741015055	4.397913605	-2.733856744	0.013519502	-2.925816756
MT4	-0.66036999	8.811256773	-2.731785711	0.013579521	-2.929695536

IGFBP5	0.620333841	3.273211817	2.73073199	0.013610156	-2.931668602
ABHD4	0.657860778	4.271914632	2.722814144	0.013842476	-2.946485518
CAMTA2	-0.663007565	4.970172326	-2.72176712	0.01387348	-2.948443647
HOPX	-1.531716454	2.989948635	-2.717554233	0.013998899	-2.956319673
TIAM1	-0.811768901	4.378384763	-2.715781346	0.014052002	-2.959632733
TTC13	0.689068489	4.97037834	2.712801569	0.014141688	-2.965199319
CHST10	-1.103610816	6.571357852	-2.712614706	0.01414733	-2.965548325
DUSP10	0.702061137	5.413983771	2.711262329	0.01418823	-2.968073905
SOX9-AS1	-0.817548137	9.254661568	-2.710553315	0.014209718	-2.969397807
RAB11FIP1	-0.757419279	3.860422617	-2.705303445	0.014369794	-2.979196518
ALDH3A2	1.041097534	7.428783591	2.702195838	0.014465358	-2.984993371
HIST1H2AE	-1.478890701	3.691297953	-2.696881411	0.014630194	-2.994900889
STK19	-0.718730111	5.338177367	-2.69506768	0.014686859	-2.998280466
SPATA7	-0.832271709	5.071590014	-2.694974854	0.014689765	-2.998453408
IFNAR1	0.608943478	3.74226408	2.692883343	0.014755381	-3.002349447
RAPGEF5	-1.172797805	6.358016776	-2.692168369	0.014777875	-3.003681025
ZBED9	-0.940872631	3.434692516	-2.690933511	0.014816803	-3.005980523
TTLL4	0.60291541	4.48470535	2.681583629	0.015114743	-3.023378267
FLNC	0.861158166	3.947421776	2.676341244	0.015284285	-3.033122749
CYFIP2	0.626737244	3.280275814	2.668101107	0.015554444	-3.04842439
NT5DC3	-0.81839505	4.700437865	-2.66677797	0.015598246	-3.050879688
NOVA1	0.762176079	8.792959438	2.666290406	0.015614416	-3.051784322
PDGFRB	0.974676826	3.977907331	2.655166506	0.015987707	-3.072406091
PCK2	-0.609387889	7.549826921	-2.651311584	0.016119039	-3.079544483
CHUK	0.68211056	6.495209977	2.642568055	0.016420735	-3.095720087
LDOC1	-0.608976703	6.829587615	-2.639538766	0.016526507	-3.101319311
ADNP2	-0.824169954	6.998263308	-2.636929762	0.016618123	-3.10613963
PPP1R15A	-0.809298205	8.045945148	-2.630838171	0.016833916	-3.117386792
MVP	0.630117677	5.092293079	2.630735897	0.016837561	-3.117575535
IFIT3	1.224422011	6.912638555	2.628401776	0.016920967	-3.121882267
DNAJC12	0.874576962	5.431019937	2.619528754	0.017241626	-3.138239878
LPL	-0.978083949	6.412331484	-2.615809832	0.017377731	-3.145089081
DYNLRB2	-0.636937905	3.896131692	-2.610126366	0.017587707	-3.155548712
SPTSSB	-1.26286057	4.778213976	-2.60676585	0.017712991	-3.161728872
CAPG	-0.656010351	6.21717943	-2.606459078	0.01772447	-3.162292878
ZNF367	-0.722179422	5.20621653	-2.602565552	0.017870775	-3.169448814
NR4A3	-0.782194416	4.142330089	-2.591604959	0.018288809	-3.189569503
IFNAR2	0.64268632	6.934409028	2.58672675	0.018477827	-3.198513181
IL17RD	-0.754909315	3.431781744	-2.583349359	0.018609776	-3.204701128
SLC25A33	-0.630471325	8.396677557	-2.583064865	0.018620931	-3.205222214
TOLLIP-AS1	-0.641235097	3.214765146	-2.578804769	0.018788734	-3.213022213
ZNF204P	-0.72968916	3.338433344	-2.576206277	0.018891788	-3.21777724
MAN1A1	1.277828753	5.022894089	2.573818936	0.018986941	-3.22214409
MYD88	0.603613138	5.123652749	2.568024843	0.019219766	-3.232735324
ADH1B	0.925103006	2.05486445	2.565911497	0.019305357	-3.236595859
GPR137B	0.63935783	8.334779742	2.561501685	0.019485118	-3.244647072
SSPN	-0.605219542	6.406272727	-2.557689097	0.01964181	-3.251603128
CD80	0.848999477	3.40375302	2.557186799	0.019662542	-3.252519238
MRPL34	0.645785075	6.345029036	2.555990337	0.019712009	-3.254701079
NUDT9P1	-0.697850007	3.454489114	-2.555202402	0.01974465	-3.2561377
ACTR1A	0.625753741	5.513426905	2.547613689	0.020061646	-3.269964226
NIPSNAP3B	-0.833296142	4.142700462	-2.546269061	0.020118313	-3.272412266
MT1M	-1.212321266	8.214867469	-2.545514517	0.020150178	-3.27378575

PNPLA4	-0.692569932	4.664336686	-2.545043908	0.020170077	-3.274642302
ZMYND12	-0.770229474	3.059192909	-2.542734069	0.020268011	-3.278845428
TCP11L2	0.611949588	3.349496897	2.542072578	0.020296141	-3.280048813
CXCL8	1.883541952	5.631364715	2.541545377	0.020318586	-3.2810078
ILF3-AS1	-0.770241378	8.056315848	-2.53087641	0.020777873	-3.300396186
S100A2	-1.563529121	10.67226982	-2.530461753	0.02079592	-3.301149009
TLR2	1.049678477	3.800613384	2.525448594	0.021015278	-3.310246281
MSMO1	-0.765032231	11.11390671	-2.518619869	0.021317592	-3.322625438
SOCS2	-0.994970431	4.876902117	-2.517487809	0.021368104	-3.324676214
GBP1	1.03638574	4.763902126	2.507994338	0.021796161	-3.341857932
CDH11	1.113361817	4.572775378	2.505315541	0.0219184	-3.346700912
SALL3	-0.747463015	2.415252279	-2.503083518	0.022020746	-3.350734407
LIMA1	0.708474823	6.719701918	2.496746901	0.022313757	-3.362176542
ST8SIA1	-0.691859396	2.327267794	-2.493805046	0.022451036	-3.36748427
EBP	-0.639630132	4.131719168	-2.490691711	0.022597181	-3.37309831
GDAP1	-0.660893631	3.422345212	-2.490210769	0.022619837	-3.373965272
ITGBL1	1.037623677	6.413080305	2.488669331	0.022692595	-3.376743415
SOX4	1.31424054	6.203557844	2.481093618	0.023053386	-3.390385836
ARSI	0.72296102	3.584384927	2.476658813	0.023267089	-3.398363299
ST6GALNAC4	0.646916844	4.957470309	2.469231318	0.023629178	-3.411709495
LXN	-0.677328198	5.265312325	-2.466651365	0.023756184	-3.416341018
PTHLH	0.916276265	3.364521179	2.459032176	0.024135015	-3.430005942
RIN2	1.33630252	8.525354169	2.458395056	0.024166948	-3.431147725
PRKD1	-0.750476475	7.712391413	-2.457069709	0.024233504	-3.433522442
GGH	-0.686338886	3.664010962	-2.450632941	0.024559197	-3.445047197
CFB	-0.949588012	8.19427599	-2.4499915	0.024591878	-3.446194901
ADRB2	-0.649528608	6.511622085	-2.436002065	0.025314837	-3.471190676
PWAR6	0.730460559	4.301220665	2.434041275	0.025417746	-3.474688776
T	-1.110196968	3.303458428	-2.430401385	0.02560982	-3.481178915
AKR1B1	0.703648946	10.15379566	2.429687732	0.025647638	-3.482450863
PPP1R3B	-0.682472263	3.762350094	-2.429354681	0.025665305	-3.483044402
TEX9	-0.697446364	5.191342719	-2.417024226	0.026327458	-3.504991669
RARB	0.677047506	3.496219123	2.414351397	0.026473084	-3.509742093
MFSD6	0.659882802	4.241377684	2.410476765	0.026685526	-3.516624031
SETD6	0.836178424	5.599965637	2.406662625	0.026896207	-3.523393356
POGLUT1	0.719927233	5.343333667	2.405396765	0.026966473	-3.525638864
HCG4	-1.067704671	5.88368378	-2.402896491	0.027105762	-3.53007243
TMEM158	-0.665782824	3.124162256	-2.402198838	0.027144748	-3.531309135
GCLM	0.69453052	10.17731161	2.401700088	0.027172651	-3.532193145
TMPRSS4	-1.003004733	4.284404656	-2.399706998	0.027284423	-3.53572492
SERTAD1	-0.815909338	7.738225585	-2.398771231	0.027337049	-3.537382621
LPAR4	0.620010754	3.967442531	2.39394458	0.027610001	-3.545928015
C2orf82	-0.973139072	9.66575813	-2.393322722	0.027645353	-3.547028384
KCNN4	1.026644575	5.42941119	2.391070637	0.027773732	-3.55101226
ALCAM	1.001591531	6.761156895	2.388868401	0.027899809	-3.554906195
PEG10	-0.838044139	3.807839231	-2.386149351	0.028056209	-3.559711539
CCDC151	-0.658612039	5.363220352	-2.383782405	0.028193022	-3.563892446
SDC4	-0.656148987	10.95827336	-2.373080248	0.028819428	-3.582771114
WIF1	-1.715006608	8.34271622	-2.371269508	0.028926687	-3.585961149
KRT19	-1.69930153	3.266810402	-2.370812444	0.02895382	-3.586766184
DLGAP1-AS2	-0.851582677	4.553687296	-2.36587377	0.029248513	-3.595459875
COL4A1	1.354341619	4.236660092	2.365396101	0.029277163	-3.596300256
LINC00847	-0.667552481	4.994823898	-2.362565295	0.029447492	-3.601278877

MAP3K5	1.006143517	5.996563382	2.361202602	0.029529813	-3.603674433
SFT2D2	0.624485061	5.301756589	2.357331055	0.029764867	-3.61047671
GLDN	-0.856739093	7.252431799	-2.356805378	0.029796917	-3.611399894
HMGCR	-0.761316296	9.054546469	-2.354116888	0.029961332	-3.616119766
PLEKHG4	-0.774570837	3.836470354	-2.352727625	0.030046623	-3.618557684
NANOS1	1.252369519	5.373884289	2.348606294	0.030300975	-3.625785696
CALR	0.629276801	6.401017725	2.342970827	0.030652017	-3.635658956
CYP4X1	-0.601336866	3.298271676	-2.341104183	0.030769125	-3.63892667
RAP1A	1.098144622	10.0031597	2.329705914	0.031493287	-3.658851844
MRC2	-0.749423329	6.787448288	-2.328366741	0.0315794	-3.661189613
PRMT6	0.93043048	5.671687135	2.32712231	0.031659616	-3.663361383
CTSO	-0.786147848	5.246573993	-2.326255384	0.031715611	-3.664873988
ERICH1	-0.74165731	7.525949983	-2.321257891	0.032040192	-3.673587991
PSRC1	0.837189332	3.457604019	2.312473991	0.032618179	-3.688881128
MGLL	-0.644756712	7.493849041	-2.311313137	0.032695283	-3.690900008
TTC32	-0.67655601	7.32501152	-2.310406718	0.032755605	-3.69247603
LAYN	-0.682890901	6.670144699	-2.298037088	0.033589181	-3.713951805
CHAC2	1.230877027	6.83995027	2.293410694	0.033905966	-3.721968732
RAB38	-0.61373157	3.241618285	-2.29239111	0.033976151	-3.723734412
SCO2	0.682870414	5.525654479	2.288467245	0.034247516	-3.730525826
MICB	0.753092434	3.759730666	2.287347354	0.034325332	-3.732463022
ZNF329	0.675116368	3.53209718	2.286521293	0.034382835	-3.733891633
LY96	0.625991448	4.150975691	2.283962909	0.034561496	-3.738314464
GUSBP11	0.675040372	3.973502238	2.275935186	0.035127688	-3.752175693
PTGR2	0.788738507	4.833441385	2.274234932	0.035248701	-3.755108196
PCSK1	-0.705530066	4.422808089	-2.265714218	0.035860975	-3.769786906
WBP1L	0.601894932	5.518600641	2.258769625	0.036367242	-3.781728936
AKNAD1	-0.800868386	4.215921636	-2.257215462	0.036481441	-3.784398844
DUSP5	1.00414603	7.814128073	2.25704395	0.036494064	-3.784693427
NTN4	0.875262725	9.21080194	2.254644413	0.036671088	-3.78881353
HLF	-0.74724514	4.167907102	-2.25422416	0.036702173	-3.789534883
SPARCL1	2.001784532	3.691416945	2.236879955	0.03800648	-3.819243179
GADD45G	-0.716677607	5.489179051	-2.219486309	0.039357255	-3.848912312
RERG	1.024758116	3.742576386	2.219302008	0.039371802	-3.849226012
VEGFC	0.974780245	2.387305088	2.211770983	0.03997044	-3.862032522
SLC26A6	0.615416533	3.285801324	2.206941524	0.040358721	-3.870232538
MMP2	2.159729649	3.138074124	2.206114252	0.040425579	-3.871636195
WARS	0.692057236	8.328529825	2.205670961	0.040461446	-3.872388221
TGFB2	0.878193779	2.904148765	2.197041865	0.041165484	-3.887010658
ZNF33B	-0.631264428	3.555384751	-2.195530731	0.041289927	-3.889568113
JARID2	0.820190904	5.535795949	2.190445955	0.041711197	-3.898166508
PDGFA	0.615082998	3.910405822	2.19021362	0.041730539	-3.898559126
TSLP	-1.304916385	6.38596644	-2.190108097	0.041739327	-3.898737438
RFTN1	0.891740977	2.777841836	2.18968155	0.041774867	-3.899458171
CPVL	-1.497938496	3.348890963	-2.180411216	0.042554149	-3.915102992
BIRC3	0.930350197	6.23224317	2.170165861	0.043430864	-3.932350363
KLF3-AS1	-0.929494849	5.413994425	-2.160432341	0.044279057	-3.948694034
SESN3	0.967629831	4.396013691	2.159943993	0.044322009	-3.94951294
FAM110C	0.732406743	6.311995436	2.158318523	0.044465248	-3.952237923
MXRA8	0.81567749	6.061506528	2.157118748	0.044571246	-3.95424852
ITGA5	0.776464284	7.450091967	2.155896878	0.044679433	-3.956295496
TRIM22	0.927204938	2.81784448	2.144092507	0.045737033	-3.97603741
THBS1	0.891974197	7.715623398	2.143638137	0.045778195	-3.976796082

COL4A2	0.957525746	2.841571169	2.137911878	0.046299846	-3.986349534
ADAMTS6	0.618311361	2.292504944	2.136812694	0.046400598	-3.988181705
BANK1	-0.916547129	4.781557977	-2.133296104	0.046724273	-3.994039709
CYTL1	1.120473104	10.25093291	2.13212611	0.046832418	-3.995987489
NUDT7	-0.748413119	3.524870553	-2.131327552	0.046906361	-3.99731656
TXNIP	0.745181624	9.816409075	2.128116597	0.047204756	-4.002657802
FLJ38717	-0.683259755	7.548562844	-2.126552047	0.047350776	-4.005258674
CHURC1	-0.961747139	7.49569332	-2.114851153	0.04845593	-4.024675131
HMGCS1	-0.703017141	6.319842625	-2.109808726	0.048939383	-4.033023495
CA9	-0.969804714	5.221236282	-2.106843294	0.049225743	-4.037927756
STEAP1	0.719695812	6.108703305	2.100641368	0.049829568	-4.048171629
CA12	-0.921574562	5.523197057	-2.098824098	0.050007769	-4.051169937
CPM	0.748499621	5.769458789	2.092939585	0.050588779	-4.060868396
WFDC21P	0.615124748	4.514516434	2.09070113	0.050811398	-4.064553496
GATA3	0.675335671	2.465536105	2.085215736	0.051360692	-4.073574176
KCNK6	0.600072133	5.03123157	2.084105011	0.051472571	-4.075399064
SOD2	0.616225932	11.33518612	2.073101977	0.05259285	-4.093445814
ANPEP	0.815558458	2.978158167	2.072347823	0.052670437	-4.094680685
SLC30A1	-0.671869884	7.10994136	-2.07133415	0.052774887	-4.096340084
PPL	-0.679926214	3.665830867	-2.071024002	0.052806883	-4.096847706
NDNF	-0.645495629	3.99234836	-2.068763661	0.053040594	-4.100545862
PON3	-0.729296038	4.82169351	-2.067784496	0.053142126	-4.102147137
CMAHP	0.691458583	7.816022562	2.064136909	0.053521904	-4.108108261
SLC7A2	0.634810732	10.14951375	2.05593991	0.054384325	-4.121481517
MANF	0.649189878	10.03092987	2.050741699	0.054937726	-4.129945878
PRC1	-0.616572389	4.131022728	-2.049179677	0.055105009	-4.13248685
JUN	-0.657667437	10.02559697	-2.048595991	0.055167636	-4.133436048
INHBA	-0.750644755	10.33232053	-2.042429198	0.055833246	-4.143454663
C5orf34	0.716818878	4.355913493	2.040971601	0.055991625	-4.145820039
RP2	0.695483436	7.0857732	2.037381293	0.056383472	-4.151642029
ZNF165	-1.341815051	5.893896719	-2.032842509	0.056882372	-4.15899322
C12orf66	-0.629695888	3.817954447	-2.032249384	0.056947861	-4.159953137
SEMA3C	0.720913216	7.644521497	2.029389357	0.057264601	-4.164579453
NEFM	-1.383725998	3.786927988	-2.028140029	0.057403458	-4.166599103
CD151	0.66444258	5.474846302	2.021727909	0.058120919	-4.176953011
APCDD1L	0.934299364	3.362981658	2.019319545	0.058392472	-4.180836754
LINC01207	-0.638573114	2.86051803	-2.012856975	0.059126803	-4.19124442
CBLB	0.765218655	9.368922984	2.012000022	0.059224798	-4.192622977
TCIRG1	0.628075926	4.361403756	2.009385771	0.059524649	-4.196826236
FICD	0.886852444	6.706343039	2.0074717	0.059745053	-4.19990161
COL10A1	1.932913349	8.489563227	2.005019847	0.060028455	-4.203838431
CHADL	-0.630115409	6.112223472	-2.001893307	0.060391592	-4.20885429
LTBP1	0.926148712	6.023971462	1.999660378	0.060652146	-4.212433603
GULP1	0.677995909	9.38967272	1.997651498	0.060887417	-4.215651677
SQLE	-0.76041121	7.203009278	-1.989146761	0.061892552	-4.229253565
SBF2-AS1	-0.641143088	3.637086904	-1.98911611	0.061896202	-4.229302523
HYOU1	0.766832636	7.625758506	1.986432228	0.062216488	-4.233587465
DHCR7	-0.640851976	5.148917633	-1.985589057	0.062317416	-4.234932888
CHRDL2	-1.542476353	7.880595402	-1.983836154	0.062527708	-4.237728812
BEND6	0.649324673	2.843722531	1.978424196	0.063180985	-4.246351371
BMP6	-0.733402542	10.26337414	-1.9731159	0.063827679	-4.254794559
FDPS	-0.702142228	8.790030277	-1.971725429	0.063998051	-4.25700386
SLC47A1	0.65681352	3.638844012	1.971009745	0.064085902	-4.258140622

ULBP2	-0.692333623	7.24096976	-1.965540567	0.064760813	-4.266819138
AKR1C1	0.969042257	10.67837144	1.954348575	0.066161764	-4.284531583
FZD10	1.169980341	6.490733904	1.951739074	0.066492271	-4.288652249
HSF2BP	-0.728803275	3.750662299	-1.949324464	0.066799405	-4.292462076
SPAG5-AS1	-0.635082761	5.073647212	-1.945187399	0.067328574	-4.298982721
SGK223	-0.674644091	3.745275862	-1.943345973	0.067565309	-4.301882277
RCAN1	-0.956223093	5.508761752	-1.940373518	0.067949015	-4.306559117
NPR3	0.821358864	3.700003739	1.935061239	0.0686396	-4.314906123
C10orf10	1.12899078	6.63472512	1.932962715	0.06891412	-4.318199465
RDH10	0.8734151	6.195993085	1.931900637	0.069053429	-4.319865383
HTRA1	-0.673254778	11.23011457	-1.92957367	0.069359525	-4.323513304
CFD	0.830344012	6.068912969	1.924852754	0.069984239	-4.330905547
COL2A1	-0.696273174	11.17707598	-1.924268748	0.070061866	-4.33181921
GPR56	0.649150697	5.334007023	1.92084756	0.070518158	-4.33716802
ADAMTS5	1.28388547	4.023819124	1.920209546	0.070603544	-4.338164842
MOXD1	-0.799079215	5.076741852	-1.917688598	0.07094182	-4.342101454
RBPMS2	-0.636834953	6.727615442	-1.915364332	0.071254978	-4.345728002
IFITM2	1.01210015	7.461920203	1.909574635	0.072040392	-4.354749388
NAP1L5	-0.758565007	6.717886741	-1.907667563	0.072300776	-4.357717107
GPR133	-0.819014878	5.570151218	-1.905533062	0.072593203	-4.36103648
PCDHB16	0.779864329	6.366669226	1.901273678	0.073179874	-4.367653114
FXYD6	-0.679808751	8.494103155	-1.900594691	0.073273782	-4.368706986
CYR61	-0.625980472	9.005881409	-1.892636238	0.074382473	-4.381041355
LRP12	0.705354033	6.466673754	1.880940069	0.076038808	-4.399107648
PCOLCE	-0.789230958	4.756184776	-1.88032562	0.076126716	-4.400054732
DPT	-0.95804974	4.582316946	-1.876192565	0.076720366	-4.406419988
DSC2	-0.792575928	4.916929099	-1.874469482	0.076969065	-4.40907098
F13A1	0.674457757	10.74985972	1.864259779	0.078457321	-4.424746048
PTGER2	0.730492374	5.542352339	1.863445246	0.078577141	-4.42599419
TSIX	-1.013860489	2.891868057	-1.854388245	0.079920348	-4.439848403
HOTAIRM1	0.756048061	4.954982219	1.850499183	0.080503296	-4.445783713
SLC26A9	0.876068972	3.0927986	1.83684548	0.082579627	-4.466555974
SERPING1	-0.790840869	9.144305563	-1.83636754	0.082653152	-4.467281245
CHI3L1	1.04702941	10.16905298	1.832342485	0.083274641	-4.473384241
FOS	-0.73298083	10.50980034	-1.828011445	0.083947953	-4.479941212
PNMAL1	-0.862913886	5.285443935	-1.826722331	0.08414928	-4.481890862
PLD6	-0.648316746	4.136457328	-1.820402553	0.08514241	-4.491435544
ECM1	-0.695657071	3.980817774	-1.814370778	0.086099841	-4.500524561
LOC100507557	-0.661932532	4.909905465	-1.803596275	0.087833552	-4.516709632
RGS16	-0.894682326	6.96045809	-1.793109981	0.089550098	-4.532399158
NEFH	-0.602797325	2.530969723	-1.768905122	0.093624365	-4.568376241
SDF2L1	0.788145212	5.731750285	1.762282958	0.094766739	-4.578160764
P4HA3	-0.60061747	4.588384553	-1.750195247	0.096883179	-4.595955604
VCAM1	1.00613448	1.981728171	1.743383894	0.098093728	-4.605945551
ACAT2	-0.606430447	8.105795313	-1.73870722	0.098932451	-4.612788987
CIDEA	-1.157833577	4.694817322	-1.735537246	0.099504479	-4.617420379
LNX1	0.652474837	5.826488453	1.735374057	0.099534004	-4.617658641
SPHK1	0.664246694	4.831613915	1.726408508	0.101167773	-4.6307247
GADD45B	-0.60748828	10.56449483	-1.721828887	0.1020112	-4.63738061
COL1A1	1.561899014	4.413434314	1.721798022	0.102016905	-4.637425427
TCEA3	-0.624307496	6.288904458	-1.720587274	0.10224091	-4.639183006
C15orf48	0.873224733	3.01078111	1.719266519	0.102485751	-4.641099289
LIPG	-0.6224129	2.498899733	-1.718244014	0.102675649	-4.642582131

DNAJB6	0.604636661	8.213520664	1.7096079	0.104291665	-4.65508154
GPR158	-0.896824418	3.392508502	-1.704056233	0.105342029	-4.663093271
TREM1	1.132566215	6.531866671	1.703707578	0.105408296	-4.663595809
PGD	0.790790511	7.113332759	1.693926985	0.107281901	-4.677663531
MCOLN3	0.690854307	6.198549745	1.686985685	0.108628878	-4.68761256
GALNT3	-0.893574203	5.00829656	-1.686404758	0.108742263	-4.688443892
COL12A1	0.724529537	3.06367415	1.685884746	0.108843844	-4.689187878
C3orf14	-0.696886959	7.14521368	-1.684620303	0.109091186	-4.69099625
ABCA1	0.721459629	4.054811049	1.683949502	0.109222599	-4.691955218
HTR2A	0.633295763	3.076335484	1.676227976	0.110745068	-4.702974201
AKAP12	-0.735264442	5.085072905	-1.676149258	0.110760682	-4.703086349
SLCO4A1	0.7803145	4.257542262	1.674936591	0.111001458	-4.70481354
CST3	-0.600317146	9.246796716	-1.669191213	0.112148297	-4.712984473
OGN	-0.8235482	10.72987023	-1.661569689	0.113685245	-4.723792543
FRZB	-0.725953103	9.710007442	-1.65914217	0.114178535	-4.727227542
SRGN	1.24578231	7.337322479	1.649667901	0.116121275	-4.740599239
IFIT1	0.896930185	4.17978396	1.642791658	0.117548842	-4.750269514
H1F0	-0.809126068	8.474223268	-1.633937776	0.119408937	-4.762677875
WISP2	-0.767048191	3.68961009	-1.631234023	0.119981921	-4.766457365
PDGFRA	0.718276129	6.881485687	1.628831711	0.120492978	-4.769811658
S100A4	-0.918014506	9.890372442	-1.620048588	0.122377199	-4.782044639
SERPINF1	0.911183564	2.773237997	1.614887398	0.12349601	-4.789210502
TMEM140	0.775747069	4.238138417	1.605444013	0.125565487	-4.802278407
NOV	-0.872307573	5.7157124	-1.598303393	0.127149696	-4.81212228
BEX5	-0.663806559	8.489763292	-1.594080136	0.12809457	-4.817929126
F5	-0.869858669	3.59286688	-1.582649935	0.130681564	-4.833588282
CILP	-1.282928999	10.93487455	-1.571422753	0.133265218	-4.848887849
MEOX2	0.788794905	2.36591769	1.565669531	0.134605694	-4.856696464
LOC643733	0.611392825	2.178495284	1.559618257	0.136027782	-4.864886523
CD14	0.930312482	5.830440883	1.557150801	0.136611247	-4.868219273
GPX3	0.631663555	11.94040155	1.545123433	0.139485317	-4.88440772
TGFBI	-0.998025778	9.248750478	-1.540645295	0.140568224	-4.890411021
GCA	-0.734279161	5.801770727	-1.534488581	0.142068473	-4.898643127
IFIT2	0.90988601	4.358912579	1.533372022	0.142341976	-4.900133406
ATP8B1	0.621042142	3.122909117	1.525825836	0.144201925	-4.910183813
ELTD1	0.850126076	2.002360633	1.522199895	0.145102785	-4.914999662
KCNK5	0.689635766	8.536153971	1.507166846	0.148887673	-4.934873036
TWIST1	1.218011882	4.774403321	1.503026796	0.149944249	-4.940319669
SFN	-0.712891366	9.802057948	-1.500920316	0.150484215	-4.94308655
PMAIP1	1.125070008	5.462375861	1.499346856	0.150888596	-4.945151366
RSPO3	0.999312087	4.492803702	1.487545375	0.153950269	-4.960585229
BLM	-0.691988303	6.05098946	-1.4858561	0.154392678	-4.96278678
FRMD6-AS1	-0.632020041	5.358616867	-1.467946266	0.159147781	-4.986009097
DUSP4	0.729761022	5.331767513	1.462045408	0.160740536	-4.993612563
SPINK1	-0.895545196	4.226659066	-1.461878823	0.16078569	-4.993826869
PRSS23	-0.688082114	8.001956368	-1.459231592	0.161504622	-4.997229896
TNFAIP3	0.816330263	4.735620665	1.455703166	0.162466957	-5.001758255
TF	1.148324759	5.834584753	1.453833063	0.162978902	-5.004154876
MAMDC2	0.859841288	2.824469981	1.451183406	0.163706508	-5.007546423
S100A8	0.801524528	2.371766442	1.442874045	0.166005506	-5.018151071
ORM1	0.760501199	10.07472243	1.440126421	0.166771472	-5.021647205
OLFML2A	-0.624998921	2.634443024	-1.417993943	0.173046868	-5.049618322
ABCB4	-0.729041974	4.261912971	-1.3965101	0.17931998	-5.076442666

ECHDC3	-0.610102389	3.624662281	-1.390306691	0.181165048	-5.0841277
NPTX2	1.025810715	5.708976578	1.37966759	0.184364936	-5.097244408
ACVR1C	-0.674516869	4.058362309	-1.365105233	0.188818118	-5.115067425
NUP62CL	-0.640393618	5.508595751	-1.3567832	0.191401354	-5.125184717
SPOCK1	-0.687837487	6.536665631	-1.351999834	0.192898861	-5.130977445
HBD	0.962478241	2.713908188	1.348692712	0.193939651	-5.134972781
ENO2	-0.69249002	7.052139171	-1.343363863	0.195626095	-5.141393954
MMP13	0.803193923	2.431184909	1.327560179	0.200696092	-5.160316109
SGCG	-0.735159353	4.215024694	-1.319611573	0.203285091	-5.169764457
CYP1A1	0.704437875	3.703156002	1.296928923	0.210818183	-5.196472163
MAFB	0.685257313	9.001363568	1.287416639	0.214041724	-5.207559457
HBB	1.715659644	6.045222103	1.287035605	0.214171648	-5.208002185
XIST	-2.153036302	5.572347819	-1.28110053	0.216203339	-5.21488428
COCH	-0.877505296	3.194703815	-1.279956599	0.216596652	-5.216207728
CSRP2	-0.60451921	6.853436888	-1.277866737	0.217316641	-5.218623036
DDX3Y	1.650926795	6.173892428	1.266968366	0.221101549	-5.231165739
CAPN8	-0.698371916	3.429126217	-1.263726605	0.222237213	-5.234879465
EFEMP1	0.709831138	2.923384874	1.257078708	0.224580277	-5.242470551
ASPN	-1.281810393	7.229044445	-1.246827217	0.228230867	-5.254111302
PTX3	0.628749122	9.081778302	1.207900584	0.242510498	-5.297586915
PSMD5-AS1	-0.604654797	4.446621638	-1.198382852	0.246103544	-5.308040499
MAOA	-0.844258958	3.933539367	-1.163851325	0.259479152	-5.345378735
LAMB3	-0.854947648	5.837784052	-1.139902175	0.269071439	-5.370727378
MXRA5	-0.691795937	4.958971456	-1.139007716	0.269434742	-5.371665356
GPNMB	0.853281917	3.930335782	1.135315329	0.270938346	-5.3755307
TAC1	-0.920534623	6.089479029	-1.094415975	0.28801121	-5.417620724
THBD	0.621734241	6.694403231	1.094177378	0.288113069	-5.417862343
EPYC	1.068359299	6.958993118	1.074742291	0.296498638	-5.43738942
COL15A1	0.881012439	5.303107181	1.029913415	0.316511535	-5.481260181
EIF1AY	1.327561734	5.501764709	1.017677087	0.322137632	-5.49294867
FABP4	0.744000157	3.381845807	1.010666005	0.325392934	-5.499590096
POSTN	1.176463468	4.245419841	0.992036553	0.334155137	-5.517039075
RPS4Y1	1.733505767	7.98573024	0.985671534	0.337186347	-5.522934497
KDM5D	1.046614283	4.718077983	0.951531943	0.353770852	-5.553975409
USP9Y	0.77481378	3.656179028	0.919395961	0.369884819	-5.582294084
PAX8-AS1	-0.621137587	5.392151753	-0.885678382	0.3873155	-5.611057476
AGR2	0.684029639	5.914248162	0.840887816	0.411296438	-5.647746538
CCL20	0.658580958	6.44383656	0.745352418	0.465553269	-5.720105722
PRG4	0.801002282	9.763376807	0.738371004	0.46968127	-5.725074374
