# Supplementary material for: CircEYA3 aggravates intervertebral disc degeneration through the miR-196a-5p/EBF1 axis and NF-κB signaling
Source: Commun Biol. 2024 Mar 30;7:390. doi: 10.1038/s42003-024-06055-2 (PMC10981674; doi:10.1038/s42003-024-06055-2)
Supplement: Supplementary file 7 — Supplementary Data 5 [file 42003_2024_6055_MOESM7_ESM.pdf]

Supplementary data 5. The regulatory network

miRNA	mRNA	Target	mirnaLogFC	mrnaLogFC
hsa-miR-93-5p	ZFPM2	target	4.022878217	-1.118446022
hsa-miR-378h	ZFPM2	target	2.142129164	-1.118446022
hsa-miR-378c	ZFPM2	target	2.172121133	-1.118446022
hsa-miR-301a-3p	ZFPM2	target	4.377116122	-1.118446022
hsa-miR-103a-3p	ZFPM2	target	1.973244744	-1.118446022
hsa-miR-96-5p	ZFP36L1	target	-3.69065925	1.528745549
hsa-miR-377-3p	ZFP36L1	target	-2.083955044	1.528745549
hsa-miR-29b-3p	ZFP36L1	target	-3.614460994	1.528745549
hsa-miR-29a-3p	ZFP36L1	target	-7.710169639	1.528745549
hsa-miR-27b-3p	ZFP36L1	target	-8.0156531	1.528745549
hsa-miR-27a-3p	ZFP36L1	target	-7.027874406	1.528745549
hsa-miR-181d-5p	ZFP36L1	target	-2.538257378	1.528745549
hsa-miR-181c-5p	ZFP36L1	target	-5.975134372	1.528745549
hsa-miR-181b-5p	ZFP36L1	target	-4.229699617	1.528745549
hsa-miR-181a-5p	ZFP36L1	target	-8.086162172	1.528745549
hsa-miR-128-3p	ZFP36L1	target	-3.2456183	1.528745549
hsa-miR-1271-5p	ZFP36L1	target	-3.057933239	1.528745549
hsa-miR-532-3p	ZEB2	target	-3.840000894	1.650922664
hsa-miR-502-3p	ZEB2	target	-2.727154031	1.650922664
hsa-miR-377-3p	ZEB2	target	-2.083955044	1.650922664
hsa-miR-16-5p	WIF1	target	2.170767889	-1.715006608
hsa-miR-16-5p	VAMP8	target	2.170767889	-1.572016478
hsa-miR-103a-3p	VAMP8	target	1.973244744	-1.572016478
hsa-miR-96-5p	TWIST1	target	-3.69065925	1.218011882
hsa-miR-214-5p	TWIST1	target	-3.476266878	1.218011882
hsa-miR-151a-3p	TWIST1	target	-4.3826325	1.218011882
hsa-miR-1271-5p	TWIST1	target	-3.057933239	1.218011882
hsa-miR-23b-3p	TNFAIP6	target	-13.60579603	1.376896967
hsa-miR-23a-3p	TNFAIP6	target	-14.74406994	1.376896967
hsa-miR-27b-3p	TFPI	target	-8.0156531	2.055735683
hsa-miR-27a-3p	TFPI	target	-7.027874406	2.055735683
hsa-miR-374c-5p	TAF13	target	-2.066745369	1.061927181
hsa-miR-454-3p	SULF1	target	-1.793108858	1.096191063
hsa-miR-19a-3p	SULF1	target	-2.335140461	1.096191063
hsa-miR-199b-5p	SULF1	target	-6.704986694	1.096191063
hsa-miR-199a-5p	SULF1	target	-7.464223567	1.096191063
hsa-miR-152-3p	SULF1	target	-5.366644456	1.096191063
hsa-miR-148b-3p	SULF1	target	-3.844370844	1.096191063
hsa-miR-148a-3p	SULF1	target	-6.9178194	1.096191063
hsa-miR-23c	SPTSSB	target	3.754610411	-1.26286057
hsa-miR-182-5p	SPTSSB	target	2.937748733	-1.26286057
hsa-miR-454-3p	SOX4	target	-1.793108858	1.31424054
hsa-miR-3666	SOX4	target	-1.888857728	1.31424054
hsa-miR-214-5p	SOX4	target	-3.476266878	1.31424054
hsa-miR-20b-5p	SOX4	target	-2.910447375	1.31424054
hsa-miR-20a-5p	SOX4	target	-3.760302156	1.31424054
hsa-miR-19b-3p	SOX4	target	-5.130085539	1.31424054
hsa-miR-19a-3p	SOX4	target	-2.335140461	1.31424054
hsa-miR-17-5p	SOX4	target	-2.907048831	1.31424054
hsa-miR-140-5p	SOX4	target	-7.6651035	1.31424054
hsa-miR-132-3p	SOX4	target	-3.373475364	1.31424054

hsa-miR-130b-3p	SOX4	target	-5.001102139	1.31424054
hsa-miR-130a-3p	SOX4	target	-7.083430956	1.31424054
hsa-miR-106b-5p	SOX4	target	-5.687222906	1.31424054
hsa-miR-98-5p	SMIM3	target	-5.885407408	2.303470985
hsa-let-7i-5p	SMIM3	target	-10.44359602	2.303470985
hsa-let-7g-5p	SMIM3	target	-9.970715511	2.303470985
hsa-let-7f-5p	SMIM3	target	-12.84678967	2.303470985
hsa-let-7e-5p	SMIM3	target	-8.750119272	2.303470985
hsa-let-7c-5p	SMIM3	target	-13.21630578	2.303470985
hsa-let-7b-5p	SMIM3	target	-25.01169358	2.303470985
hsa-let-7a-5p	SMIM3	target	-13.68176841	2.303470985
hsa-miR-324-5p	SLITRK4	target	-3.233792569	1.151402046
hsa-miR-145-5p	SLITRK4	target	-4.396319578	1.151402046
hsa-miR-194-5p	SLC40A1	target	-1.448772494	1.240892822
hsa-miR-222-3p	SLC16A6	target	-4.5975339	1.239865427
hsa-miR-221-3p	SLC16A6	target	-7.122492578	1.239865427
hsa-miR-20b-5p	SLC16A6	target	-2.910447375	1.239865427
hsa-miR-20a-5p	SLC16A6	target	-3.760302156	1.239865427
hsa-miR-193b-3p	SLC16A6	target	-4.598413106	1.239865427
hsa-miR-193a-3p	SLC16A6	target	-1.549934411	1.239865427
hsa-miR-17-5p	SLC16A6	target	-2.907048831	1.239865427
hsa-miR-152-3p	SLC16A6	target	-5.366644456	1.239865427
hsa-miR-148b-3p	SLC16A6	target	-3.844370844	1.239865427
hsa-miR-148a-3p	SLC16A6	target	-6.9178194	1.239865427
hsa-miR-106b-5p	SLC16A6	target	-5.687222906	1.239865427
hsa-miR-92a-3p	S100A2	target	2.653037772	-1.563529121
hsa-miR-19b-3p	RIN2	target	-5.130085539	1.33630252
hsa-miR-19a-3p	RIN2	target	-2.335140461	1.33630252
hsa-miR-144-3p	RIN2	target	-2.246704806	1.33630252
hsa-miR-223-3p	RERG	target	-5.561533161	1.024758116
hsa-miR-182-5p	RAPGEF5	target	2.937748733	-1.172797805
hsa-miR-502-3p	RAP1A	target	-2.727154031	1.098144622
hsa-miR-320d	RAP1A	target	-6.863046828	1.098144622
hsa-miR-320b	RAP1A	target	-6.736763533	1.098144622
hsa-miR-320a	RAP1A	target	-5.854823167	1.098144622
hsa-miR-24-3p	RAP1A	target	-8.852555428	1.098144622
hsa-miR-19b-3p	RAP1A	target	-5.130085539	1.098144622
hsa-miR-19a-3p	RAP1A	target	-2.335140461	1.098144622
hsa-miR-149-5p	RAP1A	target	-3.016498922	1.098144622
hsa-miR-454-3p	PXDN	target	-1.793108858	1.143151734
hsa-miR-29b-3p	PXDN	target	-3.614460994	1.143151734
hsa-miR-29a-3p	PXDN	target	-7.710169639	1.143151734
hsa-miR-19b-3p	PXDN	target	-5.130085539	1.143151734
hsa-miR-19a-3p	PXDN	target	-2.335140461	1.143151734
hsa-miR-19b-3p	POSTN	target	-5.130085539	1.176463468
hsa-miR-19a-3p	POSTN	target	-2.335140461	1.176463468
hsa-miR-98-5p	PMAIP1	target	-5.885407408	1.125070008
hsa-miR-23b-3p	PMAIP1	target	-13.60579603	1.125070008
hsa-miR-23a-3p	PMAIP1	target	-14.74406994	1.125070008
hsa-miR-144-3p	PMAIP1	target	-2.246704806	1.125070008
hsa-miR-142-3p	PMAIP1	target	-2.005220017	1.125070008
hsa-let-7i-5p	PMAIP1	target	-10.44359602	1.125070008
hsa-let-7g-5p	PMAIP1	target	-9.970715511	1.125070008

hsa-let-7f-5p	PMAIP1	target	-12.84678967	1.125070008
hsa-let-7e-5p	PMAIP1	target	-8.750119272	1.125070008
hsa-let-7c-5p	PMAIP1	target	-13.21630578	1.125070008
hsa-let-7b-5p	PMAIP1	target	-25.01169358	1.125070008
hsa-let-7a-5p	PMAIP1	target	-13.68176841	1.125070008
hsa-miR-92a-3p	PALLD	target	2.653037772	-1.196309679
hsa-miR-34a-5p	PALLD	target	17.52203323	-1.196309679
hsa-miR-182-5p	PALLD	target	2.937748733	-1.196309679
hsa-miR-29c-3p	NUDT11	target	21.99464321	-1.007698068
hsa-miR-1271-5p	NPTX2	target	-3.057933239	1.025810715
hsa-miR-92a-3p	NEFM	target	2.653037772	-1.383725998
hsa-let-7d-5p	NEFM	target	1.549151417	-1.383725998
hsa-miR-96-5p	NANOS1	target	-3.69065925	1.252369519
hsa-miR-708-5p	NANOS1	target	-4.858929633	1.252369519
hsa-miR-29b-3p	NANOS1	target	-3.614460994	1.252369519
hsa-miR-29a-3p	NANOS1	target	-7.710169639	1.252369519
hsa-miR-28-5p	NANOS1	target	-2.831242669	1.252369519
hsa-miR-20b-5p	NANOS1	target	-2.910447375	1.252369519
hsa-miR-20a-5p	NANOS1	target	-3.760302156	1.252369519
hsa-miR-17-5p	NANOS1	target	-2.907048831	1.252369519
hsa-miR-1271-5p	NANOS1	target	-3.057933239	1.252369519
hsa-miR-106b-5p	NANOS1	target	-5.687222906	1.252369519
hsa-miR-20b-5p	MMP2	target	-2.910447375	2.159729649
hsa-miR-20a-5p	MMP2	target	-3.760302156	2.159729649
hsa-miR-17-5p	MMP2	target	-2.907048831	2.159729649
hsa-miR-106b-5p	MMP2	target	-5.687222906	2.159729649
hsa-miR-20b-5p	MAP3K8	target	-2.910447375	1.091357104
hsa-miR-20a-5p	MAP3K8	target	-3.760302156	1.091357104
hsa-miR-17-5p	MAP3K8	target	-2.907048831	1.091357104
hsa-miR-144-3p	MAP3K8	target	-2.246704806	1.091357104
hsa-miR-106b-5p	MAP3K8	target	-5.687222906	1.091357104
hsa-miR-425-5p	MAP3K5	target	-3.864192222	1.006143517
hsa-miR-20b-5p	MAP3K5	target	-2.910447375	1.006143517
hsa-miR-20a-5p	MAP3K5	target	-3.760302156	1.006143517
hsa-miR-199a-3p	MAP3K5	target	-8.798220544	1.006143517
hsa-miR-17-5p	MAP3K5	target	-2.907048831	1.006143517
hsa-miR-106b-5p	MAP3K5	target	-5.687222906	1.006143517
hsa-miR-5195-3p	LYVE1	target	6.534320478	-1.50362957
hsa-miR-339-5p	LYVE1	target	5.250190517	-1.50362957
hsa-let-7d-5p	LYVE1	target	1.549151417	-1.50362957
hsa-miR-223-3p	LMO2	target	-5.561533161	1.920074763
hsa-miR-301a-3p	LDLR	target	4.377116122	-1.601830469
hsa-miR-497-5p	KCNN4	target	-6.000204056	1.026644575
hsa-miR-424-5p	KCNN4	target	-2.868822222	1.026644575
hsa-miR-195-5p	KCNN4	target	-7.895415794	1.026644575
hsa-miR-15b-5p	KCNN4	target	-7.4728635	1.026644575
hsa-miR-15a-5p	KCNN4	target	-7.474686522	1.026644575
hsa-miR-374c-5p	IRX5	target	-2.066745369	1.229607855
hsa-miR-222-3p	IRX5	target	-4.5975339	1.229607855
hsa-miR-221-3p	IRX5	target	-7.122492578	1.229607855
hsa-miR-493-5p	IRX3	target	-2.287147097	1.533096142
hsa-miR-377-3p	IRX3	target	-2.083955044	1.533096142
hsa-miR-340-5p	IGFBP3	target	-2.299785889	3.236412271

hsa-miR-19b-3p	IGFBP3	target	-5.130085539	3.236412271
hsa-miR-19a-3p	IGFBP3	target	-2.335140461	3.236412271
hsa-miR-877-5p	HSD11B1	target	-3.979460428	1.48908376
hsa-miR-340-5p	HSD11B1	target	-2.299785889	1.48908376
hsa-miR-132-3p	HSD11B1	target	-3.373475364	1.48908376
hsa-miR-324-5p	hsa_circ_100906	target	-3.233792569	1.03217136
hsa-miR-182-5p	hsa_circ_100896	target	2.937748733	-1.27638136
hsa-miR-339-5p	hsa_circ_100893	target	5.250190517	-1.0679431
hsa-miR-339-5p	hsa_circ_100892	target	5.250190517	-1.00594502
hsa-miR-98-5p	hsa_circ_100882	target	-5.885407408	1.4391607
hsa-miR-665	hsa_circ_100882	target	-1.51720975	1.4391607
hsa-let-7i-5p	hsa_circ_100882	target	-10.44359602	1.4391607
hsa-let-7g-5p	hsa_circ_100882	target	-9.970715511	1.4391607
hsa-let-7f-5p	hsa_circ_100882	target	-12.84678967	1.4391607
hsa-let-7e-5p	hsa_circ_100882	target	-8.750119272	1.4391607
hsa-let-7c-5p	hsa_circ_100882	target	-13.21630578	1.4391607
hsa-let-7b-5p	hsa_circ_100882	target	-25.01169358	1.4391607
hsa-let-7a-5p	hsa_circ_100882	target	-13.68176841	1.4391607
hsa-miR-665	hsa_circ_100876	target	-1.51720975	1.91686344
hsa-miR-320c	hsa_circ_100844	target	1.871100867	-2.38715834
hsa-miR-16-5p	hsa_circ_100844	target	2.170767889	-2.38715834
hsa-miR-103a-3p	hsa_circ_100844	target	1.973244744	-2.38715834
hsa-miR-182-5p	hsa_circ_100836	target	2.937748733	-1.42659808
hsa-miR-520b	hsa_circ_100823	target	7.073403739	-1.22774796
hsa-miR-708-5p	hsa_circ_100815	target	-4.858929633	1.06539044
hsa-miR-29b-3p	hsa_circ_100815	target	-3.614460994	1.06539044
hsa-miR-29a-3p	hsa_circ_100815	target	-7.710169639	1.06539044
hsa-miR-28-5p	hsa_circ_100815	target	-2.831242669	1.06539044
hsa-miR-28-5p	hsa_circ_100815	target	-2.831242669	1.06539044
hsa-miR-665	hsa_circ_100802	target	-1.51720975	1.62299234
hsa-miR-425-5p	hsa_circ_100802	target	-3.864192222	1.62299234
hsa-miR-182-5p	hsa_circ_100772	target	2.937748733	-2.6984196
hsa-miR-16-5p	hsa_circ_100772	target	2.170767889	-2.6984196
hsa-miR-103a-3p	hsa_circ_100772	target	1.973244744	-2.6984196
hsa-miR-760	hsa_circ_100723	target	3.333884622	-1.88863978
hsa-miR-5195-3p	hsa_circ_100723	target	6.534320478	-1.88863978
hsa-miR-455-5p	hsa_circ_100723	target	17.33461372	-1.88863978
hsa-miR-376b-3p	hsa_circ_100723	target	3.651956789	-1.88863978
hsa-miR-301a-3p	hsa_circ_100723	target	4.377116122	-1.88863978
hsa-miR-103a-3p	hsa_circ_100723	target	1.973244744	-1.88863978
hsa-miR-5195-3p	hsa_circ_100701	target	6.534320478	-1.98306782
hsa-miR-134-5p	hsa_circ_100701	target	4.379412022	-1.98306782
hsa-miR-5195-3p	hsa_circ_100698	target	6.534320478	-2.41655144
hsa-miR-134-5p	hsa_circ_100698	target	4.379412022	-2.41655144
hsa-miR-376c-3p	hsa_circ_100688	target	-4.708504289	1.02484966
hsa-miR-98-5p	hsa_circ_100685	target	-5.885407408	1.04794418
hsa-miR-532-5p	hsa_circ_100685	target	-5.295597783	1.04794418
hsa-miR-532-5p	hsa_circ_100685	target	-5.295597783	1.04794418
hsa-miR-377-3p	hsa_circ_100685	target	-2.083955044	1.04794418
hsa-miR-29b-3p	hsa_circ_100685	target	-3.614460994	1.04794418
hsa-miR-29a-3p	hsa_circ_100685	target	-7.710169639	1.04794418
hsa-miR-199a-3p	hsa_circ_100685	target	-8.798220544	1.04794418
hsa-miR-193b-3p	hsa_circ_100685	target	-4.598413106	1.04794418

hsa-miR-193a-3p	hsa_circ_100685	target	-1.549934411	1.04794418
hsa-let-7i-5p	hsa_circ_100685	target	-10.44359602	1.04794418
hsa-let-7g-5p	hsa_circ_100685	target	-9.970715511	1.04794418
hsa-let-7f-5p	hsa_circ_100685	target	-12.84678967	1.04794418
hsa-let-7e-5p	hsa_circ_100685	target	-8.750119272	1.04794418
hsa-let-7c-5p	hsa_circ_100685	target	-13.21630578	1.04794418
hsa-let-7b-5p	hsa_circ_100685	target	-25.01169358	1.04794418
hsa-let-7a-5p	hsa_circ_100685	target	-13.68176841	1.04794418
hsa-miR-98-5p	hsa_circ_100668	target	-5.885407408	1.59573632
hsa-miR-96-5p	hsa_circ_100668	target	-3.69065925	1.59573632
hsa-miR-374c-5p	hsa_circ_100668	target	-2.066745369	1.59573632
hsa-miR-149-5p	hsa_circ_100668	target	-3.016498922	1.59573632
hsa-miR-149-5p	hsa_circ_100668	target	-3.016498922	1.59573632
hsa-miR-143-3p	hsa_circ_100668	target	-2.558538	1.59573632
hsa-miR-132-3p	hsa_circ_100668	target	-3.373475364	1.59573632
hsa-miR-1271-5p	hsa_circ_100668	target	-3.057933239	1.59573632
hsa-miR-101-3p	hsa_circ_100668	target	-4.824183106	1.59573632
hsa-let-7i-5p	hsa_circ_100668	target	-10.44359602	1.59573632
hsa-let-7g-5p	hsa_circ_100668	target	-9.970715511	1.59573632
hsa-let-7f-5p	hsa_circ_100668	target	-12.84678967	1.59573632
hsa-let-7e-5p	hsa_circ_100668	target	-8.750119272	1.59573632
hsa-let-7c-5p	hsa_circ_100668	target	-13.21630578	1.59573632
hsa-let-7b-5p	hsa_circ_100668	target	-25.01169358	1.59573632
hsa-let-7a-5p	hsa_circ_100668	target	-13.68176841	1.59573632
hsa-miR-320d	hsa_circ_100646	target	-6.863046828	2.04021004
hsa-miR-520b	hsa_circ_100640	target	7.073403739	-1.50197352
hsa-miR-5195-3p	hsa_circ_100633	target	6.534320478	-1.81514382
hsa-miR-27b-3p	hsa_circ_100604	target	-8.0156531	2.67199762
hsa-miR-27a-3p	hsa_circ_100604	target	-7.027874406	2.67199762
hsa-miR-144-3p	hsa_circ_100604	target	-2.246704806	2.67199762
hsa-miR-708-5p	hsa_circ_100600	target	-4.858929633	1.03440484
hsa-miR-497-5p	hsa_circ_100600	target	-6.000204056	1.03440484
hsa-miR-424-5p	hsa_circ_100600	target	-2.868822222	1.03440484
hsa-miR-28-5p	hsa_circ_100600	target	-2.831242669	1.03440484
hsa-miR-223-3p	hsa_circ_100600	target	-5.561533161	1.03440484
hsa-miR-195-5p	hsa_circ_100600	target	-7.895415794	1.03440484
hsa-miR-194-5p	hsa_circ_100600	target	-1.448772494	1.03440484
hsa-miR-15b-5p	hsa_circ_100600	target	-7.4728635	1.03440484
hsa-miR-15a-5p	hsa_circ_100600	target	-7.474686522	1.03440484
hsa-miR-383-5p	hsa_circ_100579	target	2.153888914	-1.60890002
hsa-miR-502-3p	hsa_circ_100571	target	-2.727154031	2.00809222
hsa-miR-29b-3p	hsa_circ_100525	target	-3.614460994	1.41262602
hsa-miR-29a-3p	hsa_circ_100525	target	-7.710169639	1.41262602
hsa-miR-134-5p	hsa_circ_100477	target	4.379412022	-1.18776898
hsa-miR-320c	hsa_circ_100476	target	1.871100867	-2.162316
hsa-miR-134-5p	hsa_circ_100476	target	4.379412022	-2.162316
hsa-miR-497-5p	hsa_circ_100470	target	-6.000204056	1.22561234
hsa-miR-424-5p	hsa_circ_100470	target	-2.868822222	1.22561234
hsa-miR-195-5p	hsa_circ_100470	target	-7.895415794	1.22561234
hsa-miR-15b-5p	hsa_circ_100470	target	-7.4728635	1.22561234
hsa-miR-15a-5p	hsa_circ_100470	target	-7.474686522	1.22561234
hsa-miR-152-3p	hsa_circ_100453	target	-5.366644456	1.09279654
hsa-miR-148b-3p	hsa_circ_100453	target	-3.844370844	1.09279654

hsa-miR-148a-3p	hsa_circ_100453	target	-6.9178194	1.09279654
hsa-miR-182-5p	hsa_circ_100443	target	2.937748733	-1.01819448
hsa-miR-374c-5p	hsa_circ_100422	target	-2.066745369	1.57102538
hsa-miR-374c-5p	hsa_circ_100421	target	-2.066745369	1.50703614
hsa-miR-103a-3p	hsa_circ_100420	target	1.973244744	-1.65018748
hsa-miR-103a-3p	hsa_circ_100420	target	1.973244744	-1.65018748
hsa-miR-454-3p	hsa_circ_100411	target	-1.793108858	1.31800384
hsa-miR-3666	hsa_circ_100411	target	-1.888857728	1.31800384
hsa-miR-19b-3p	hsa_circ_100411	target	-5.130085539	1.31800384
hsa-miR-19a-3p	hsa_circ_100411	target	-2.335140461	1.31800384
hsa-miR-152-3p	hsa_circ_100411	target	-5.366644456	1.31800384
hsa-miR-148b-3p	hsa_circ_100411	target	-3.844370844	1.31800384
hsa-miR-148a-3p	hsa_circ_100411	target	-6.9178194	1.31800384
hsa-miR-130b-3p	hsa_circ_100411	target	-5.001102139	1.31800384
hsa-miR-130a-3p	hsa_circ_100411	target	-7.083430956	1.31800384
hsa-miR-93-5p	hsa_circ_100407	target	4.022878217	-1.40093756
hsa-miR-301a-3p	hsa_circ_100407	target	4.377116122	-1.40093756
hsa-miR-181d-5p	hsa_circ_100374	target	-2.538257378	2.11558444
hsa-miR-181c-5p	hsa_circ_100374	target	-5.975134372	2.11558444
hsa-miR-181b-5p	hsa_circ_100374	target	-4.229699617	2.11558444
hsa-miR-181a-5p	hsa_circ_100374	target	-8.086162172	2.11558444
hsa-miR-196b-5p	hsa_circ_100350	target	-3.858799039	1.10611924
hsa-miR-196a-5p	hsa_circ_100350	target	-6.248512556	1.10611924
hsa-miR-212-3p	hsa_circ_100332	target	4.252929717	-1.285155
hsa-miR-23b-3p	hsa_circ_100329	target	-13.60579603	1.45547144
hsa-miR-23a-3p	hsa_circ_100329	target	-14.74406994	1.45547144
hsa-miR-455-5p	hsa_circ_100290	target	17.33461372	-2.1012706
hsa-miR-29c-3p	hsa_circ_100290	target	21.99464321	-2.1012706
hsa-miR-29c-3p	hsa_circ_100290	target	21.99464321	-2.1012706
hsa-miR-383-5p	hsa_circ_100283	target	2.153888914	-1.17878776
hsa-miR-34a-5p	hsa_circ_100270	target	17.52203323	-1.4706532
hsa-miR-378h	hsa_circ_100266	target	2.142129164	-1.0576901
hsa-miR-378c	hsa_circ_100266	target	2.172121133	-1.0576901
hsa-miR-877-5p	hsa_circ_100236	target	-3.979460428	1.52880272
hsa-miR-143-3p	hsa_circ_100236	target	-2.558538	1.52880272
hsa-miR-24-3p	hsa_circ_100227	target	-8.852555428	1.87763952
hsa-miR-24-3p	hsa_circ_100227	target	-8.852555428	1.87763952
hsa-miR-24-3p	hsa_circ_100226	target	-8.852555428	1.5606671
hsa-miR-24-3p	hsa_circ_100226	target	-8.852555428	1.5606671
hsa-miR-199a-3p	hsa_circ_100226	target	-8.798220544	1.5606671
hsa-miR-194-5p	hsa_circ_100226	target	-1.448772494	1.5606671
hsa-miR-378h	hsa_circ_100219	target	2.142129164	-1.2632643
hsa-miR-378c	hsa_circ_100219	target	2.172121133	-1.2632643
hsa-miR-182-5p	hsa_circ_100219	target	2.937748733	-1.2632643
hsa-miR-378h	hsa_circ_100213	target	2.142129164	-1.63603092
hsa-miR-378c	hsa_circ_100213	target	2.172121133	-1.63603092
hsa-miR-34a-5p	hsa_circ_100213	target	17.52203323	-1.63603092
hsa-miR-320c	hsa_circ_100213	target	1.871100867	-1.63603092
hsa-miR-128-3p	hsa_circ_100202	target	-3.2456183	1.63754254
hsa-miR-760	hsa_circ_100188	target	3.333884622	-1.29563954
hsa-miR-378h	hsa_circ_100188	target	2.142129164	-1.29563954
hsa-miR-378c	hsa_circ_100188	target	2.172121133	-1.29563954
hsa-miR-301a-3p	hsa_circ_100188	target	4.377116122	-1.29563954

hsa-miR-29c-3p	hsa_circ_100188	target	21.99464321	-1.29563954
hsa-miR-29c-3p	hsa_circ_100188	target	21.99464321	-1.29563954
hsa-miR-16-5p	hsa_circ_100188	target	2.170767889	-1.29563954
hsa-miR-134-5p	hsa_circ_100188	target	4.379412022	-1.29563954
hsa-miR-103a-3p	hsa_circ_100188	target	1.973244744	-1.29563954
hsa-miR-96-5p	hsa_circ_100177	target	-3.69065925	1.39822884
hsa-miR-1271-5p	hsa_circ_100177	target	-3.057933239	1.39822884
hsa-miR-98-5p	hsa_circ_100117	target	-5.885407408	1.66873586
hsa-miR-196b-5p	hsa_circ_100117	target	-3.858799039	1.66873586
hsa-miR-196a-5p	hsa_circ_100117	target	-6.248512556	1.66873586
hsa-miR-142-3p	hsa_circ_100117	target	-2.005220017	1.66873586
hsa-let-7i-5p	hsa_circ_100117	target	-10.44359602	1.66873586
hsa-let-7g-5p	hsa_circ_100117	target	-9.970715511	1.66873586
hsa-let-7f-5p	hsa_circ_100117	target	-12.84678967	1.66873586
hsa-let-7e-5p	hsa_circ_100117	target	-8.750119272	1.66873586
hsa-let-7c-5p	hsa_circ_100117	target	-13.21630578	1.66873586
hsa-let-7b-5p	hsa_circ_100117	target	-25.01169358	1.66873586
hsa-let-7a-5p	hsa_circ_100117	target	-13.68176841	1.66873586
hsa-miR-151a-3p	hsa_circ_100086	target	-4.3826325	2.23862012
hsa-miR-151a-3p	hsa_circ_100085	target	-4.3826325	2.0592209
hsa-miR-497-5p	hsa_circ_100045	target	-6.000204056	1.2146054
hsa-miR-493-5p	hsa_circ_100045	target	-2.287147097	1.2146054
hsa-miR-424-5p	hsa_circ_100045	target	-2.868822222	1.2146054
hsa-miR-195-5p	hsa_circ_100045	target	-7.895415794	1.2146054
hsa-miR-15b-5p	hsa_circ_100045	target	-7.4728635	1.2146054
hsa-miR-15a-5p	hsa_circ_100045	target	-7.474686522	1.2146054
hsa-miR-16-5p	hsa_circ_100037	target	2.170767889	-1.43572602
hsa-miR-16-5p	hsa_circ_100037	target	2.170767889	-1.43572602
hsa-miR-103a-3p	hsa_circ_100037	target	1.973244744	-1.43572602
hsa-miR-16-5p	hsa_circ_100036	target	2.170767889	-1.16736316
hsa-miR-16-5p	hsa_circ_100036	target	2.170767889	-1.16736316
hsa-miR-103a-3p	hsa_circ_100036	target	1.973244744	-1.16736316
hsa-miR-16-5p	hsa_circ_100035	target	2.170767889	-1.51137716
hsa-miR-16-5p	hsa_circ_100035	target	2.170767889	-1.51137716
hsa-miR-103a-3p	hsa_circ_100035	target	1.973244744	-1.51137716
hsa-miR-16-5p	hsa_circ_100034	target	2.170767889	-1.56709236
hsa-miR-16-5p	hsa_circ_100034	target	2.170767889	-1.56709236
hsa-miR-103a-3p	hsa_circ_100034	target	1.973244744	-1.56709236
hsa-miR-16-5p	hsa_circ_100033	target	2.170767889	-1.31053668
hsa-miR-760	hsa_circ_100004	target	3.333884622	-1.17333056
hsa-miR-520b	hsa_circ_100004	target	7.073403739	-1.17333056
hsa-miR-103a-3p	hsa_circ_002106	target	1.973244744	-1.03718066
hsa-miR-93-5p	hsa_circ_002086	target	4.022878217	-2.11371662
hsa-miR-520b	hsa_circ_002086	target	7.073403739	-2.11371662
hsa-miR-5195-3p	hsa_circ_002086	target	6.534320478	-2.11371662
hsa-miR-16-5p	hsa_circ_002086	target	2.170767889	-2.11371662
hsa-miR-92a-3p	hsa_circ_001769	target	2.653037772	-1.1074031
hsa-miR-16-5p	hsa_circ_001769	target	2.170767889	-1.1074031
hsa-miR-377-3p	hsa_circ_001653	target	-2.083955044	2.22151978
hsa-miR-152-3p	hsa_circ_001653	target	-5.366644456	2.22151978
hsa-miR-148b-3p	hsa_circ_001653	target	-3.844370844	2.22151978
hsa-miR-148a-3p	hsa_circ_001653	target	-6.9178194	2.22151978
hsa-miR-502-3p	hsa_circ_001405	target	-2.727154031	1.49576896

hsa-miR-377-3p	hsa_circ_001405	target	-2.083955044	1.49576896
hsa-miR-532-3p	hsa_circ_001175	target	-3.840000894	2.28857146
hsa-miR-497-5p	hsa_circ_001175	target	-6.000204056	2.28857146
hsa-miR-497-5p	hsa_circ_001175	target	-6.000204056	2.28857146
hsa-miR-424-5p	hsa_circ_001175	target	-2.868822222	2.28857146
hsa-miR-424-5p	hsa_circ_001175	target	-2.868822222	2.28857146
hsa-miR-376c-3p	hsa_circ_001175	target	-4.708504289	2.28857146
hsa-miR-374c-5p	hsa_circ_001175	target	-2.066745369	2.28857146
hsa-miR-374c-5p	hsa_circ_001175	target	-2.066745369	2.28857146
hsa-miR-340-5p	hsa_circ_001175	target	-2.299785889	2.28857146
hsa-miR-320d	hsa_circ_001175	target	-6.863046828	2.28857146
hsa-miR-320b	hsa_circ_001175	target	-6.736763533	2.28857146
hsa-miR-320a	hsa_circ_001175	target	-5.854823167	2.28857146
hsa-miR-27b-3p	hsa_circ_001175	target	-8.0156531	2.28857146
hsa-miR-27a-3p	hsa_circ_001175	target	-7.027874406	2.28857146
hsa-miR-222-3p	hsa_circ_001175	target	-4.5975339	2.28857146
hsa-miR-221-3p	hsa_circ_001175	target	-7.122492578	2.28857146
hsa-miR-218-5p	hsa_circ_001175	target	-2.920309808	2.28857146
hsa-miR-214-5p	hsa_circ_001175	target	-3.476266878	2.28857146
hsa-miR-20b-5p	hsa_circ_001175	target	-2.910447375	2.28857146
hsa-miR-20a-5p	hsa_circ_001175	target	-3.760302156	2.28857146
hsa-miR-19b-3p	hsa_circ_001175	target	-5.130085539	2.28857146
hsa-miR-19b-3p	hsa_circ_001175	target	-5.130085539	2.28857146
hsa-miR-19a-3p	hsa_circ_001175	target	-2.335140461	2.28857146
hsa-miR-19a-3p	hsa_circ_001175	target	-2.335140461	2.28857146
hsa-miR-199b-5p	hsa_circ_001175	target	-6.704986694	2.28857146
hsa-miR-199b-5p	hsa_circ_001175	target	-6.704986694	2.28857146
hsa-miR-199a-5p	hsa_circ_001175	target	-7.464223567	2.28857146
hsa-miR-199a-5p	hsa_circ_001175	target	-7.464223567	2.28857146
hsa-miR-195-5p	hsa_circ_001175	target	-7.895415794	2.28857146
hsa-miR-195-5p	hsa_circ_001175	target	-7.895415794	2.28857146
hsa-miR-194-5p	hsa_circ_001175	target	-1.448772494	2.28857146
hsa-miR-181d-5p	hsa_circ_001175	target	-2.538257378	2.28857146
hsa-miR-181d-5p	hsa_circ_001175	target	-2.538257378	2.28857146
hsa-miR-181c-5p	hsa_circ_001175	target	-5.975134372	2.28857146
hsa-miR-181c-5p	hsa_circ_001175	target	-5.975134372	2.28857146
hsa-miR-181b-5p	hsa_circ_001175	target	-4.229699617	2.28857146
hsa-miR-181b-5p	hsa_circ_001175	target	-4.229699617	2.28857146
hsa-miR-181a-5p	hsa_circ_001175	target	-8.086162172	2.28857146
hsa-miR-181a-5p	hsa_circ_001175	target	-8.086162172	2.28857146
hsa-miR-17-5p	hsa_circ_001175	target	-2.907048831	2.28857146
hsa-miR-15b-5p	hsa_circ_001175	target	-7.4728635	2.28857146
hsa-miR-15b-5p	hsa_circ_001175	target	-7.4728635	2.28857146
hsa-miR-15a-5p	hsa_circ_001175	target	-7.474686522	2.28857146
hsa-miR-15a-5p	hsa_circ_001175	target	-7.474686522	2.28857146
hsa-miR-151a-3p	hsa_circ_001175	target	-4.3826325	2.28857146
hsa-miR-145-5p	hsa_circ_001175	target	-4.396319578	2.28857146
hsa-miR-143-3p	hsa_circ_001175	target	-2.558538	2.28857146
hsa-miR-142-3p	hsa_circ_001175	target	-2.005220017	2.28857146
hsa-miR-128-3p	hsa_circ_001175	target	-3.2456183	2.28857146
hsa-miR-106b-5p	hsa_circ_001175	target	-5.687222906	2.28857146
hsa-miR-331-3p	hsa_circ_001109	target	-3.481406728	1.1582763
hsa-miR-206	hsa_circ_001109	target	-3.869800733	1.1582763

hsa-miR-1-3p	hsa_circ_001109	target	-3.903586272	1.1582763
hsa-miR-324-5p	hsa_circ_001100	target	-3.233792569	1.2243097
hsa-miR-23c	hsa_circ_001072	target	3.754610411	-1.28896542
hsa-miR-199b-5p	hsa_circ_001046	target	-6.704986694	1.3837194
hsa-miR-199a-5p	hsa_circ_001046	target	-7.464223567	1.3837194
hsa-miR-143-3p	hsa_circ_001046	target	-2.558538	1.3837194
hsa-miR-383-5p	hsa_circ_000943	target	2.153888914	-2.56591412
hsa-miR-16-5p	hsa_circ_000941	target	2.170767889	-2.26248584
hsa-miR-320d	hsa_circ_000926	target	-6.863046828	1.08593422
hsa-miR-320d	hsa_circ_000926	target	-6.863046828	1.08593422
hsa-miR-320b	hsa_circ_000926	target	-6.736763533	1.08593422
hsa-miR-320b	hsa_circ_000926	target	-6.736763533	1.08593422
hsa-miR-320a	hsa_circ_000926	target	-5.854823167	1.08593422
hsa-miR-320a	hsa_circ_000926	target	-5.854823167	1.08593422
hsa-miR-222-3p	hsa_circ_000926	target	-4.5975339	1.08593422
hsa-miR-221-3p	hsa_circ_000926	target	-7.122492578	1.08593422
hsa-miR-140-5p	hsa_circ_000926	target	-7.6651035	1.08593422
hsa-miR-149-5p	hsa_circ_000881	target	-3.016498922	2.02632116
hsa-miR-493-5p	hsa_circ_000791	target	-2.287147097	1.116836
hsa-miR-425-5p	hsa_circ_000791	target	-3.864192222	1.116836
hsa-miR-376c-3p	hsa_circ_000791	target	-4.708504289	1.116836
hsa-miR-340-5p	hsa_circ_000791	target	-2.299785889	1.116836
hsa-miR-23c	hsa_circ_000750	target	3.754610411	-2.3494982
hsa-miR-98-5p	hsa_circ_000684	target	-5.885407408	1.38981146
hsa-let-7i-5p	hsa_circ_000684	target	-10.44359602	1.38981146
hsa-let-7g-5p	hsa_circ_000684	target	-9.970715511	1.38981146
hsa-let-7f-5p	hsa_circ_000684	target	-12.84678967	1.38981146
hsa-let-7e-5p	hsa_circ_000684	target	-8.750119272	1.38981146
hsa-let-7c-5p	hsa_circ_000684	target	-13.21630578	1.38981146
hsa-let-7b-5p	hsa_circ_000684	target	-25.01169358	1.38981146
hsa-let-7a-5p	hsa_circ_000684	target	-13.68176841	1.38981146
hsa-miR-222-3p	hsa_circ_000200	target	-4.5975339	2.48946402
hsa-miR-221-3p	hsa_circ_000200	target	-7.122492578	2.48946402
hsa-miR-132-3p	hsa_circ_000200	target	-3.373475364	2.48946402
hsa-miR-708-5p	hsa_circ_000178	target	-4.858929633	2.45118972
hsa-miR-497-5p	hsa_circ_000178	target	-6.000204056	2.45118972
hsa-miR-424-5p	hsa_circ_000178	target	-2.868822222	2.45118972
hsa-miR-28-5p	hsa_circ_000178	target	-2.831242669	2.45118972
hsa-miR-195-5p	hsa_circ_000178	target	-7.895415794	2.45118972
hsa-miR-15b-5p	hsa_circ_000178	target	-7.4728635	2.45118972
hsa-miR-15a-5p	hsa_circ_000178	target	-7.474686522	2.45118972
hsa-miR-149-5p	hsa_circ_000178	target	-3.016498922	2.45118972
hsa-let-7d-5p	hsa_circ_000094	target	1.549151417	-1.7381442
hsa-let-7d-5p	hsa_circ_000094	target	1.549151417	-1.7381442
hsa-miR-760	HIST1H2AE	target	3.333884622	-1.478890701
hsa-miR-93-5p	HAS2	target	4.022878217	-1.435044745
hsa-miR-92a-3p	HAS2	target	2.653037772	-1.435044745
hsa-miR-455-5p	HAS2	target	17.33461372	-1.435044745
hsa-miR-376b-3p	HAS2	target	3.651956789	-1.435044745
hsa-miR-23c	HAS2	target	3.754610411	-1.435044745
hsa-miR-212-3p	HAS2	target	4.252929717	-1.435044745
hsa-miR-182-5p	HAS2	target	2.937748733	-1.435044745
hsa-miR-16-5p	HAS2	target	2.170767889	-1.435044745

hsa-miR-134-5p	HAS2	target	4.379412022	-1.435044745
hsa-let-7d-5p	HAS2	target	1.549151417	-1.435044745
hsa-miR-218-5p	GREM1	target	-2.920309808	1.224310377
hsa-miR-199a-3p	GREM1	target	-8.798220544	1.224310377
hsa-miR-193b-3p	GREM1	target	-4.598413106	1.224310377
hsa-miR-193a-3p	GREM1	target	-1.549934411	1.224310377
hsa-miR-142-3p	GREM1	target	-2.005220017	1.224310377
hsa-miR-128-3p	GREM1	target	-3.2456183	1.224310377
hsa-miR-532-5p	GBP1	target	-5.295597783	1.03638574
hsa-miR-27b-3p	GATA6	target	-8.0156531	1.903094593
hsa-miR-27a-3p	GATA6	target	-7.027874406	1.903094593
hsa-miR-196b-5p	GATA6	target	-3.858799039	1.903094593
hsa-miR-196a-5p	GATA6	target	-6.248512556	1.903094593
hsa-miR-181d-5p	GATA6	target	-2.538257378	1.903094593
hsa-miR-181c-5p	GATA6	target	-5.975134372	1.903094593
hsa-miR-181b-5p	GATA6	target	-4.229699617	1.903094593
hsa-miR-181a-5p	GATA6	target	-8.086162172	1.903094593
hsa-miR-128-3p	GATA6	target	-3.2456183	1.903094593
hsa-miR-93-5p	FOXQ1	target	4.022878217	-1.382805735
hsa-miR-320c	FOXQ1	target	1.871100867	-1.382805735
hsa-miR-182-5p	FOXQ1	target	2.937748733	-1.382805735
hsa-miR-520b	FOXF2	target	7.073403739	-1.394098208
hsa-miR-301a-3p	FOXF2	target	4.377116122	-1.394098208
hsa-miR-182-5p	FOXF2	target	2.937748733	-1.394098208
hsa-miR-497-5p	FAT4	target	-6.000204056	1.446208783
hsa-miR-424-5p	FAT4	target	-2.868822222	1.446208783
hsa-miR-195-5p	FAT4	target	-7.895415794	1.446208783
hsa-miR-193b-3p	FAT4	target	-4.598413106	1.446208783
hsa-miR-193a-3p	FAT4	target	-1.549934411	1.446208783
hsa-miR-15b-5p	FAT4	target	-7.4728635	1.446208783
hsa-miR-15a-5p	FAT4	target	-7.474686522	1.446208783
hsa-miR-144-3p	FAT4	target	-2.246704806	1.446208783
hsa-miR-29c-3p	ENPP2	target	21.99464321	-1.518038678
hsa-miR-196b-5p	EBF1	target	-3.858799039	1.498717114
hsa-miR-196a-5p	EBF1	target	-6.248512556	1.498717114
hsa-miR-331-3p	DUSP5	target	-3.481406728	1.00414603
hsa-miR-27b-3p	DUSP5	target	-8.0156531	1.00414603
hsa-miR-27a-3p	DUSP5	target	-7.027874406	1.00414603
hsa-miR-23b-3p	DUSP5	target	-13.60579603	1.00414603
hsa-miR-23a-3p	DUSP5	target	-14.74406994	1.00414603
hsa-miR-218-5p	DUSP5	target	-2.920309808	1.00414603
hsa-miR-199a-3p	DUSP5	target	-8.798220544	1.00414603
hsa-miR-128-3p	DUSP5	target	-3.2456183	1.00414603
hsa-miR-19b-3p	DMXL2	target	-5.130085539	1.210445757
hsa-miR-19a-3p	DMXL2	target	-2.335140461	1.210445757
hsa-miR-665	DLX3	target	-1.51720975	1.147831995
hsa-miR-19b-3p	DLX3	target	-5.130085539	1.147831995
hsa-miR-19a-3p	DLX3	target	-2.335140461	1.147831995
hsa-miR-520b	DKK1	target	7.073403739	-1.142102848
hsa-miR-103a-3p	DKK1	target	1.973244744	-1.142102848
hsa-miR-497-5p	DDX3Y	target	-6.000204056	1.650926795
hsa-miR-424-5p	DDX3Y	target	-2.868822222	1.650926795
hsa-miR-29b-3p	DDX3Y	target	-3.614460994	1.650926795

hsa-miR-29a-3p	DDX3Y	target	-7.710169639	1.650926795
hsa-miR-19b-3p	DDX3Y	target	-5.130085539	1.650926795
hsa-miR-19a-3p	DDX3Y	target	-2.335140461	1.650926795
hsa-miR-199b-5p	DDX3Y	target	-6.704986694	1.650926795
hsa-miR-199a-5p	DDX3Y	target	-7.464223567	1.650926795
hsa-miR-195-5p	DDX3Y	target	-7.895415794	1.650926795
hsa-miR-15b-5p	DDX3Y	target	-7.4728635	1.650926795
hsa-miR-15a-5p	DDX3Y	target	-7.474686522	1.650926795
hsa-miR-101-3p	DDX3Y	target	-4.824183106	1.650926795
hsa-miR-5195-3p	CRISPLD2	target	6.534320478	-1.233301817
hsa-miR-376b-3p	CRISPLD2	target	3.651956789	-1.233301817
hsa-miR-29c-3p	COL6A2	target	21.99464321	-1.093370373
hsa-miR-98-5p	COL4A1	target	-5.885407408	1.354341619
hsa-miR-29b-3p	COL4A1	target	-3.614460994	1.354341619
hsa-miR-29a-3p	COL4A1	target	-7.710169639	1.354341619
hsa-miR-152-3p	COL4A1	target	-5.366644456	1.354341619
hsa-miR-148b-3p	COL4A1	target	-3.844370844	1.354341619
hsa-miR-148a-3p	COL4A1	target	-6.9178194	1.354341619
hsa-let-7f-5p	COL4A1	target	-12.84678967	1.354341619
hsa-let-7e-5p	COL4A1	target	-8.750119272	1.354341619
hsa-let-7c-5p	COL4A1	target	-13.21630578	1.354341619
hsa-let-7a-5p	COL4A1	target	-13.68176841	1.354341619
hsa-miR-98-5p	COL1A1	target	-5.885407408	1.561899014
hsa-miR-29b-3p	COL1A1	target	-3.614460994	1.561899014
hsa-miR-29a-3p	COL1A1	target	-7.710169639	1.561899014
hsa-miR-218-5p	COL1A1	target	-2.920309808	1.561899014
hsa-miR-196b-5p	COL1A1	target	-3.858799039	1.561899014
hsa-miR-196a-5p	COL1A1	target	-6.248512556	1.561899014
hsa-miR-143-3p	COL1A1	target	-2.558538	1.561899014
hsa-let-7i-5p	COL1A1	target	-10.44359602	1.561899014
hsa-let-7g-5p	COL1A1	target	-9.970715511	1.561899014
hsa-let-7f-5p	COL1A1	target	-12.84678967	1.561899014
hsa-let-7e-5p	COL1A1	target	-8.750119272	1.561899014
hsa-let-7c-5p	COL1A1	target	-13.21630578	1.561899014
hsa-let-7b-5p	COL1A1	target	-25.01169358	1.561899014
hsa-let-7a-5p	COL1A1	target	-13.68176841	1.561899014
hsa-miR-502-3p	COL10A1	target	-2.727154031	1.932913349
hsa-miR-101-3p	COL10A1	target	-4.824183106	1.932913349
hsa-miR-497-5p	CNIH3	target	-6.000204056	1.001700646
hsa-miR-424-5p	CNIH3	target	-2.868822222	1.001700646
hsa-miR-195-5p	CNIH3	target	-7.895415794	1.001700646
hsa-miR-15b-5p	CNIH3	target	-7.4728635	1.001700646
hsa-miR-15a-5p	CNIH3	target	-7.474686522	1.001700646
hsa-miR-383-5p	CLEC3A	target	2.153888914	-1.708521224
hsa-miR-23c	CHST10	target	3.754610411	-1.103610816
hsa-miR-24-3p	CHI3L1	target	-8.852555428	1.04702941
hsa-miR-27b-3p	CDH11	target	-8.0156531	1.113361817
hsa-miR-27a-3p	CDH11	target	-7.027874406	1.113361817
hsa-miR-144-3p	CDH11	target	-2.246704806	1.113361817
hsa-miR-128-3p	CDH11	target	-3.2456183	1.113361817
hsa-miR-101-3p	CDH11	target	-4.824183106	1.113361817
hsa-miR-497-5p	CCND1	target	-6.000204056	1.484930221
hsa-miR-424-5p	CCND1	target	-2.868822222	1.484930221

hsa-miR-20b-5p	CCND1	target	-2.910447375	1.484930221
hsa-miR-20a-5p	CCND1	target	-3.760302156	1.484930221
hsa-miR-19b-3p	CCND1	target	-5.130085539	1.484930221
hsa-miR-19a-3p	CCND1	target	-2.335140461	1.484930221
hsa-miR-195-5p	CCND1	target	-7.895415794	1.484930221
hsa-miR-193b-3p	CCND1	target	-4.598413106	1.484930221
hsa-miR-193a-3p	CCND1	target	-1.549934411	1.484930221
hsa-miR-17-5p	CCND1	target	-2.907048831	1.484930221
hsa-miR-15b-5p	CCND1	target	-7.4728635	1.484930221
hsa-miR-15a-5p	CCND1	target	-7.474686522	1.484930221
hsa-miR-142-3p	CCND1	target	-2.005220017	1.484930221
hsa-miR-106b-5p	CCND1	target	-5.687222906	1.484930221
hsa-let-7i-5p	CCND1	target	-10.44359602	1.484930221
hsa-let-7b-5p	CCND1	target	-25.01169358	1.484930221
hsa-miR-29c-3p	C4orf32	target	21.99464321	-1.15013865
hsa-miR-92a-3p	ASPN	target	2.653037772	-1.281810393
hsa-miR-222-3p	ASPH	target	-4.5975339	1.212288754
hsa-miR-221-3p	ASPH	target	-7.122492578	1.212288754
hsa-miR-206	ASPH	target	-3.869800733	1.212288754
hsa-miR-152-3p	ASPH	target	-5.366644456	1.212288754
hsa-miR-148b-3p	ASPH	target	-3.844370844	1.212288754
hsa-miR-148a-3p	ASPH	target	-6.9178194	1.212288754
hsa-miR-142-3p	ASPH	target	-2.005220017	1.212288754
hsa-miR-1-3p	ASPH	target	-3.903586272	1.212288754
hsa-miR-376c-3p	ALCAM	target	-4.708504289	1.001591531
hsa-miR-152-3p	ALCAM	target	-5.366644456	1.001591531
hsa-miR-148b-3p	ALCAM	target	-3.844370844	1.001591531
hsa-miR-148a-3p	ALCAM	target	-6.9178194	1.001591531
hsa-miR-142-3p	ALCAM	target	-2.005220017	1.001591531
hsa-miR-96-5p	AHR	target	-3.69065925	1.096324214
hsa-miR-502-3p	AHR	target	-2.727154031	1.096324214
hsa-miR-29b-3p	AHR	target	-3.614460994	1.096324214
hsa-miR-29a-3p	AHR	target	-7.710169639	1.096324214
hsa-miR-1271-5p	AHR	target	-3.057933239	1.096324214
hsa-miR-98-5p	ADAMTS5	target	-5.885407408	1.28388547
hsa-miR-497-5p	ADAMTS5	target	-6.000204056	1.28388547
hsa-miR-424-5p	ADAMTS5	target	-2.868822222	1.28388547
hsa-miR-195-5p	ADAMTS5	target	-7.895415794	1.28388547
hsa-miR-181d-5p	ADAMTS5	target	-2.538257378	1.28388547
hsa-miR-181c-5p	ADAMTS5	target	-5.975134372	1.28388547
hsa-miR-181b-5p	ADAMTS5	target	-4.229699617	1.28388547
hsa-miR-181a-5p	ADAMTS5	target	-8.086162172	1.28388547
hsa-miR-15b-5p	ADAMTS5	target	-7.4728635	1.28388547
hsa-miR-15a-5p	ADAMTS5	target	-7.474686522	1.28388547
hsa-miR-152-3p	ADAMTS5	target	-5.366644456	1.28388547
hsa-miR-148b-3p	ADAMTS5	target	-3.844370844	1.28388547
hsa-miR-148a-3p	ADAMTS5	target	-6.9178194	1.28388547
hsa-miR-140-5p	ADAMTS5	target	-7.6651035	1.28388547
hsa-let-7i-5p	ADAMTS5	target	-10.44359602	1.28388547
hsa-let-7g-5p	ADAMTS5	target	-9.970715511	1.28388547
hsa-let-7f-5p	ADAMTS5	target	-12.84678967	1.28388547
hsa-let-7e-5p	ADAMTS5	target	-8.750119272	1.28388547
hsa-let-7c-5p	ADAMTS5	target	-13.21630578	1.28388547

hsa-let-7b-5p	ADAMTS5	target	-25.01169358	1.28388547
hsa-let-7a-5p	ADAMTS5	target	-13.68176841	1.28388547
hsa-miR-92a-3p	ACTC1	target	2.653037772	-1.459980616
