# Supplementary material for: CircEYA3 aggravates intervertebral disc degeneration through the miR-196a-5p/EBF1 axis and NF-κB signaling
Source: Commun Biol. 2024 Mar 30;7:390. doi: 10.1038/s42003-024-06055-2 (PMC10981674; doi:10.1038/s42003-024-06055-2)
Supplement: Supplementary file 8 — Reporting Summary [file 42003_2024_6055_MOESM8_ESM.pdf]

Corresponding author(s): Dapeng Feng

Last updated by author(s): Jan 26, 2024

Reporting Summary

Nature Portfolio wishes to improve the reproducibility of the work that we publish. This form provides structure for consistency and transparency in reporting. For further information on Nature Portfolio policies, see our Editorial Policies and the Editorial Policy Checklist.

Statistics

For all statistical analyses, confirm that the following items are present in the figure legend, table legend, main text, or Methods section.

n/a	Confirmed	
☐	☒	The exact sample size (n) for each experimental group/condition, given as a discrete number and unit of measurement
☐	☒	A statement on whether measurements were taken from distinct samples or whether the same sample was measured repeatedly
☐	☒	The statistical test(s) used AND whether they are one- or two-sided<br>*Only common tests should be described solely by name; describe more complex techniques in the Methods section.*
☐	☒	A description of all covariates tested
☐	☒	A description of any assumptions or corrections, such as tests of normality and adjustment for multiple comparisons
☐	☒	A full description of the statistical parameters including central tendency (e.g. means) or other basic estimates (e.g. regression coefficient) AND variation (e.g. standard deviation) or associated estimates of uncertainty (e.g. confidence intervals)
☐	☒	For null hypothesis testing, the test statistic (e.g. F, t, r) with confidence intervals, effect sizes, degrees of freedom and P value noted<br>*Give P values as exact values whenever suitable.*
☐	☒	For Bayesian analysis, information on the choice of priors and Markov chain Monte Carlo settings
☐	☒	For hierarchical and complex designs, identification of the appropriate level for tests and full reporting of outcomes
☐	☒	Estimates of effect sizes (e.g. Cohen's d, Pearson's r), indicating how they were calculated

Our web collection on statistics for biologists contains articles on many of the points above.

Software and code

Policy information about availability of computer code

Data collection	Gene Expression Omnibus (https://www.ncbi.nlm.nih.gov/geo/) and Cistrome Data Browser (cistrome.org/db/#/).
Data analysis	'R' software (www.R-project.org/).

For manuscripts utilizing custom algorithms or software that are central to the research but not yet described in published literature, software must be made available to editors and reviewers. We strongly encourage code deposition in a community repository (e.g. GitHub). See the Nature Portfolio guidelines for submitting code & software for further information.

Data

Policy information about availability of data

All manuscripts must include a data availability statement. This statement should provide the following information, where applicable:

- Accession codes, unique identifiers, or web links for publicly available datasets
- A description of any restrictions on data availability
- For clinical datasets or third party data, please ensure that the statement adheres to our policy

The data that support the findings of this study are available on request from the corresponding author upon reasonable request.

Research involving human participants, their data, or biological material

Policy information about studies with human participants or human data. See also policy information about sex, gender (identity/presentation), and sexual orientation and race, ethnicity and racism.

Reporting on sex and gender	Between October 2019 and March 2022, we procured NP tissues from a cohort of patients who had undergone Transforaminal Lumbar Interbody Fusion (n=33, mean age 66.8 ± 2.1 years, 13 males, 20 females). Additionally, we obtained NP tissues from a group of patients who had undergone percutaneous endoscopic lumbar discectomy without IDD (n=6, mean age 32.7±2.5 years, 4 males, 2 females).
Reporting on race, ethnicity, or other socially relevant groupings	Between October 2019 and March 2022, we procured NP tissues from a cohort of patients who had undergone Transforaminal Lumbar Interbody Fusion (n=33, mean age 66.8 ± 2.1 years, 13 males, 20 females). Additionally, we obtained NP tissues from a group of patients who had undergone percutaneous endoscopic lumbar discectomy without IDD (n=6, mean age 32.7±2.5 years, 4 males, 2 females).
Population characteristics	See Above.
Recruitment	See Above.
Ethics oversight	Ethics Committee of the Second Hospital of Dalian Medical University

Note that full information on the approval of the study protocol must also be provided in the manuscript.

Field-specific reporting

Please select the one below that is the best fit for your research. If you are not sure, read the appropriate sections before making your selection.

☒ Life sciences ☐ Behavioural & social sciences ☐ Ecological, evolutionary & environmental sciences

For a reference copy of the document with all sections, see nature.com/documents/nr-reporting-summary-flat.pdf

Life sciences study design

All studies must disclose on these points even when the disclosure is negative.

Sample size	Between October 2019 and March 2022, we procured NP tissues from a cohort of patients who had undergone Transforaminal Lumbar Interbody Fusion (n=33, mean age 66.8 ± 2.1 years, 13 males, 20 females). Additionally, we obtained NP tissues from a group of patients who had undergone percutaneous endoscopic lumbar discectomy without IDD (n=6, mean age 32.7±2.5 years, 4 males, 2 females).
Data exclusions	See Above.
Replication	See Above.
Randomization	See Above.
Blinding	See Above.

Reporting for specific materials, systems and methods

We require information from authors about some types of materials, experimental systems and methods used in many studies. Here, indicate whether each material, system or method listed is relevant to your study. If you are not sure if a list item applies to your research, read the appropriate section before selecting a response.

Materials & experimental systems

n/a	Involved in the study	
☐	☒	Antibodies
☒	☐	Eukaryotic cell lines
☒	☐	Palaeontology and archaeology
☐	☒	Animals and other organisms
☒	☐	Clinical data
☒	☐	Dual use research of concern
☒	☐	Plants

Methods

n/a	Involved in the study	
☐	☒	ChIP-seq
☐	☒	Flow cytometry
☒	☐	MRI-based neuroimaging

Antibodies

Antibodies used	ab214429, ab32535, ab3778, ab41037, ab307674, ab32561, ab133462, ab76429, ab124957, ab181602, ab32536, ab313636, Abcam, Cambridge, UK.<br>PA5-61136, Invitrogen, USA.<br>18165-1-AP, SA00013-4, SA00013-2, Proteintech, China.
Validation	See above.

Animals and other research organisms

Policy information about studies involving animals; ARRIVE guidelines recommended for reporting animal research, and Sex and Gender in Research

Laboratory animals	In all animal experiments, the Animal Ethics Committee at Dalian Medical University ratified all procedures. The 2011 guidelines for the care and use of laboratory animals were followed during the experiment. The Experimental Animal Center at Dalian Medical University provided the rats. The IDD model was established as described previously. Briefly, Following the administration of anesthesia, the rats were positioned in a supine manner, whereupon the abdominal wall was incised, thereby revealing the omentum and abdominal contents. Concurrently, the surrounding abdominal wall was provided with support. Subsequently, the posterior peritoneum was exposed and incised, while ensuring the preservation of the inferior vena cava and the detachment of the psoas major from the spine. This procedure facilitated the complete exposure and puncturing of the lumbar disc. The annulus fibrosus was then punctured with a syringe and left for 1 minute. Finally, the rat abdomen was sutured layer by layer. A total of 50 nM miR-NC, miR-196a-5p agomir and lentivirus expressing EBF1 or circEYA3 were injected into the lumbar discs of the recipients (n=8 per group). After a period of eight weeks following the surgical procedure, the lumbar region of rats was subjected to scanning and subsequently utilized for further experimental investigations.<br>Histology staining<br>The lumbar region of the rats was fixed in a 4% paraformaldehyde solution for paraffin embedding. Tissue decalcification was performed using EDTA decalcification solution for a duration of two months. The sections were subjected to pretreatment steps, including drying, deparaffinization, and rehydration. Subsequently, the tissues were sliced into 4 μm sections. Safranin O and fast green staining (G1371, Solarbio, China) and H&E staining (G1120, Solarbio, China) were employed to assess the degeneration of the nucleus pulposus and the morphology of the lumbar region.
Wild animals	See above.
Reporting on sex	See above.
Field-collected samples	See above.
Ethics oversight	See above.

Note that full information on the approval of the study protocol must also be provided in the manuscript.

Plants

Seed stocks	none.
Novel plant genotypes	none.
Authentication	none.

ChIP-seq

Data deposition

- [x] Confirm that both raw and final processed data have been deposited in a public database such as GEO.
- [ ] Confirm that you have deposited or provided access to graph files (e.g. BED files) for the called peaks.

Data access links<br>*May remain private before publication.*	cistrome.org/db/#/
Files in database submission	Figure S2
Genome browser session<br>(e.g. UCSC)	UCSC

Methodology

Replicates	n/a
Sequencing depth	n/a
Antibodies	n/a
Peak calling parameters	n/a
Data quality	n/a
Software	n/a

Flow Cytometry

Plots

Confirm that:

- [x] The axis labels state the marker and fluorochrome used (e.g. CD4-FITC).
- [ ] The axis scales are clearly visible. Include numbers along axes only for bottom left plot of group (a 'group' is an analysis of identical markers).
- [ ] All plots are contour plots with outliers or pseudocolor plots.
- [ ] A numerical value for number of cells or percentage (with statistics) is provided.

Methodology

Sample preparation	The apoptosis rate of treated NPCs was determined using a PE Annexin V Apoptosis Detection Kit I (559763, BD Pharmingen, USA). A fluorescence-activated cell sorting (FACS) flow cytometer (BD Biosciences, USA) was then used to analyse the results. In each experiment, three replicates were used.
Instrument	See above.
Software	See above.
Cell population abundance	See above.
Gating strategy	See above.

- [x] Tick this box to confirm that a figure exemplifying the gating strategy is provided in the Supplementary Information.
